# Supplementary material for: The silkworm (Bombyx mori) gut microbiota is involved in metabolic detoxification by glucosylation of plant toxins
Source: Commun Biol. 2023 Jul 29;6:790. doi: 10.1038/s42003-023-05150-0 (PMC10387059; doi:10.1038/s42003-023-05150-0)
Supplement: Supplementary file 2 — Supplementary Information [file 42003_2023_5150_MOESM2_ESM.pdf]

Supplementary Information

The silkworm (*Bombyx mori*) gut microbiota is involved in metabolic detoxification by glucosylation of plant toxins

Shuangzhi Yuan[1], Yong Sun[1], Wenqiang Chang[1], Jiaozhen Zhang[1], Jifa Sang[2], Jiachun Zhao[2], Minghui Song[1], Yanan Qiao[1], Chunyang Zhang[1], Mingzhu Zhu[1], Yajie Tang[3], and Hongxiang Lou[1]*

[1]Department of Natural Products Chemistry, Key Laboratory of Chemical Biology of the Ministry of Education, School of Pharmaceutical Sciences, Shandong University, Jinan 250012, P. R. China
[2] Linyi University, Yishui, Linyi 276400, P. R. China
[3] State Key Laboratory of Microbial Technology, Shandong University, Qingdao 266237, P. R. China
Email: louhongxiang@ sdu.edu.cn

Supplementary Note 1: The structural elucidation of compounds 1-21 for details

Structure Elucidation of Compounds 1-21

Silexcrin A (**1**) was obtained as off-white solid and assigned the molecular formula $C_{37}H_{46}O_{16}$ based on the HRESIMS (*m/z* 745.27228 $[M-H]^-$, calcd 745.27131) and ^{13}C NMR data, displaying fifteen degrees of unsaturation. The IR spectrum with the absorption bands at 3364, 2967, 2913, 1640, 1581, 1508 and 1435 cm^{-1} implied the presence of hydroxyl, methyl and benzene moiety. The ^{1}H NMR data (Supplementary Table 1) included signals at δ_H 8.54 (1H, s, H-2), a characteristic ABX system at δ_H 6.96 (1H, dd, *J*=2.0, 8.5 Hz, H-6'), 7.08 (1H, d, *J*= 2.0 Hz, H-2'), 7.19(1H, d, *J*=8.5 Hz, H-5'), and hydroxyls in the downfield at δ_H 8.71 (1H, s, 3'-OH) and δ_H 13.11 (1H, s, 5-OH). Analysis of the ^{13}C NMR (Supplementary Table 2) and HMQC data (Supplementary Figure 7) of **1** indicated 37 carbon resonances, corresponding to four methyl (δ_C 25.52, 25.48, 18.05, and 18.03), six sp^2 methines (δ_C 155.3, 122.8, 122.7, 120.1, 116.8, and 116.6), ten sp^3 methines (δ_C 104.8, 102.3, 77.5, 77.3, 76.5, 75.9, 74.0, 73.4, 69.87, and 69.86), four sp^3 methylene (δ_C 61.0, 60.8, 22.5 (C-1''), 22.5 (C-1''')), twelve quaternary carbons (δ_C 158.1, 156.9, 152.7, 146.5, 145.4, 143.0,131.3, 130.7, 125.4, 122.1, 118.3, 113.8, and 108.0), and one conjugated carbonyl carbon (δ_C 181.1). It was preliminarily confirmed as an isoflavone glycoside.

According to ^{1}H and ^{13}C NMR, there were presence of two typical prenyl groups, 3,3-dimethylallyl with the signals at δ_H [1.63 (3H, s, H-4''), 1.74 (3H, s, H-5''), 3.41, 3.60 (2H, m, H-1''), and 5.19 (1H, m, H-2'')], δc [130.7 (C-3''), 122.8 (C-2''), 22.5 (C-1''), 25.48 (C-4''), and 18.03 (C-5'')], and at δ_H [1.64 (3H, s, H-4'''), 1.78 (3H, s, H-5''') 3.50, 3.72 (2H, m, H-1'''), and 5.19 (1H, m, H-2''')], δ_C [131.3 (C-3'''), 122.7 (C-2'''), 25.52 (C-4'''), 22.5 (C-1'''), and 18.05 (C-5''')]. The signals of prenyl groups in 2D NMR data: two spin systems, CH_2 (1'')-CH (2'') and CH_2 (1''')-CH (2''') were identified by ^{1}H-^{1}H COSY spectrum (Supplementary Figure 8). And the HMBC correlation signals (Supplementary Figure 9), from H-1'' (δ_H 3.41, 3.60, m) to C-5 (δ_C 156.9), C-6 (δ_C 118.3), C-7 (δ_C 158.1), C-2''(δ_C 122.8), C-3''(δ_C 130.7); H-1''' (δ_H 3.50, 3.72, m) to C-7 (δ_C 158.1), C-8 (δ_C 113.8), C-9 (δ_C 152.7), C-2'''(δ_C 122.7), C-3'''(δ_C 131.8), further proved two prenyl groups attached at C-6 and C-8. Additionally, compared with the previous report[1], the aglycone moiety was 6,8-diprenylorobol. Moreover, the ^{1}H NMR spectrum also displayed two anomeric protons with doublets at δ_H 4.68 (d, *J*=7.6 Hz, 1H), 4.78 (d, *J*=7.3 Hz, 1H) and the other protons in sugar moiety at 2.8-3.8 ppm. There were two anomeric carbons at δ_C 102.3, 104.8 and others located at 60.8-77.5 ppm in ^{13}C NMR spectrum. The aforementioned data suggested the sugar moieties were β-glucoses. Based on the HMBC data, two anomeric protons with correlation to C-7 (δ_C 158.1), C-4' (δ_C 145.4) were indicated that the sugar moieties were conjugated at the positions of 7, 4'-hydroxyl. Thus, the structure of **1** were confirmed to be 6,8-diprenylorobol-7,4'-di-*O*-β-D-glucoside, named as Silexcrin A.

Silexcrin B (**2**) was confirmed the molecular formula as $C_{37}H_{46}O_{16}$ according to its HRESIMS at *m/z* 745.27179 $[M-H]^-$ (calcd 745.27131). The 1D NMR (Supplementary Table 1-2) of compound **2** exhibited similar spectral data to **1**, except for the protons resonance signals in an ABX system at δ_H 6.89 (1H, d, *J*=8.3 Hz, H-5'), 7.17 (1H, dd, *J*=1.9, 8.3 Hz, H-6'), 7.34 (1H, d, *J*=1.9 Hz, H-2') of **2**. It implied that compound **2** was a prenylated isoflavone glycoside with an aglycone moiety of 6,8-diprenylorobol, and two β-glucoses with two anomeric protons and carbon signals at δ_H 4.68 (d,

J=7.6 Hz, 1H), δ_C 104.8, and δ_H 4.77 (d, J=7.3 Hz, 1H), δ_C 102.2. Another difference between the two was that the cross peaks in HMBC (Supplementary Figure 19) between the anomeric proton at δ_H 4.68 and C-7 (δ_C 158.1), δ_H 4.77 and C-3' (δ_C 145.0). It further provided evidence for the attachment of glucose moieties at the 7, 3'-hydroxyl positions. Thus, the compound **2** was confirmed as 6,8-diprenylorobol-7,3'-di-*O*-β-D-glucoside, named as Silexcrin B.

Silexcrin C (**3**), obtained as pale-yellow powder, was corresponded to molecular formula $C_{31}H_{36}O_{11}$ according to the HRESIMS *m/z* 583.21851 [M-H]$^-$ (calcd 583.21849) with fourteen unsaturation degrees. The 1D NMR spectroscopic data (Supplementary Tables 1-2) were close to **1**, showing only one β-glucose with an anomeric proton and carbon at δ_H 4.68(d, J=7.8 Hz) and δ_C 104.8, attached to the aglycone moiety of 6,8-diprenylorobol and three free hydroxyls at δ_H 9.00 (1H, s, 3'-OH), 9.07 (1H, s, 4'-OH), and 13.16 (1H, s, 5-OH) in the ^{1}H NMR spectrum of **3**. The anomeric proton (δ_H 4.68, d, J=7.8 Hz) in sugar moiety was related with C-7 (δ_C 158.1) in the HMBC correlations (Supplementary Figure 29). Thus, it was identified the molecular structure as 6,8-diprenylorobol-7-*O*-β-D-glucoside, named as Silexcrin C.

Silexcrin D (**4**) was obtained as pale-yellow solid with the molecular formula $C_{31}H_{36}O_{11}$ based on the HRESIMS *m/z* 583.21912 [M-H]$^-$ (calcd 583.21849) and ^{13}C NMR data containing fourteen degrees of unsaturation. The 1D NMR spectroscopic data of **4** (Supplementary Tables 1-2) indicated its structure closely resembled to 3, but distinct signals in ^{1}H and ^{13}C NMR displaying an anomeric proton and carbon δ_H 4.73 (d, J=7.3 Hz) and δ_C 102.3. The HMBC correlation (Supplementary Figure 39) showed an anomeric proton (δ_H 4.73, d, J=7.3 Hz) in glucosyl residue related to C-4' (δ_C 145.2), suggesting the 4'-hydroxyl in aglycone moiety substituted by a glucose. Thus, it was verified the molecule of **4** as 6,8-diprenylorobol-4'-*O*-β-D-glucoside, named as Silexcrin D.

Silexcrin E (**5**), as pale-yellow solid, was shown the molecular formula as $C_{31}H_{36}O_{11}$ combined with the HRESIMS *m/z* 583.21832 [M-H]$^-$ (calcd 583.21849) assuming the same degrees unsaturation of **3**. Comparison of the 1D NMR data (Supplementary Tables 1-2) of **3** and **5**, it indicated the closer structure of **3** and **5**. The differences uncovered in ^{1}H NMR data of **5** appeared a distinct ABX system at δ_H 7.32 (1H, d, J= 2.0 Hz, H-2'), 6.88 (1H, d, J=8.30 Hz, H-5'), 7.15 (1H, dd, J=2.0, 8.3 Hz, H-6'), a different anomeric proton δ_H 4.76 (d, J=7.6 Hz), and an obvious hydroxyl signal at δ_H 8.77 (s, 4'-OH). On the basis of the HMBC data (Supplementary Figure 49), an anomeric proton (δ_H 4.76, d, J=7.60 Hz) had correlation to C-3' (δ_C 144.9), thus the molecule structure of **5** was confirmed as 6,8-diprenylorobol-3'-*O*-β-D-glucoside, named as Silexcrin E.

Silexcrin F (**6**) was as off-white solid and established its molecular formula as $C_{37}H_{46}O_{15}$ based on the HRESIMS *m/z* 729.27808 [M-H]$^-$ (calcd 729.27639) with fifteen degrees of unsaturation. Its IR exhibited the characteristic absorption peak at 3420, 2964, 2916, 2873, 1641, 1610, 1583, 1450 cm^{-1}, implying the presence of hydroxyl, methyl, and benzene ring.

The ^{1}H NMR spectra data (Spplementary Table 3) showed a downfield signal δ_H 8.54 (1H, s, H-2), an obvious AA'BB' system at δ_H 7.11(2H, d, *J*=8.5 Hz, H-3', H-5'), 7.52 (2H, d, *J*=8.7 Hz, H-2', H-6'), two prenyl groups at δ_H [1.63 (3H, s, H-4''), 1.74 (3H, s, H-5''), 3.36, 3.59 (2H, m, H-1''), and 5.18 (2H, m, H-2'')]; [1.64 (3H, s, H-4'''), 1.78 (3H, s, H-5'''), 3.48, 3.71 (2H, m, H-1'''), and 5.18 (H, m, H-2''')], and a hydroxyl at δ_H 13.09 (1H, s, 5-OH). Its ^{13}C NMR (Supplementary Table 4) and HSQC spectrum (Supplementary Figure 57) of **6** showed four methyls (δ_C 25.50, 25.47, 18.03, and 18.02), seven sp^2 methines (δ_C 155.3, 130.1(C-2', C-6'), 122.7, 122.6, and 116.1(C-3', C-5')), ten sp^3 methines (δ_C 104.8, 100.3, 77.5, 77.1, 76.6, 76.4, 74.0, 73.2, 69.9, and 69.7), four sp^3 methylene (δ_C 61.0, 60.7, 22.5(C-2'', C-2''')), eleven quaternary carbons (δ_C 158.1, 157.3, 156.9, 152.8, 131.2, 130.7, 124.2, 122.0, 118.3, 113.8, and 108.0), and one carbonyl carbon (δ_C 181.1). It revealed that it was a prenylated isoflavone glycoside according to the above data.

The prenyl units with two spin systems, CH_2 (1'')-CH (2'') and CH_2 (1''')-CH (2''') showed in ^{1}H-^{1}H COSY spectrum (Supplementary Figure 58), and were placed at C-6, C-8, due to the HMBC correlation (Supplementary Figure 59) of H-1'' (δ_H 3.36, 3.59 2H, m) with C-6 (δ_C 118.3), and H-1''' (δ_H 3.36, 3.59 2H, m) with C-8 (δ_C 113.8). The 1D NMR spectra were also compared with those of previous reports[2], revealing the aglycone as 6,8-diprenylgenistein. According to the two anomeric protons and carbons at δ_H 4.68 (d, *J*=7.6 Hz) and δ_C 104.8, δ_H 4.92 (d, *J*=7.2 Hz) and δ_C 102.3 of **6**, the two sugar moieties were *β*-glucoses. They were attached to the 7,4'-hydroxyls in the 6,8-diprenylgenistein aglycone on the basis of the HMBC correlation of anomeric protons δ_H 4.68(d, *J*=7.6 Hz) with C-7 (δ_C 158.1) and δ_H 4.92 (d, *J*=7.2 Hz) with C-4' (δ_C 157.3). The molecule of **6** was 6,8-diprenylgenistein-7,4'-di-*O*-*β*-D-glucoside, named as Silexcrin F.

Silexcrin G (**7**) was obtained as pale-yellow solid, and established the molecular formula as $C_{31}H_{36}O_{10}$ with fourteen degrees of unsaturation based on a deprotonated molecular ion [M-H]$^-$ peak at *m/z* 567.22455 (calcd 567.22357) in the HRESIMS. The ^{1}H and ^{13}C NMR spectral data (Supplementary Tables 3-4) of **7** were comparable with **6**. The ^{1}H and ^{13}C NMR data of **7** showed only one anomeric proton and carbon NMR data at δ_H 4.67 (d, *J*=7.7 Hz) and δ_C 104.8. On the basis of the HMBC correlation of an anomeric proton with C-7 (δ_C 158.1), it was indicated a glucose was attached to the 7-hydroxyl in 6,8-diprenylgenistein as the aglycone moiety of **7**. Thus, the structure of **7** was 6,8-diprenylgenistein-7-*O*-*β*-D-glucoside, and named as Silexcrin G.

Silexcrin H (**8**), was gained as pale-yellow solid, shown a deprotonated molecular ion peak at *m/z* 567.22458 [M-H]$^-$ (calcd 568.2308) based on the HRESIMS and ^{13}C NMR data, in accordance with the molecular formula $C_{31}H_{36}O_{10}$. The ^{1}H and ^{13}C NMR spectra data (Supplementary Tables 3-4) of **8** were almost the same with **7**, except that the anomeric proton and carbon in sugar moiety at δ_H 4.92 (d, *J*=6.9 Hz) and δ_C 100.3. The HMBC correlation of anomeric protons δ_H 4.92 (d, *J*=6.9 Hz) with C-4' (δ_C 157.2) was indicated that the molecule of **8** was 6,8-diprenylgenistein-4'-*O*-*β*-D-glucoside, named as Silexcrin H.

Silexcrin I (**9**) was identified as off-white solid with a deprotonated molecular ion peak at *m/z* 513.17749 $[M-H]^-$ (calcd 513.17662) in the HRESIMS, corresponding to the molecular formula as $C_{27}H_{30}O_{10}$. The ^{1}H and ^{13}C NMR spectra data (Supplementary Tables 3-4) of **9** were similar to **8** as an isoflavone glycoside. But obvious distinction in 1D NMR data of **9** displayed only one prenyl group, δ_H [1.62 (3H, s, H-4"), 1.75 (3H, s, H-5"), 3.23,3.44 (2H, m, H-1"), and 5.23 (H, m, H-2")], δ_C [130.8 (C-3"), 121.9 (C-2"), 25.5 (C-4"), 21.2 (C-1"), and δ_C 17.7 (C-5"), a downfield signal at δ_H 6.81 (H, s, H-8) and δ_C 93.2 (C-8), and the presence of a methoxyl group δ_H 3.8 (3H, s) and δ_C 55.2. The HMBC correlation of the methoxy hydrogen δ_H 3.8 (3H, s) with C-4' (δ_C 159.2), H-1" with C-5 (δ_C 158.0), C-6 (δ_C 112.8), C-7 (δ_C 160.8) was indicated the methoxyl and prenyl group were located at C-4' and C-6, respectively. It showed the aglycone moiety was Gancaonin A [3, 4] and the sugar was β-glucose, thus the molecule of **9** was Gancaonin A -7-*O*-β-D-glucoside, named as Silexcrin I.

Silexcrin J (**10**) was identified as pale-yellow solid with *m/z* 729.27814 $[M-H]^-$ (calcd 729.27639) by the HRESIMS and ^{13}C NMR spectrum, in consistent to the molecular formula as $C_{37}H_{46}O_{15}$. Analysis of the ^{1}H NMR data (Supplementary Table 3) of **10** displayed a ABX system δ_H 7.15 (1H, d, *J*=8.3 Hz, H-5'), 7.32 (1H, d, *J*= 2.0 Hz, H-2'), 7.34 (1H, dd, *J*=2.0, 8.3 Hz, H-6') in benzene ring, two prenyl groups δ_H [1.62 (3H, s, H-4"), 1.75 (3H, s, H-5"), 3.22, 3.45 (2H, m, H-1"), and 5.22 (H, m, H-2")]; δ_H [1.68 (3H, s, H-4'''), 1.70 (3H, s, H-5'''), 3.30, 3.42 (2H, m, H-1'''), and 5.34 (H, m, H-2''')], other downfield signals at δ_H 6.80 (H, s, H-8), 8.44 (H, s, H-2), and the presence of two anomeric protons signals at δ_H 4.85 (d, *J*=7.5 Hz), 5.03 (d, *J*=7.5 Hz). The ^{13}C NMR data (Supplementary Table 4) of **10** appeared the key carbon signals, two prenyl groups at δ_C [130.9 (C-3"), 122.0 (C-2"), 25.56 (C-4"), 21.3 (C-1"), 17.78(C-5")], δ_C [131.5 (C-3'''), 122.7 (C-2'''), 28.2 (C-1'''), 25.61 (C-4'''), 17.79(C-5''')], δ_C 154.9 (C-2), δ_C 93.2 (C-8), and two anomeric carbon in sugar moiety at δ_C 100.4, 101.2.The forementioned data of **10** in contrast to a previous report[5] indicated it was a prenylated isoflavone glycoside with 5,7,4'-trihydroxy-6,3'-diprenylisoflavone as the aglycone and β-glucose as the sugar moiety. The HMBC correlation of anomeric protons in sugar moiety at δ_H 5.03 (d, *J*=7.5 Hz) with C-7 (δ_C 160.8), and δ_H 4.85 (d, *J*=7.50 Hz) with C-4' (δ_C 155.2) were indicated glucoses were located at 7,4'-hydroxyls in aglycone moiety. Thus, compound **10** was 5-hydroxy-6,3'-diprenylisoflavone-7,4'-di-*O*-β-D-glucoside, named as Silexcrin J.

Silexcrin K (**11**) was as pale-yellow solid and identified with *m/z* 567.22400 $[M-H]^-$ (calcd 567.22357) by the HRESIMS and ^{13}C NMR spectrum, showing the molecular formula in consistence with $C_{31}H_{36}O_{10}$. The ^{1}H and ^{13}C NMR spectra data (Supplementary Table 3-4) of **11** in contrast to those of **10** indicated both were prenylated isoflavone glycoside with similar structure, expect that the absence of an anomeric signal in sugar moiety of **11**. Analysis of the HMBC correlation revealed that the presence of only one anomeric proton in sugar at δ_H 5.03 (d, *J*=7.3 Hz) with C-7 (δ_C 160.7) existed correlation. It indicated a β-glucose linked to 7-hydroxyl in aglycone moiety, the molecule of (**11**) was 5,4'-dihydroxy-6,3'-diprenylisoflavone-7-*O*-β-D-glucoside, named as Silexcrin K.

Silexcrin L (**12**) was identified as pale-yellow solid with *m/z* 729.27777 $[M-H]^-$ (calcd 729.27639) by the HRESIMS, as an isomeride of **11** with the same molecular formula as $C_{37}H_{46}O_{15}$. Analysis of the 1D NMR spectral data of **11** and **12** (Supplementary Tables 7-8) exihibited both of the data were almost same, expect that the signals at δ_H 6.63 (H-6, s) and δ_C 98.2 (C-6) of **12**, but at δ_H 6.80 (H-8, s) and δ_C 93.2 (C-8) of **11**, indicating the main difference between the two molecules was the linkage position of a prenyl group in A ring. The HMBC correlation signals of **12** demonstrated that protons at H-1'' (δ_H 3.35, 3.58, m, 2H) in prenyl group were related to C-7 (δ_C 160.5), C-8 (δ_C 108.2), C-9 (δ_C 154.0), revealing the prenyl group located at C-8 in A-ring. Thus, the molecule of **12** was 5-hydroxy-8,3'-diprenylisoflavone-7,4'-di-*O*-*β*-D-glucoside, named as Silexcrin L.

Silexcrin M (**13**) was obtained as a pale-yellow solid with the molecular formula as $C_{31}H_{36}O_{12}$ based on the HRESIMS (*m/z* 599.21411 $[M-H]^-$, calcd 599.21340) and ^{13}C NMR spectrum with fourteen degrees of unsaturation. The ^{1}H NMR data (Supplementary Table 5) of **13** appeared a deshielded proton signal at δ_H 8.43 (H, s, H-2), an ABX system in benzene ring at δ_H [6.88 (1H, d, *J*=8.3 Hz, H-5'), 7.17 (1H, dd, *J*=1.9, 8.3 Hz, H-6'), 7.34 (1H, d, *J*= 1.9 Hz, H-2')], hinting that the structure of **13** contained an isoflavone skeleton. Besides, two prenyl substituents included the ^{1}H and ^{13}C NMR (Supplementary Table 6) signals in 2-(1-hydroxy-1-methylethyl) dihydrofuran ring at δ_H [1.11 (3H, s, H-5''), 1.17 (3H, s, H-4''), 3.12 (2H, d, *J*=8.4 Hz, H-1''), and 4.75 (1H, t, *J*=7.4 Hz, H-2'')], and δ_C [90.1 (C-2''), 70.2 (C-3''), 26.3 (C-1''), 26.1 (C-4''), and 24.2 (C-5'')]; and in 3,3-dimethylallyl at δ_H [1.64 (3H, s, H-4''') 1.75 (3H, s, H-5''') 3.31 (2H, d, *J*=7.3 Hz, H-1'''), and 5.20 (1H, t, *J*=7.3 Hz, H-2''')], and δ_C [131.6 (C-3'''), 121.4 (C-2'''), 25.4 (C-4'''), 21.5 (C-1'''), and 17.6 (C-5''')]. Based on the anomeric proton and carbon signals in 1D NMR spectra and others' signals at δ_H 4.76 (d, *J*=7.1 Hz) and δ_C 102.2, at δ_H 3.0-3.8 (m, 5H) and δ_C 77.2, 69.9, 76.0, 73.4, 60.8, suggested the sugar moiety was *β*-glucose. According to the HMBC data (Supplementary Figure 129), the correlation of protons in prenyl groups from H-1''(δ_H 3.12, d, *J*=8.4 Hz) to C-6 (δ_C 108.7), and from H-1''' (δ_H 3.31, d, *J*=8.4 Hz) to C-8 (δ_C 101.2), implied the substituent positions at C-6 and C-8, respectively. In addition, the correlation of anomeric proton in sugar at δ_H 4.76 (d, *J*=7.1 Hz) with C-3' (δ_C 145.0) determined a sugar was attached to 3'-hydroxyl in aglycone moiety. Analysis of the ^{1}H and ^{13}C NMR forementioned data of **13** indicated that it was a prenylated isoflavone glucoside with the aglycone of Furowanin A, in contrast to previous report[6] and with the *β*-glucose moiety.

According to the structure of the aglycone moiety of **13**, it contained a dihydrofuran-ring bearing a 1-hydroxy-1-methylethyl moiety at C-2'' formed by the cyclization of 3,3-dimethylallyl side chains at C-6 and 7-hydroxyl, and exposed a chiral center at C-2'', which configuration was not determined. Due to the configuration of glucose moiety being beta (*β*), a chiral center at C-2'' confirmed its configuration as *R* by calculated ECD. The absolute configuration of **13** confirmed as (*2''R*)-Furowanin A-3'-*O*-*β*-D-glucoside, named as Silexcrin M.

Silexcrin N (**14**) was isolated as a pale-yellow solid with $C_{31}H_{36}O_{12}$ given by the HRESIMS (*m/z* 599.21501 $[M-H]^-$, calcd 599.21340). Analysis of the ^{1}H and ^{13}C NMR spectra data of **13** and **14** (Supplementary Tables 5-6) were discovered with the same spectra data. It suggested that **13** possessed same planar configuration as **14**, as an isoflavone glucoside with the aglycone of

Furowanin A and the *β*-glucose moiety. The chiral center at C-2'' confirmed its configuration as *S* by calculated ECD, revealing that **13** and **14** were a pair of epimers. The absolute configuration of **14** was confirmed as (*2'' S*)-Furowanin A-3'-*O*-*β*-D-glucoside, named as Silexcrin N.

Silexcrin O (**15**) was obtained as pale-yellow solid with the molecular formula as $C_{31}H_{36}O_{12}$ based on the HRESIMS (*m/z* 599.21484 $[M-H]^-$, calcd 599.21340) and ^{13}C NMR spectrum containing fourteen unsaturation degrees. The ^1H and ^{13}C NMR spectra data (Supplementary Tables 5-6) of **15** resembled those of **13** and **14**, except that the proton signals in 3,3-dimethylallyl of **15** at δ_H [δ_H 1.63 (s, H-4''), δ_H 1.72 (s, H-5''), 3.18 (d, *J*=7.3 Hz, H-1''), and δ_H 5.12 (t, *J*=7.3 Hz, H-2'')], and in 2-(1-hydroxy-1-methylethyl) dihydrofuran ring at δ_H [3.24 (d, *J*=8.2 Hz, H-1'''), 4.78 (t, *J*=8.2 Hz, H-2'''), 1.18 (s, H-4'''), 1.12 (s, H-5''')]. Based on the HMBC spectrum (Supplementary Figure 149): from H-1'' (δ_H 3.18, d, *J*=7.3 Hz) in 2-(1-hydroxy-1-methylethyl) dihydrofuran ring to C-5 (δ_C 159.0), C-6 (δ_C 106.3), and C-8 (δ_C 164.6); from H-1''' (δ_H 3.24, d, *J*=8.2 Hz) in 3,3-dimethylallyl to C-6 (δ_C 164.4), C-8 (δ_C 103.0), and C-9 (δ_C 150.3), it determined the substituent positions of prenyl groups at C-6 and C-8, respectively.

And then, combined with the forementioned and publish data[7], it was a glucoside with Furowanin B as the aglycone, containing a chiral center at C-2''' formed by the cyclization of 3,3-dimethylallyl side chains at C-8 and 7-hydroxyl, and with a *β*-glucose moiety. The chiral center at C-2''' confirmed as *S*-configuration by calculated ECD. The absolute configuration of **15** confirmed as (*2'''S*)-Furowanin B-3'-*O*-*β*-D-glucoside, named as Silexcrin O.

Silexcrin P (**16**) was assigned the molecular formula as $C_{31}H_{36}O_{12}$ on the basis of the HRESIMS *m/z* 599.21405 $[M-H]^-$ (calcd 599.21340). The ^1H and ^{13}C NMR spectra data of **15** and **16** (Supplementary Tables 5-6) showed the same spectra data, indicating the two compounds with same planar configuration with *β*-glucose and a chiral center at C-2''' in Furowanin B aglycone. The configuration of chiral C-2''' was established as *R*-type by calculated ECD, uncovering molecules of **15** and **16** were a pair of epimers. The absolute configuration of **16** was confirmed as (*2''' R*)-Furowanin B-3'-*O*-*β*-D-glucoside, named as Silexcrin P.

Silexcrin Q (**17**) was obtained as a pale-yellow solid with the molecular formula as $C_{26}H_{28}O_{11}$ by HRESIMS *m/z* 515.15739 $[M-H]^-$ (calcd 515.15588) and ^{13}C NMR spectrum. The 1D NMR spectra data of **9** and **17** (Supplementary Tables 7-8) displayed similar data, except for an ABX system at δ_H [6.80(1H, d, *J*=8.1 Hz, H-5'), 6.82 (1H, dd, *J*=1.9, 8.1 Hz, H-6'), 7.02 (1H, d, *J*= 1.9 Hz, H-2')] in C ring. And compared to the previous data[8], the structure of **17** was confirmed as 6-C-prenylluteolin-7-*O*-*β*-D-glucoside, named as Silexcrin Q.

Silexcrin R (**18**) was given the molecular formula as $C_{27}H_{30}O_{11}$ on the basis of the HRESIMS *m/z* 529.17206 $[M-H]^-$ (calcd 529.17153). The 1D NMR spectra data of **17** and **18** (Supplementary Tables 7-8) displayed closely, except for the presence of a methoxy protons at δ_H 3.80 (3H, s) of **18**. Based on the HMBC correlation, the methoxy protons at δ_H 3.80 had the correlation with C-4' (δ_C

147.8). Thus, compound **18** was confirmed as 4'-methoxyl-6-C-prenylluteolin-7-*O*-*β*-D-glucoside, named as Silexcrin R.

Silexcrin S (**19**) was determined the molecular formula ($C_{26}H_{28}O_{11}$) based on the HRESIMS *m/z* 515.15680 $[M-H]^-$ (calcd 515.15588) and ^{13}C NMR spectrum with thirteen indices of hydrogen deficiency. The 1D NMR spectral data of **17** and **19** (Supplementary Tables 5-6) resembled, except for these signals at δ_H 6.62 (s, H-6) and δ_C 98.2 (C-6) in the ^1H and ^{13}C NMR spectra of **19**. According to the HMBC correlation, the prenyl protons at δ_H 3.34, 3.54 (m, H-1'') had the correlation with C-7 (δ_C 160.4), C-8 (δ_C 108.1), C-9 (δ_C 154.0), and combined with a previous report[4], thus the molecule structure of (**19**) was determined as Gancaonin L-7-*O*-*β*-D-glucoside, named as Silexcrin S.

Silexcrin T (**20**), was obtained as a pale-yellow solid with the molecular formula as $C_{28}H_{32}O_{11}$ by the HRESIMS *m/z* 543.18762 $[M-H]^-$ (calcd 544.1945). There were similar structures through analysis of the 1D NMR spectra between **17** and **20**, except for the presence of these signals at δ_H 2.86 (2H, t, *J*=6.5 Hz, H-1''), 3.62 (2H, t, *J*=6.5 Hz, H-2''), and δ_C 25.8 (C-1''), 60.7 (C-2'') in the ^1H and ^{13}C NMR spectra (Supplementary Tables 7-8) of **20**, impiying the presence of a hydroxyethyl. According to a spin system, CH_2 (1'')-CH_2 (2'') identified by ^1H–^1H COSY spectrum (Supplementary Figure 198) and δ_H 3.62 (H-2'') and δ_C 60.7 (C-2''), we could also determine the hydroxyethyl moiety. The HMBC correlation of the protons H-1'' (δ_H 2.86, t, *J*=6.50Hz,) possessed the correlation signals with C-5 (δ_C 157.6), C-6 (δ_C 110.2), C-7 (δ_C 160.7), suggesting the hydroxyethyl located at C-6. Through comparison with previous report data[9], the aglycone moiety was confirmed as lupiwighteone with hydroxyethyl substituent at C-6. Thus, the molecule structure of **20** was determined as 6-hydroxyethyl lupiwighteone-4'-*O*-*β*-D-glucoside, named as Silexcrin T.

Silexcrin U (**21**), as a pale-yellow solid, was assigned its $[M-H]^-$ *m/z* 583.21899 (calcd 583.21849) consistent with the molecular formula $C_{31}H_{36}O_{11}$ on the basis of HRESIMS and ^{13}C NMR spectrum. Compounds **8** and **21** showed similar structure moiety through comparison of the 1D NMR spectral data (Supplementary Tables 7-8), indicating it was a prenylated isoflavone glycoside with a *β*-glucose. The major difference signals displayed at δ_H [1.76 (3H, s, H-5'''), 2.88, 2.97 (2H, m, H-1''), 4.27 (H, m, H-2''), 4.75(H, s, H-4*α*''), and 4.78(H, s, H-4*β*'')], and δ_C [146.9 (C-3''), 110.1 (C-4''), 74.6 (C-2''), 28.9 (C-1''), and 18.1 (C-5'')] in the 1D NMR spectra of **21**, indicating it also contained a 3-hydroxy-methylbut-1-enyl group, rather than two 3,3-dimethylallyl substituents. Based on the HMBC correlation (Figure S211), from H-1'' (δ_H 2.88, 2.972, m) to C-5 (δ_C 157.2), C-6 (δ_C 109.2), and C-7 (δ_C 160.7), it indicated that the prenyl group of 3-hydroxy-methylbut-1-enyl was substituent at C-6. By comparing with the published data[6], the aglcone moiety of **21** was consistent with isoerysenegalensein E. Thus, the molecule of **21** was verified as isoerysenegalensein E-4'-*O*-*β*-D-glucoside, named as Silexcrin U.

There were 21 compounds, Silexcrins A-U (**1-21**) undescribed before, and 10 known compounds, lupiwighteone-7-*β*-D-glucoside (**22**)[10], genisteone (**23**)[11], 6,8-diprenylorobol (**24**)[1], lupalbigenin (**25**)[12], isolupalbigenin (**26**)[13], auriculasin (**27**)[1, 8], 4'-*O*-methylerythrinin C (**28**)[14], lupiwighteone (**29**)[9], erysenegalensein E (**30**)[15], millewanins H (**31**)[7], isoerysenegalensein E (**32**)[6], millewanins G (**33**)[7] published in previous reports.

Supplementary Note 2: Physicochemical properties of compounds 1-21.

Silexcrin A (**1**), off-white solid, $[\alpha]^{20}_{D}$ -83.1 (*c* 0.1 MeOH); UV (MeOH) λ_{max} (log ε) 265 (4.57) nm; ECD (MeOH) 224 ($\Delta\varepsilon$ -9.29), 268 ($\Delta\varepsilon$ -5.68) nm; IR ν_{max} 3364, 2967, 2913, 1641, 1581, 1508, 1435 cm^{-1}; ^{1}H and ^{13}C NMR spectral data seen in Table S1 and S2; HRESIMS *m/z* 745.27228 $[M-H]^-$ (calcd for $C_{37}H_{45}O_{16}$ 745.27131).

Silexcrin B (**2**), off-white solid, $[\alpha]^{20}_{D}$ -59.9 (*c* 0.1 MeOH); UV (MeOH) λ_{max} (log ε) 265 (4.57) nm; ECD (MeOH) 220 ($\Delta\varepsilon$ -7.96), 268 ($\Delta\varepsilon$ -6.07) nm; IR ν_{max} 3325, 2921, 1642, 1586, 1521, 1435 cm^{-1}; ^{1}H and ^{13}C NMR spectral data seen in Table S1 and S2; HRESIMS *m/z* 745.27179 $[M-H]^-$ (calcd for $C_{37}H_{45}O_{16}$ 745.27131).

Silexcrin C (**3**), pale-yellow solid, $[\alpha]^{20}_{D}$ -66.8 (*c* 0.1 MeOH); UV (MeOH) λ_{max} (log ε) 265 (4.50) nm; ECD (MeOH) 230 ($\Delta\varepsilon$ -7.32), 268 ($\Delta\varepsilon$ -4.62) nm; IR ν_{max} 3361, 2909, 1639, 1610, 1580, 1519, 1435 cm^{-1}; ^{1}H and ^{13}C NMR spectral data seen in Table S1 and S2; HRESIMS *m/z* 583.21851 $[M-H]^-$ (calcd for $C_{31}H_{35}O_{11}$ 583.21849).

Silexcrin D (**4**), pale-yellow solid, $[\alpha]^{20}_{D}$ -78.0 (*c* 0.1 MeOH); UV (MeOH) λ_{max} (log ε) 215 (sh), 270 (4.68) nm; ECD (MeOH) 210 ($\Delta\varepsilon$ -2.55), 285 ($\Delta\varepsilon$ -0.93) nm; IR ν_{max} 3380, 2975, 2926, 1645, 1510, 1433 cm^{-1}; ^{1}H and ^{13}C NMR spectral data seen in Table S1 and S2; HRESIMS *m/z* 583.21912 $[M-H]^-$ (calcd for $C_{31}H_{35}O_{11}$ 583.21849).

Silexcrin E (**5**), pale-yellow solid, $[\alpha]^{20}_{D}$ -15.6 (*c* 0.1 MeOH); UV (MeOH) λ_{max} (log ε) 215 (sh), 270 (4.66) nm; ECD (MeOH) 220 ($\Delta\varepsilon$ -1.45) nm; IR ν_{max} 3380, 2975, 2926, 1645, 1510, 1433 cm^{-1}; ^{1}H and ^{13}C NMR spectral data seen in Table S1 and S2; HRESIMS *m/z* 583.21832 $[M-H]^-$ (calcd for $C_{31}H_{35}O_{11}$ 583.21849).

Silexcrin F (**6**), off-white solid, $[\alpha]^{20}_{D}$ -80.4 (*c* 0.1 MeOH); UV (MeOH) λ_{max} (log ε) 265 (4.67) nm; ECD (MeOH) 220 ($\Delta\varepsilon$ -11.14), 268 ($\Delta\varepsilon$ -7.52) nm; IR ν_{max} 3420, 2964, 2916, 2873, 1641, 1610, 1583, 1450 cm^{-1}; ^{1}H and ^{13}C NMR spectral data seen in Table S3 and S4; HRESIMS *m/z* 729.27808 $[M-H]^-$ (calcd for $C_{37}H_{45}O_{15}$ 729.27639).

Silexcrin G (**7**), pale-yellow solid, $[\alpha]^{20}_{D}$ −66.7 (*c* 0.1 MeOH); UV (MeOH) λ_{max} (log ε) 265 (4.52) nm; ECD (MeOH) 220 ($\Delta\varepsilon$ -6.11), 265 ($\Delta\varepsilon$ -4.78) nm; IR ν_{max} 3390, 2970, 2912, 1642, 1612, 1584, 1444 cm^{-1}; ^{1}H and ^{13}C NMR spectral data seen in Table S3 and S4; HRESIMS *m/z* 567.22455 $[M-H]^-$ (calcd for $C_{31}H_{35}O_{10}$ 567.22357).

Silexcrin H (**8**), pale-yellow solid, $[\alpha]^{20}_{D}$ -30.8 (*c* 0.1 MeOH); UV (MeOH) λ_{max} (log ε) 270 (4.55) nm; ECD (MeOH) 220 ($\Delta\varepsilon$ +1.76), 240 ($\Delta\varepsilon$ -0.88) nm; IR ν_{max} 3394, 2925, 1647, 1578, 1510, 1435 cm^{-1}; ^{1}H and ^{13}C NMR spectral data seen in Table S3 and S4; HRESIMS *m/z* 567.22458 $[M-H]^-$ (calcd for $C_{31}H_{35}O_{10}$ 567.22357).

Silexcrin I (**9**), pale-yellow solid, $[\alpha]^{20}_{D}$ 6.5 (*c* 0.1 MeOH); UV (MeOH) λ_{max} (log ε) 215 (sh),

265 (4.79) nm; ECD (MeOH) 210 (Δε -4.08), 270 (Δε +8.25) nm; IR ν_{max} 3395, 2918, 1648, 1610, 1581, 1513, 1449 cm^{-1}; ^{1}H and ^{13}C NMR spectral data seen in Table S3 and S4; HRESIMS *m/z* 513.17749 [M-H]$^{-}$ (calcd for $C_{27}H_{29}O_{10}$ 513.17662).

Silexcrin J (**10**), off-white solid, $[\alpha]^{20}_{D}$ -13.2 (*c* 0.1 MeOH); UV (MeOH) λ_{max} (log *ε*) 215 (sh), 265 (4.60) nm; ECD (MeOH) 270 (Δε +6.38) nm; IR ν_{max} 3367, 2913, 1650, 1620, 1580, 1497, 1448 cm^{-1}; ^{1}H and ^{13}C NMR spectral data seen in Table S3 and S4; HRESIMS *m/z* 729.27814 [M-H]$^{-}$ (calcd for $C_{37}H_{45}O_{15}$ 729.27639).

Silexcrin K (**11**), pale-yellow solid, $[\alpha]^{20}_{D}$ -3.2 (*c* 0.1 MeOH); UV (MeOH) λ_{max} (log *ε*) 265 (4.69) nm; ECD (MeOH) 220 (Δε -2.61), 270 (Δε +6.91) nm; IR ν_{max} 3371, 2912, 2858, 1647, 1580, 1504, 1447 cm^{-1}; ^{1}H and ^{13}C NMR spectral data seen in Table S3 and S4; HRESIMS *m/z* 567.22400 [M-H]$^{-}$ (calcd for $C_{31}H_{35}O_{10}$ 567.22357).

Silexcrin L (**12**), off-white solid, $[\alpha]^{20}_{D}$ -9.5 (*c* 0.1 MeOH); UV (MeOH) λ_{max} (log *ε*) 265 (3.93) nm; ECD (MeOH) 220 (Δ*ε* +1.24), 260 (Δ*ε* -0.31) nm; IR ν_{max} 3376, 2921, 2858, 16480, 1580, 1504, 1447 cm^{-1}; ^{1}H and ^{13}C NMR spectral data seen in Table S7 and S8; HRESIMS *m/z* 729.27777 [M-H]$^{-}$ (calcd for $C_{37}H_{45}O_{15}$ 729.27639).

Silexcrin M (**13**), pale-yellow solid, $[\alpha]^{20}_{D}$ -42.2 (*c* 0.1 MeOH); UV (MeOH) λ_{max} (log *ε*) 218 (sh), 270 (4.66) nm; ECD (MeOH) 210 (Δε +4.1), 220 (Δε -1.56), 270 (Δε -4.16) nm; IR ν_{max} 3367, 2967, 2920, 1661, 1630, 1574, 1515, 1477, 1431 cm^{-1}; ^{1}H and ^{13}C NMR spectral data seen in Table S5 and S6; HRESIMS *m/z* 599.21411 [M-H]$^{-}$ (calcd for $C_{31}H_{35}O_{12}$ 599.21340).

Silexcrin N (**14**), pale-yellow solid, $[\alpha]^{20}_{D}$ 13.4 (*c* 0.1 MeOH); UV (MeOH) λ_{max} (log *ε*) 218 (sh), 270 (4.63) nm; ECD (MeOH) 210 (Δε +5.55), 220 (Δε -1.55), 270 (Δε +3.6) nm; IR ν_{max} 3356, 2973, 2920, 1661, 1630, 1574, 1515, 1477, 1431 cm^{-1}; ^{1}H and ^{13}C NMR spectral data seen in Table S5 and S6; HRESIMS *m/z* 599.21501 [M-H]$^{-}$ (calcd for $C_{31}H_{35}O_{12}$ 599.21340).

Silexcrin O (**15**), pale-yellow solid, $[\alpha]^{20}_{D}$ 2.0 (*c* 0.1 MeOH); UV (MeOH) λ_{max} (log *ε*) 218 (sh), 270 (4.52) nm; ECD (MeOH) 210 (Δε +2.82), 220 (Δε -1.64), 250 (Δε +0.73), 270 (Δε +0.64), 300 (Δε +0.73) nm; IR ν_{max} 3306, 2982, 2925, 1658, 1515, 1444 cm^{-1}; ^{1}H and ^{13}C NMR spectral data seen in Table S5 and S6; HRESIMS *m/z* 599.21484 [M-H]$^{-}$ (calcd for $C_{31}H_{35}O_{12}$ 599.21340).

Silexcrin P (**16**), pale-yellow solid, $[\alpha]^{20}_{D}$ -39.3 (*c* 0.1 MeOH); UV (MeOH) λ_{max} (log *ε*) 218 (sh), 270 (4.63) nm; ECD (MeOH) 218 (Δε +1.82), 230 (Δε -1.92), 245 (Δε +0.61), 275 (Δε -1.72) nm; IR ν_{max} 3373, 2923, 1651, 1575, 1514, 1433 cm^{-1}; ^{1}H and ^{13}C NMR spectral data seen in Table S5 and S6; HRESIMS *m/z* 599.21405 [M-H]$^{-}$ (calcd for $C_{31}H_{35}O_{12}$ 599.21340).

Silexcrin Q (**17**), pale-yellow solid, $[\alpha]^{20}_{D}$ 3.3 (*c* 0.1 MeOH); UV (MeOH) λ_{max} (log *ε*) 268 (4.58) nm; ECD (MeOH) 221 (Δ*ε* -3.31), 265 (Δ*ε* +5.88) nm; IR ν_{max} 3368, 2919, 1650, 1619, 1579, 1521, 1447 cm^{-1}; ^{1}H and ^{13}C NMR spectral data seen in Table S7 and S8; HRESIMS *m/z* 515.15739 [M-

H]$^-$ (calcd for $C_{26}H_{27}O_{11}$ 515.15588).

Silexcrin R (**18**), pale-yellow solid, $[\alpha]^{20}_{D}$ -20.4 (*c* 0.1 MeOH); UV (MeOH) λ_{max} (log *ε*) 265 (4.46) nm; ECD (MeOH) 224 (Δ*ε* -0.56), 270 (Δ*ε* +2.78) nm; IR ν_{max} 3360, 2917, 1646, 1580, 1513, 1446 cm^{-1}; ^{1}H and ^{13}C NMR spectral data seen in Table S7 and S8; HRESIMS *m/z* 529.17206 [M-H]$^-$ (calcd for $C_{27}H_{29}O_{11}$ 529.17153).

Silexcrin S (**19**), pale-yellow solid, $[\alpha]^{20}_{D}$ -39.8 (*c* 0.1 MeOH); UV (MeOH) λ_{max} (log *ε*) 265 (4.63) nm; ECD (MeOH) 210 (Δ*ε* +6.39), 225 (Δ*ε* +3.26), 260 (Δε -1.43) nm; IR ν_{max} 3352, 2923, 1649, 1613, 1581, 1520, 1488, 1436 cm^{-1}; ^{1}H and ^{13}C NMR spectral data seen in Table S7 and S8; HRESIMS *m/z* 515.15680 [M-H]$^-$ (calcd for $C_{26}H_{27}O_{11}$ 515.15588).

Silexcrin T (**20**), pale-yellow solid, $[\alpha]^{20}_{D}$ -41.4 (*c* 0.1 MeOH); UV (MeOH) λ_{max} (log *ε*) 210 (sh), 270 (4.64) nm; ECD (MeOH) 210 (Δ*ε* +4.74), 220 (Δ*ε* -1.62), 260 (Δ*ε* -0.27) nm; IR ν_{max} 3379, 2920, 1648, 1579, 1511, 1435 cm^{-1}; ^{1}H and ^{13}C NMR spectral data seen in Table S7 and S8; HRESIMS *m/z* 543.18762 [M-H]$^-$ (calcd for $C_{28}H_{31}O_{11}$ 543.18718).

Silexcrin U (**21**), pale-yellow solid, $[\alpha]^{20}_{D}$ -52.9 (*c* 0.1 MeOH); UV (MeOH) λ_{max} (log *ε*) 210 (sh), 270 (4.68) nm; ECD (MeOH) 210 (Δ*ε* +6.37), 226 (Δ*ε* -2.17) nm; IR ν_{max} 3366, 2919, 1646, 1577, 1511, 1435 cm^{-1}; ^{1}H and ^{13}C NMR spectral data seen in Table S7 and S8; HRESIMS *m/z* 583.21899 [M-H]$^-$ (calcd for $C_{31}H_{35}O_{11}$ 583.21849).

Supplementary Table:

Supplementary Table 1 ^{1}H NMR Spectroscopic Data for Compounds **1−5**

position	1[b]	2[a]	3[a]	4[a]	5[b]
2	8.54 s	8.53 s	8.47 s	8.41 s	8.41 s
2'	7.08 d (2.0)	7.34 d (1.9)	7.02 d (1.9)	7.07 d (2.0)	7.32 d (2.0)
5'	7.19 d (8.5)	6.89 d (8.3)	6.79 d (8.1)	7.17 d (8.4)	6.88 d (8.30)
6'	6.96 dd (2.0, 8.5)	7.17 dd (1.9, 8.3)	6.83 dd (1.9, 8.1)	6.95 dd (2.0, 8.4)	7.15 dd (2.0, 8.3)
1''	3.41, 3.60 m	3.50 m	3.41, 3.60 m	3.33 m	3.34 m
2''	5.19 m	5.18 m	5.18 m	5.12 m	5.13 m
4''	1.63 s	1.63 s	1.63 s	1.63 s	1.63 s
5''	1.74 s	1.78 s	1.74 s	1.73 s	1.74 s
1'''	3.50, 3.72 m	3.70 m	3.50, 3.71 m	3.42 m	3.44 m
2'''	5.19 m	5.18 m	5.18 m	5.14 m	5.13 m
4'''	1.64 s	1.64 s	1.64 s	1.63 s	1.64 s
5'''	1.78 s	1.74 s	1.78 s	1.77 s	1.77 s
7-*O*- anomeric proton	4.68 d (7.6)	4.68 d (7.6)	4.68 d (7.8)		
Others in sugar moiety	2.8-3.8 m	2.8-3.8 m	2.8-3.8 m		
3'-*O*- anomeric proton		4.77 d (7.3)			4.76 d (7.6)
Others in sugar moiety		2.8-3.8 m			3.0-3.8 m
4'-*O*- anomeric proton	4.74 d (7.3)			4.73 d (7.3)	
Others in sugar moiety	2.8-3.8 m			3.0-3.8 m	
5-OH	13.11 s	13.12 s	13.16 s	13.18 s	13.19 s
3'-OH	8.71 s		9.00 s		
4'-OH			9.07 s		8.77 s

* [a] Recorded at 400 MHz in DMSO. [b] Recorded at 600 MHz in DMSO. Chemical shifts (δ) are expressed in ppm, and *J* values are presented in Hz.

Supplementary Table 2 ^{13}C NMR Spectroscopic Data for Compounds **1−5**

position	1[b]	2[a]	3[a]	4[a]	5[b]
2	155.3 d	155.1 d	154.8 d	154.4 d	154.1 d
3	122.1 s	122.2 s	122.5 s	125.6 s	121.9 s
4	181.1 s	181.1 s	181.2 s	180.4 s	180.5 s
5	156.9 s	156.9 s	156.9 s	156.7 s	156.7 s
6	118.3 s	118.2 s	118.1 s	111.7 s	111.6 s
7	158.1 s	158.1 s	158.0 s	159.4 s	159.0 s
8	113.8 s	113.8 s	113.7 s	106.3 s	106.2 s
9	152.7 s	152.7 s	152.7 s	152.8 s	152.7 s
10	108.0 s	108.0 s	108.0 s	104.5 s	104.6 s
1'	125.4 s	121.7 s	121.6 s	121.5 s	121.6 s
2'	116.8 d	117.6 d	116.6 d	116.8 d	117.6 d
3'	146.5 s	145.0 s	145.6 s	146.4 s	144.9 s
4'	145.4 s	147.0 s	144.9 s	145.2 s	146.9 s
5'	116.6 d	115.7 d	115.4 d	116.6 d	115.6 d
6'	120.1 d	123.9 d	120.0 d	120.0 d	123.8 d
1''	22.5 t	22.5 t	22.5 t	21.4 t	21.3 t
2''	122.8 d	122.8 d	122.8 d	122.3 d	122.17 d
3''	130.7 s	130.7 s	130.6 s	130.7 s	130.7 s
4''	25.48 q	25.48 q	25.4 q	25.48 q	25.37 q
5''	18.03 q	18.1 q	17.99 q	17.8 q	17.7 q
1'''	22.5 t	22.5 t	22.4 t	21.4 t	21.3 t
2'''	122.7 d	122.6 d	122.7 d	122.3 d	122.23 d
3'''	131.3 s	131.3 s	131.2 s	131.1 s	131.1 s
4'''	25.52 q	25.52 q	25.5 q	25.45 q	25.40 q
5'''	18.05 q	18.0 q	18.01 q	17.8 q	17.7 q
7-*O*- anomeric C	104.8 d	104.8 d	104.8 d		
Others in sugar moiety	61.0 t, 69.87 d, 74.0 d, 76.5 d, 77.5 d	61.0 t, 69.94 d, 74.0 d, 76.5 d, 77.5 d	61.0 t, 69.9 d, 74.0 d, 76.4 d, 77.4 d		
3'-*O*- anomeric C		102.2 d			102.2 d
Others in sugar moiety		60.8 t, 69.90 d, 73.4 d, 76.1 d, 77.2 d			60.8 t, 69.9 d, 73.3 d, 76.0 d, 77.1 d
4'-*O*- anomeric C	102.3 d			102.3 d	
Others in sugar moiety	60.8 t, 69.86 d, 73.4 d, 75.9 d, 77.3 d			60.8 t, 69.8 d, 73.3 d, 75.9 d, 77.2 d	

* [a] Recorded at 400 MHz in DMSO. [b] Recorded at 600 MHz in DMSO. Chemical shifts (δ) are expressed in ppm.

Supplementary Table 3 ^{1}H NMR Spectroscopic Data for Compounds **6-11**

position	6[b]	7[a]	8[a]	9[a]	10[b]	11[a]
2	8.58 s	8.52 s	8.44 s	8.47 s	8.44 s	8.38 s
8				6.81 s	6.80 s	6.79 s
2'	7.52 d (8.7)	7.40 d (8.7)	7.51 d (8.8)	7.54 d (8.8)	7.32 d (2.0)	7.25 d (2.0)
3'	7.11 d (8.7)	6.83 d (8.7)	7.10 d (8.8)	7.03 d (8.8)		
5'	7.11 d (8.7)	6.83 d (8.7)	7.10 d (8.8)	7.03 d (8.8)	7.15 d (8.3)	6.85 d (8.3)
6'	7.52 d (8.7)	7.40 d (8.7)	7.51 d (8.8)	7.54 d (8.8)	7.34 dd (2.0, 8.3)	7.22 dd (2.0, 8.3)
1''	3.36, 3.59 m	3.37, 3.49 m	3.34 m	3.23, 3.44 m	3.22, 3.45 m	3.44 m
2''	5.18 m	5.17 m	5.12 m	5.23 m	5.22 m	5.22 m
4''	1.63 s	1.64 s	1.63 s	1.62 s	1.62 s	1.68 s
5''	1.74 s	1.74 s	1.73 s	1.75 s	1.75 s	1.75 s
1'''	3.48, 3.71 m	3.60, 3.70m	3.44 m		3.30, 3.42 m	3.26 m
2'''	5.18 m	5.17 m	5.14 m		5.34 m	5.30 m
4'''	1.64 s	1.63 s	1.64 s		1.68 s	1.62 s
5'''	1.78 s	1.78 s	1.77 s		1.70 s	1.68 s
7-*O*- anomeric proton	4.68 d (7.6)	4.67 d (7.7)			5.03 d (7.5)	5.03 d (7.3)
Others in sugar moiety	2.8-3.8 m	2.8-3.8 m			3.0-3.8 m	3.0-3.8 m
4'-*O*- anomeric proton	4.92 d (7.2)		4.92 d (6.9)	5.03 d (7.3)	4.85 d (7.5)	
Others in sugar moiety	2.8-3.8 m		3.0-3.8 m	3.0-3.8 m	3.0-3.8 m	
$O\text{-}CH_3$				3.80 s		
5-OH	13.09 s	13.13 s	13.16 s	13.15 s	13.15 s	13.19 s

* [a] Recorded at 400 MHz in DMSO. [b] Recorded at 600 MHz in DMSO. Chemical shifts (δ) are expressed in ppm, and J values are presented in Hz.

Supplementary Table 4 ^{13}C NMR Spectroscopic Data for Compounds **6-11**

position	6[b]	7[a]	8[a]	9[a]	10[b]	11[a]
2	155.3 d	154.9 d	154.3 d	154.7 d	154.9 d	154.3 d
3	122.0 s	122.4 s	124.4 s	122.1 s	122.4 s	122.7 s
4	181.1 s	181.3 s	180.4 s	180.5 s	180.5 s	180.6 s
5	156.9 s	156.9 s	156.7 s	158.0 s	158.1 s	158.1 s
6	118.3 s	118.2 s	111.7 s	112.8 s	112.8 s	112.6 s
7	158.1 s	158.1 s	159.3 s	160.8 s	160.8 s	160.7 s
8	113.8 s	113.7 s	106.3 s	93.2 d	93.2 d	93.1 d
9	152.8 s	152.8 s	152.9 s	155.3 s	155.4 s	155.4 s
10	108.0 s	108.0 s	104.5 s	105.8 s	105.9 s	105.8 s
1'	124.2 s	121.2 s	121.5 s	122.9 s	124.1 s	121.2 s
2'	130.1 d	130.7 d	130.1 d	130.1 d	129.9 d	130.1 d
3'	116.1 d	115.1 d	116.0 d	113.7 d	130.2 s	127.3 s
4'	157.3 s	157.5 s	157.2 s	159.2 s	155.2 s	155.1 s
5'	116.1 d	115.1 d	116.0 d	113.7 d	114.8 d	114.6 d
6'	130.1 d	130.7 d	130.1 d	130.1 d	127.7 d	127.5 d
1''	22.5 t	22.5 t	21.4 t	21.2 t	21.3 t	21.2 t
2''	122.7 d	122.8 d	122.3 d	121.9 d	122.0 d	122.0 d
3''	130.7 s	130.7 s	130.8 s	130.8 s	130.9 s	130.8 s
4''	25.47 q	25.6 q	25.45 q	25.5 q	25.56 q	25.52 q
5''	18.02 q	18.07 q	17.8 q	17.7 q	17.78 q	17.73 q
1'''	22.5 t	22.5 t	21.4 t		28.2 t	28.2 t
2'''	122.6 d	122.7 d	122.3 d		122.7 d	122.8 d
3'''	131.2 s	131.3 s	131.1 s		131.5 s	131.2 s
4'''	25.5 q	25.5 q	25.47 q		25.61 q	25.5 q
5'''	18.03 q	18.08 q	17.8 q		17.79 q	17.67 q
7-*O*-anomeric C	104.8 d	104.8 d		100.3 d	100.4 d	100.4 d
Others in sugar moiety	61.0 t, 69.9 d, 74.0 d, 76.6 d,77.5 d	61.0 t, 69.9 d,74.0 d, 76.5 d,77.5 d		60.7 t, 69.7 d, 73.3 d, 76.7 d,77.3 d	60.7 t, 69.7 d, 73.3 d, 76.7 d,77.1d	60.7 t, 69.7 d, 73.3 d, 76.7 d,77.3 d
4'-*O*-anomeric C	100.3 d		100.3 d		101.2 d	
Others in sugar moiety	60.7 t, 69.7 d, 73.2 d, 76.4 d,77.1 d		60.7 t, 69.7 d, 73.2 d, 76.6 d,77.1 d		60.8 t, 69.8 d, 73.5 d, 76.8 d,77.3 d	
O-CH_3				55.2 q		

* [a] Recorded at 400 MHz in DMSO. [b] Recorded at 600 MHz in DMSO. Chemical shifts (δ) are expressed in ppm.

Supplementary Table 5 ^{1}H NMR Spectroscopic Data for Compounds **13-16**

position	13[a]	14[b]	15[b]	16[b]
2	8.43 s	8.43 s	8.36 s	8.36 s
2'	7.34 d (1.9)	7.34 d (1.9)	7.31 d (1.9)	7.30 d (1.8)
5'	6.88 d (8.3)	6.88 d (8.3)	6.88 d (8.3)	6.88 d (8.3)
6'	7.17 dd (1.9, 8.3)	7.17 dd (1.9, 8.3)	7.15 dd (1.9, 8.3)	7.15 dd (1.8, 8.3)
1''	3.12 d (8.4)	3.12 d (8.4)	3.18 d (7.3)	3.24 d (7.3)
2''	4.75 t (8.4)	4.75 t (8.4)	5.21 t (7.3)	5.21 t (7.3)
4''	1.17 s	1.17 s	1.63	1.63 s
5''	1.11 s	1.11 s	1.72	1.72 s
1'''	3.31 d (7.3)	3.31 d (7.3)	3.24 d (8.2)	3.25 d (8.2)
2'''	5.20 t (7.3)	5.20 t (7.3)	4.78 t (8.2)	4.78 t (8.2)
4'''	1.64 s	1.64 s	1.18 s	1.18 s
5'''	1.75 s	1.75 s	1.12 s	1.12 s
3'-*O*- anomeric proton	4.76 d (7.1)	4.76 d (7.1)	4.76 d (7.0)	4.76 d (7.0)
Others in sugar moiety	3.0-3.8 m	3.0-3.8 m	3.0-3.8 m	3.0-3.8 m
4'-OH	8.78 s	8.78 s	8.78 s	8.78 s

* [a] Recorded at 400 MHz in DMSO. [b] Recorded at 600 MHz in DMSO. Chemical shifts (δ) are expressed in ppm, and J values are presented in Hz.

Supplementary Table 6 ^{13}C NMR Spectroscopic Data for Compounds **13-16**

position	13[a]	14[b]	15[b]	16[b]
2	154.1 d	154.1 d	153.8 d	153.8 d
3	121.80 s	121.81 s	122.0 s	122.0 s
4	180.5 s	180.5 s	180.3 s	180.3 s
5	154.2 s	154.2 s	159.0 s	159.0 s
6	108.7 s	108.7 s	106.3 s	106.4 s
7	164.2 s	164.2 s	164.6 s	164.6 s
8	101.2 s	101.2 s	103.0 s	103.1 s
9	154.4 s	154.4 s	150.3 s	150.3 s
10	105.5 s	105.5 s	104.7 s	104.7 s
1'	121.77 s	121.78 s	121.9 s	121.9 s
2'	117.5 d	117.5 d	117.5 d	117.5 d
3'	145.0 s	145.0 s	145.0 s	145.0 s
4'	146.9 s	146.9 s	146.9 s	146.9 s
5'	115.6 d	115.6 d	115.6 d	115.6 d
6'	123.8 d	123.8 d	123.9 d	123.9 d
1''	26.3 t	26.3 t	21.5 t	21.5 t
2''	91.0 d	91.0 d	121.4 d	121.4 d
3''	70.2 s	70.2 s	131.2 s	131.3 s
4''	26.1 q	26.1 q	25.5 q	25.5 q
5''	24.2 q	24.2 q	17.6 q	17.6 q
1'''	21.5 t	21.5 t	26.4 t	26.3 t
2'''	121.4 d	121.4 d	91.0 d	91.0 d
3'''	131.6 s	131.6 s	70.2 s	70.2 s
4'''	25.4 q	25.4 q	26.1 q	26.1 q
5'''	17.6 q	17.6 q	24.1 q	24.1 q
3'-*O*- anomeric C	102.2 d	102.2 d	102.2 d	102.1 d
Others in sugar moiety	60.8 t, 69.9 d, 73.4 d, 76.0 d, 77.2 d	60.8 d, 69.9 d, 73.4 d, 76.0 d, 77.2 d	60.8 t, 69.9 d, 73.3 d, 76.0 d, 77.2 d	60.8 t, 69.9d, 73.4 d, 76.0 d, 77.2 d

* [a] Recorded at 400 MHz in DMSO. [b] Recorded at 600 MHz in DMSO. Chemical shifts (δ) are expressed in ppm.

Supplementary Table 7 ^{1}H NMR Spectroscopic Data for Compounds **12**, **17-21**.

position	12[b]	17[a]	18[b]	19[a]	20[a]	21[a]
2	8.49 s	8.39 s	8.43 s	8.44 s	8.47 s	8.47 s
6	6.63 s			6.62 s		
8		6.79 s	6.79 s			
2'	7.33 d (1.8)	7.02 d (1.9)	7.05 d (1.9)	7.02 d (1.9)	7.51 d (8.6)	7.51 d (8.7)
3'					7.10 d (8.6)	7.10 d (8.7)
5'	7.15 d (8.3)	6.80 d (8.1)	6.99 d (8.3)	6.79 d (8.2)	7.10 d (8.6)	7.10 d (8.7)
6'	7.36 dd (1.8, 8.3)	6.82 d (1.9, 8.1)	6.97 d (1.9)	6.84 dd (1.9, 8.2)	7.51 d (8.6)	7.51 d (8.7)
1''	3.35, 3.58 m	3.23, 3.44 m	3.22, 3.45 m	3.34, 3.54 m	2.86 t (6.5)	2.88, 2.97 m
2''	5.12 t (7.4)	5.22 m	5.22 m	5.20 m	3.62 t (6.5)	4.27 m
4''	1.63 s	1.62 s	1.62 s	1.63 s		4.75 s, 4.87 s
5''	1.78 s	1.75 s	1.75 s	1.78 s		1.76 s
1'''	3.34, 3.45 m				3.42 d (6.8)	3.33, 3.40 m
2'''	5.34 t (7.4)				5.14 t (6.8)	5.15 m
4'''	1.68 s				1.63 s	1.63 s
5'''	1.70 s				1.77 s	1.76 s
7-*O*-anomeric proton	5.02 d (7.0)	5.03 d (7.2)	5.03 d (7.4)	5.01 d (7.3)		
Others in sugar moiety	3.0-3.8 m	3.0-3.8 m	3.0-3.8 m	3.0-3.8 m		
4'-*O*-anomeric proton	4.86 d (7.2)				4.92 d (7.2)	4.92 d (7.4)
Others in sugar moiety	3.0-3.8 m				3.0-3.8 m	3.0-3.8 m
O-CH_3			3.80 s			
5-OH	12.89 s	13.22 s	13.19 s	12.97 s	13.28 s	13.35 s

* [a] Recorded at 400 MHz in DMSO. [b] Recorded at 600 MHz in DMSO. Chemical shifts (δ) are expressed in ppm, and J values are presented in Hz.

Supplementary Table 8 ^{13}C NMR Spectroscopic Data for Compounds **12**,**17-21**

position	12[b]	17[a]	18[b]	19[a]	20[a]	21[a]
2	154.9 d	154.4 d	154.7 d	154.6 d	154.5 d	154.5 d
3	122.0 s	122.6 s	122.3 s	122.3 s	121.5 s	121.5 s
4	180.5 s	180.6 s	180.5 s	180.9 s	180.5 s	180.5 s
5	159.7 s	158.1 s	158.1 s	159.8 s	157.1 s	157.2 s
6	98.2 d	112.7 s	112.7 s	98.2 d	109.7 s	109.2 s
7	160.5 s	160.8 s	160.8 s	160.4 s	160.3 s	160.7 s
8	108.2 s	93.2 d	93.2 d	108.1 s	106.4 s	106.4 s
9	154.0 s	155.3 s	155.3 s	154.0 s	153.1 s	153.2 s
10	105.8 s	105.8 s	105.8 s	105.9 s	104.5 s	104.4 s
1'	124.0 s	121.6 s	123.3 s	121.5 s	124.4 s	124.4 s
2'	130.2 d	116.6 d	116.4 d	116.6 d	130.1 d	130.1 d
3'	129.8 s	144.9 s	146.1 s	144.9 s	116.0 d	116.0 d
4'	155.2 s	145.6 s	147.8 s	145.6 s	157.2 s	157.3 s
5'	114.7 d	115.4 d	112.0 d	115.4 d	116.0 d	116.0 d
6'	127.6 d	120.0 d	119.9 d	120.0 d	130.1 d	130.1 d
1''	21.2 t	21.3 t	21.2 t	21.2 t	25.8 t	28.9 t
2''	121.9	122.0 d	122.0 d	122.0 d	60.7 t	74.6 d
3''	131.1 s	130.8 s	130.9 s	131.2 s		146.9 s
4''	25.4 q	25.5 q	25.5 q	25.5 q		110.1 t
5''	17.67 q	17.8 q	17.9 q	17.8 q		18.1 q
1'''	28.1 t				21.4 t	21.4 t
2'''	122.7 d				122.3 d	122.4 d
3'''	131.3 s				131.1 s	131.1 s
4'''	25.5 q				25.5 q	25.5 q
5'''	17.65 q				17.8 q	17.7 q
7-*O*-anomeric C	100.5 d	100.4 d	100.4 d	100.5 d		
Others in sugar moiety	60.6 t, 69.7 d, 73.3 d, 76.6 d, 77.0 d	60.7 t, 69.7 d, 73.3 d, 76.7 d, 77.3 d	60.7 t, 69.7 d, 73.3 d, 76.7 d, 77.3 d	60.5 t, 69.7 d, 73.4 d, 76.6 d, 77.2 d		
4'-*O*-anomeric C	101.2 d				100.4 d	100.4 d
Others in sugar moiety	60.8 t, 69.8 d, 73.4 d, 76.8 d, 77.1 d				60.7 t, 69.7 d, 73.2 d, 76.6 d, 77.1 d	60.7 t, 69.7 d, 73.2 d, 76.6 d, 77.1 d
O-CH_3			55.7 q			

* [a] Recorded at 400 MHz in DMSO. [b] Recorded at 600 MHz in DMSO. Chemical shifts (δ) are expressed in ppm.

Supplementary Figure:

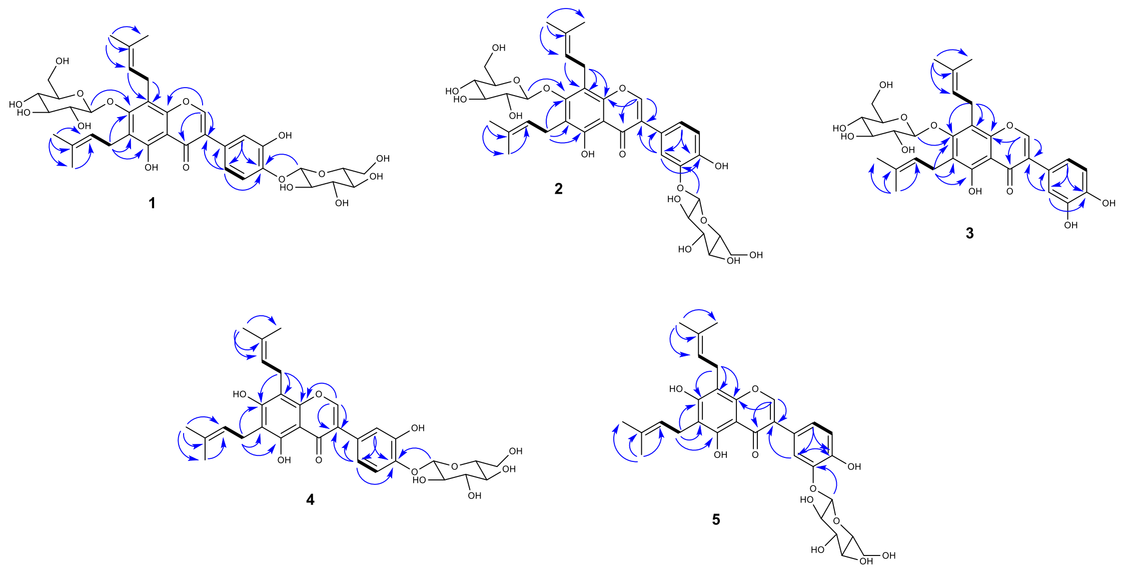

Supplementary Figure 1 Selected ^{1}H-^{1}H COSY(H—H) and HMBC (H→C) correlations of compounds **1-5**

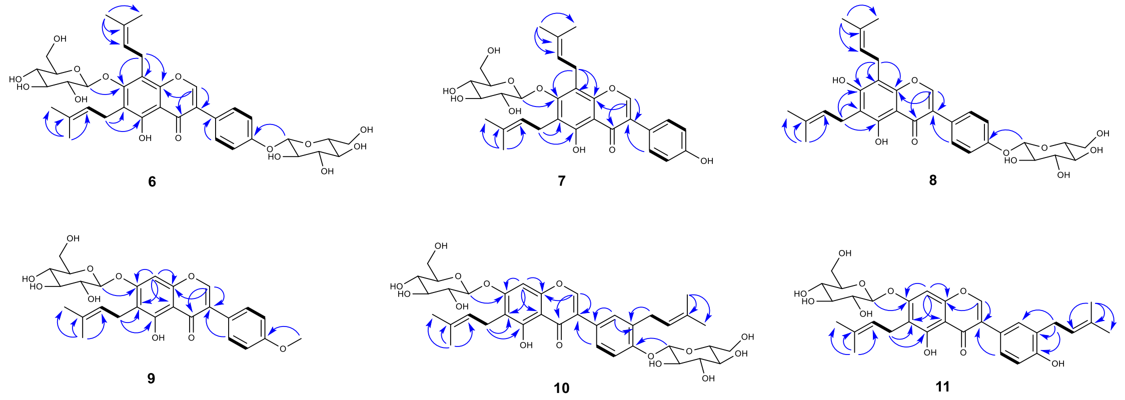

Supplementary Figure 2 Selected ^{1}H-^{1}H COSY(H—H) and HMBC (H→C) correlations of compounds **6-11**

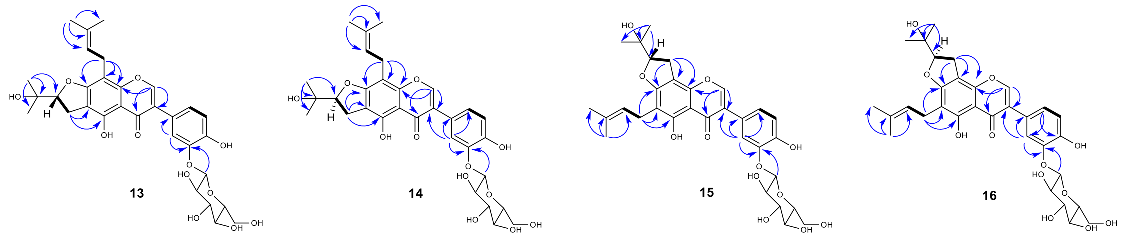

Supplementary Figure 3 Selected ^{1}H-^{1}H COSY(H—H) and HMBC (H→C) correlations of compounds **13-16**

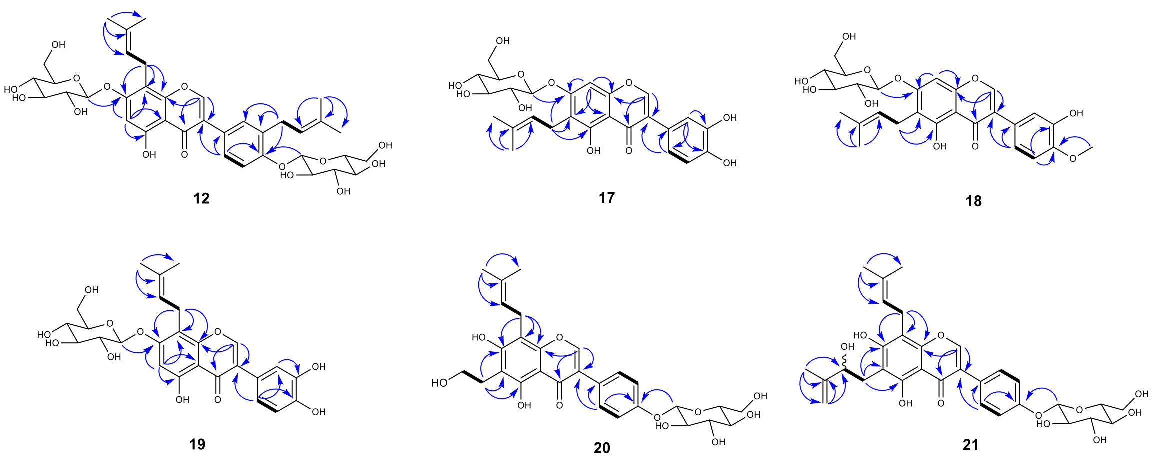

Supplementary Figure 4 Selected ^{1}H-^{1}H COSY(H—H) and HMBC (H→C) correlations of compounds **12, 17-21**

1D and 2D NMR Spectra; HRESIMS Data, IR (KBr disc), UV Spectra, and ECD Spectrum of Compounds 1-21.

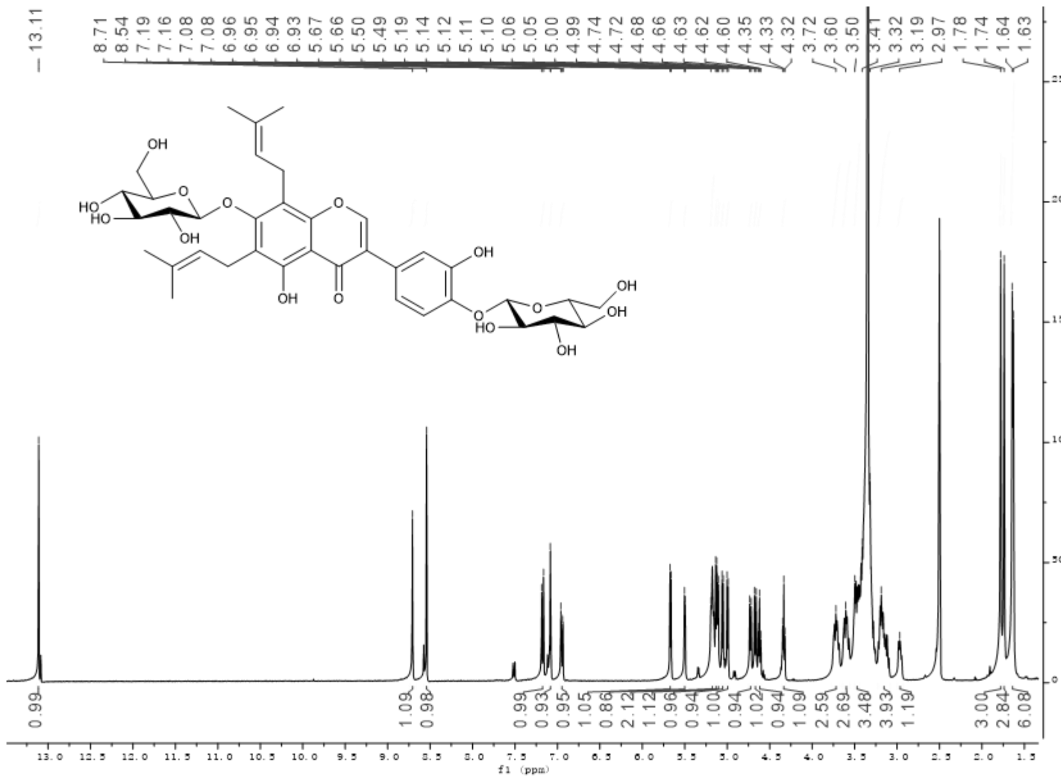

Supplementary Figure 5 ^{1}H NMR (600 MHz) spectrum of **1** in DMSO.

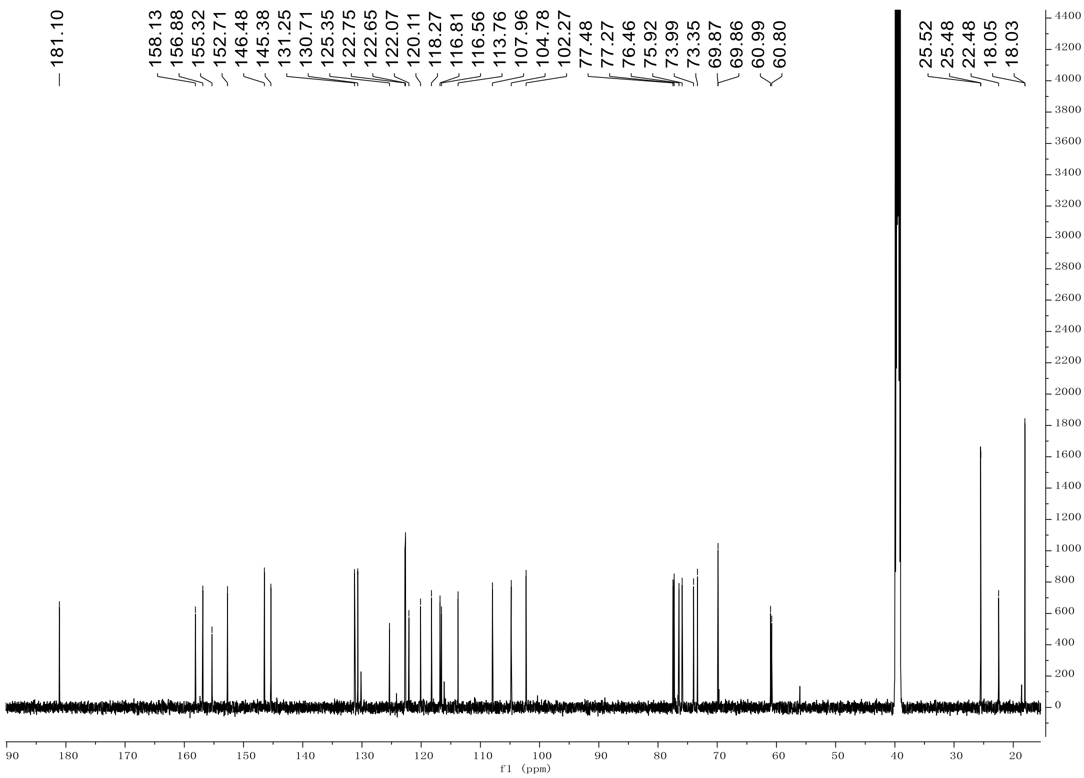

Supplementary Figure 6 ^{13}C NMR (150 MHz) spectrum of **1** in DMSO.

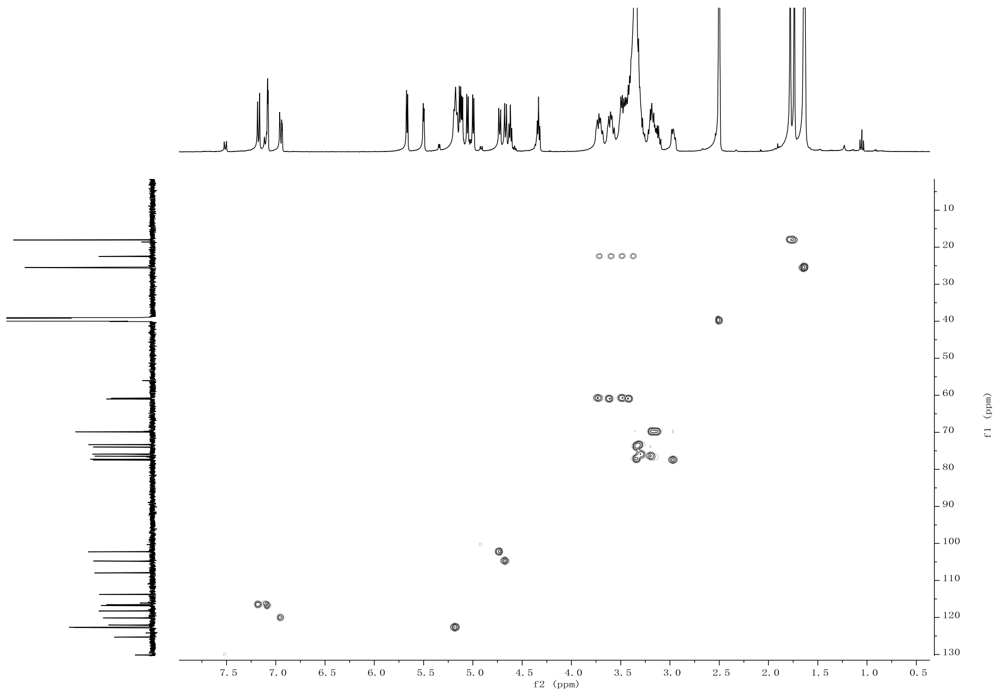

Supplementary Figure 7 HMQC (600 MHz) spectrum of **1** in DMSO.

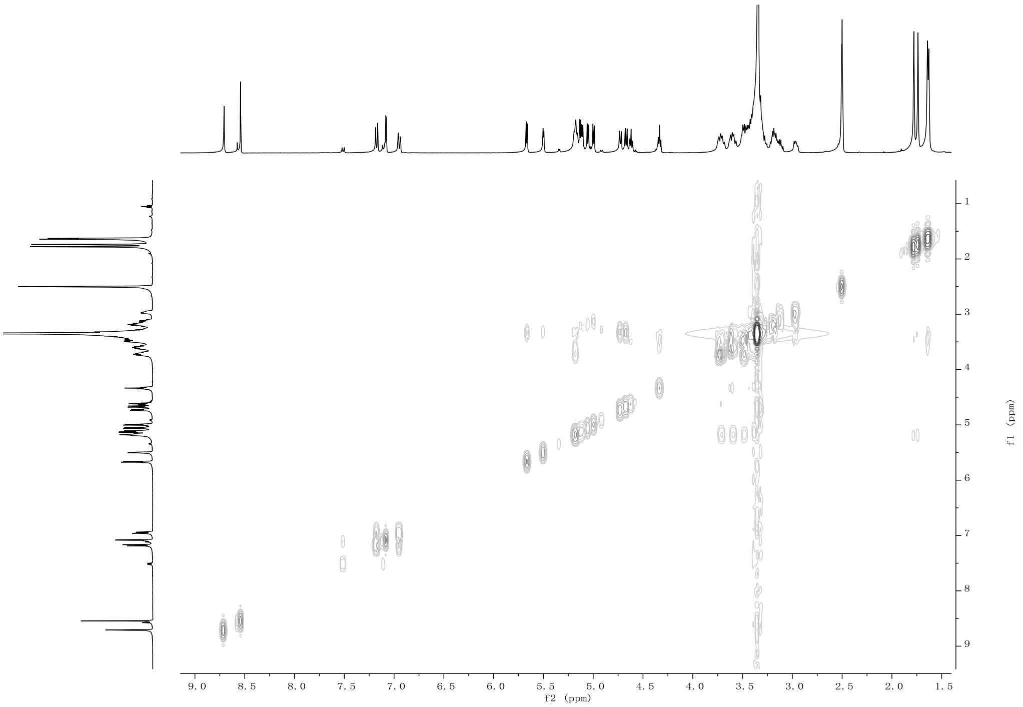

Supplementary Figure 8 ^{1}H-^{1}H COSY (600 MHz) spectrum of **1** in DMSO.

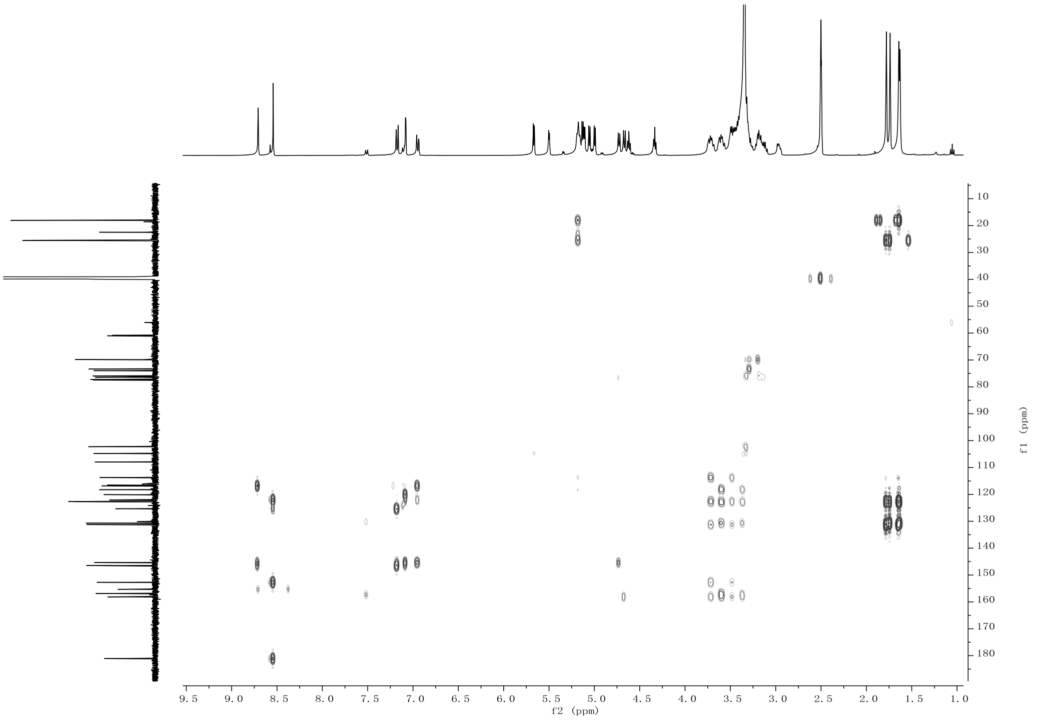

Supplementary Figure 9 HMBC (600 MHz) spectrum of **1** in DMSO.

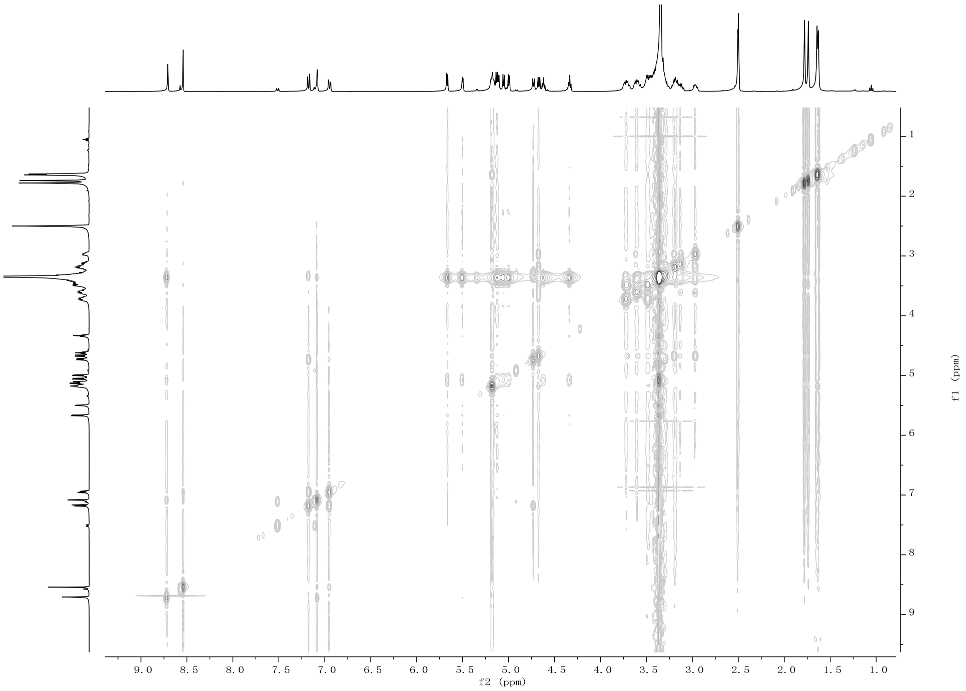

Supplementary Figure 10 ^{1}H-^{1}H NOESY (600 MHz) spectrum of **1** in DMSO.

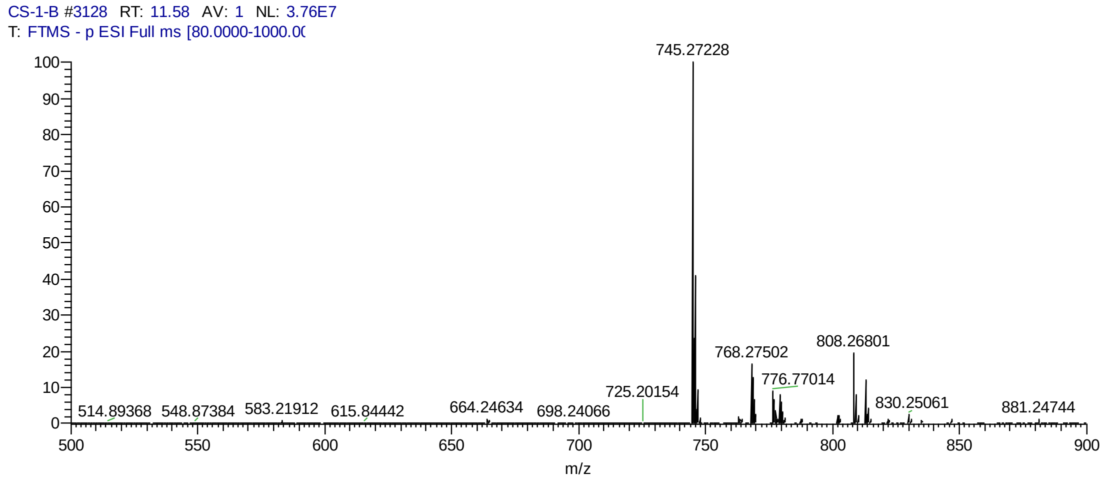

Supplementary Figure 11 HRESIMS spectrum of **1**.

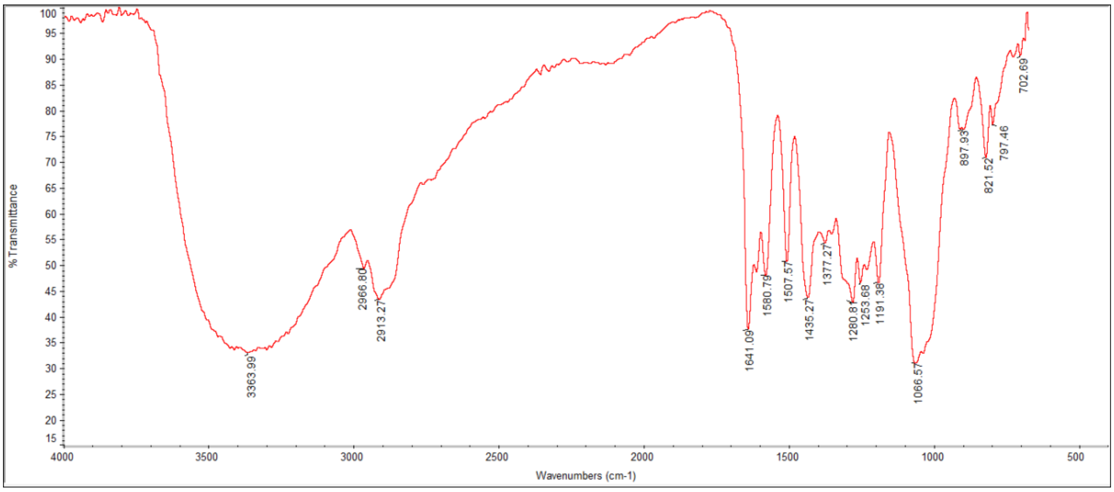

Supplementary Figure 12 IR (KBr disc) spectrum of **1**.

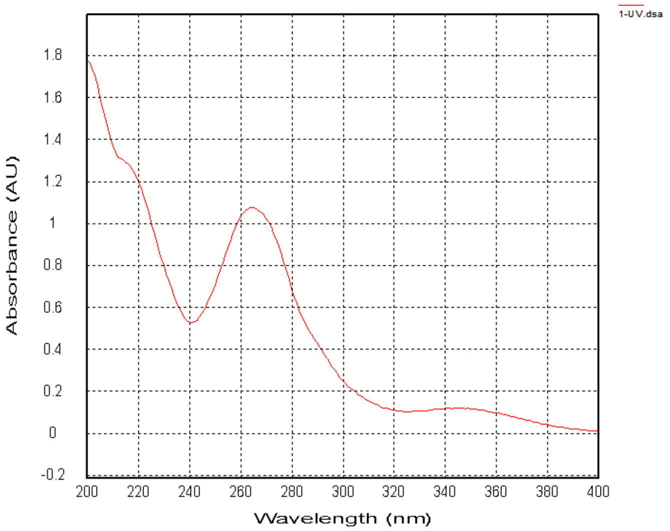

Supplementary Figure 13 UV spectrum of **1**.

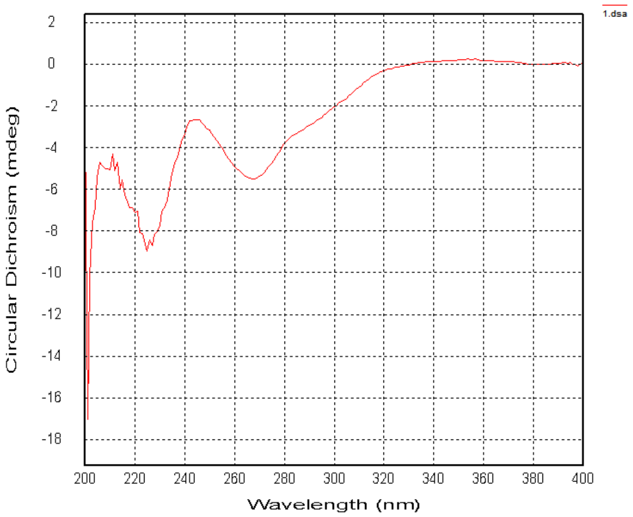

Supplementary Figure 14 ECD spectrum of **1**.

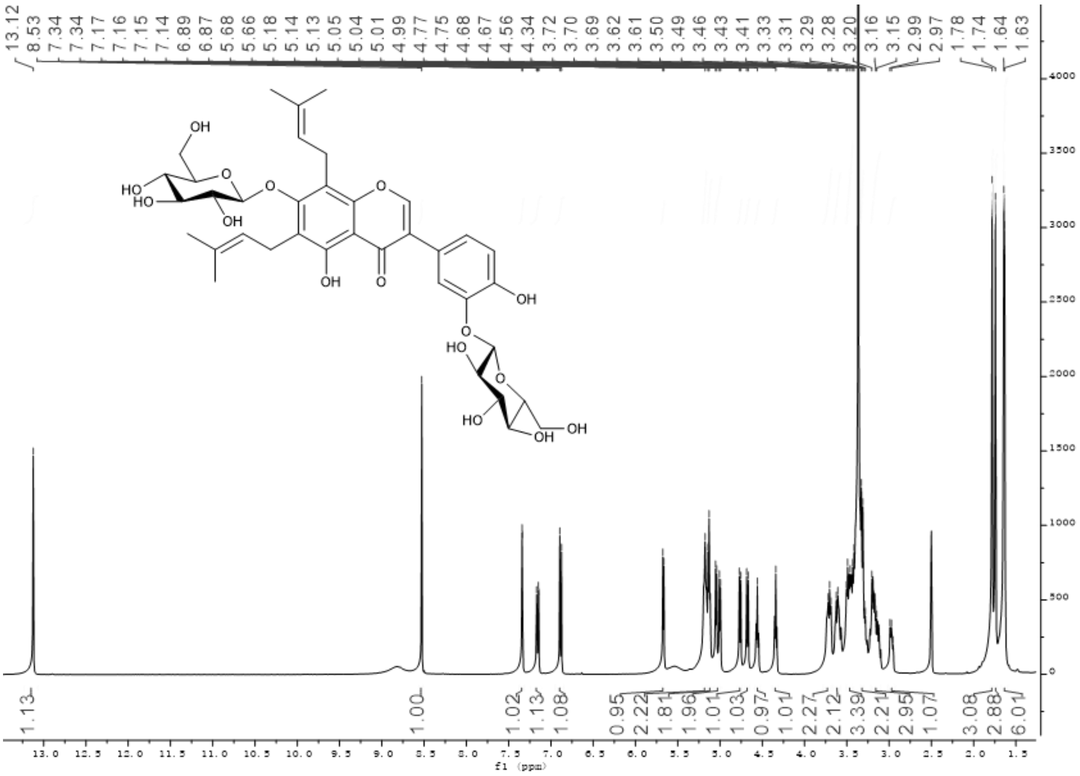

Supplementary Figure 15 ^{1}H NMR (400 MHz) spectrum of **2** in DMSO.

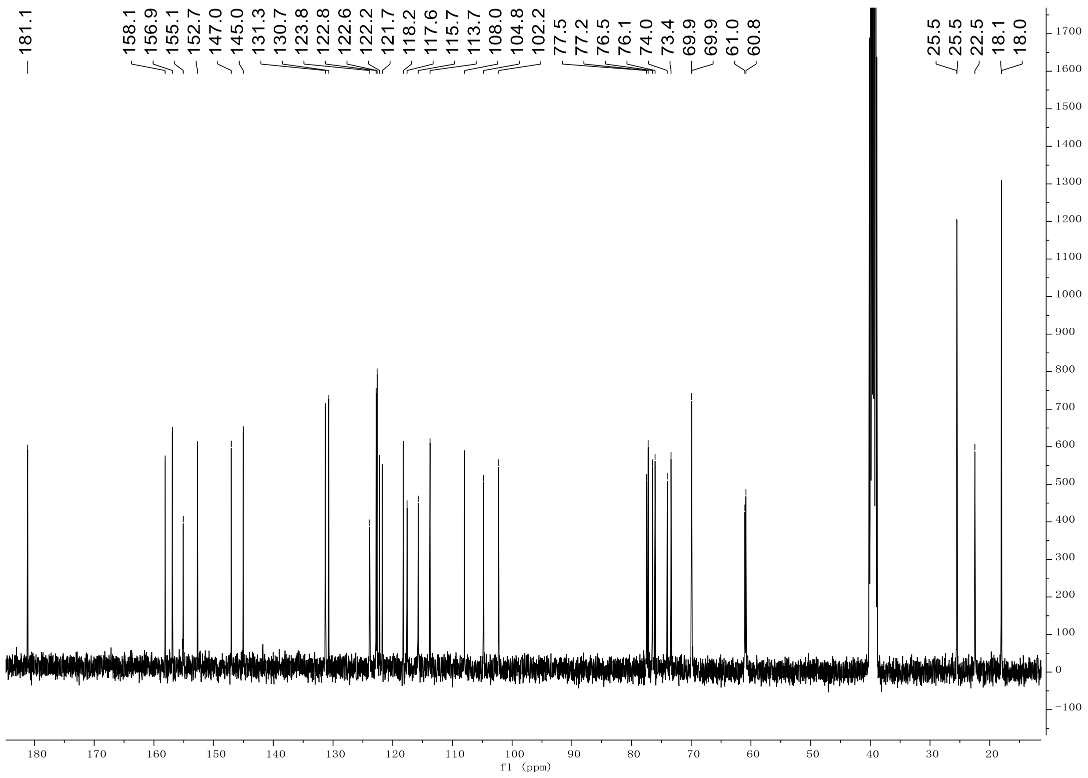

Supplementary Figure 16 ^{13}C NMR (100 MHz) spectrum of **2** in DMSO.

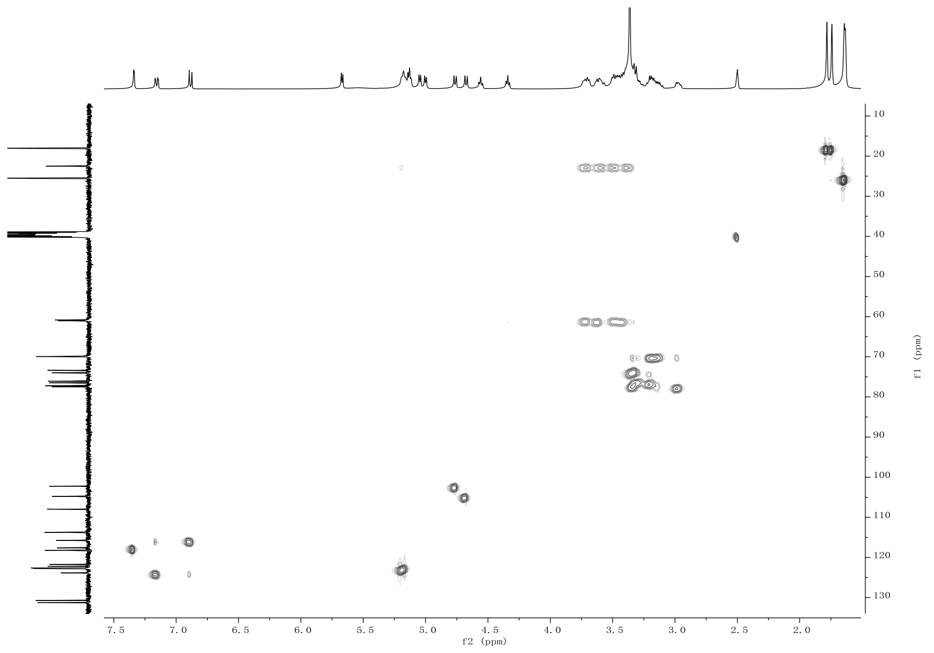

Supplementary Figure 17 HSQC (400 MHz) spectrum of **2** in DMSO.

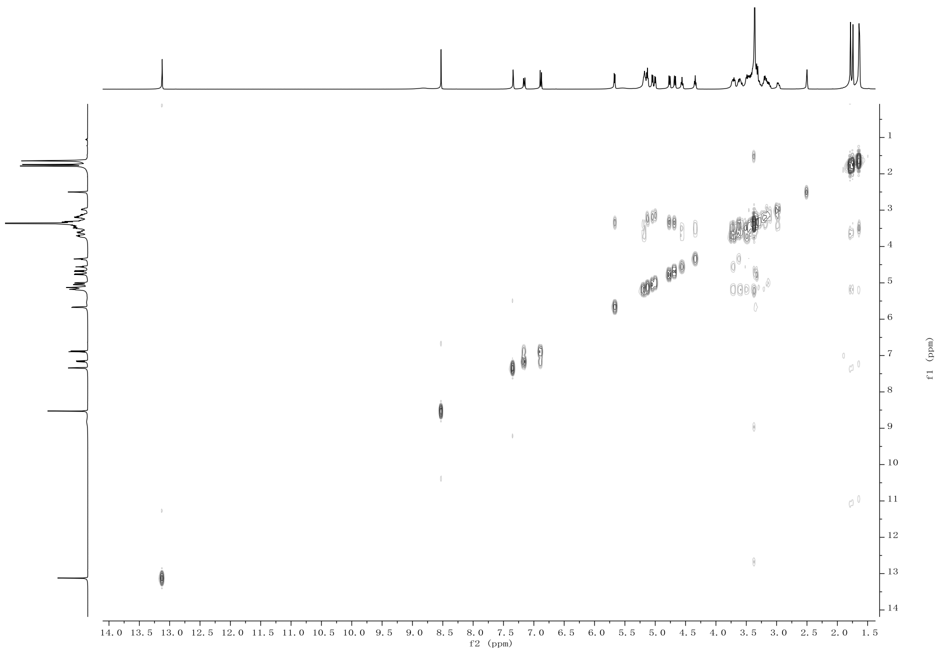

Supplementary Figure 18 ^{1}H-^{1}H COSY (600 MHz) spectrum of **2** in DMSO.

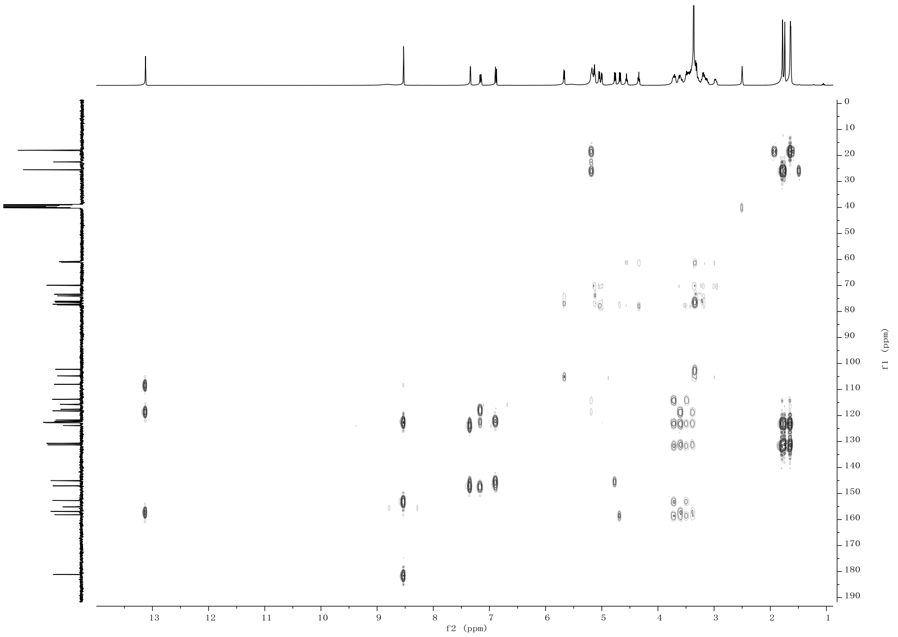

Supplementary Figure 19 HMBC (400 MHz) spectrum of **2** in DMSO.

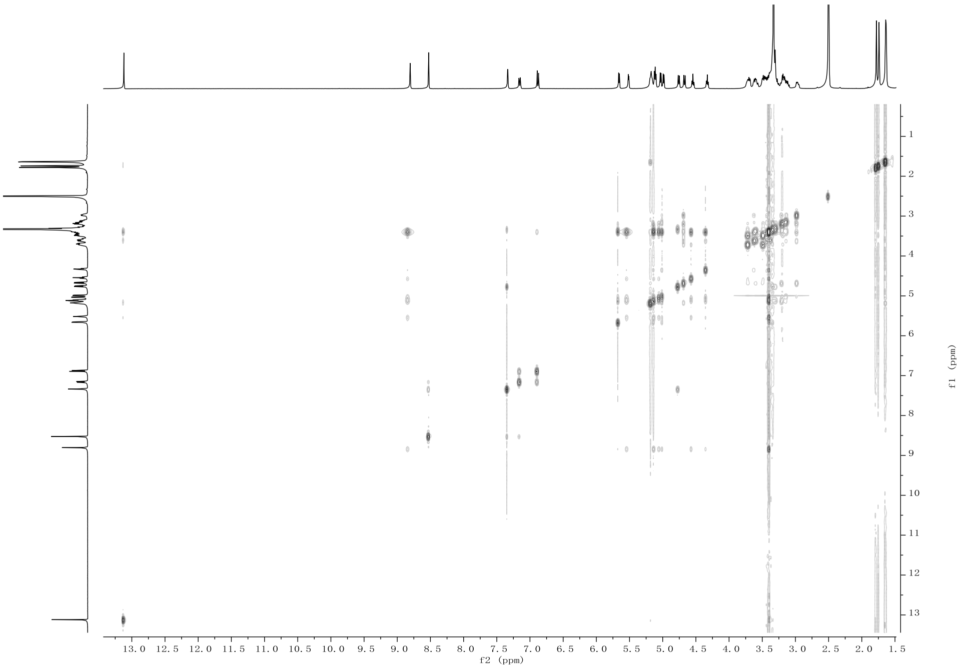

Supplementary Figure 20 ^{1}H-^{1}H NOESY (600 MHz) spectrum of **2** in DMSO.

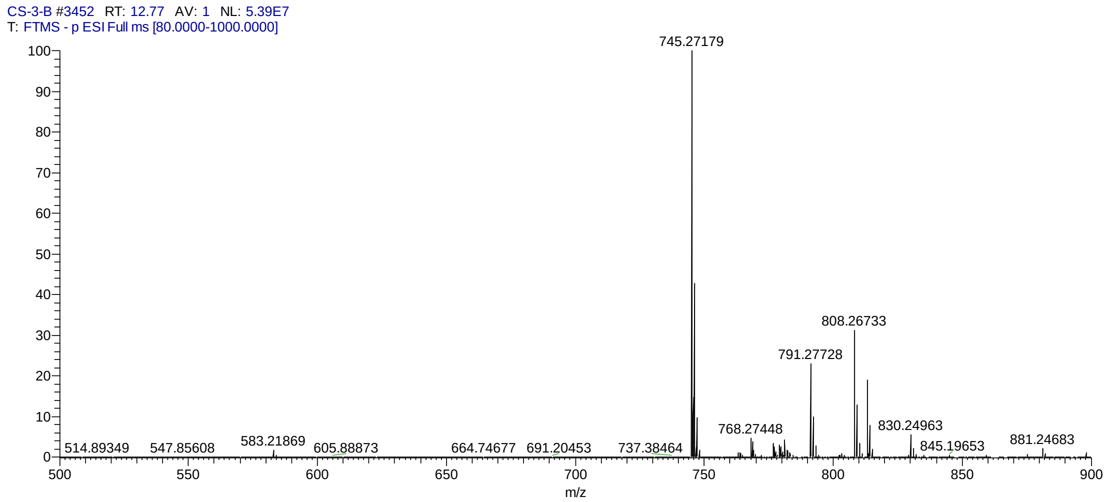

Supplementary Figure 21 HRESIMS spectrum of **2**.

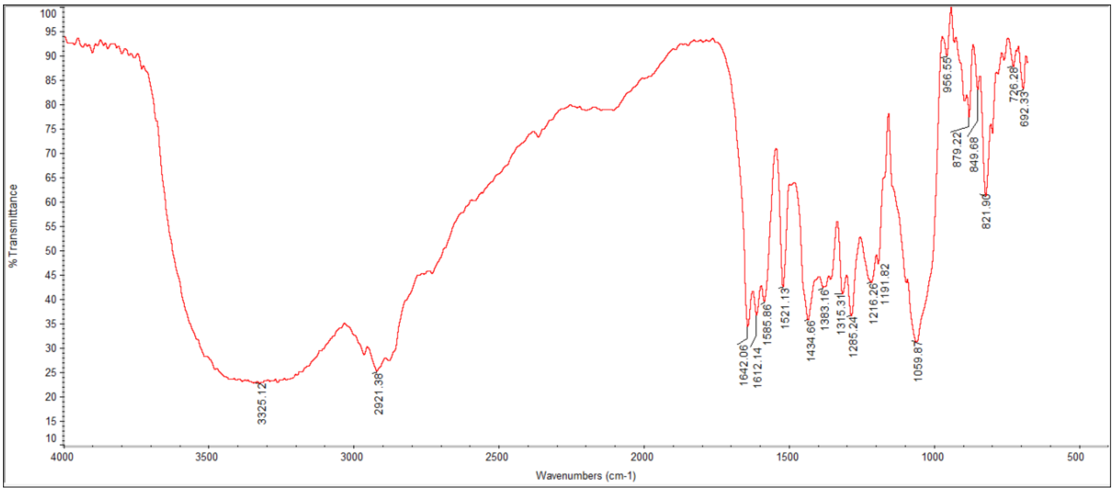

Supplementary Figure 22 IR (KBr disc) spectrum of **2**.

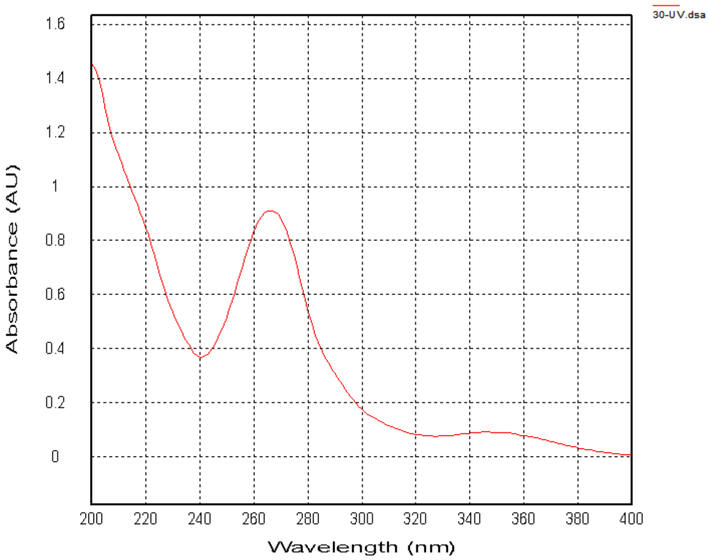

Supplementary Figure 23 UV spectrum of **2**.

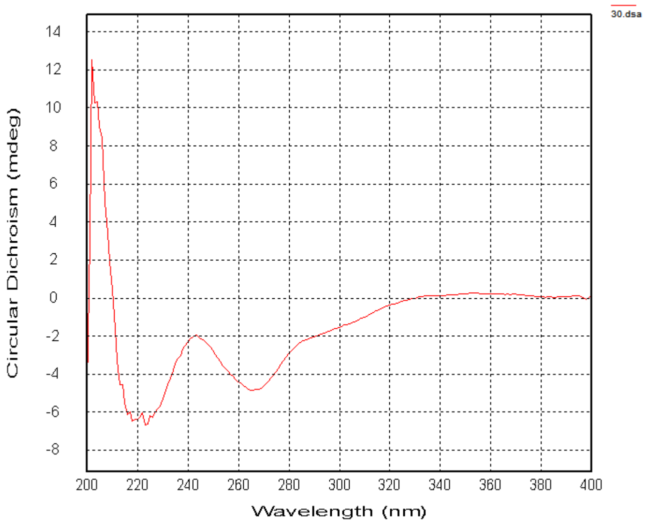

Supplementary Figure 24 ECD spectrum of **2**.

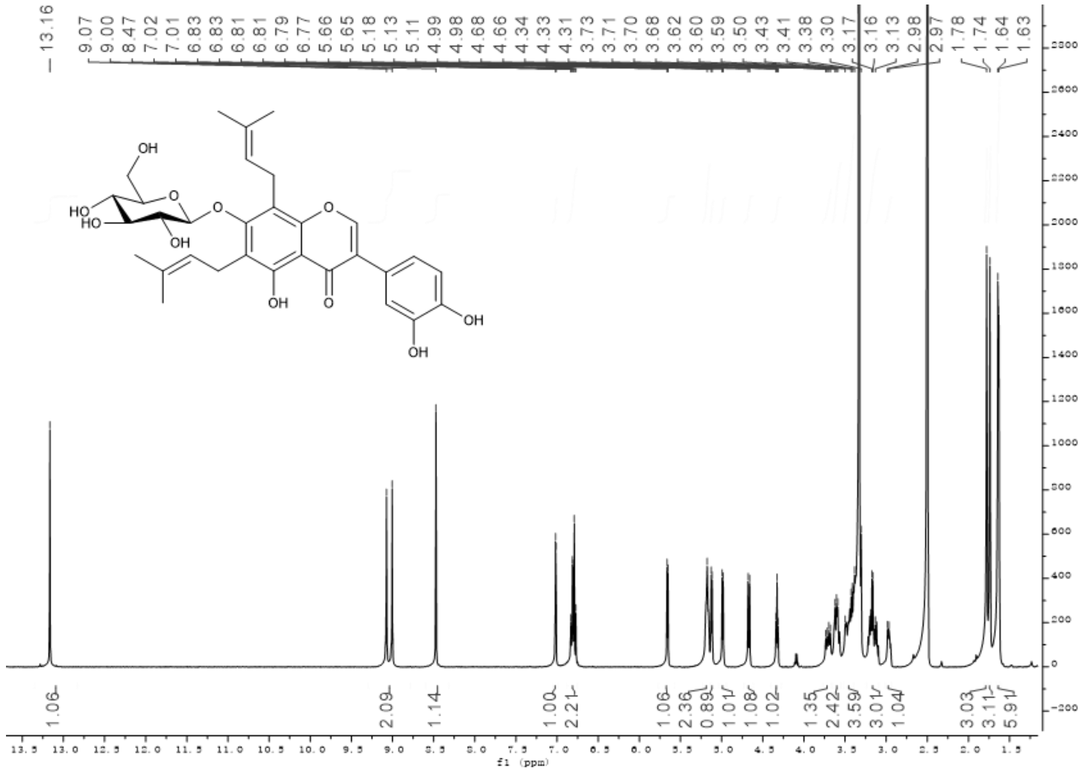

Supplementary Figure 25 ^{1}H NMR (400 MHz) spectrum of **3** in DMSO.

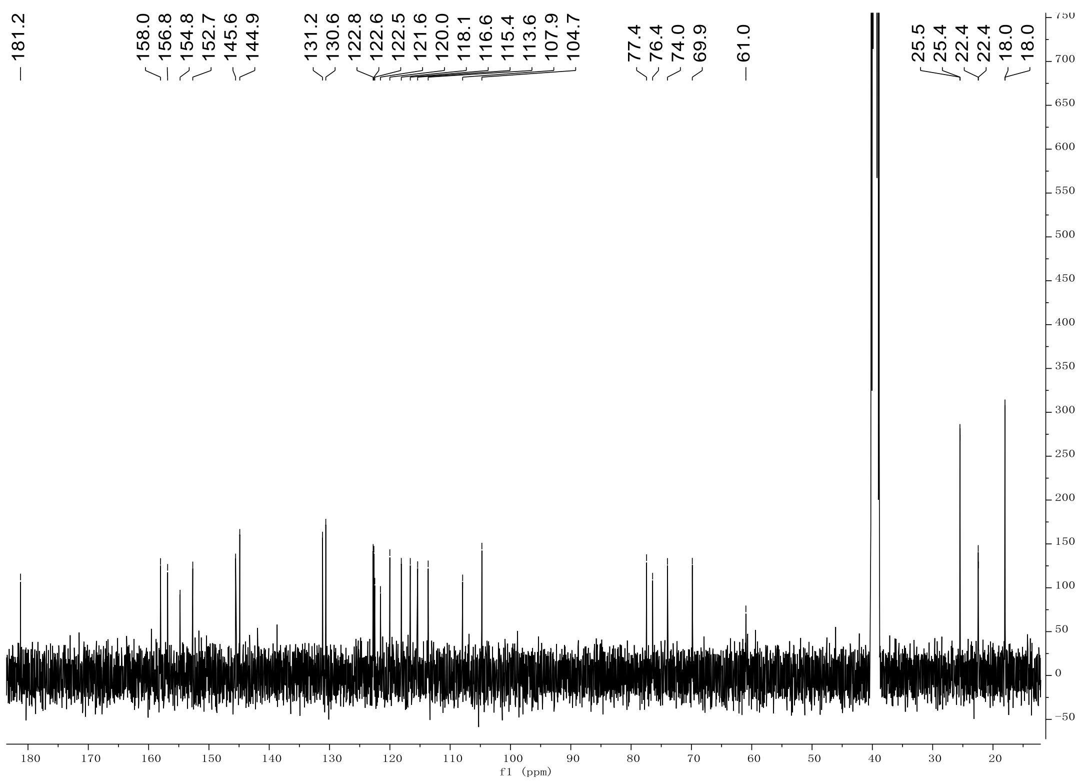

Supplementary Figure 26 ^{13}C NMR (100 MHz) spectrum of **3** in DMSO.

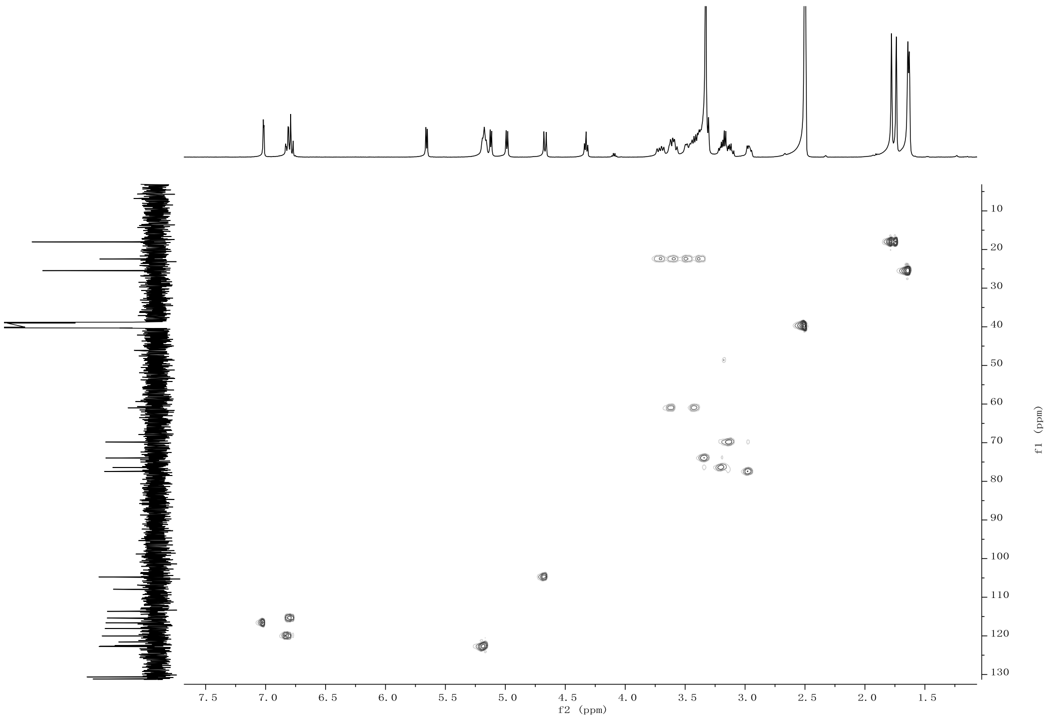

Supplementary Figure 27 HSQC (400 MHz) spectrum of **3** in DMSO.

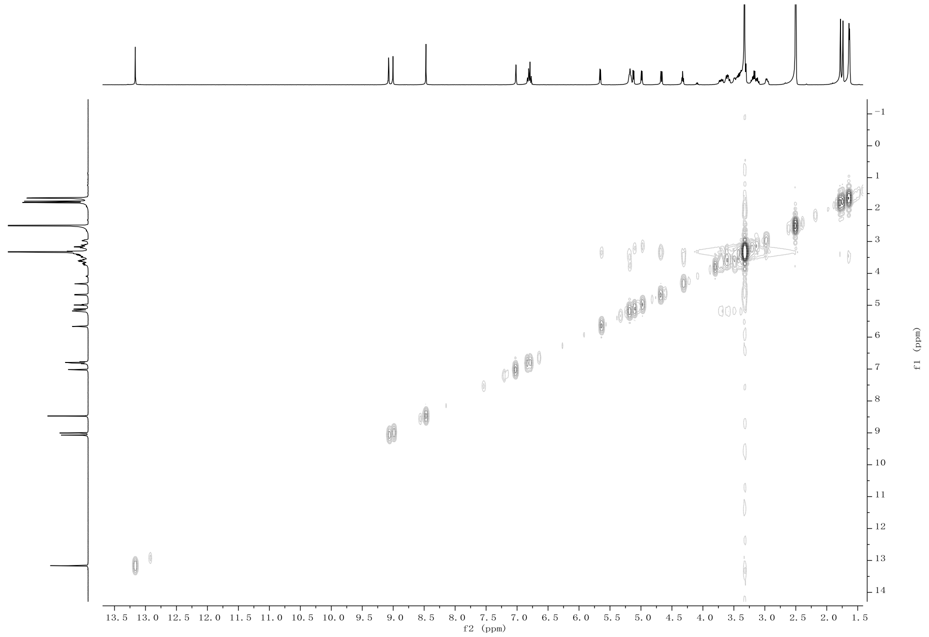

Supplementary Figure 28 ^{1}H-^{1}H COSY (600 MHz) spectrum of **3** in DMSO.

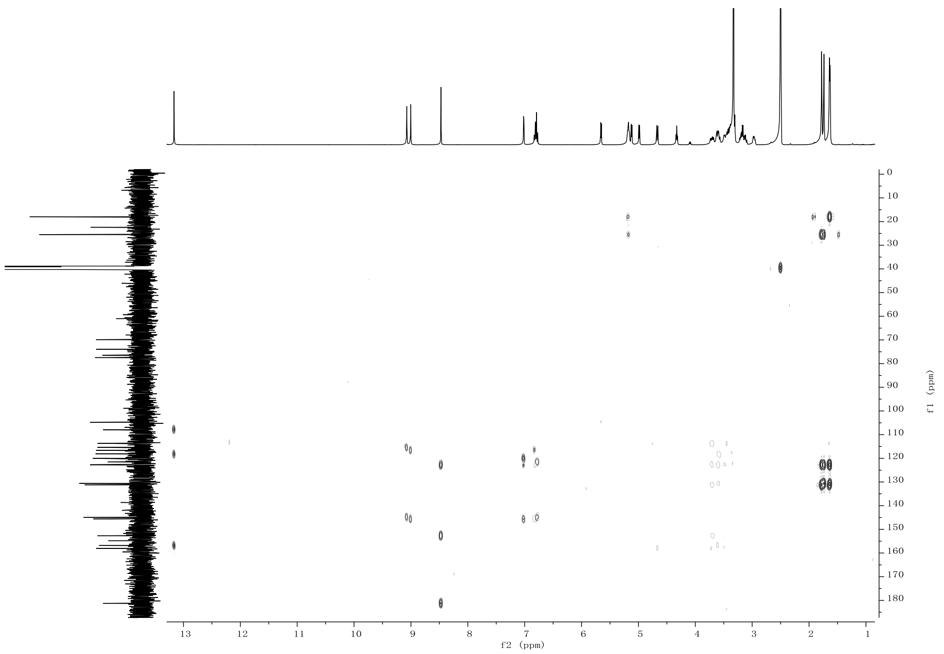

Supplementary Figure 29 HMBC (400 MHz) spectrum of **3** in DMSO.

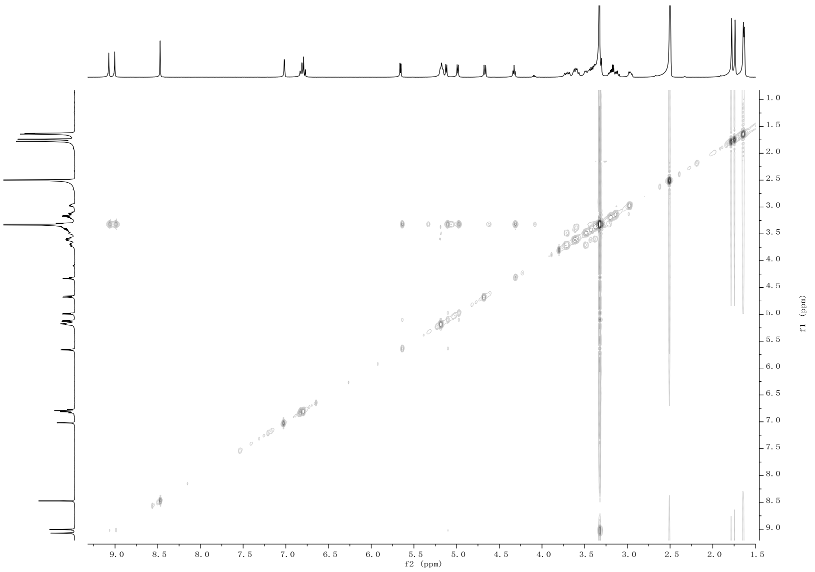

Supplementary Figure 30 ^{1}H-^{1}H NOESY (600 MHz) spectrum of **3** in DMSO.

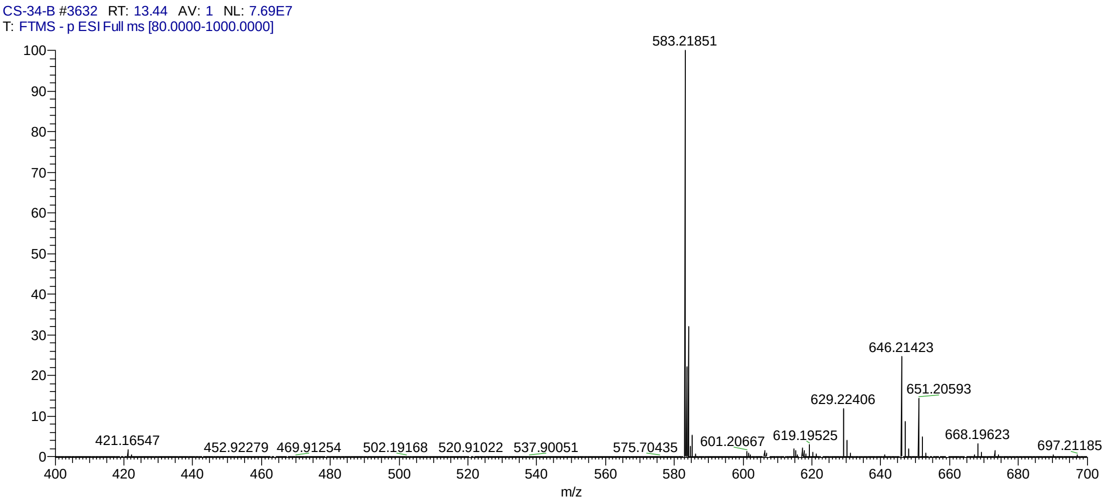

Supplementary Figure 31 HRESIMS spectrum of **3**.

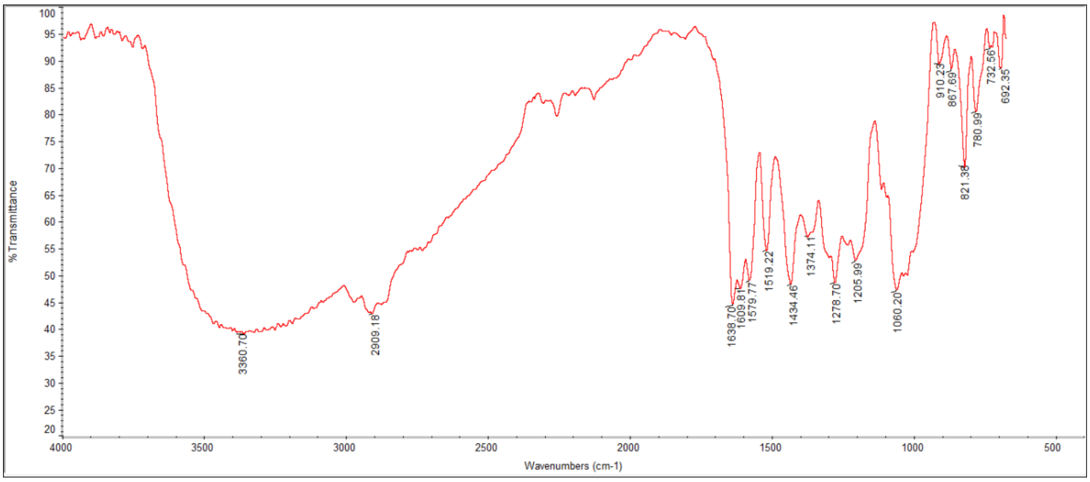

Supplementary Figure 32 IR (KBr disc) spectrum of **3**.

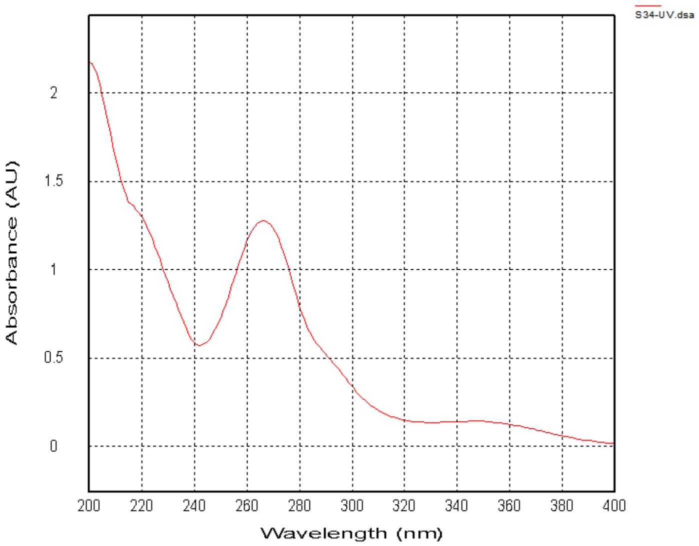

Supplementary Figure 33 UV spectrum of **3**.

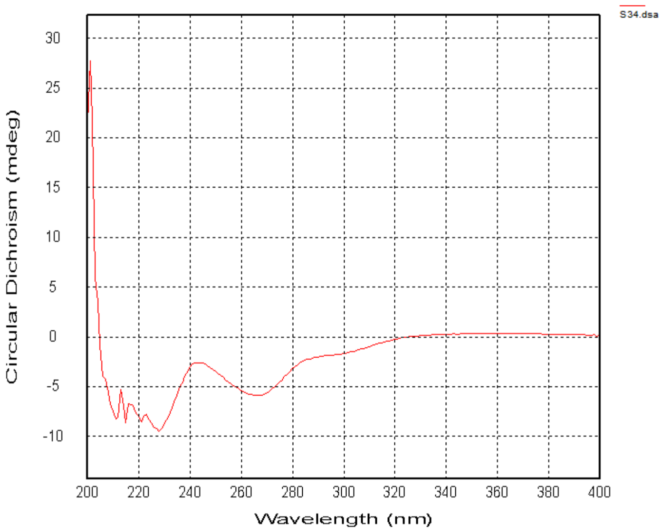

Supplementary Figure 34 ECD spectrum of **3**.

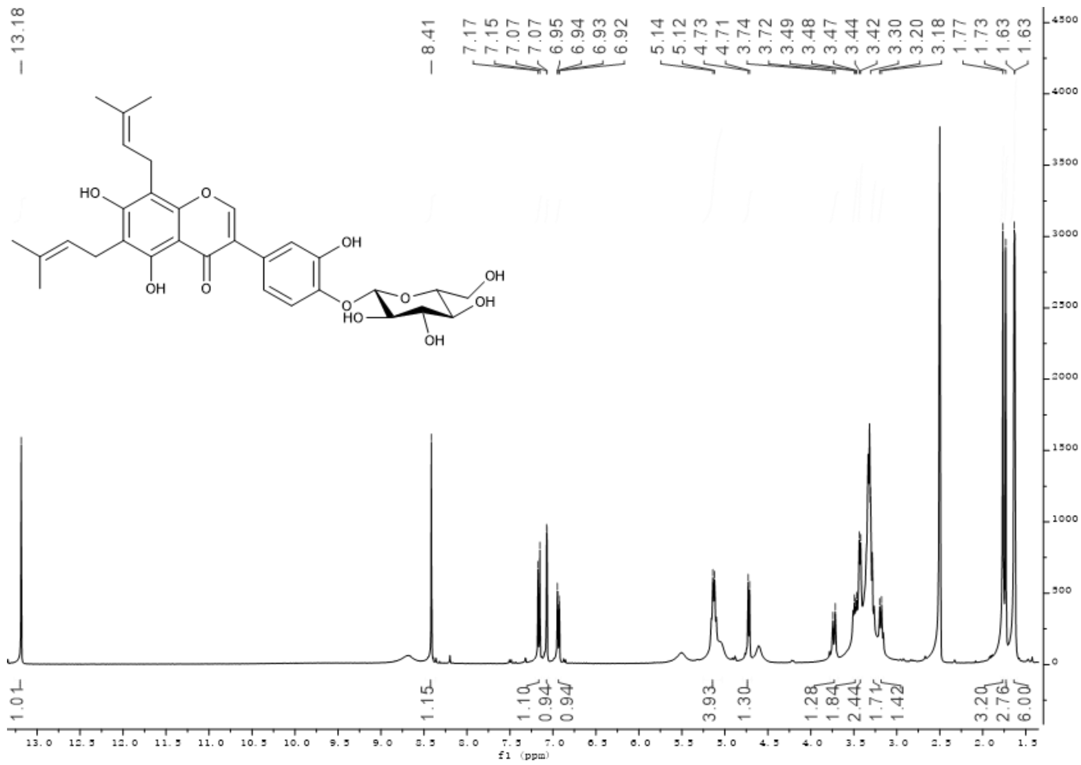

Supplementary Figure 35 ^{1}H NMR (400 MHz) spectrum of **4** in DMSO.

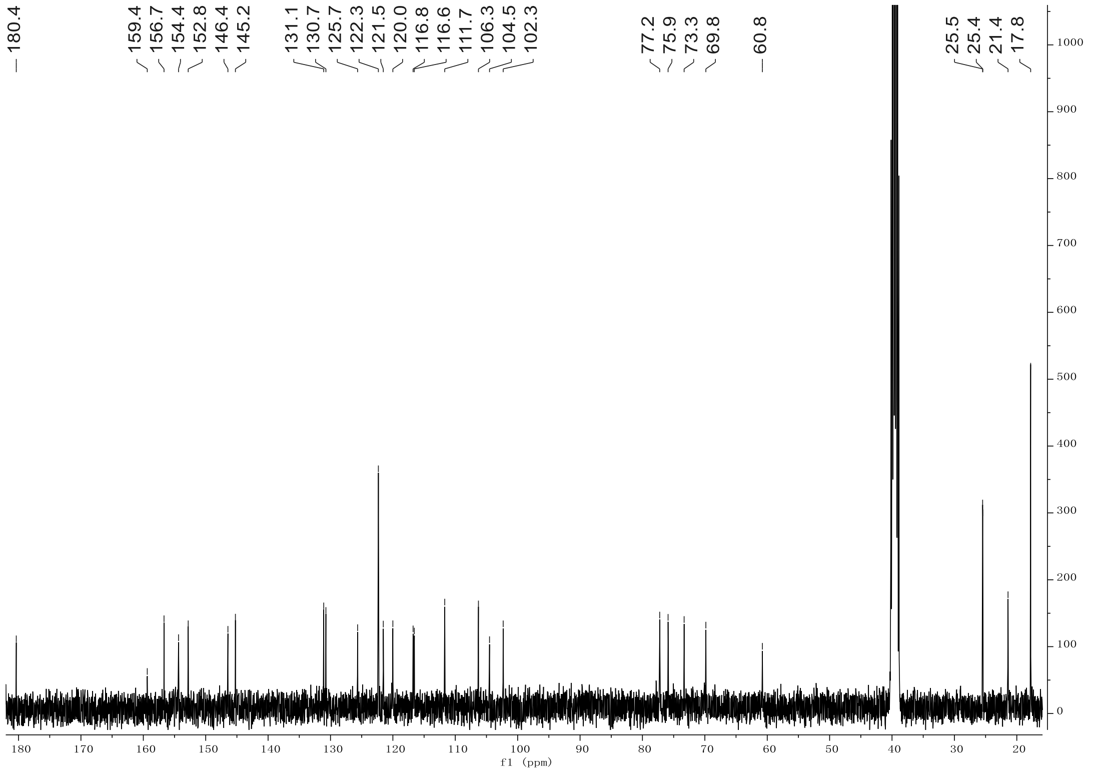

Supplementary Figure 36 ^{13}C NMR (100 MHz) spectrum of **4** in DMSO.

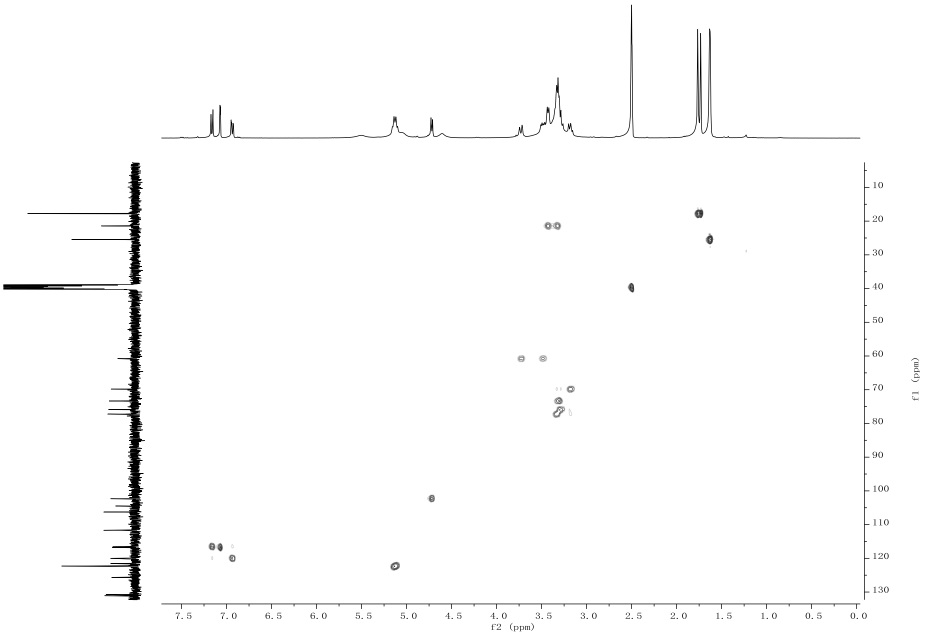

Supplementary Figure 37 HSQC (400 MHz) spectrum of **4** in DMSO.

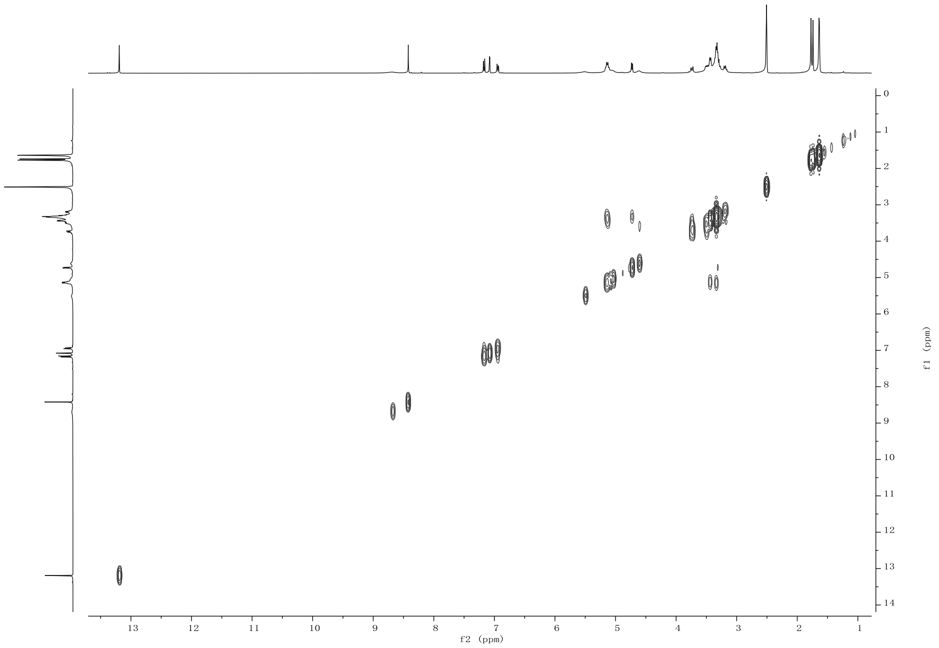

Supplementary Figure 38 ^{1}H-^{1}H COSY (600 MHz) spectrum of **4** in DMSO.

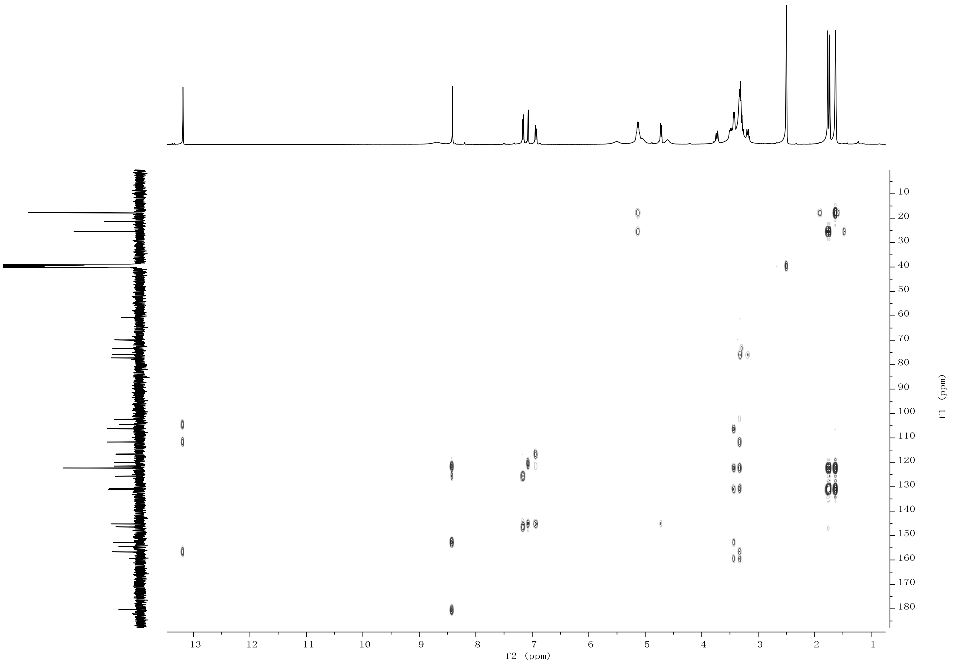

Supplementary Figure 39 HMBC (400 MHz) spectrum of **4** in DMSO.

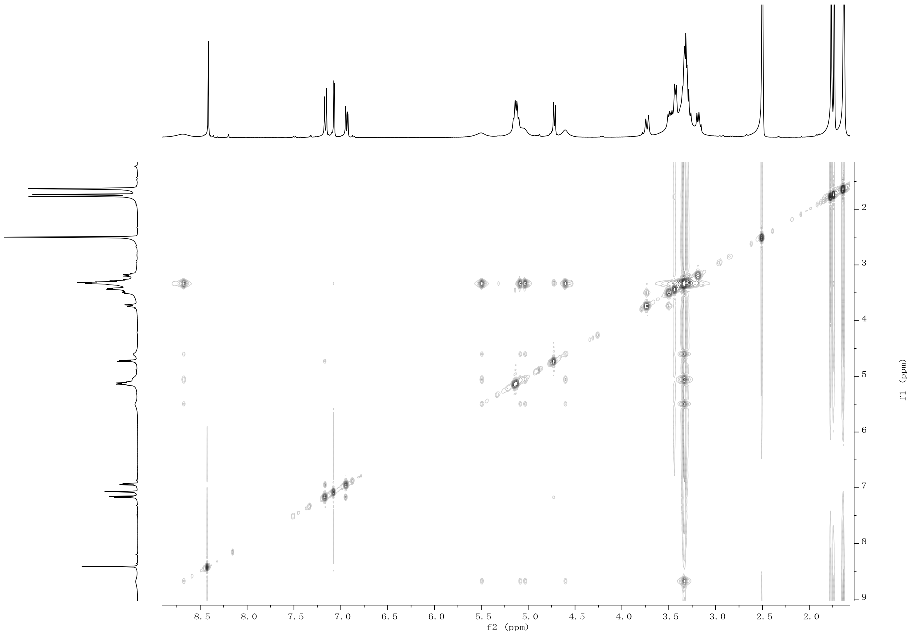

Supplementary Figure 40 ^{1}H-^{1}H NOESY (600 MHz) spectrum of **4** in DMSO.

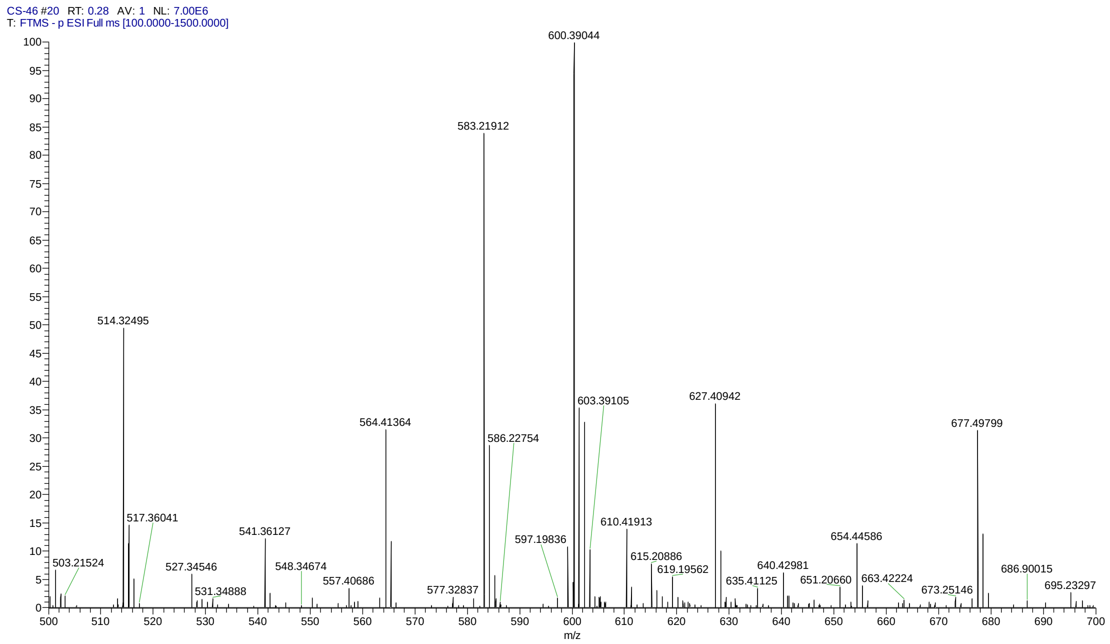

Supplementary Figure 41 HRESIMS spectrum of **4**.

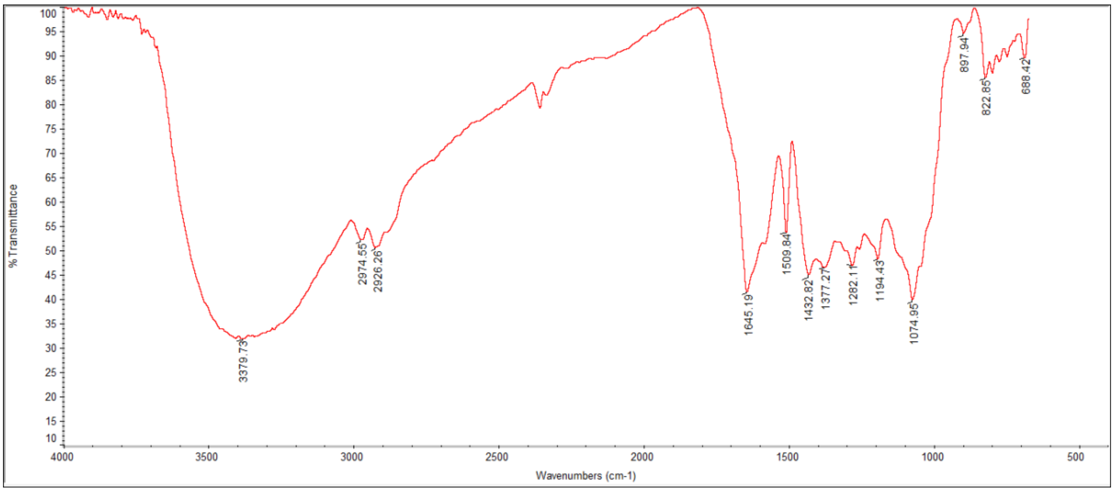

Supplementary Figure 42 IR (KBr disc) spectrum of **4**.

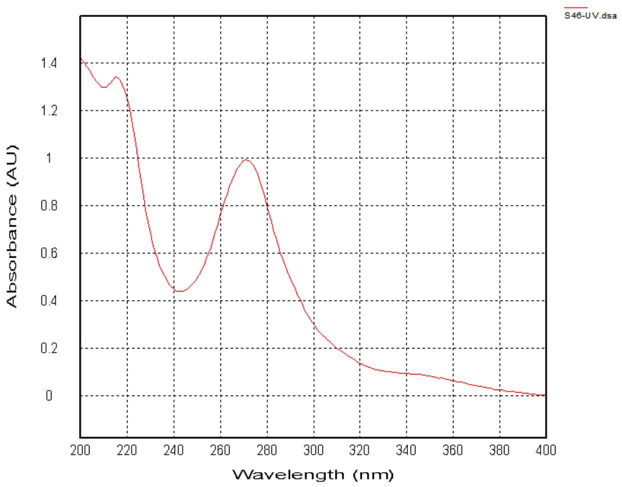

Supplementary Figure 43 UV spectrum of **4**.

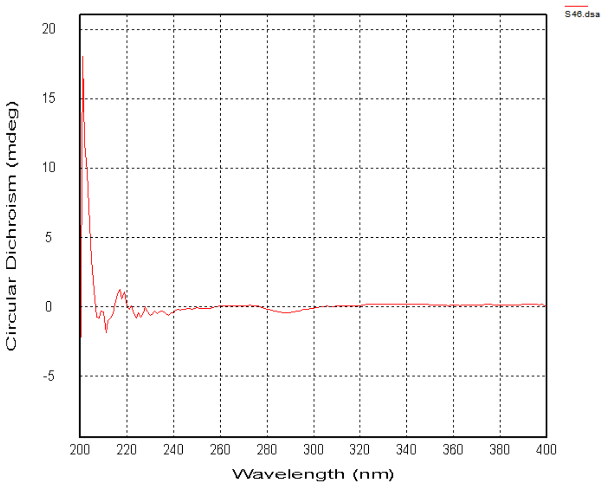

Supplementary Figure 44 ECD spectrum of **4**.

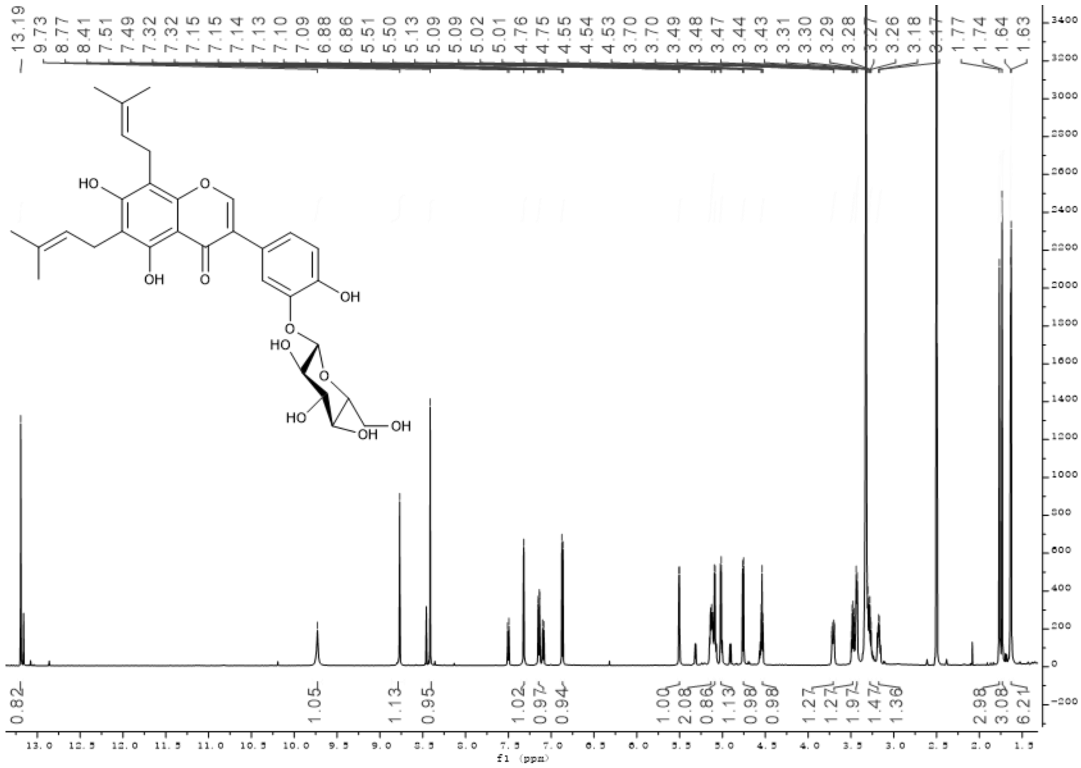

Supplementary Figure 45 ^{1}H NMR (600 MHz) spectrum of **5** in DMSO.

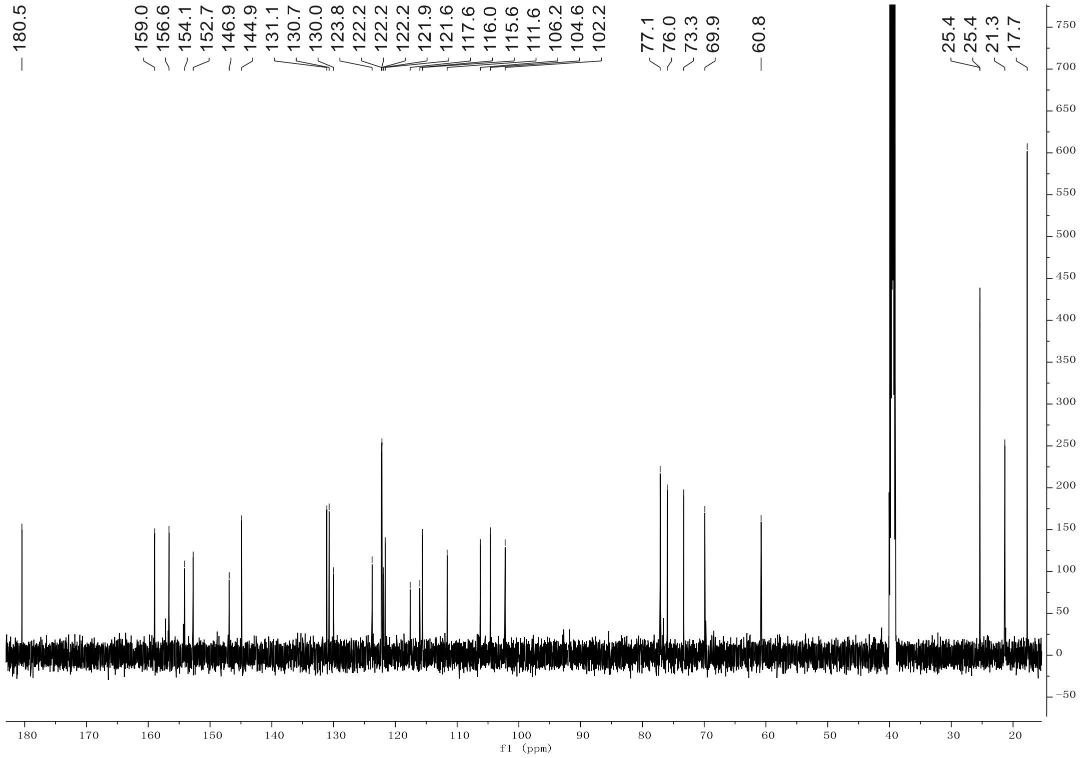

Supplementary Figure 46 ^{13}C NMR (150 MHz) spectrum of **5** in DMSO.

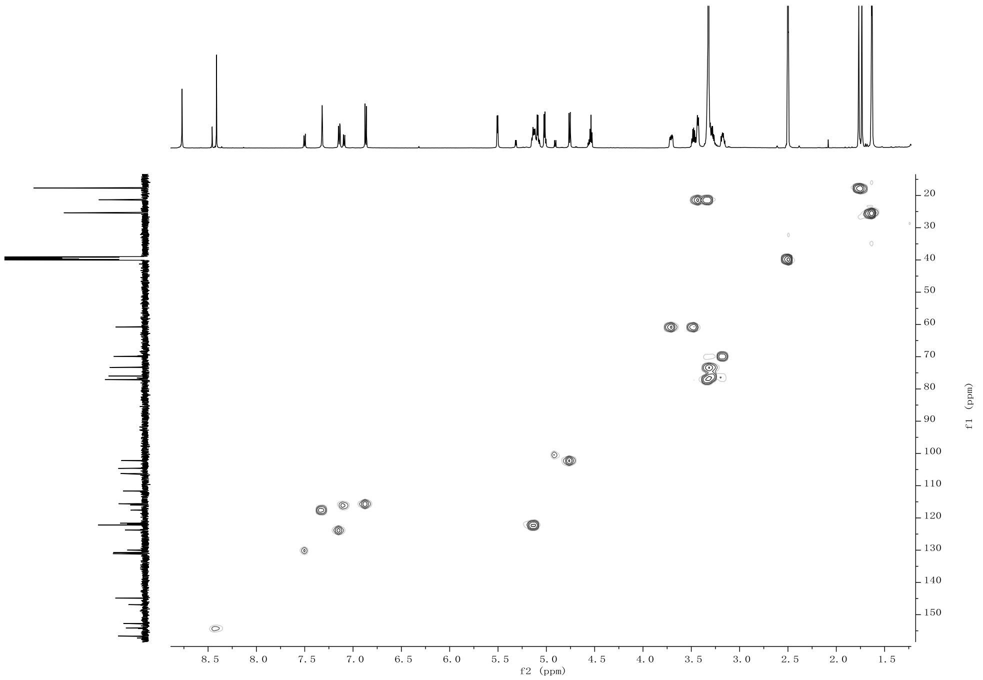

Supplementary Figure 47 HMQC (600 MHz) spectrum of **5** in DMSO.

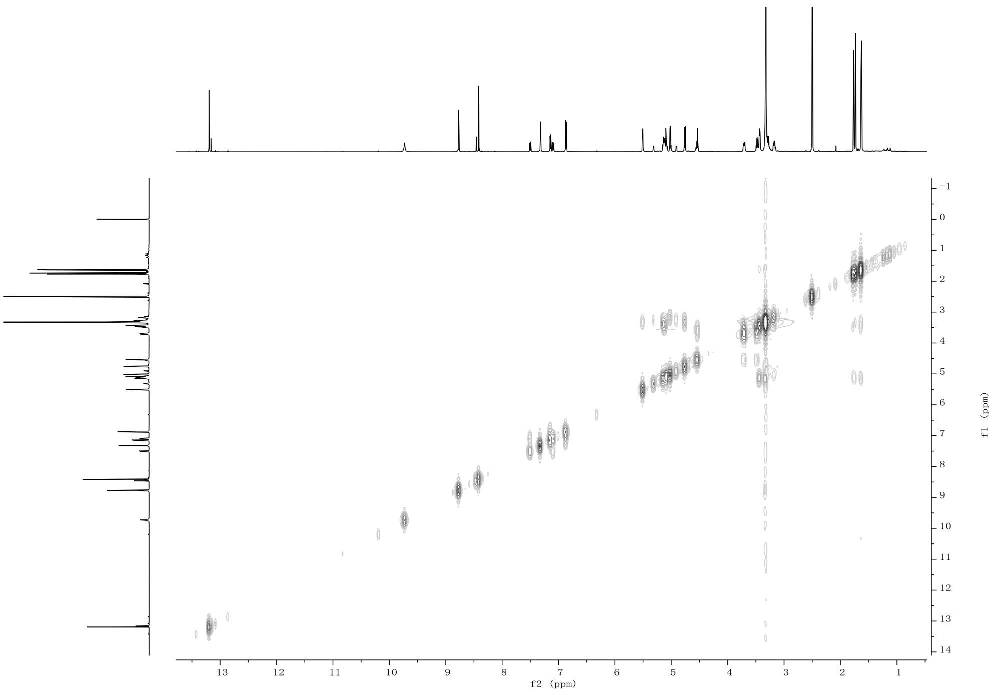

Supplementary Figure 48 ^{1}H-^{1}H COSY (600 MHz) spectrum of **5** in DMSO.

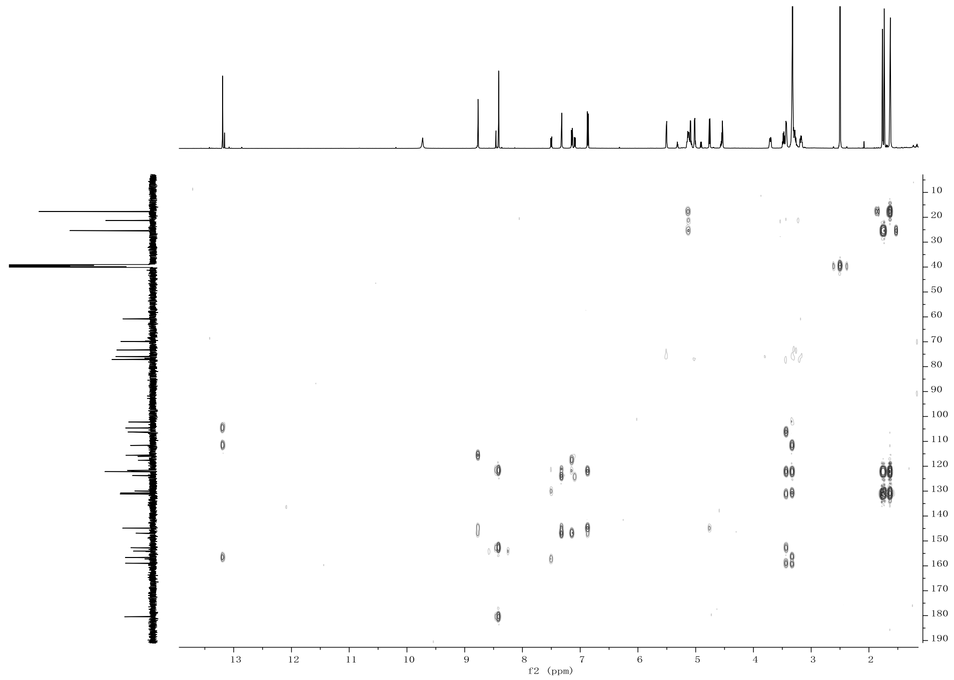

Supplementary Figure 49 HMBC (600 MHz) spectrum of **5** in DMSO.

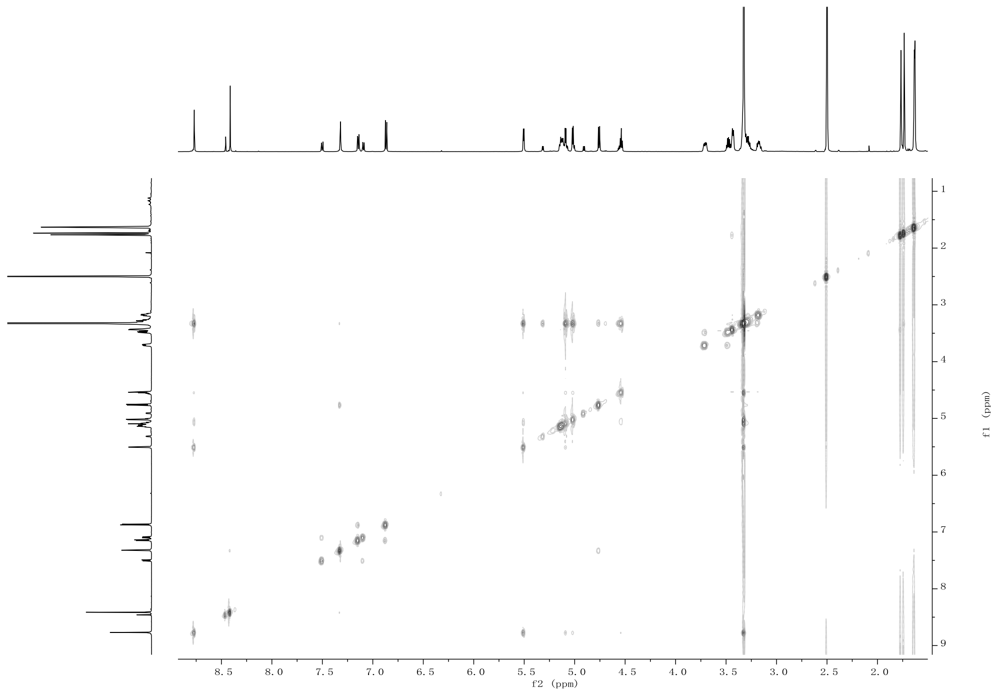

Supplementary Figure 50 ^{1}H-^{1}H NOESY (600 MHz) spectrum of **5** in DMSO.

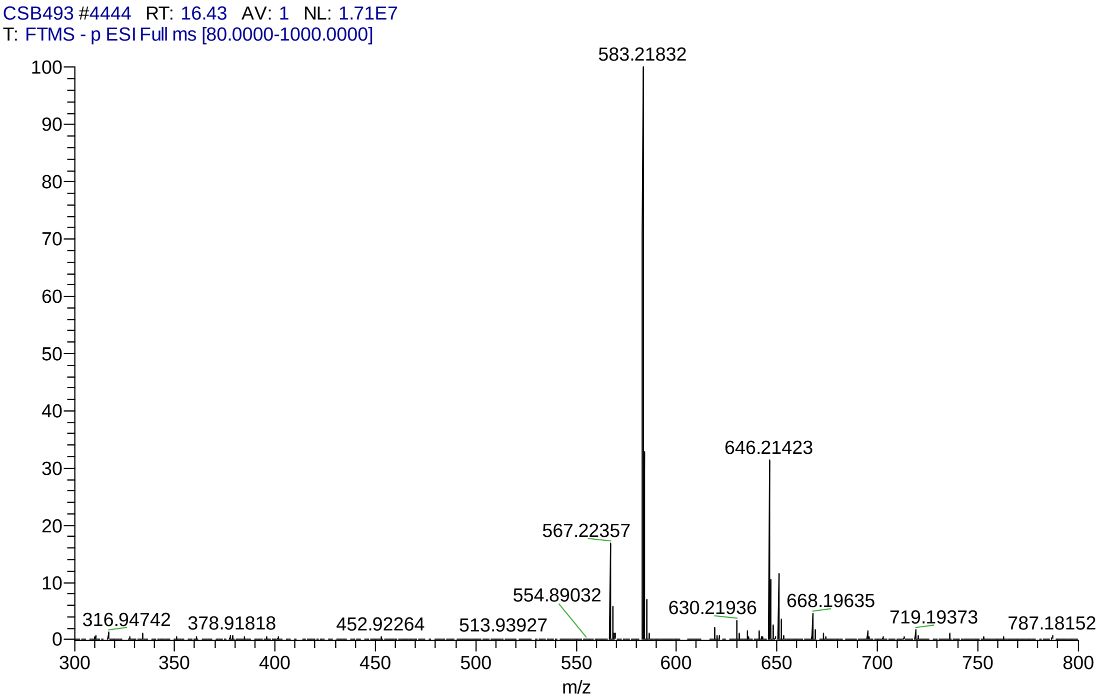

Supplementary Figure 51 HRESIMS spectrum of **5**.

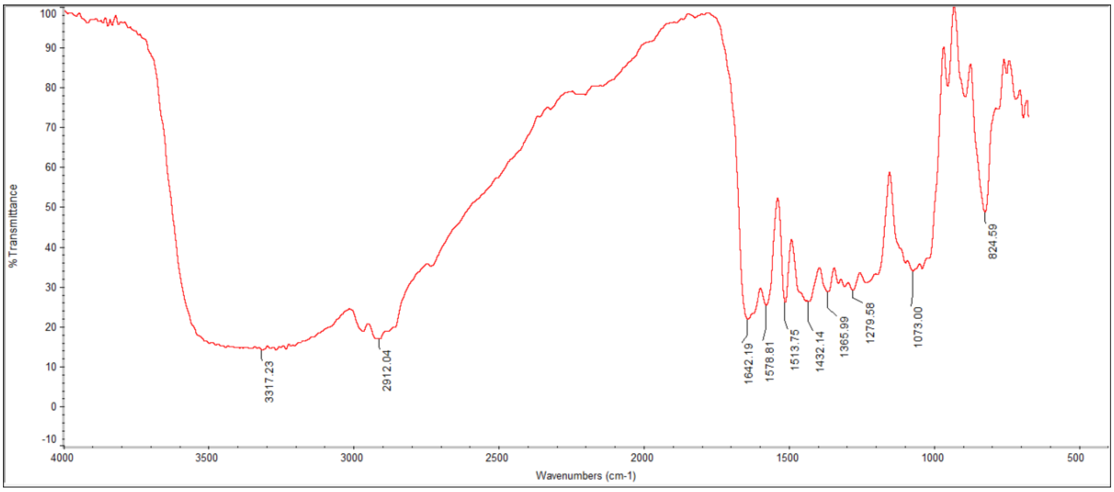

Supplementary Figure 52 IR (KBr disc) spectrum of **5**.

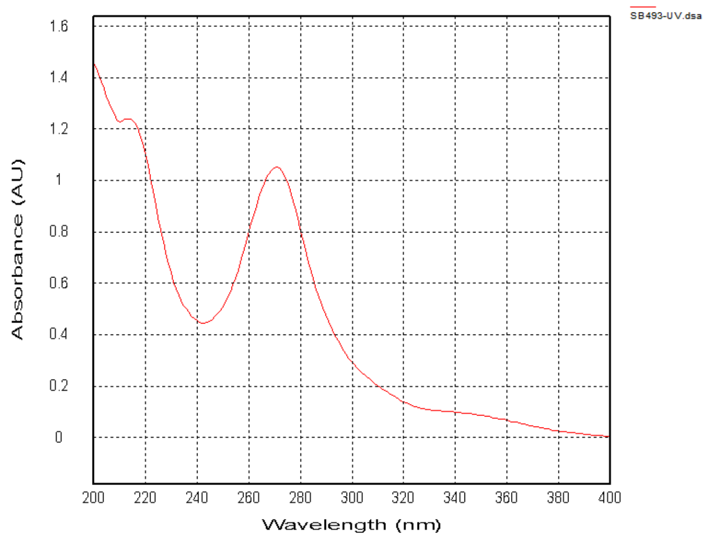

Supplementary Figure 53 UV spectrum of **5**.

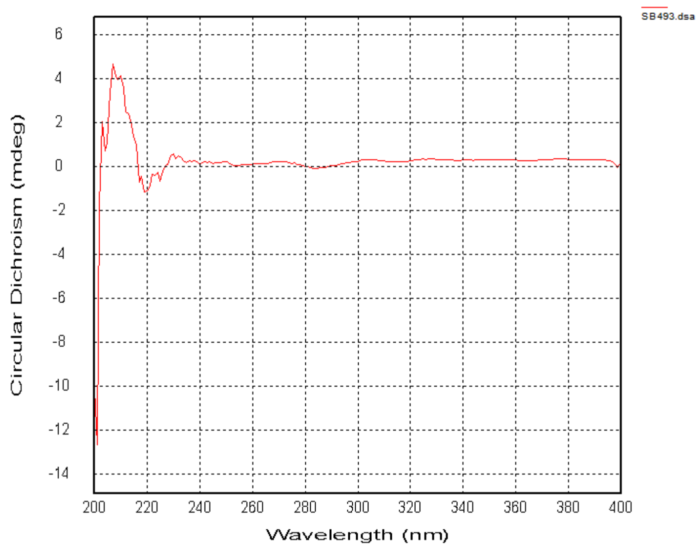

Supplementary Figure 54 ECD spectrum of **5**.

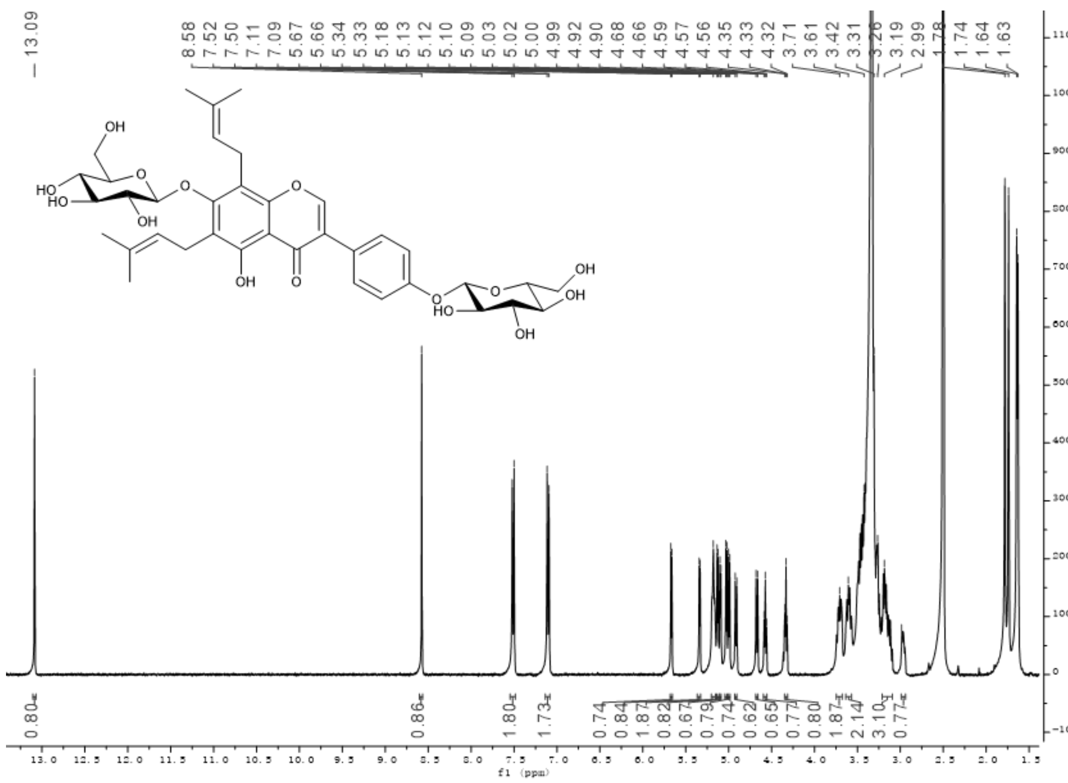

Supplementary Figure 55 ^{1}H NMR (600 MHz) spectrum of **6** in DMSO.

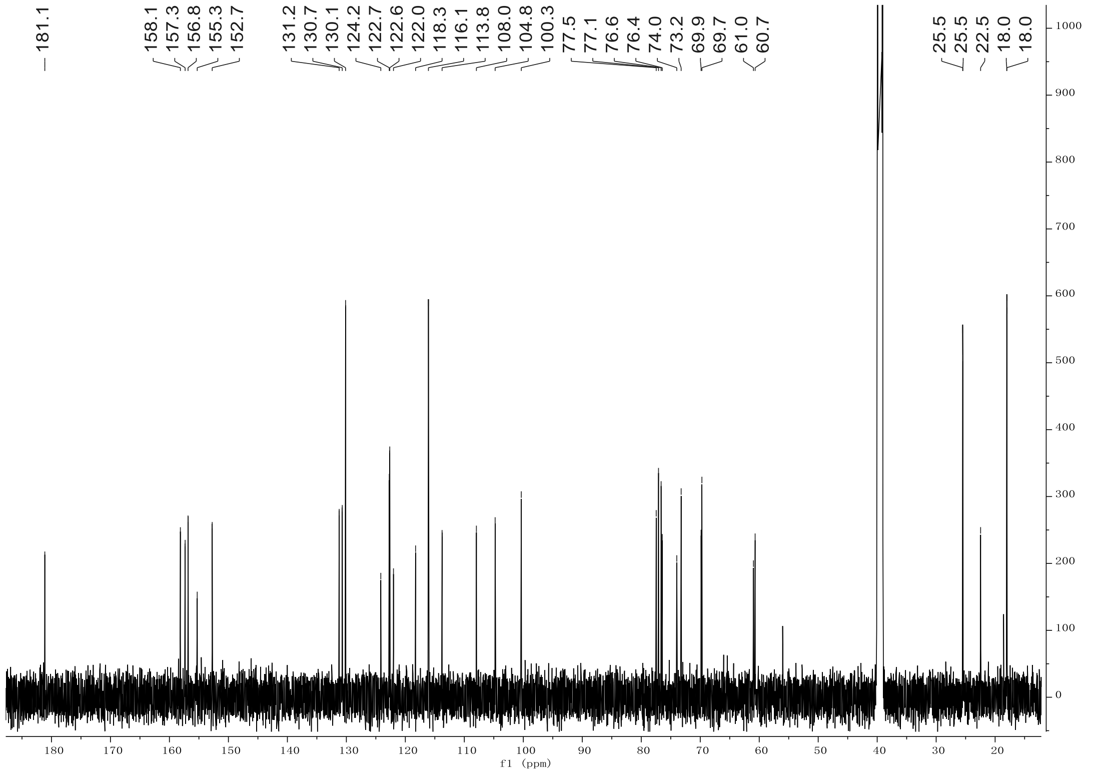

Supplementary Figure 56 ^{13}C NMR (150 MHz) spectrum of **6** in DMSO.

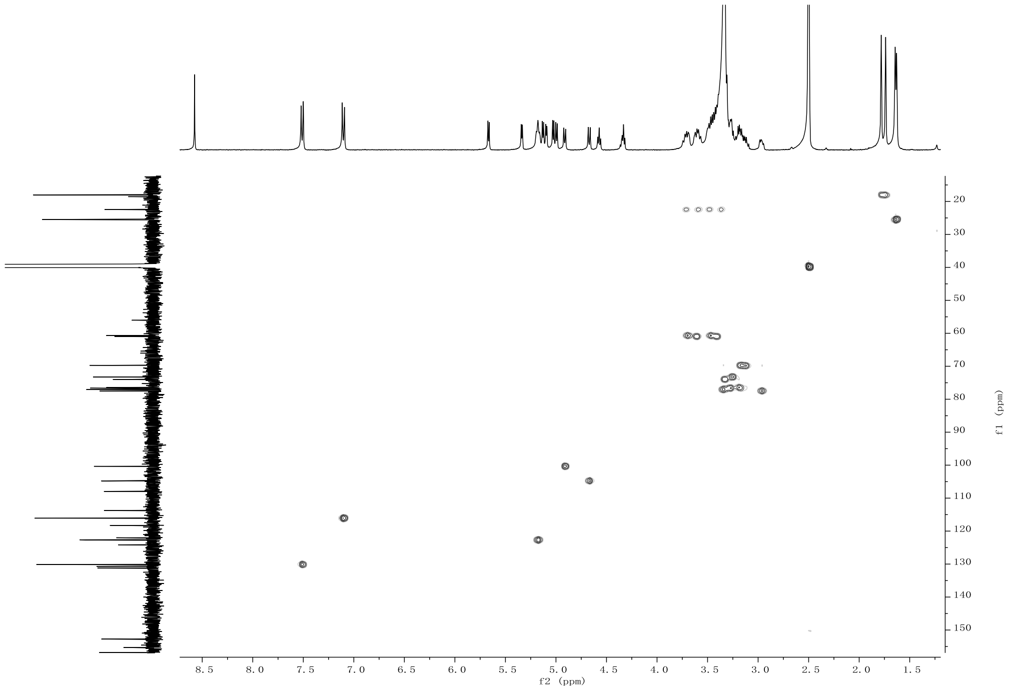

Supplementary Figure 57 HMQC (600 MHz) spectrum of **6 in** DMSO.

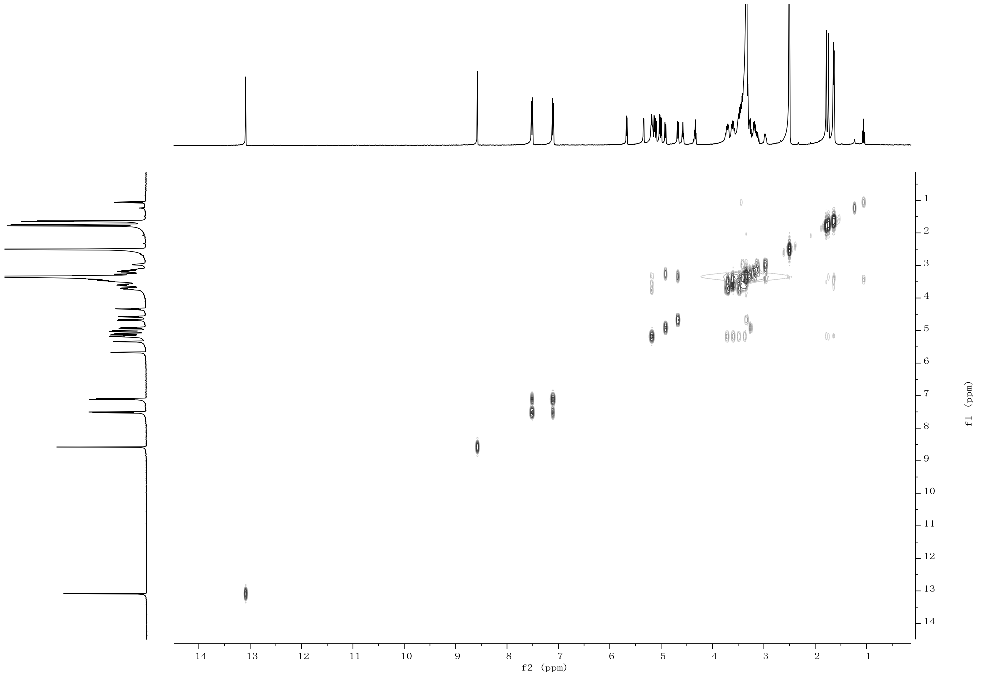

Supplementary Figure 58 ^{1}H-^{1}H COSY (600 MHz) spectrum of **6** in DMSO.

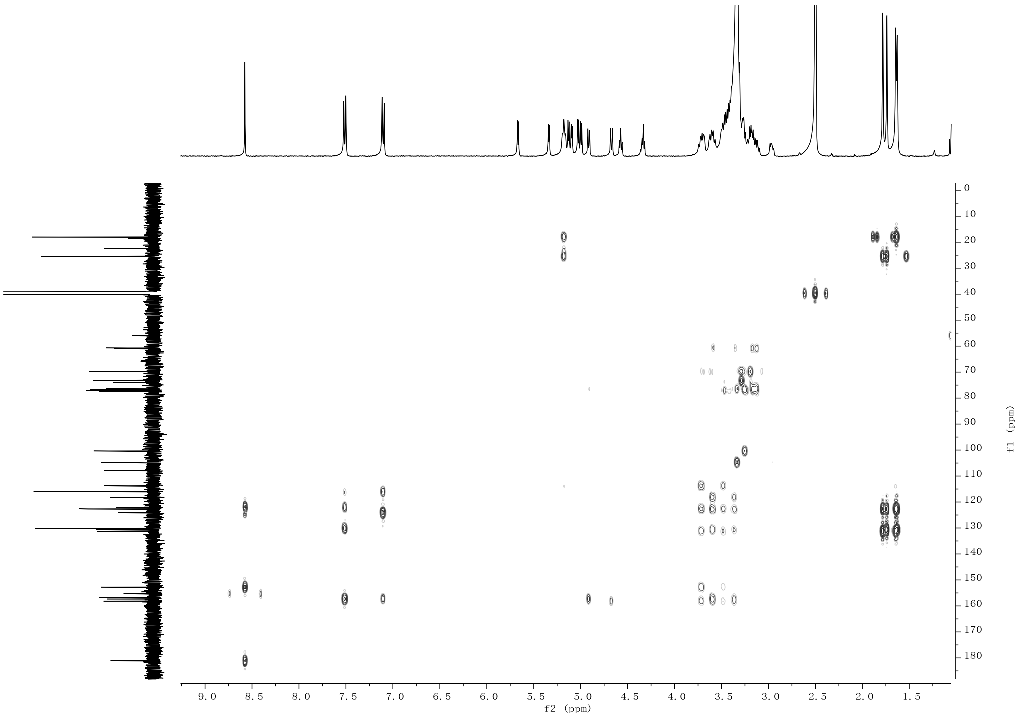

Supplementary Figure 59 HMBC (600 MHz) spectrum of **6** in DMSO.

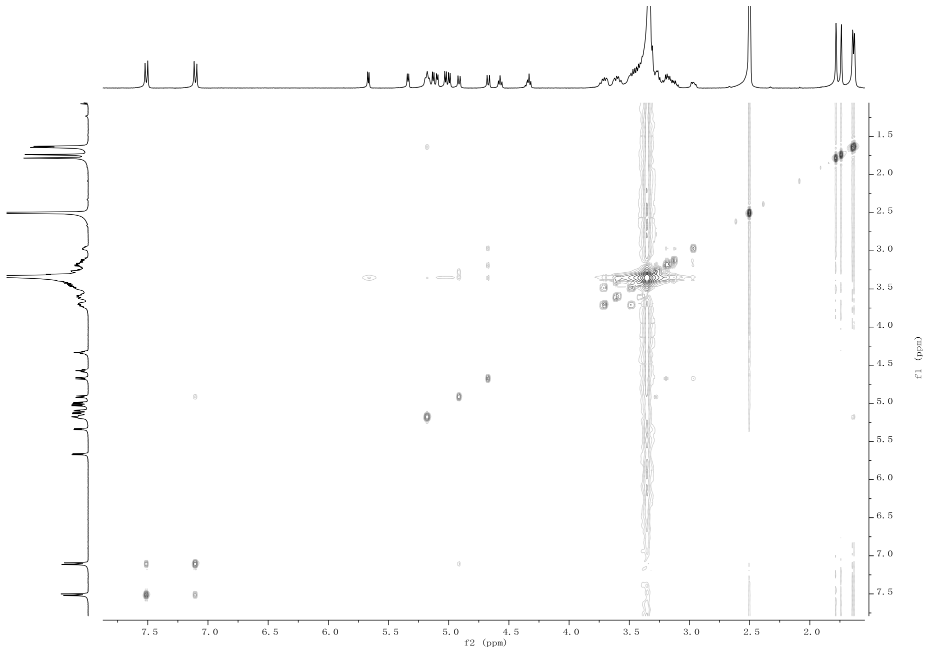

Supplementary Figure 60 ^{1}H-^{1}H NOESY (600 MHz) spectrum of **6** in DMSO.

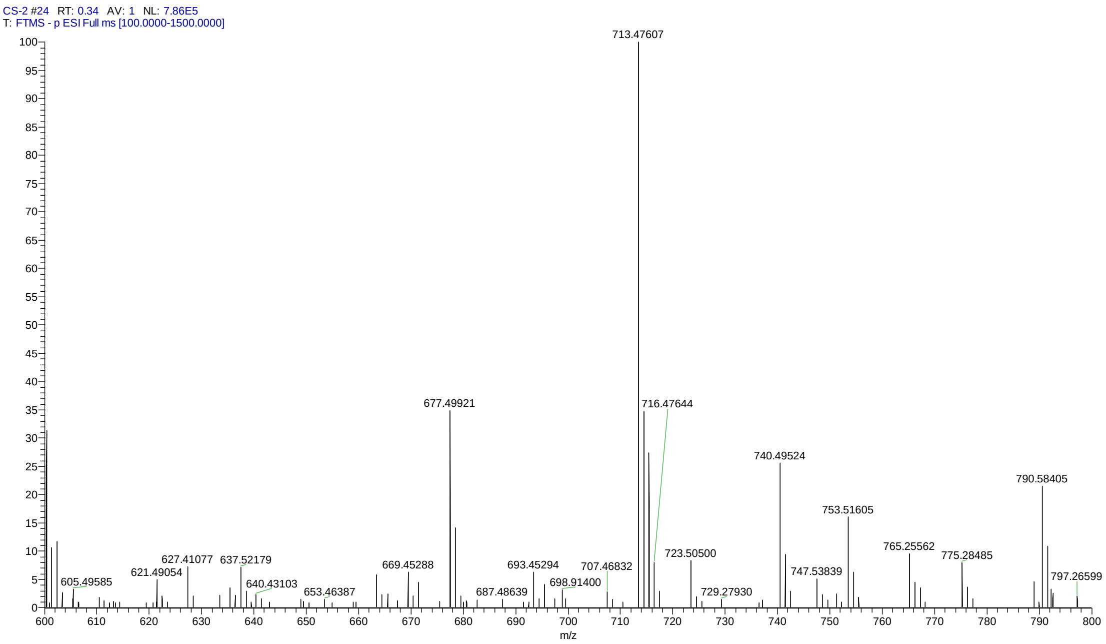

Supplementary Figure 61 HRESIMS spectrum of **6**.

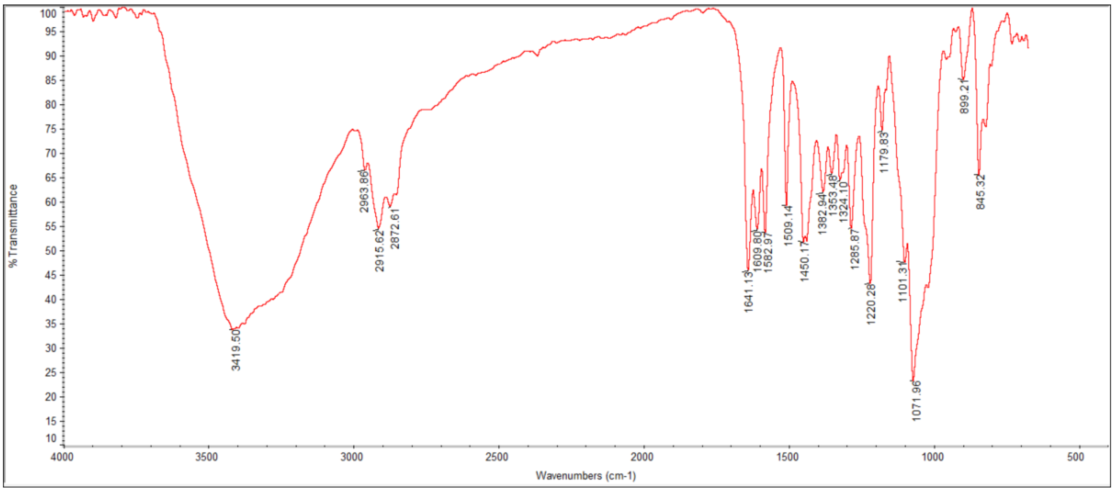

Supplementary Figure 62 IR (KBr disc) spectrum of **6**.

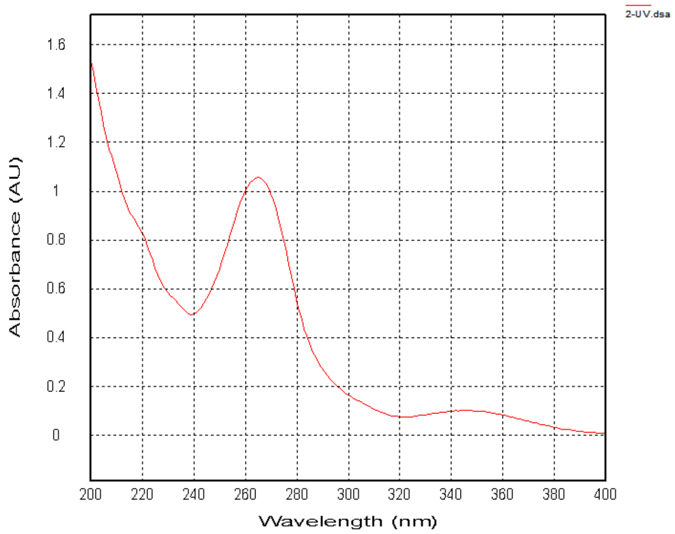

Supplementary Figure 63 UV spectrum of **6**.

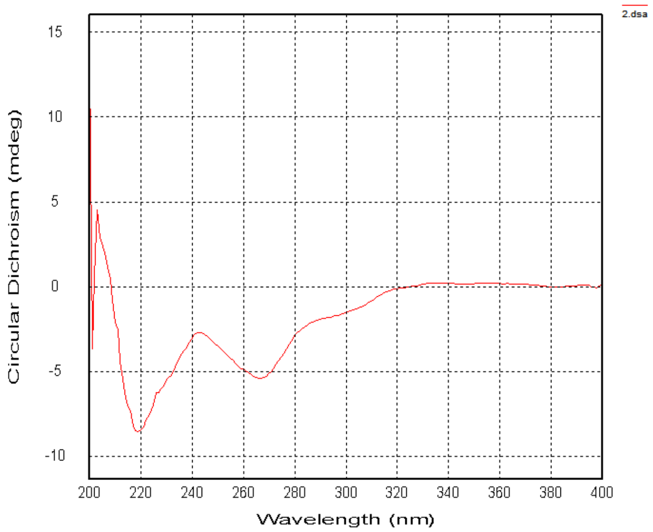

Supplementary Figure 64 ECD spectrum of **6**.

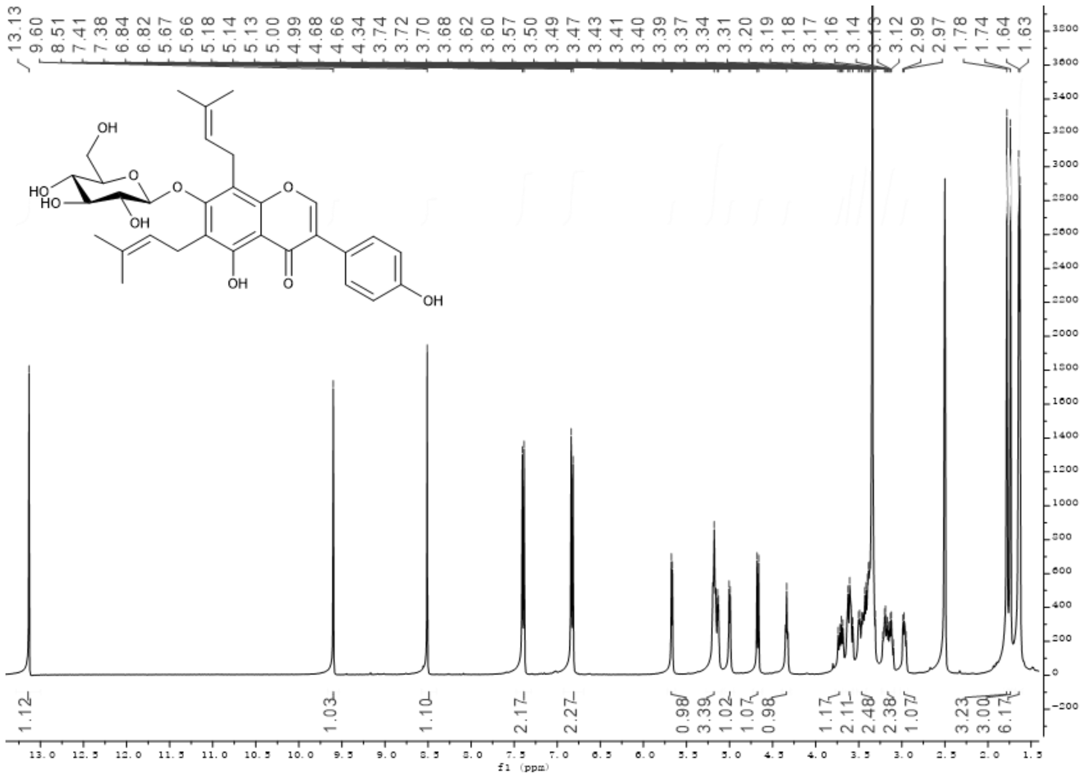

Supplementary Figure 65 ^{1}H NMR (400 MHz) spectrum of **7** in DMSO.

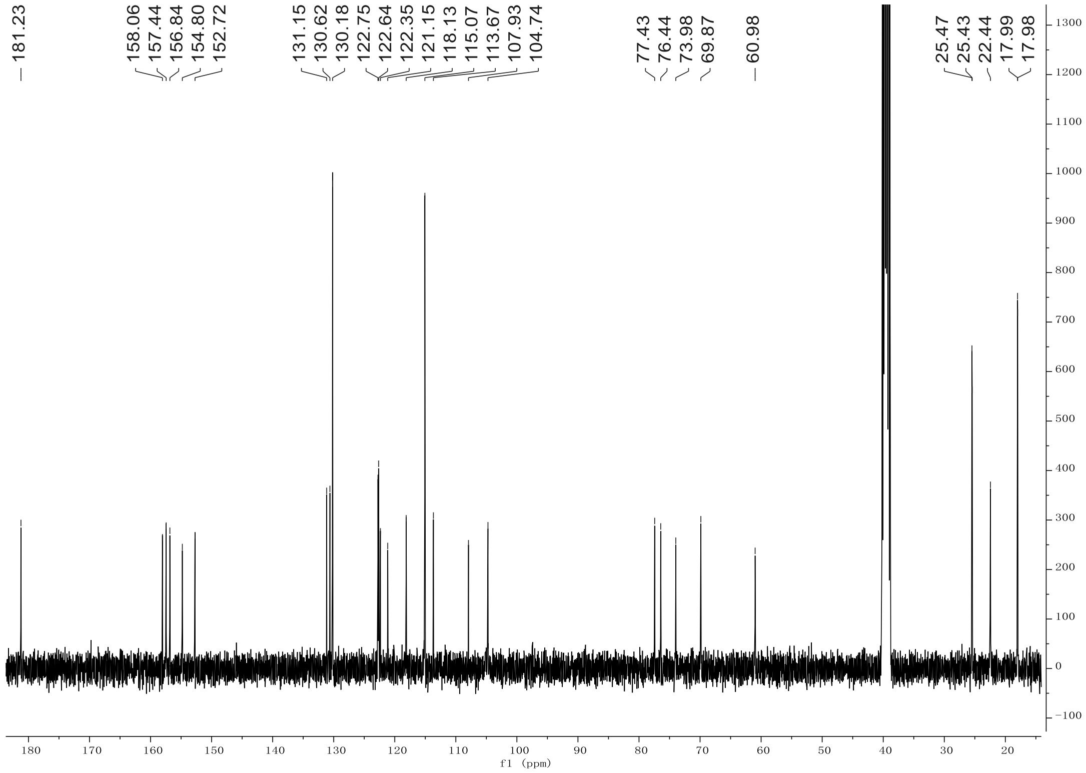

Supplementary Figure 66 ^{13}C NMR (100 MHz) spectrum of **7** in DMSO.

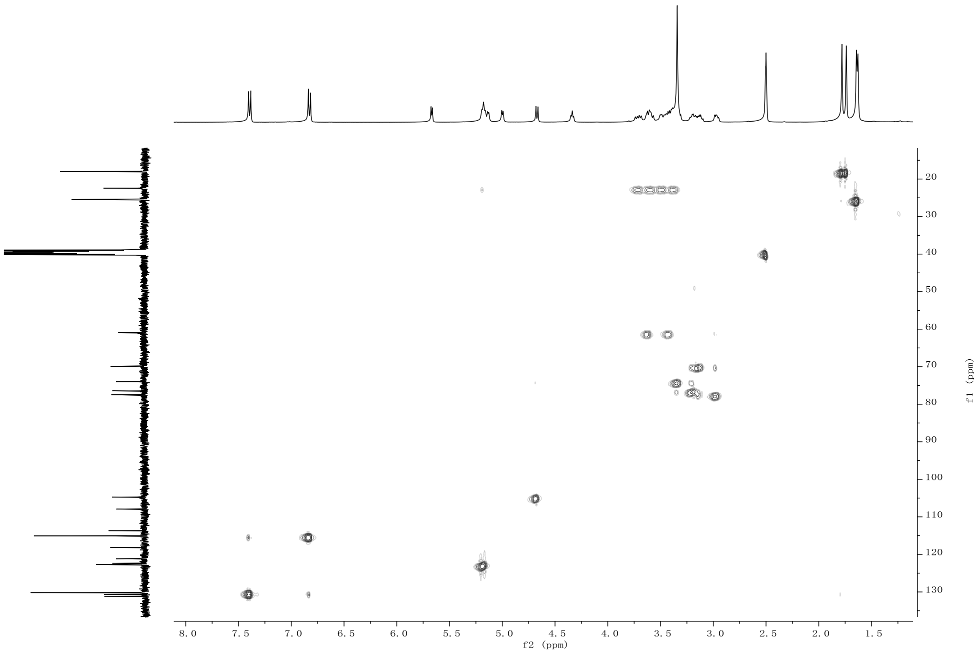

Supplementary Figure 67 HSQC (400 MHz) spectrum of **7** in DMSO.

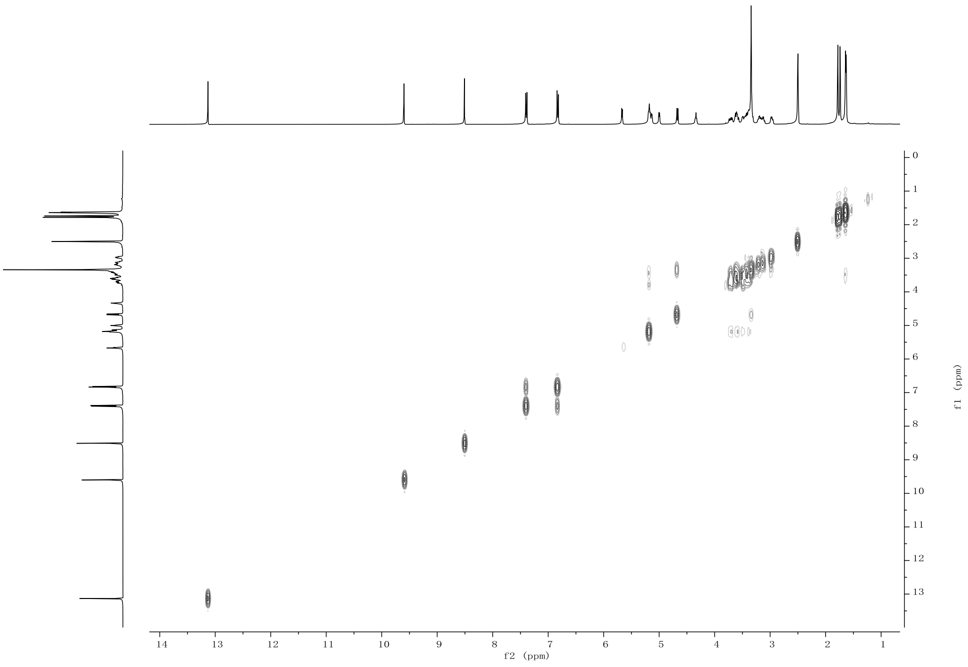

Supplementary Figure 68 ^{1}H-^{1}H COSY (600 MHz) spectrum of **7** in DMSO.

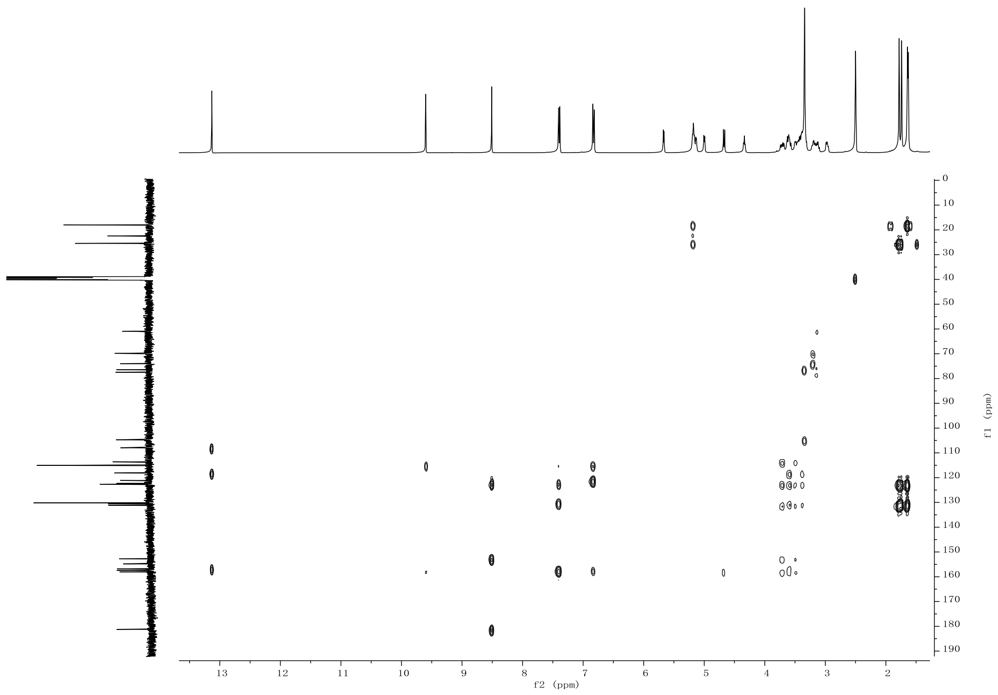

Supplementary Figure 69 HMBC (400 MHz) spectrum of **7** in DMSO.

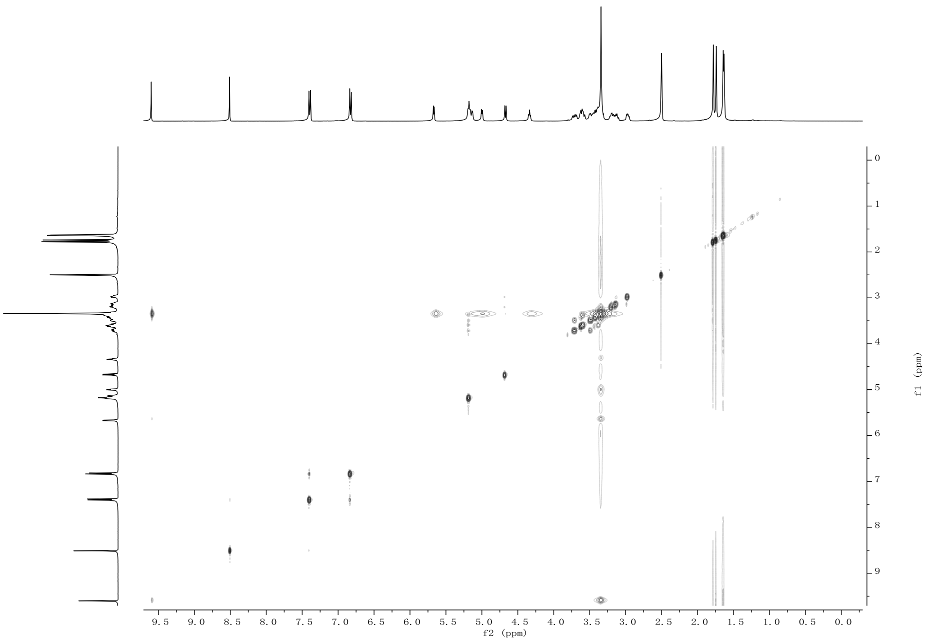

Supplementary Figure 70 ^{1}H-^{1}H NOESY (600 MHz) spectrum of **7** in DMSO.

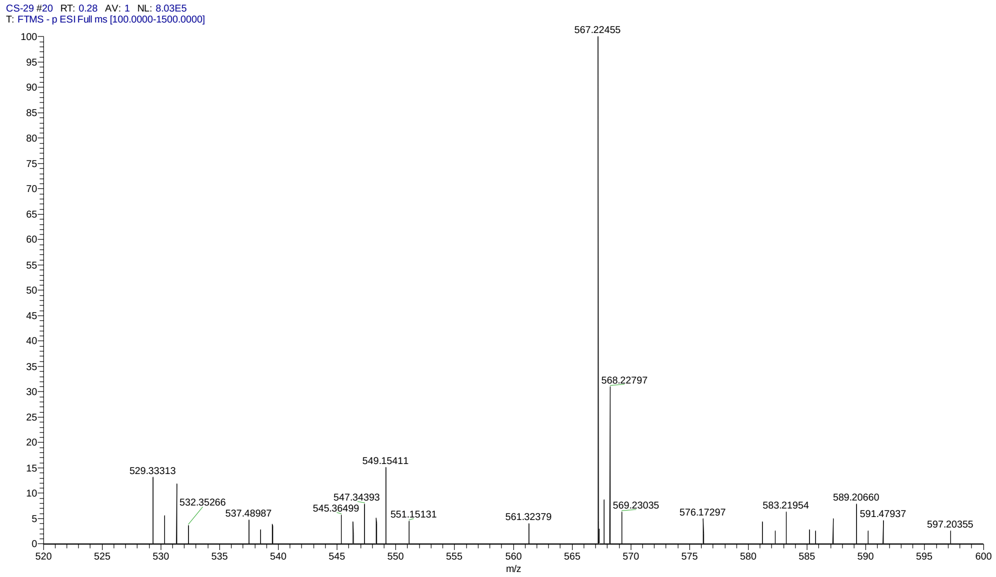

Supplementary Figure 71 HRESIMS spectrum of **7**.

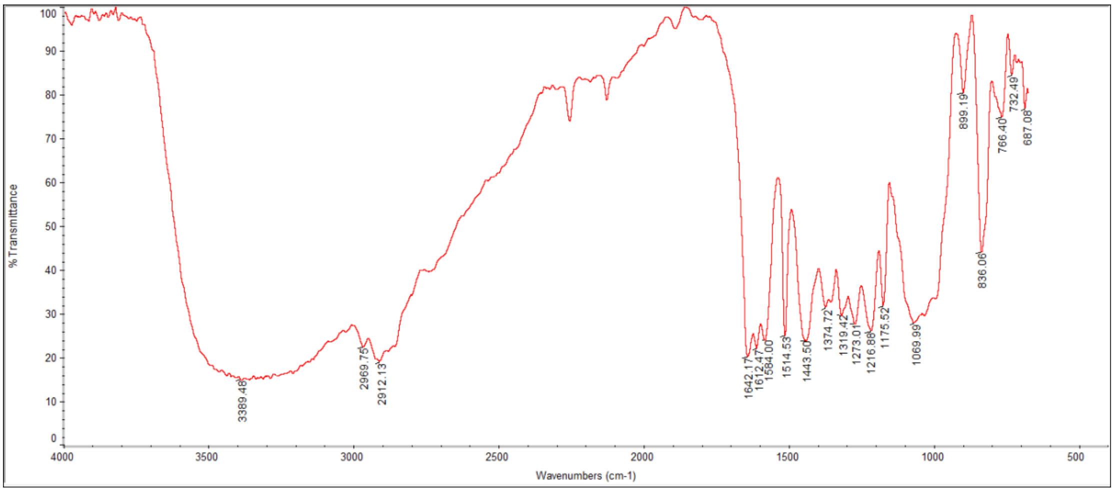

Supplementary Figure 72 IR (KBr disc) spectrum of **7**.

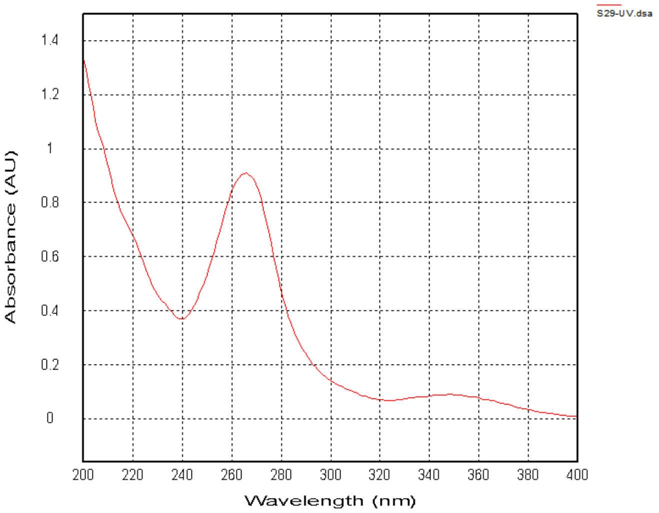

Supplementary Figure 73 UV spectrum of **7**.

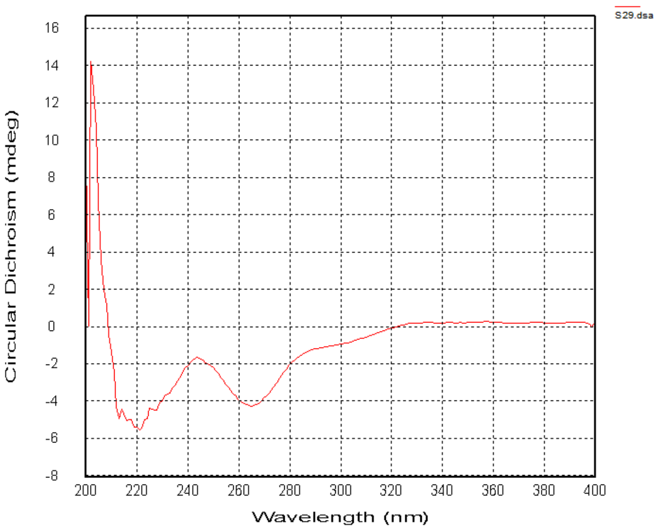

Supplementary Figure 74 ECD spectrum of **7**.

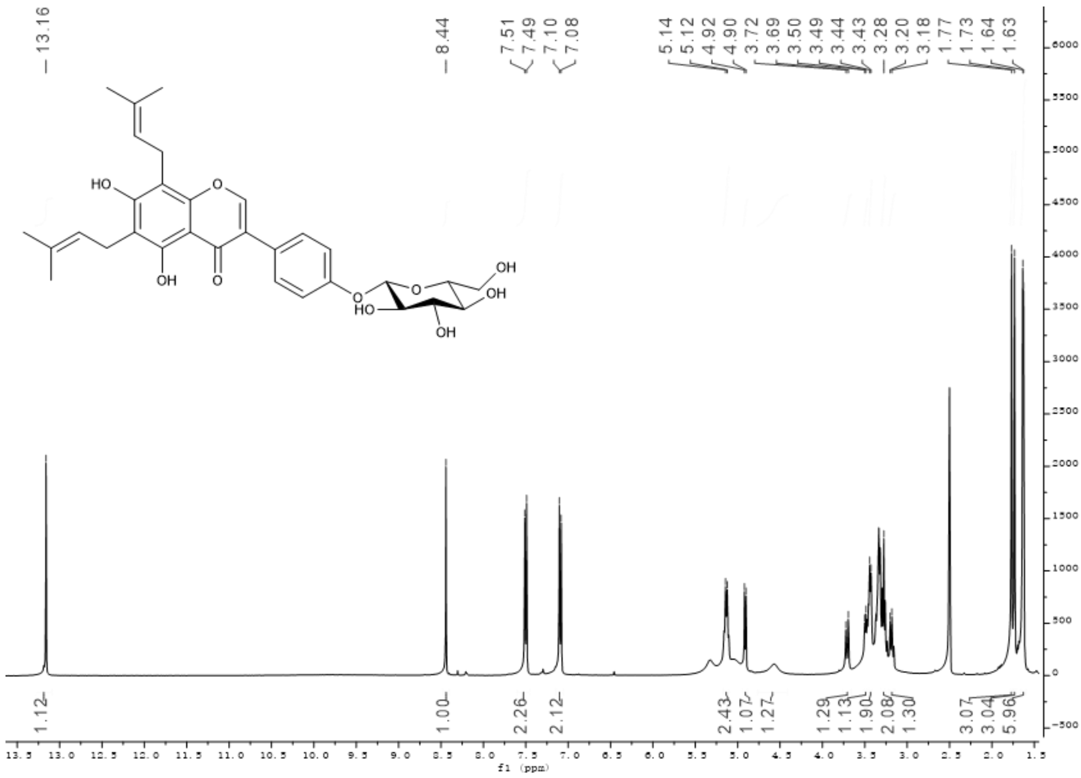

Supplementary Figure 75 ^{1}H NMR (400 MHz) spectrum of **8** in DMSO.

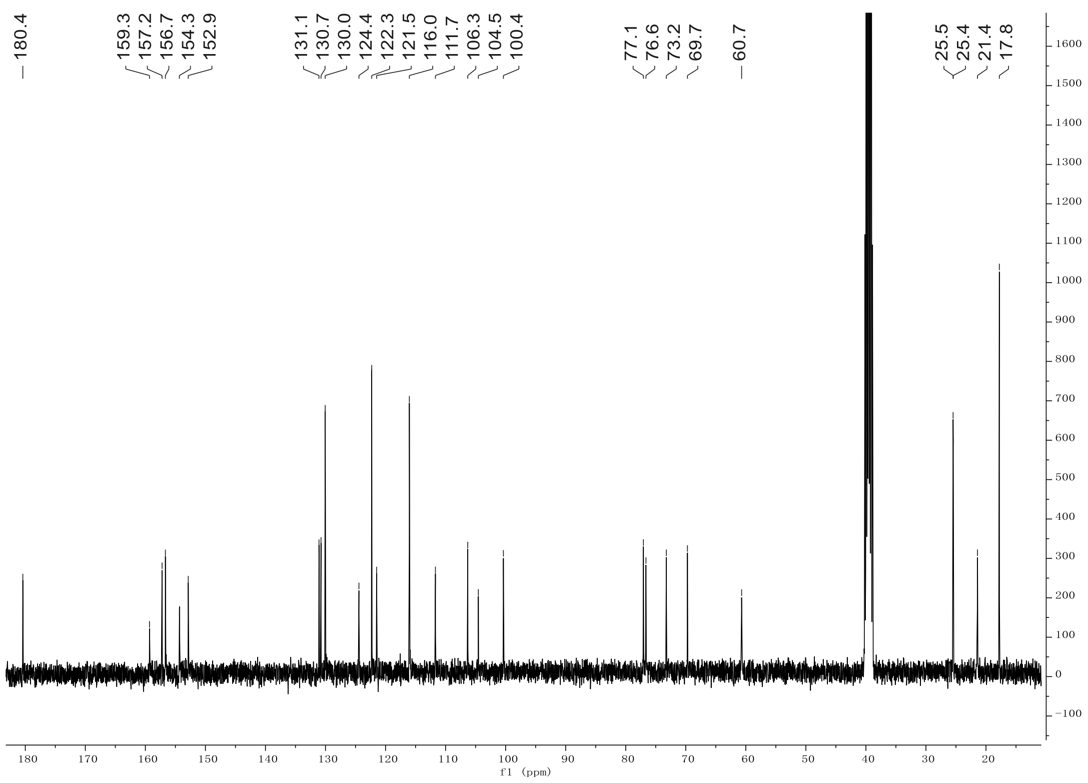

Supplementary Figure 76 ^{13}C NMR (100 MHz) spectrum of **8** in DMSO.

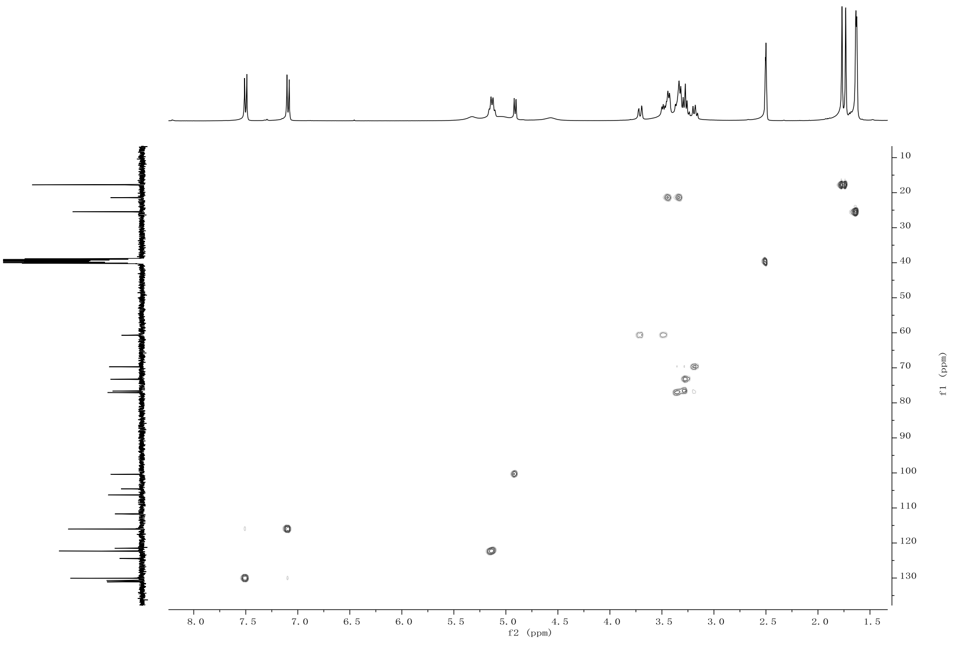

Supplementary Figure 77 HSQC (400 MHz) spectrum of **8** in DMSO.

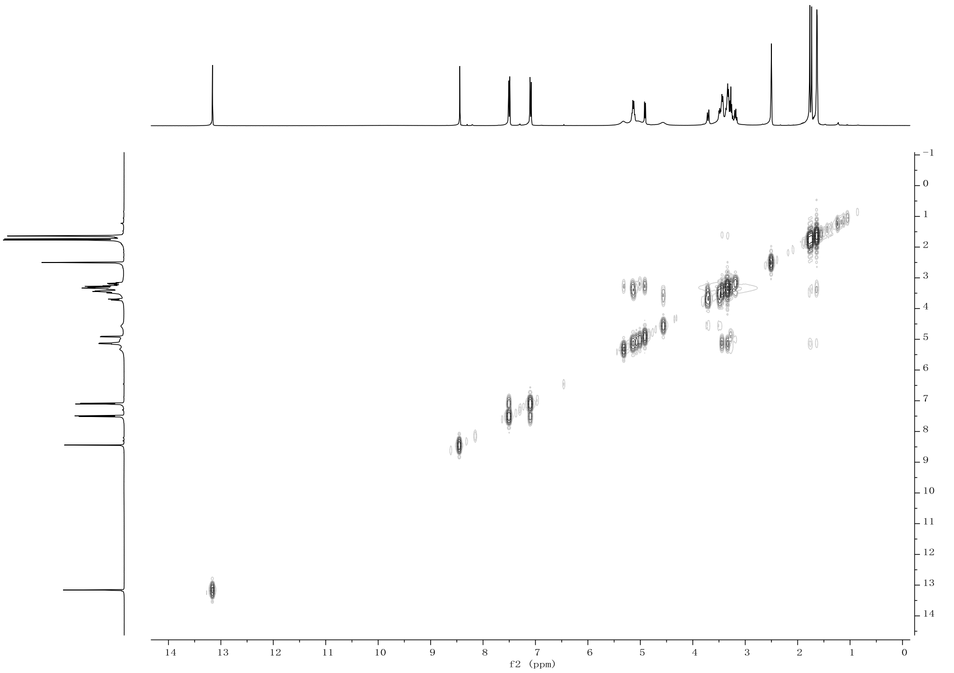

Supplementary Figure 78 ^{1}H-^{1}H COSY (600 MHz) spectrum of **8** in DMSO.

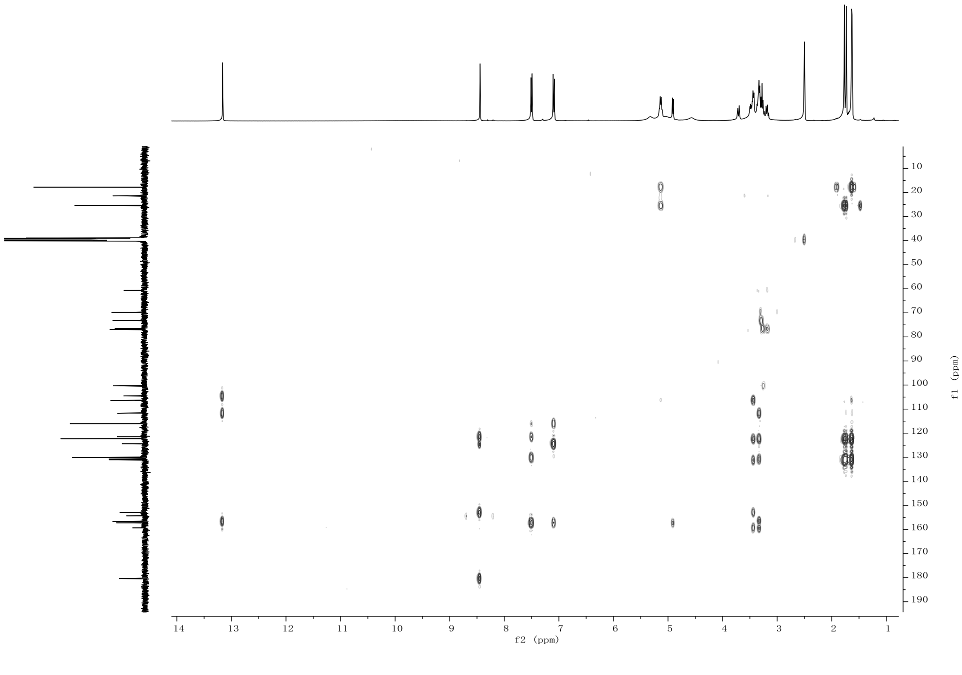

Supplementary Figure 79 HMBC (400 MHz) spectrum of **8** in DMSO.

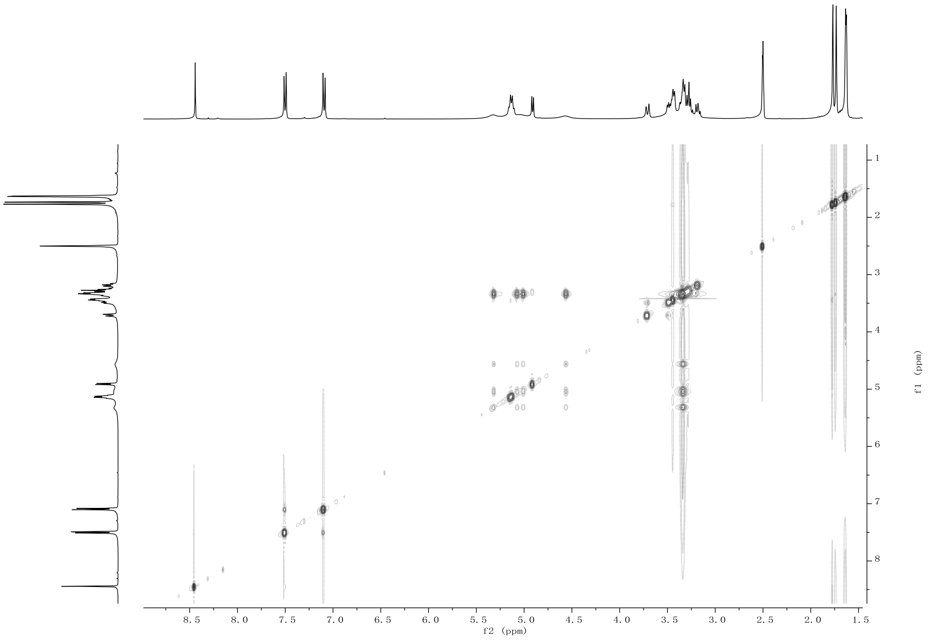

Supplementary Figure 80 ^{1}H-^{1}H NOESY (600 MHz) spectrum of **8** in DMSO.

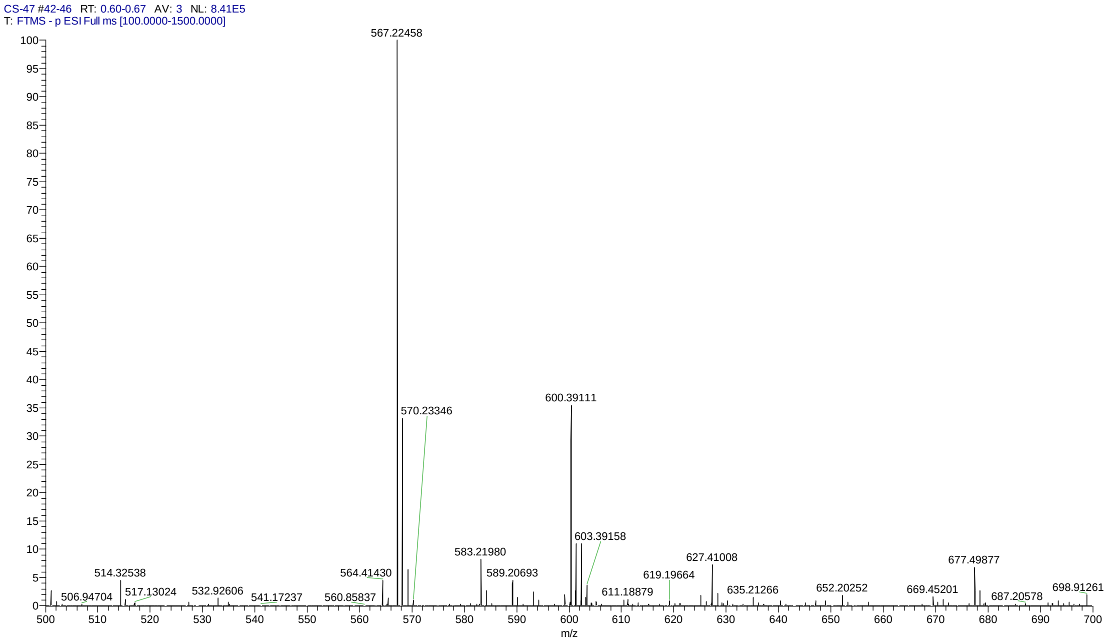

Supplementary Figure 81 HRESIMS spectrum of **8**.

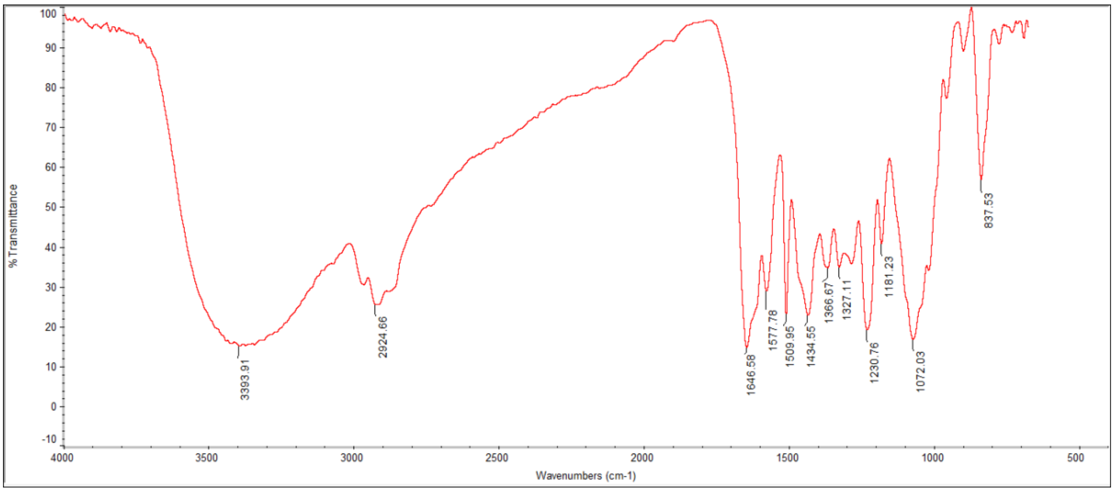

Supplementary Figure 82 IR (KBr disc) spectrum of **8**.

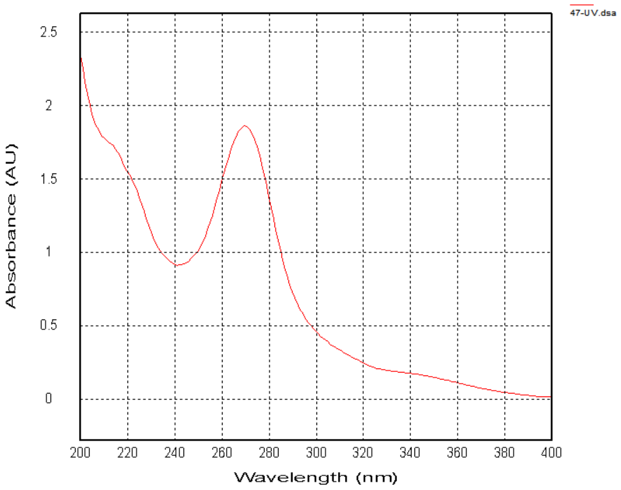

Supplementary Figure 83 UV spectrum of **8**.

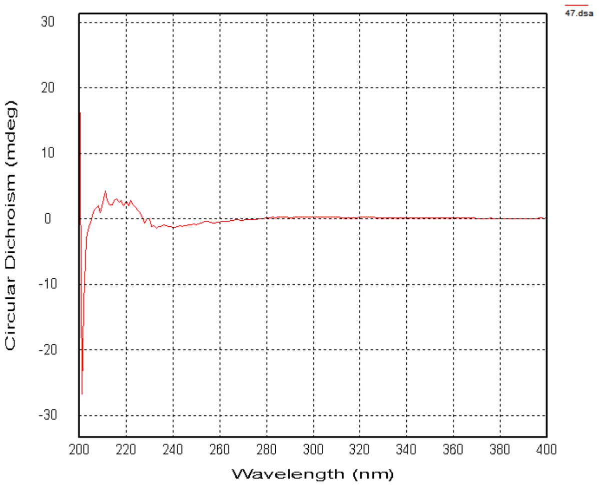

Supplementary Figure 84 ECD spectrum of **8**.

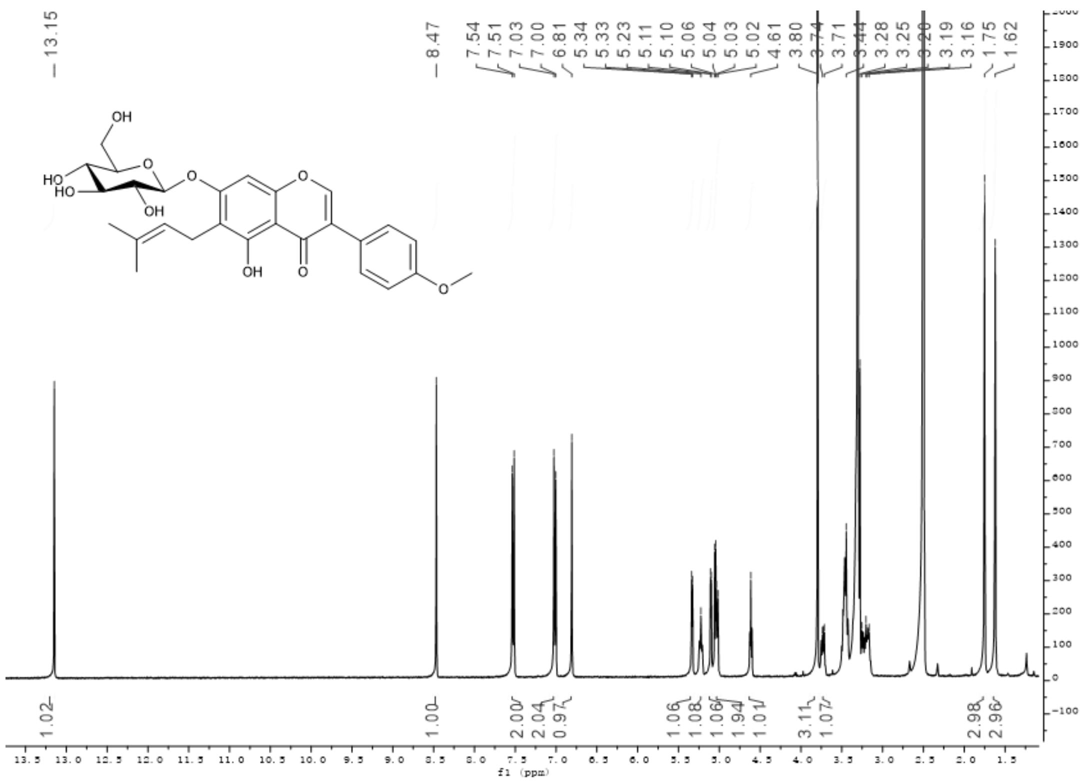

Supplementary Figure 85 ^{1}H NMR (400 MHz) spectrum of **9** in DMSO.

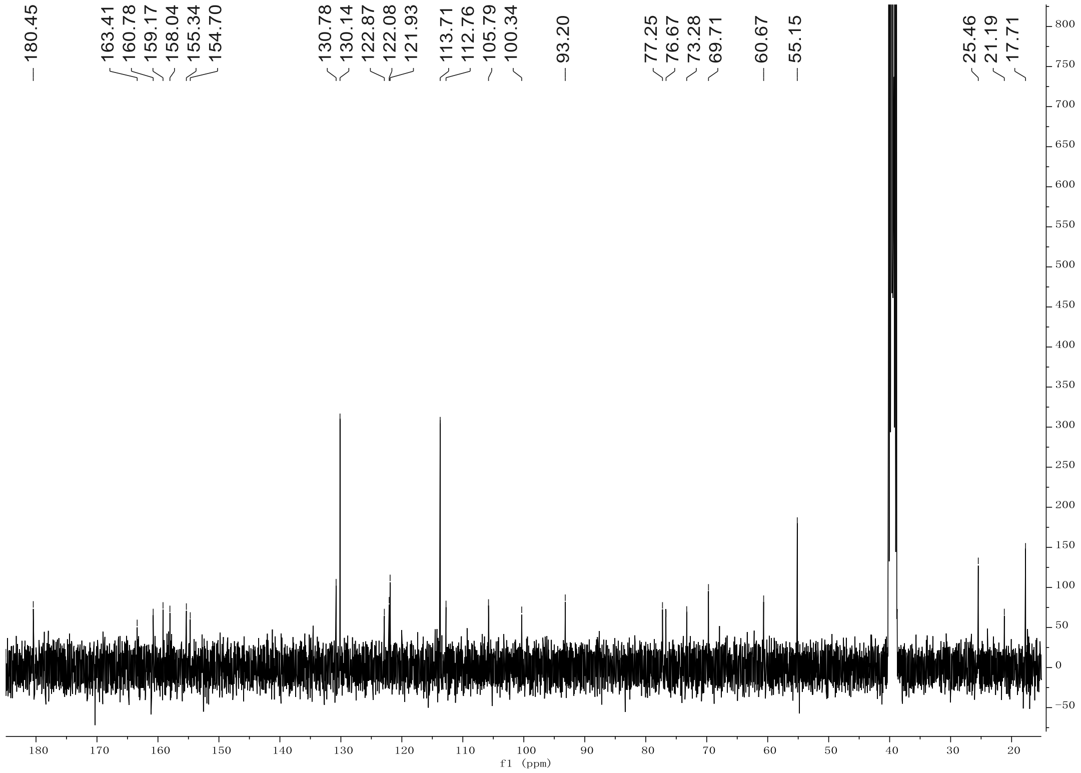

Supplementary Figure 86 ^{13}C NMR (100 MHz) spectrum of **9** in DMSO.

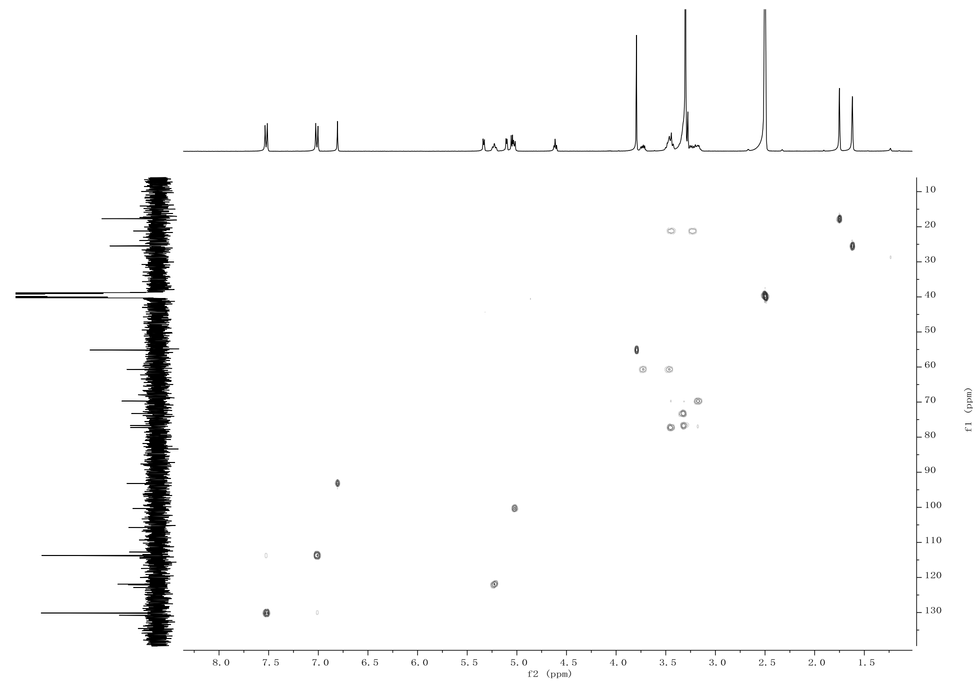

Supplementary Figure 87 HSQC (400 MHz) spectrum of **9** in DMSO.

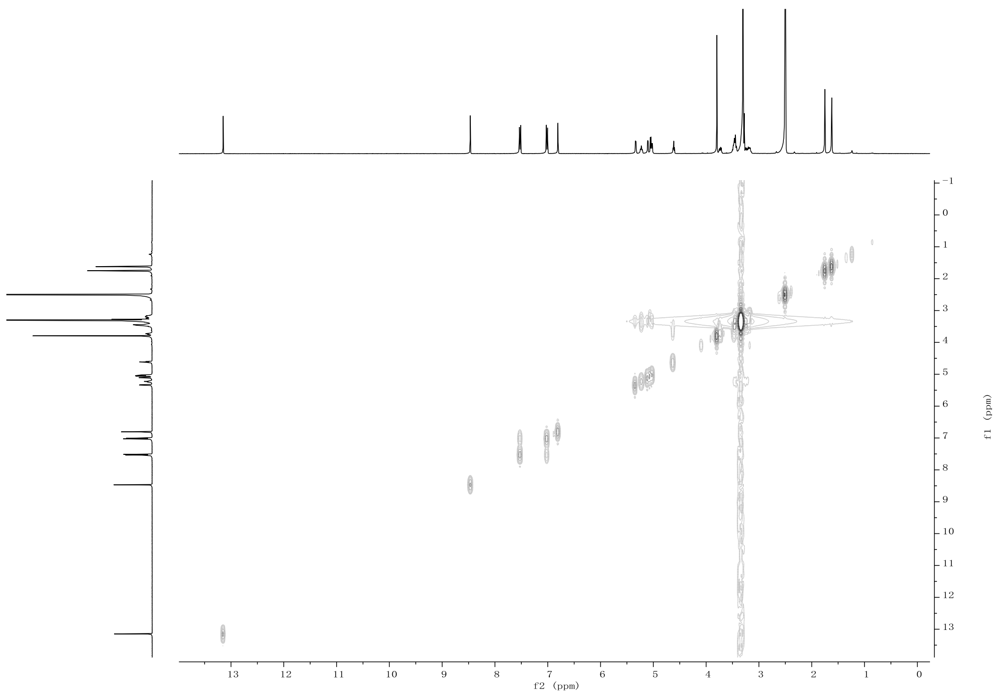

Supplementary Figure 88 ^{1}H-^{1}H COSY (600 MHz) spectrum of **9** in DMSO.

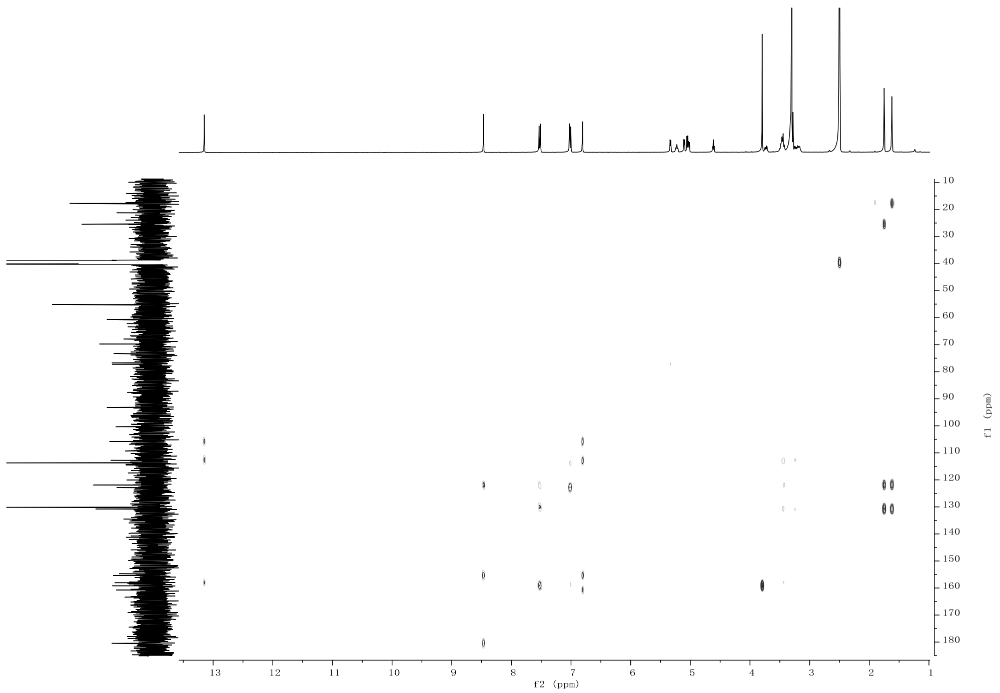

Supplementary Figure 89 HMBC (400 MHz) spectrum of **9** in DMSO.

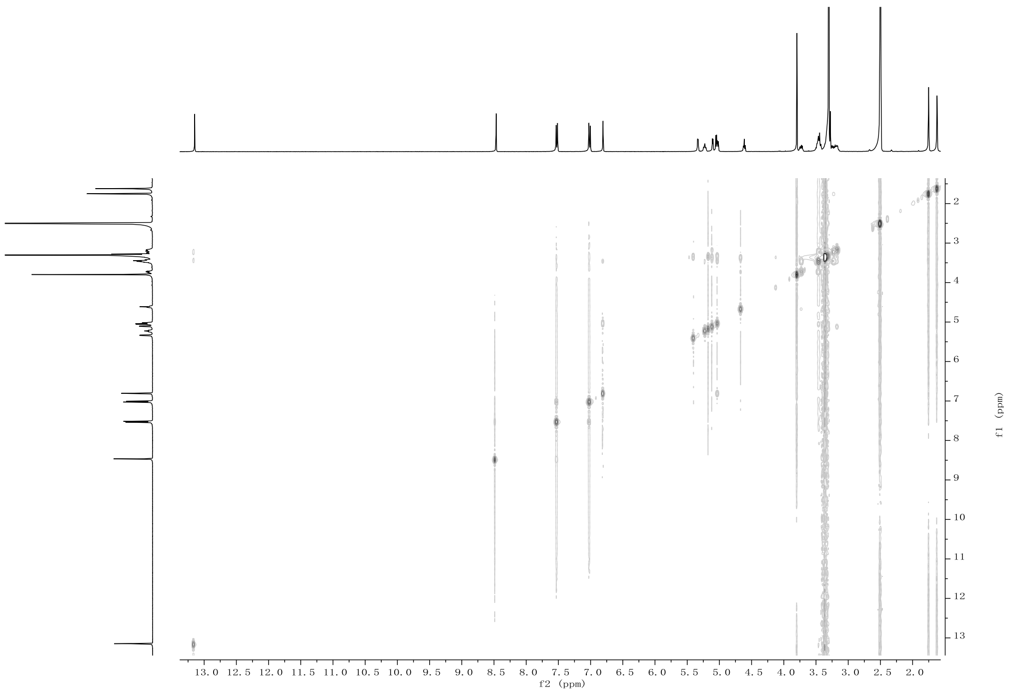

Supplementary Figure 90 ^{1}H-^{1}H NOESY (600 MHz) spectrum of **9** in DMSO.

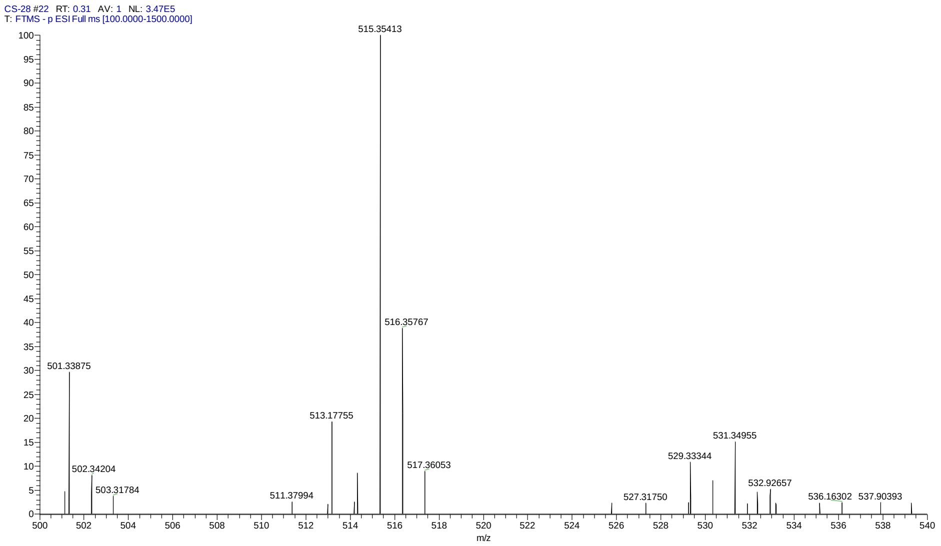

Supplementary Figure 91 HRESIMS spectrum of **9**.

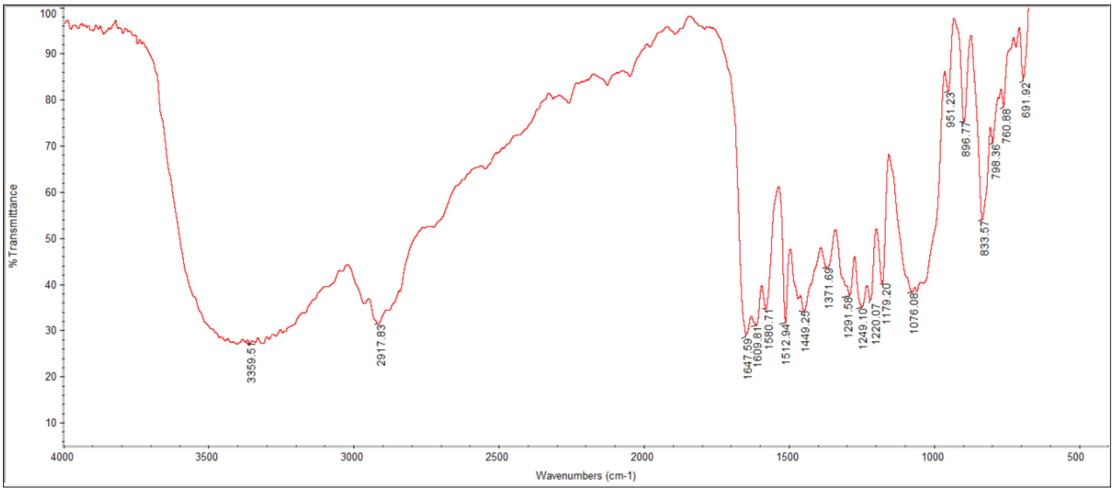

Supplementary Figure 92 IR (KBr disc) spectrum of **9**.

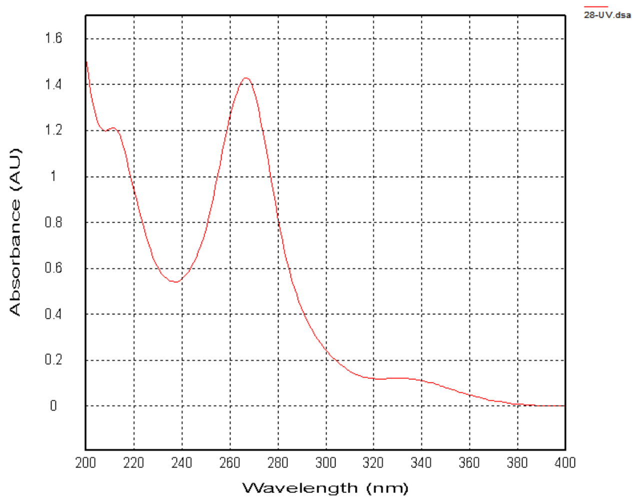

Supplementary Figure 93 UV spectrum of **9**.

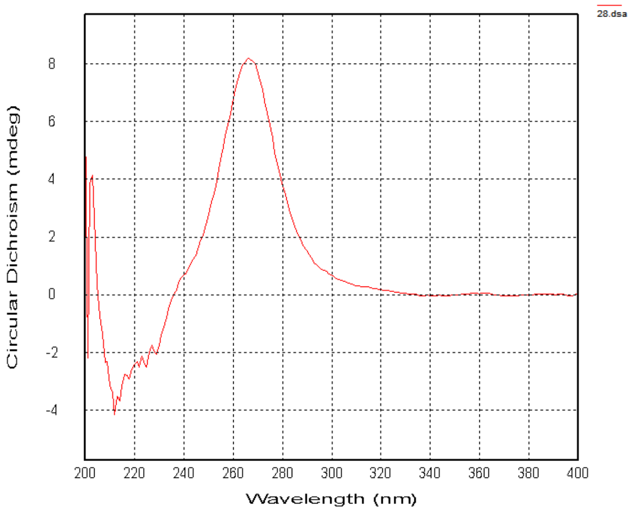

Supplementary Figure 94 ECD spectrum of **9**.

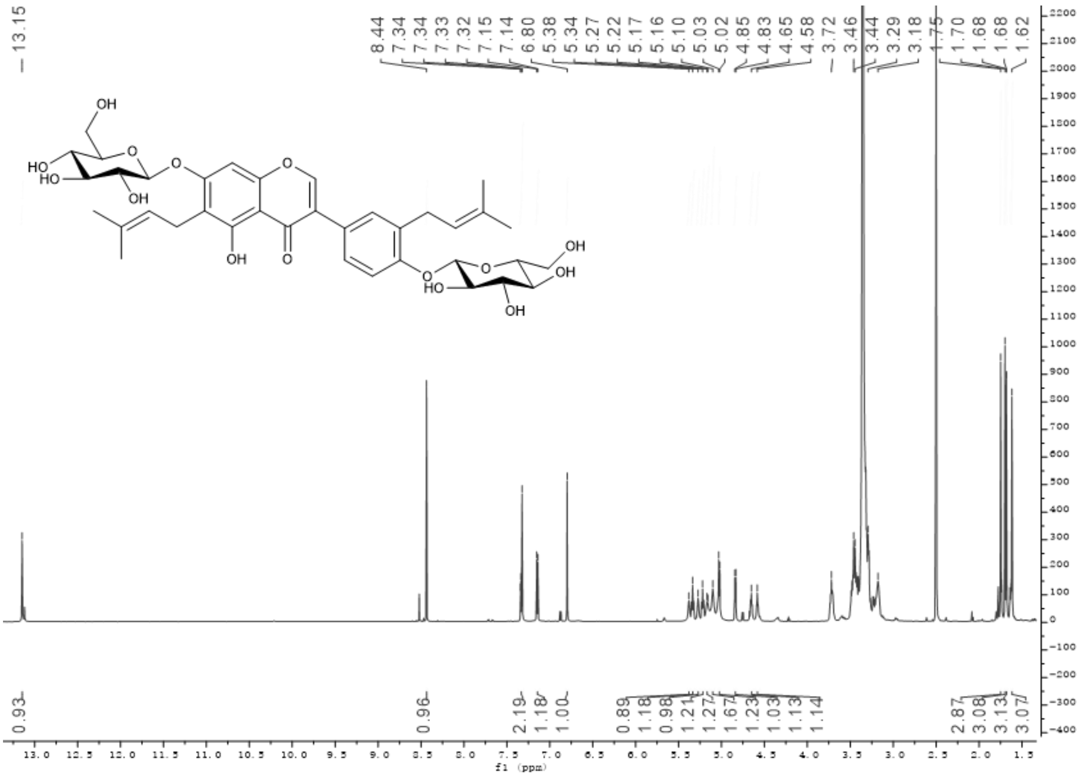

Supplementary Figure 95 ^{1}H NMR (600 MHz) spectrum of **10** in DMSO.

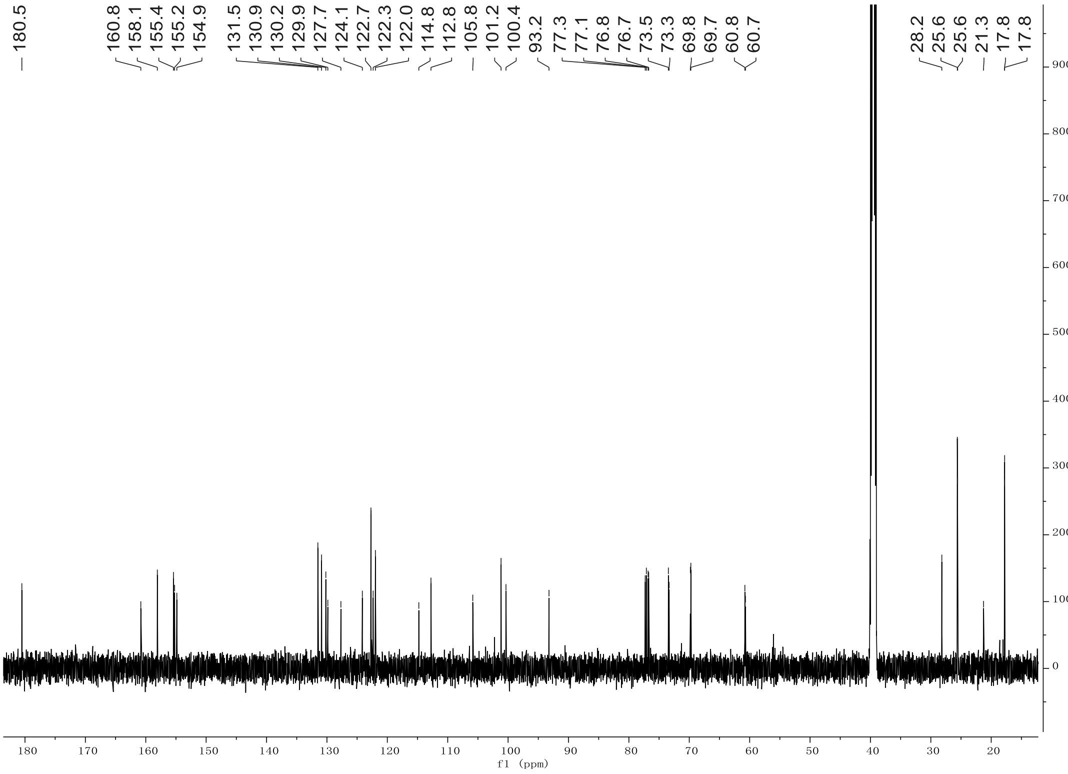

Supplementary Figure 96 ^{13}C NMR (150 MHz) spectrum of **10** in DMSO.

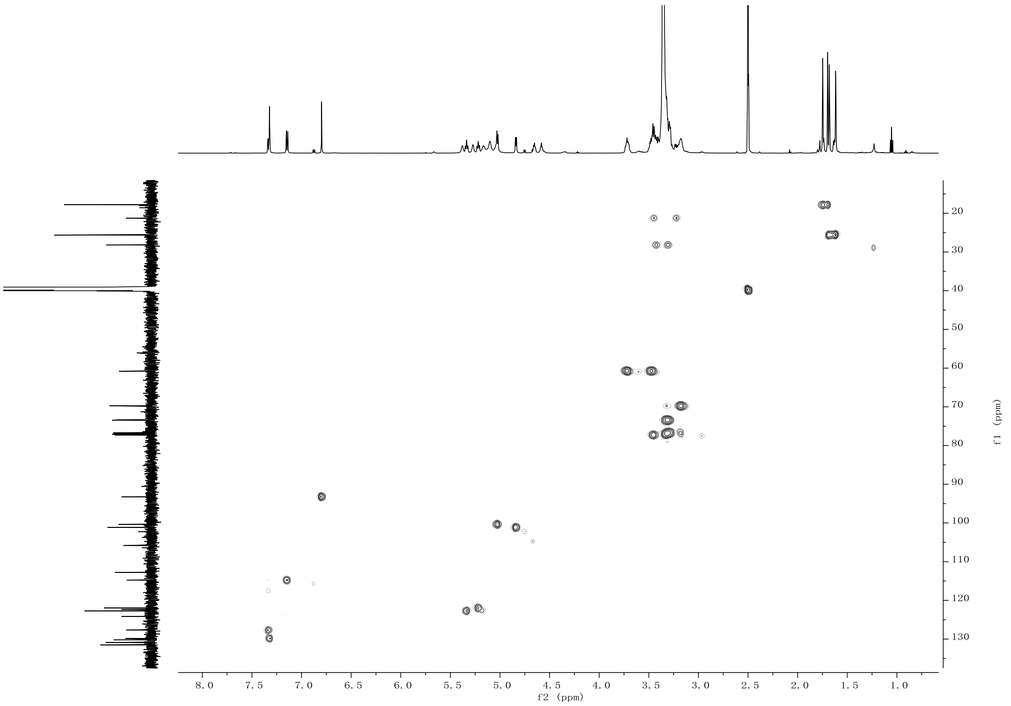

Supplementary Figure 97 HMQC (600 MHz) spectrum of **10** in DMSO.

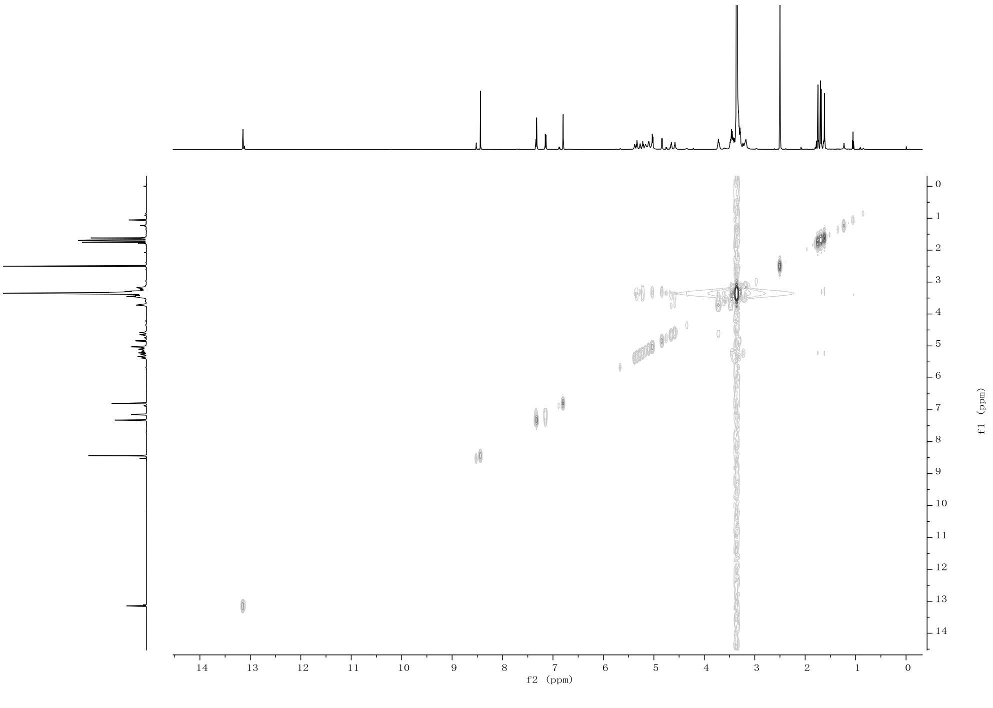

Supplementary Figure 98 ^{1}H-^{1}H COSY (600 MHz) spectrum of **10** in DMSO.

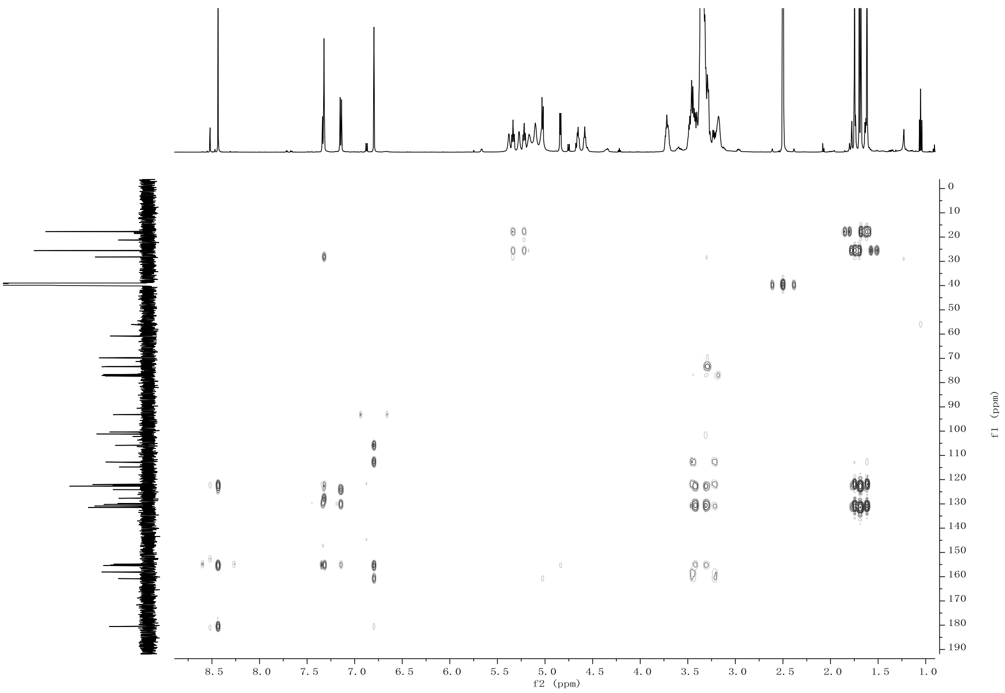

Supplementary Figure 99 HMBC (600 MHz) spectrum of **10** in DMSO.

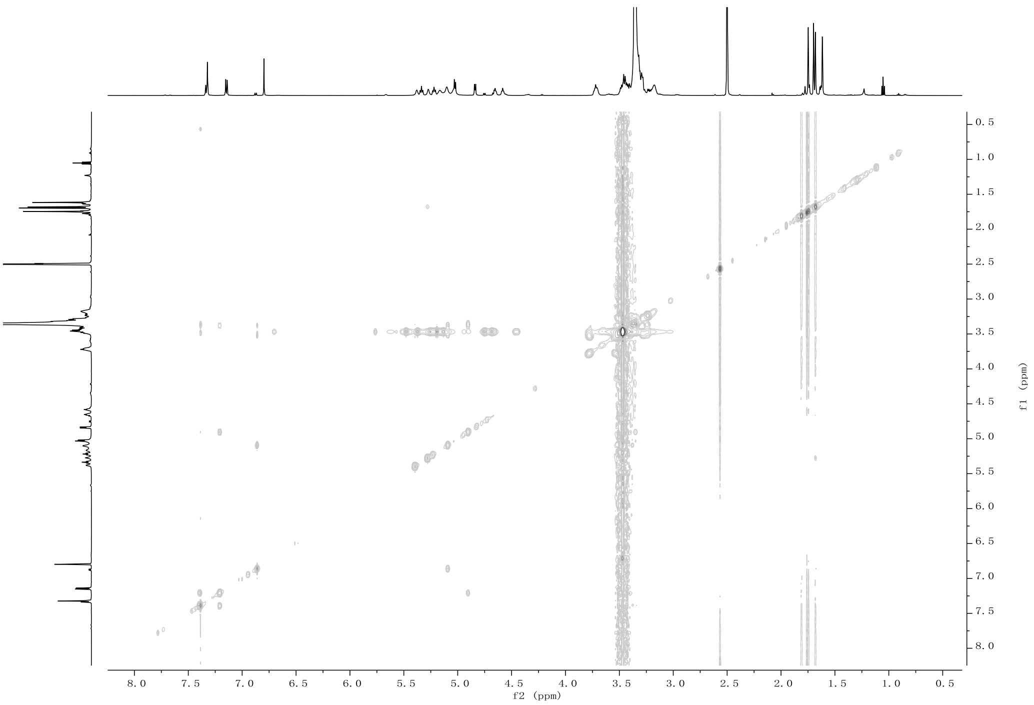

Supplementary Figure 100 ^{1}H-^{1}H NOESY (600 MHz) spectrum of **10** in DMSO.

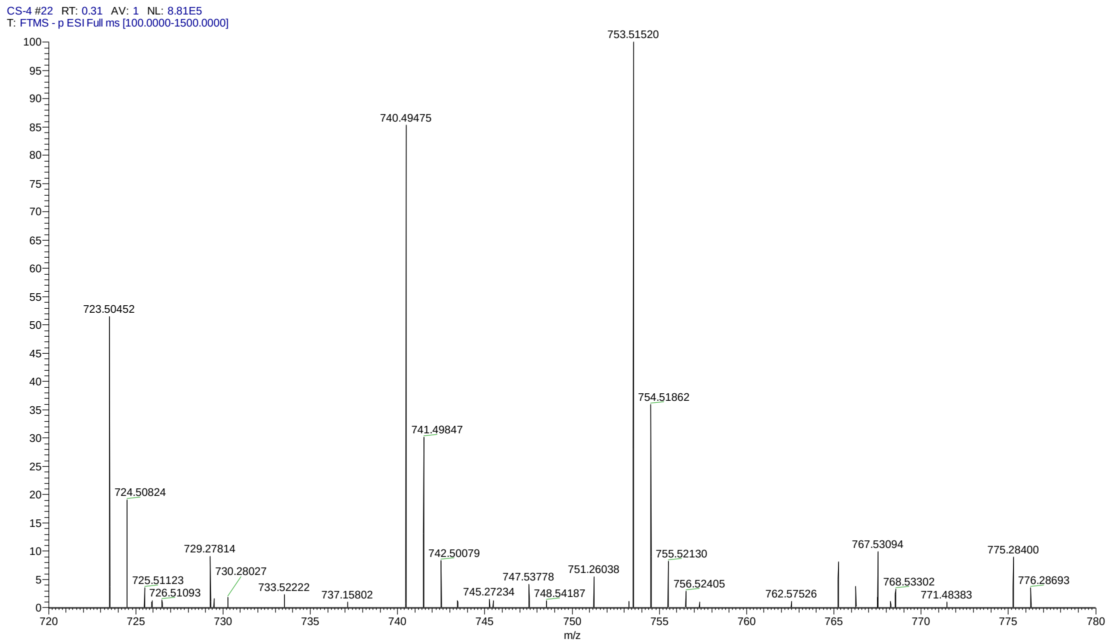

Supplementary Figure 101 HRESIMS spectrum of **10**.

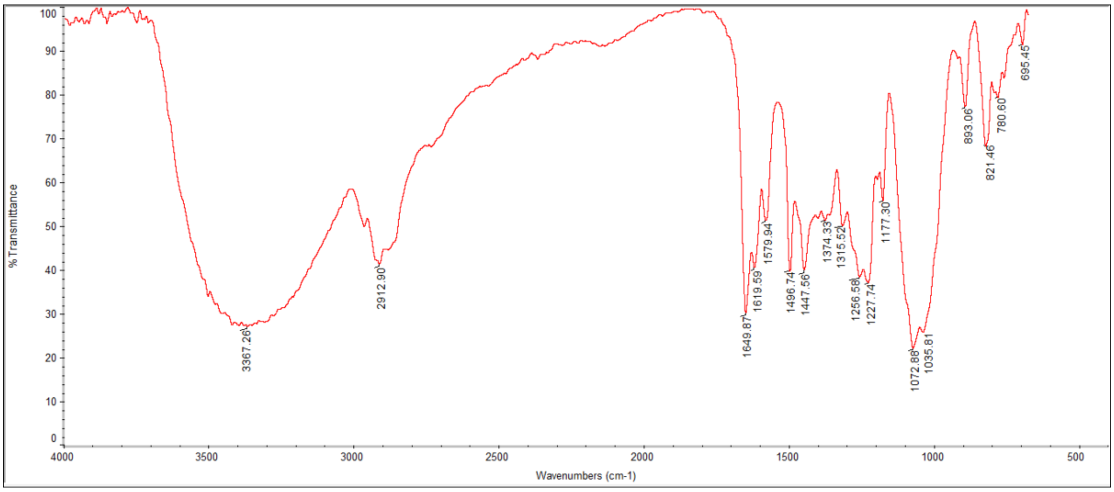

Supplementary Figure 102 IR (KBr disc) spectrum of **10**.

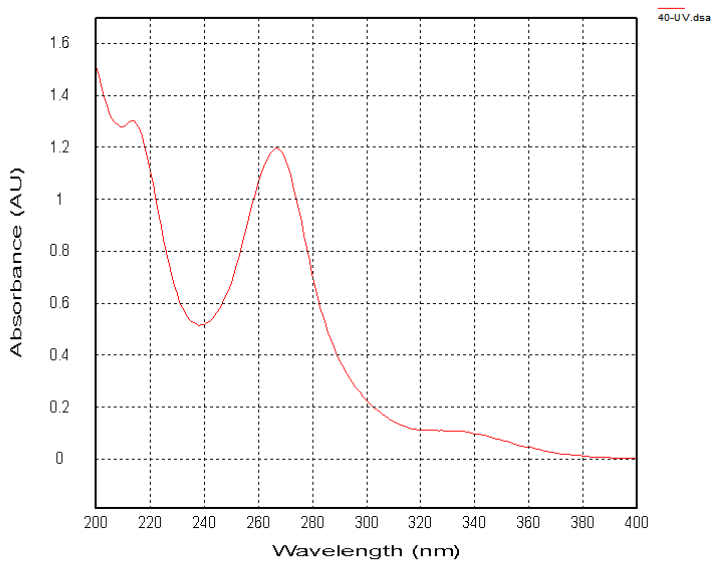

Supplementary Figure 103 UV spectrum of **10**.

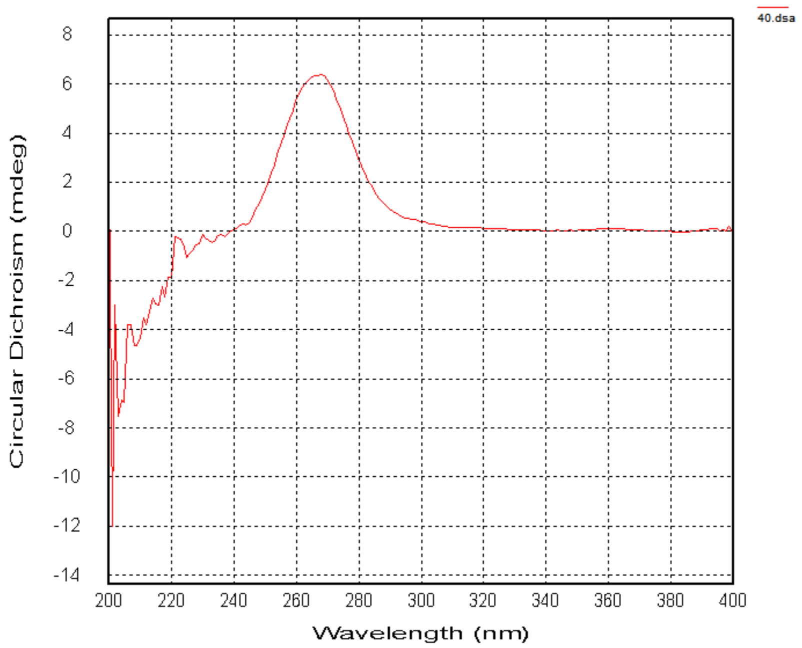

Supplementary Figure 104 ECD spectrum of **10**.

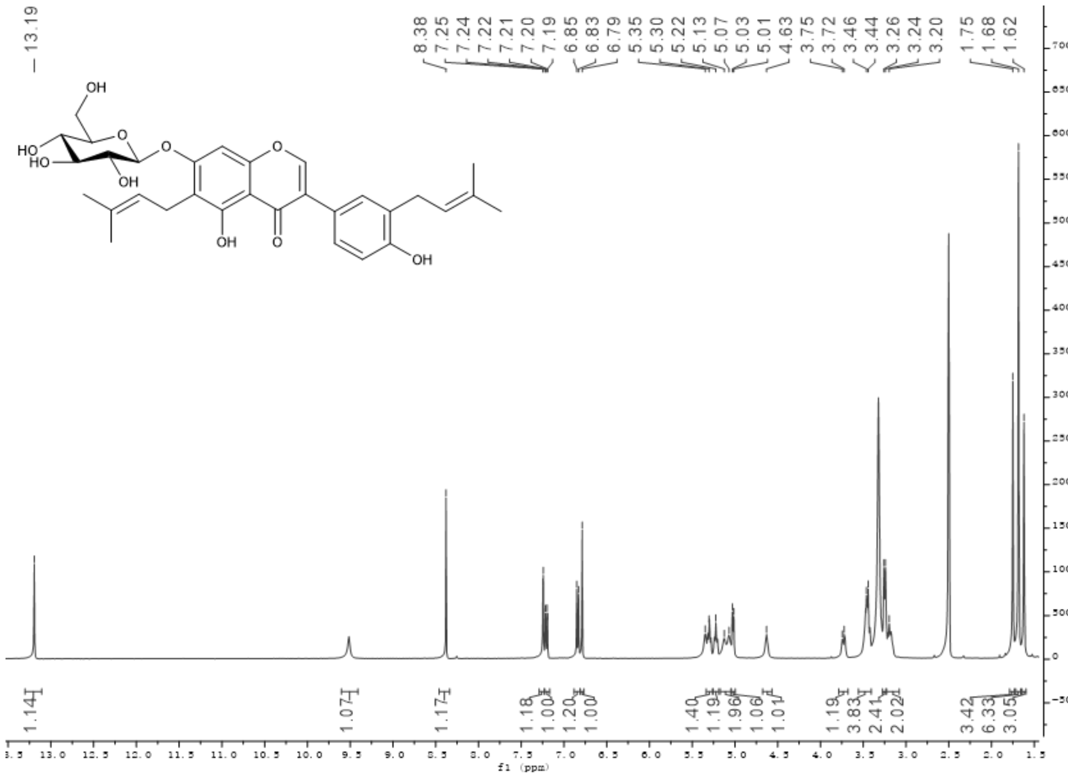

Supplementary Figure 105 ^{1}H NMR (400 MHz) spectrum of **11** in DMSO.

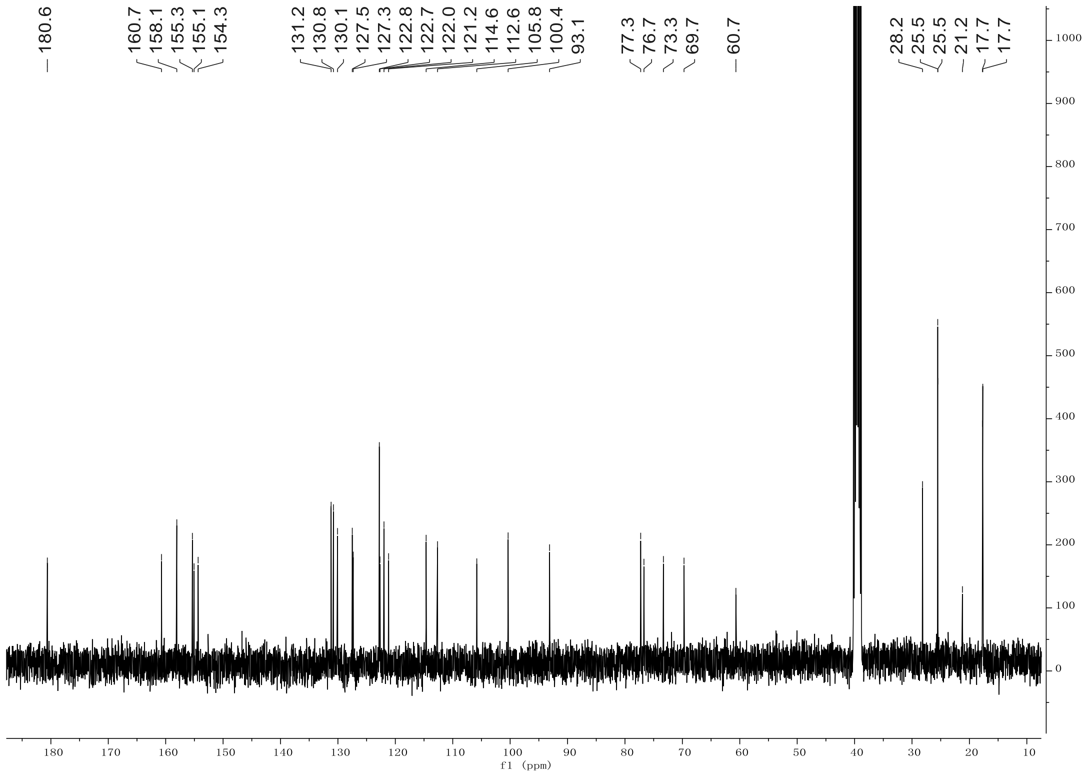

Supplementary Figure 106 ^{13}C NMR (100 MHz) spectrum of **11** in DMSO.

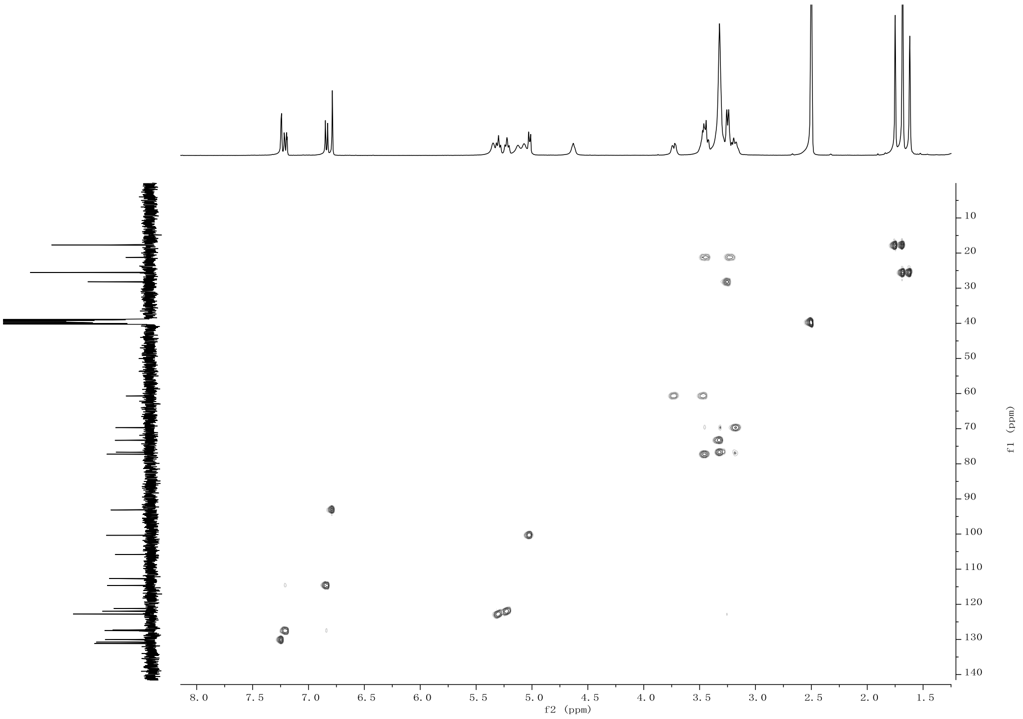

Supplementary Figure 107 HSQC (400 MHz) spectrum of **11** in DMSO.

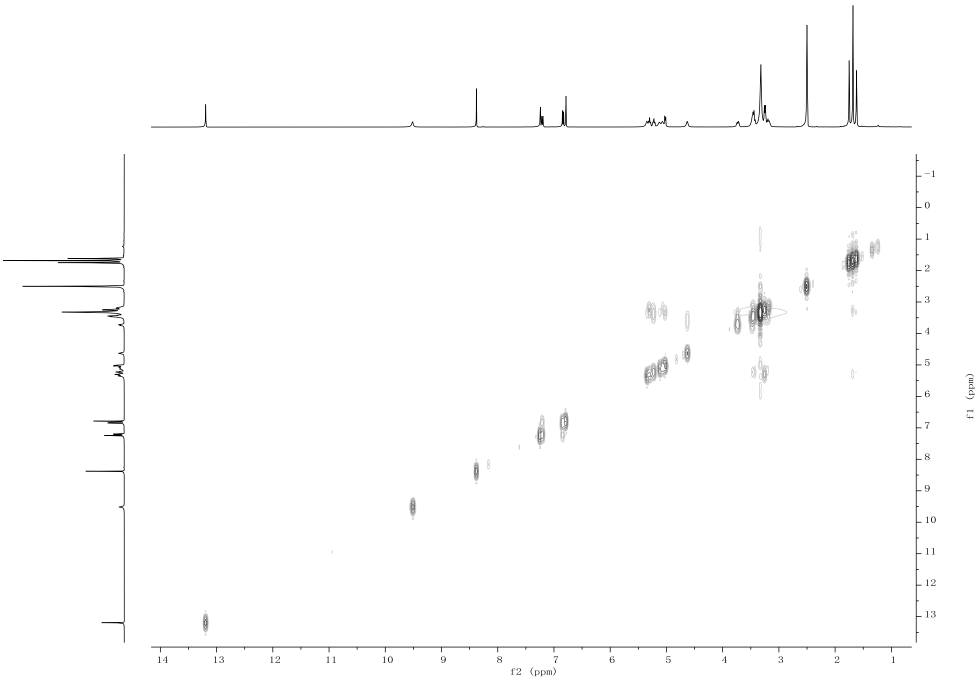

Supplementary Figure 108 ^{1}H-^{1}H COSY (600 MHz) spectrum of **11** in DMSO.

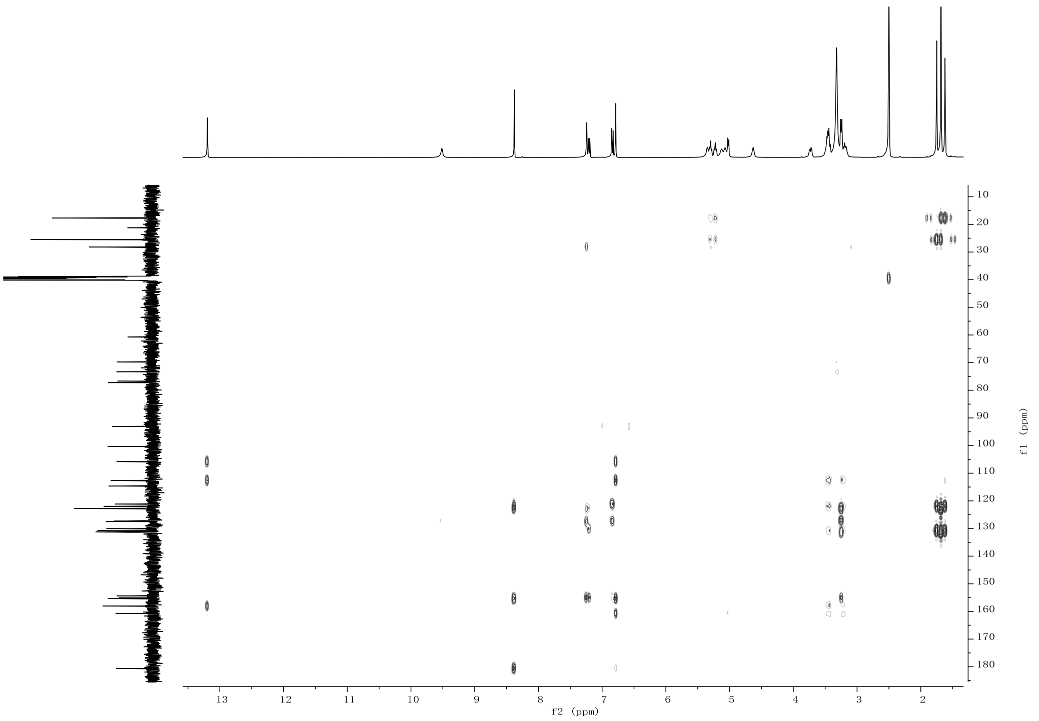

Supplementary Figure 109 HMBC (400 MHz) spectrum of **11** in DMSO.

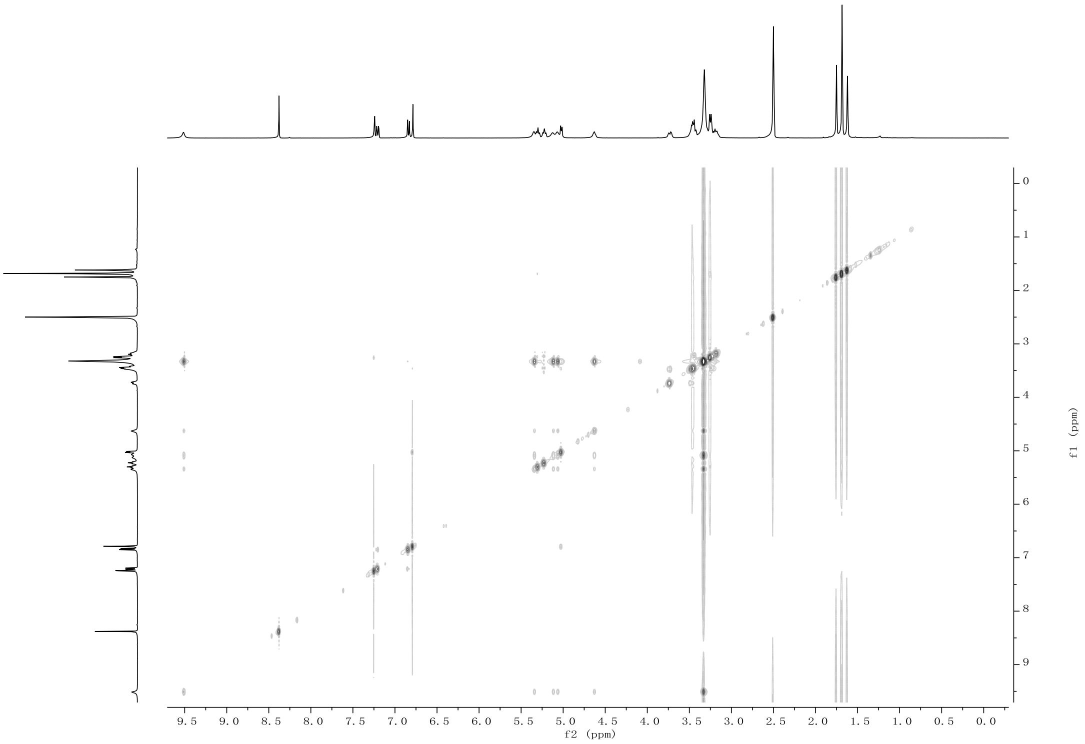

Supplementary Figure 110 ^{1}H-^{1}H NOESY (600 MHz) spectrum of **11** in DMSO.

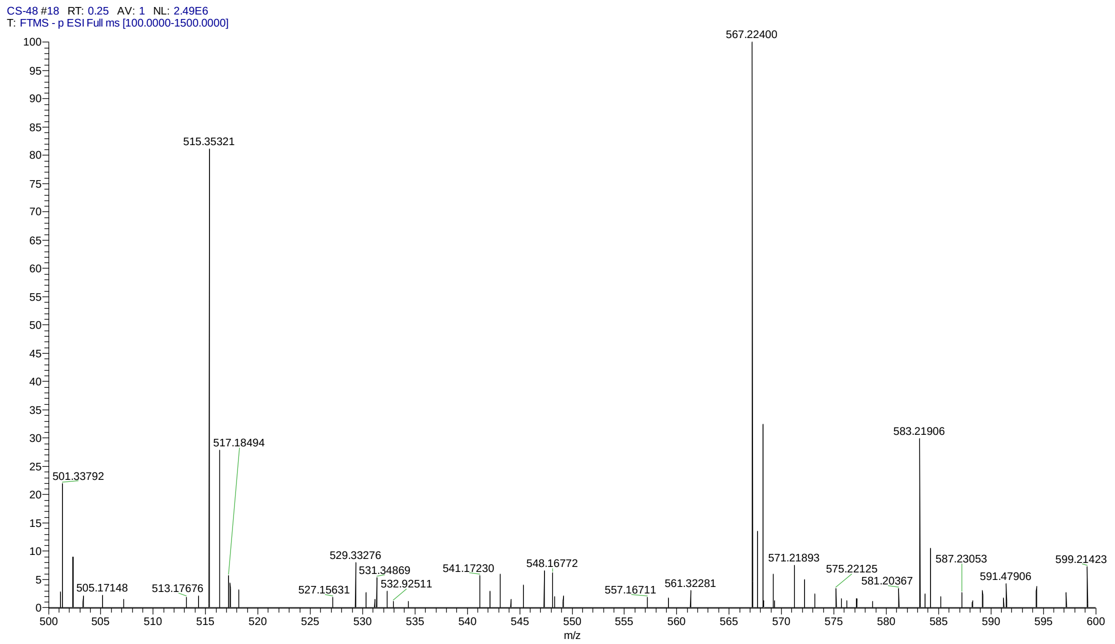

Supplementary Figure 111 HRESIMS spectrum of **11**.

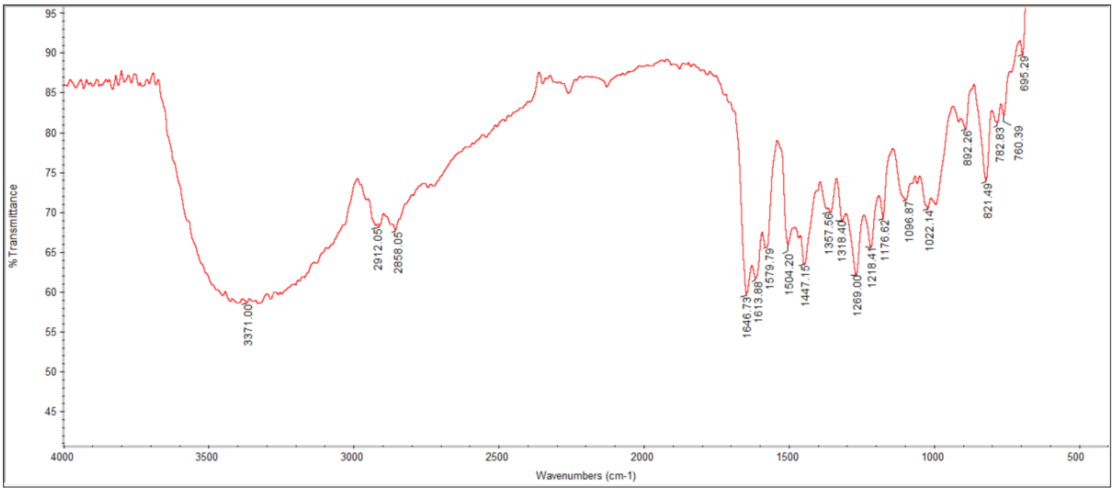

Supplementary Figure 112 IR (KBr disc) spectrum of **11**.

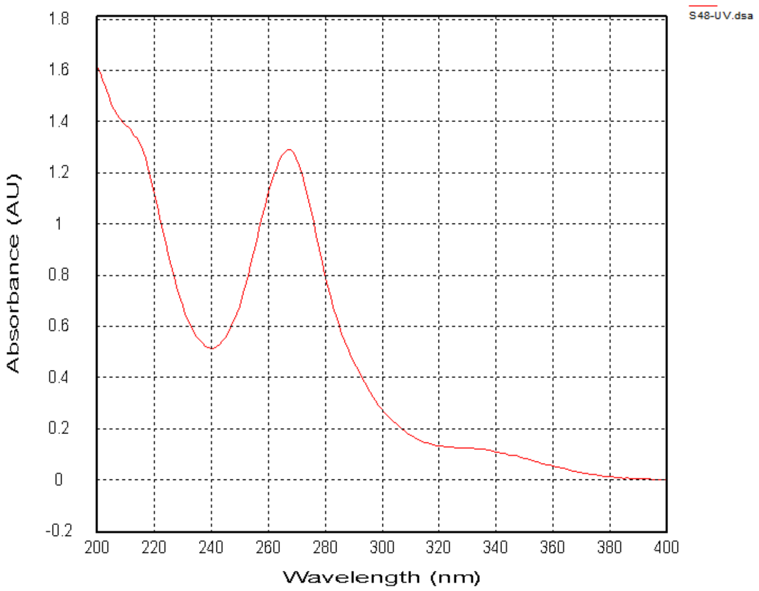

Supplementary Figure 113 UV spectrum of **11**.

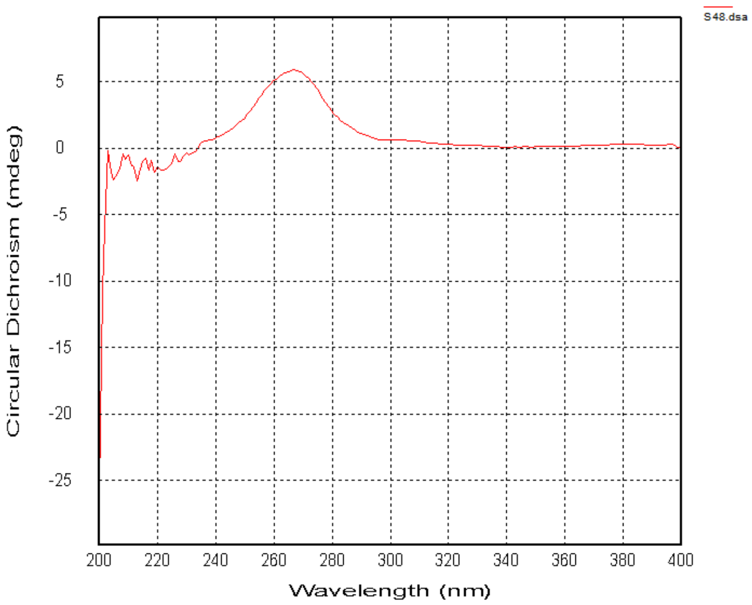

Supplementary Figure 114 ECD spectrum of **11**.

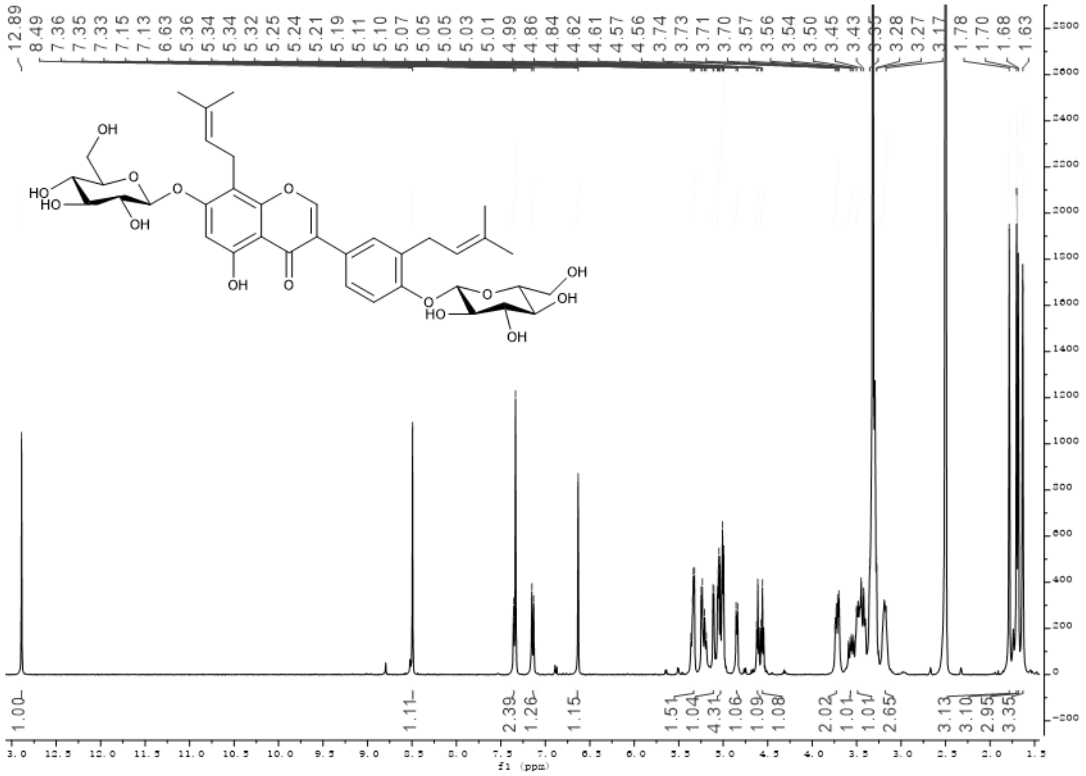

Supplementary Figure 115 ^{1}H NMR (600 MHz) spectrum of **12** in DMSO.

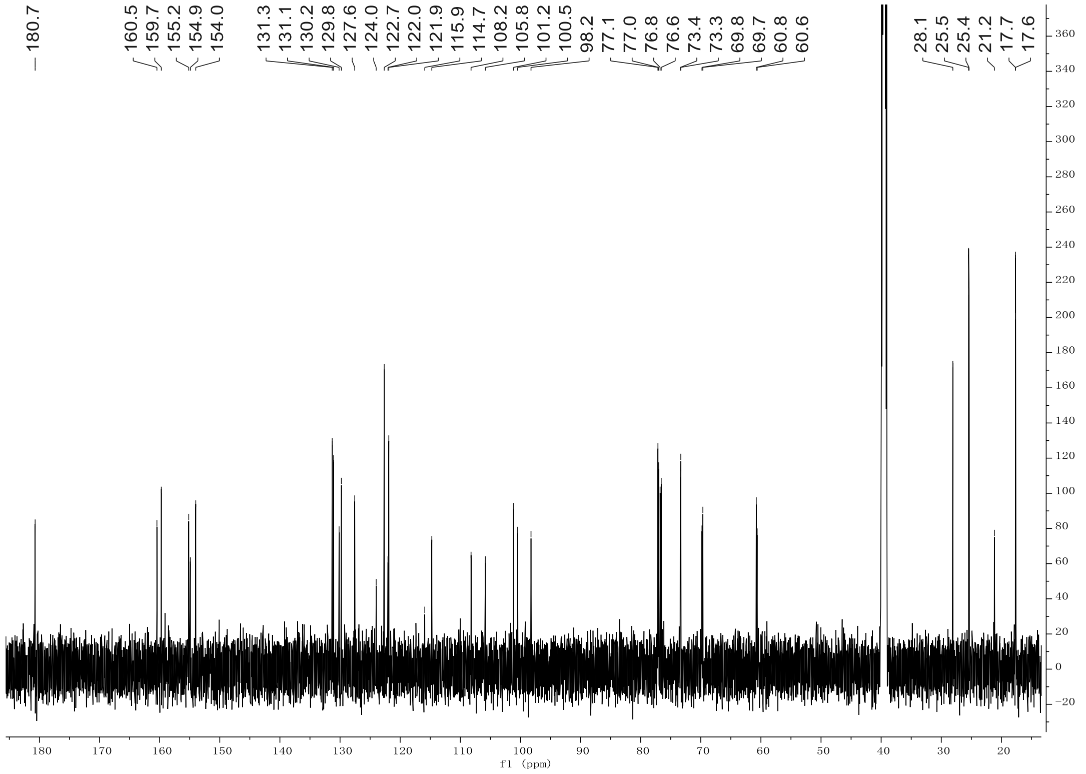

Supplementary Figure 116 ^{13}C NMR (150 MHz) spectrum of **12** in DMSO.

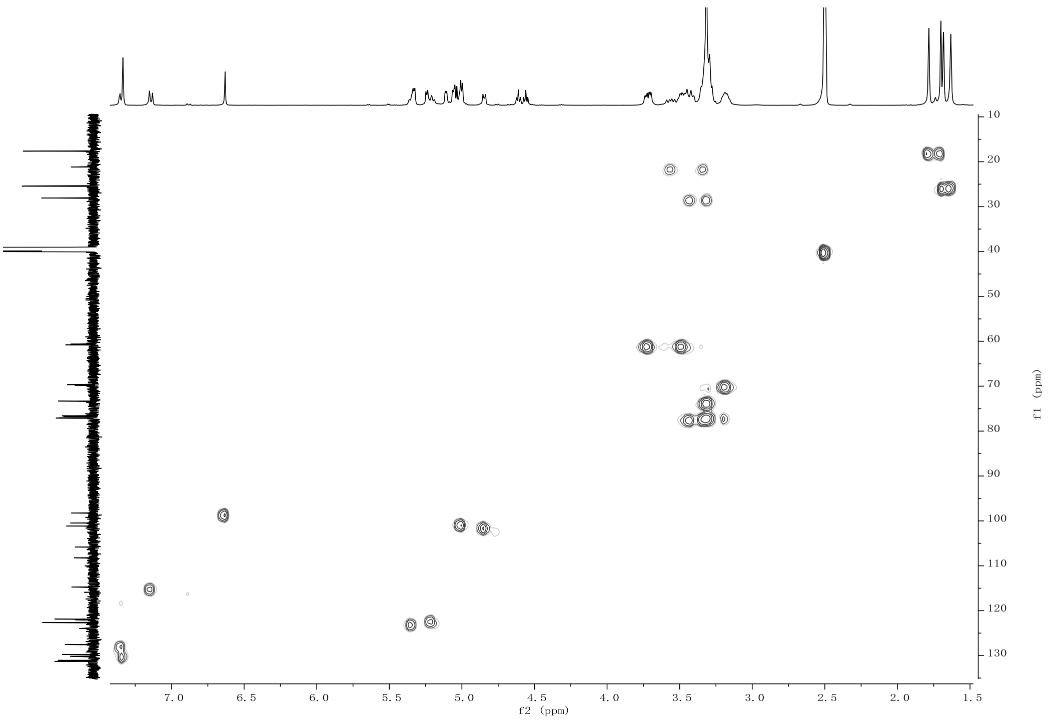

Supplementary Figure 117 HMQC spectrum of **12** in DMSO.

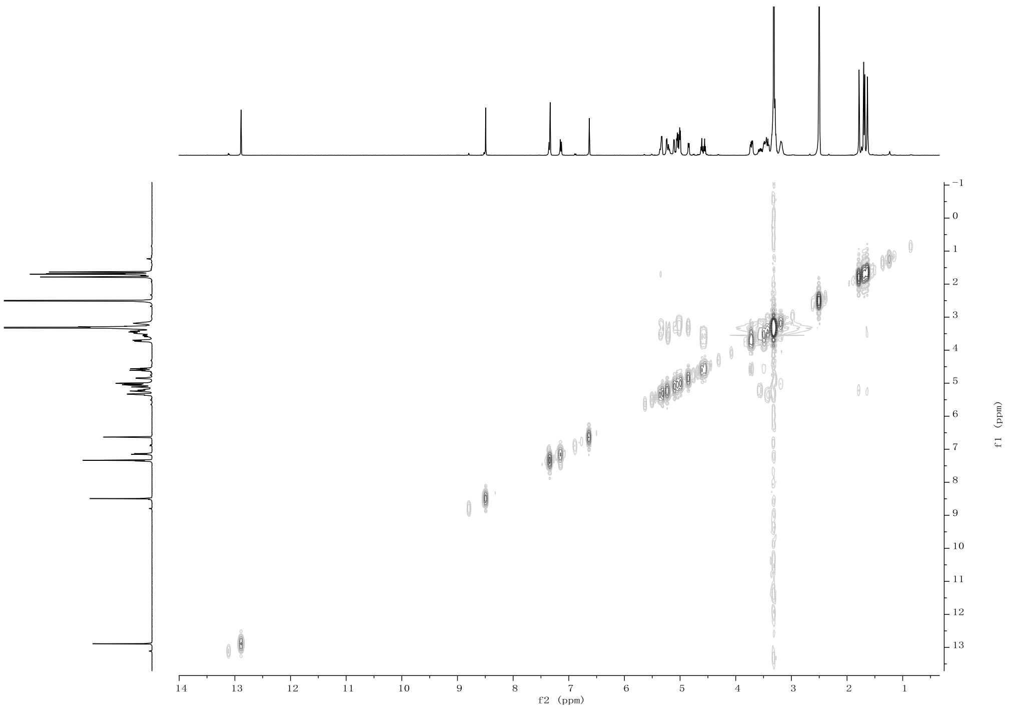

Supplementary Figure 118 ^{1}H-^{1}H COSY (600 MHz) spectrum of **12** in DMSO.

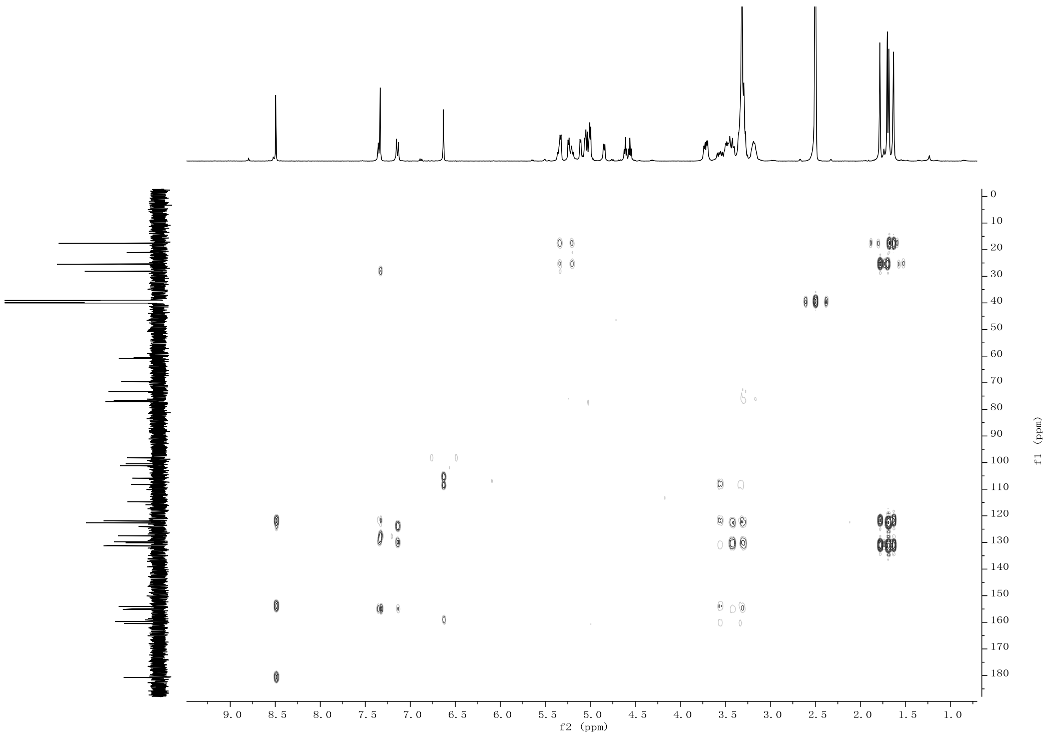

Supplementary Figure 119 HMBC (600 MHz) spectrum of **12** in DMSO.

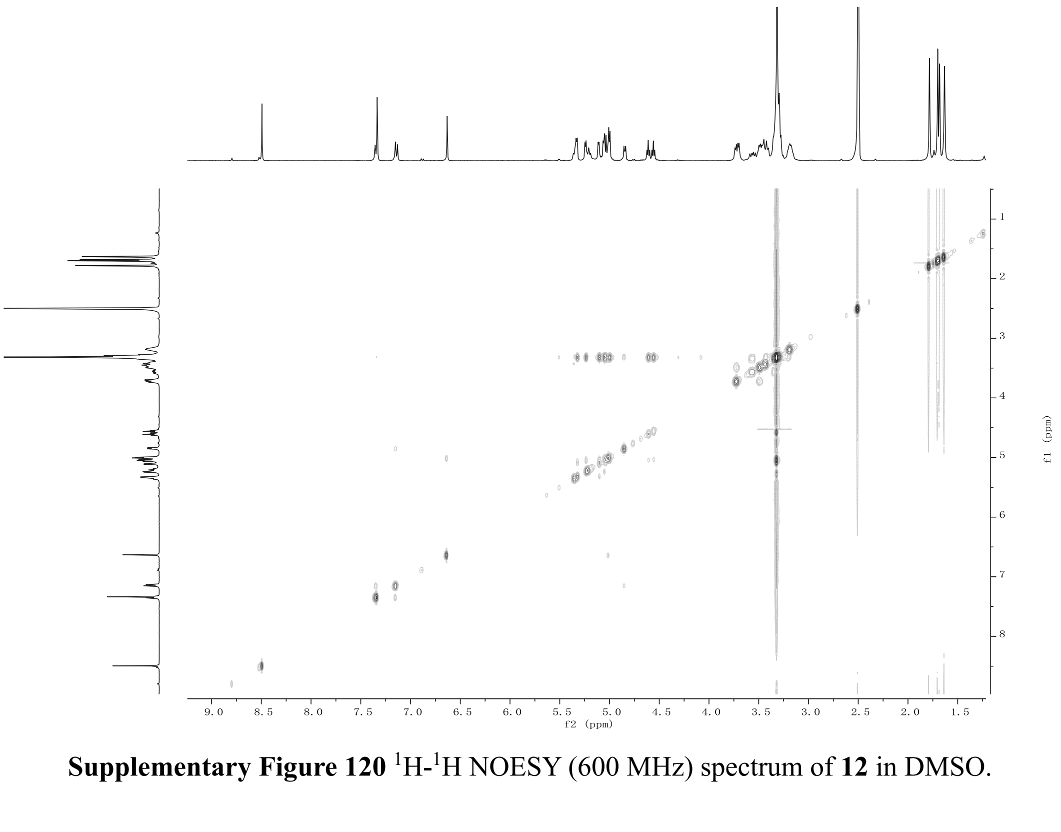

Supplementary Figure 120 ^{1}H-^{1}H NOESY (600 MHz) spectrum of **12** in DMSO.

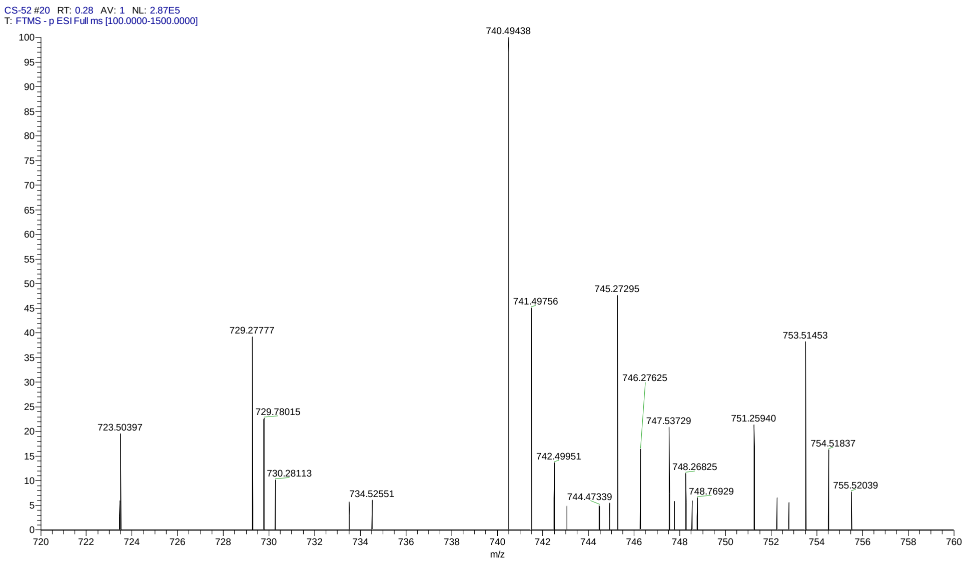

Supplementary Figure 121 HRESIMS spectrum of **12**.

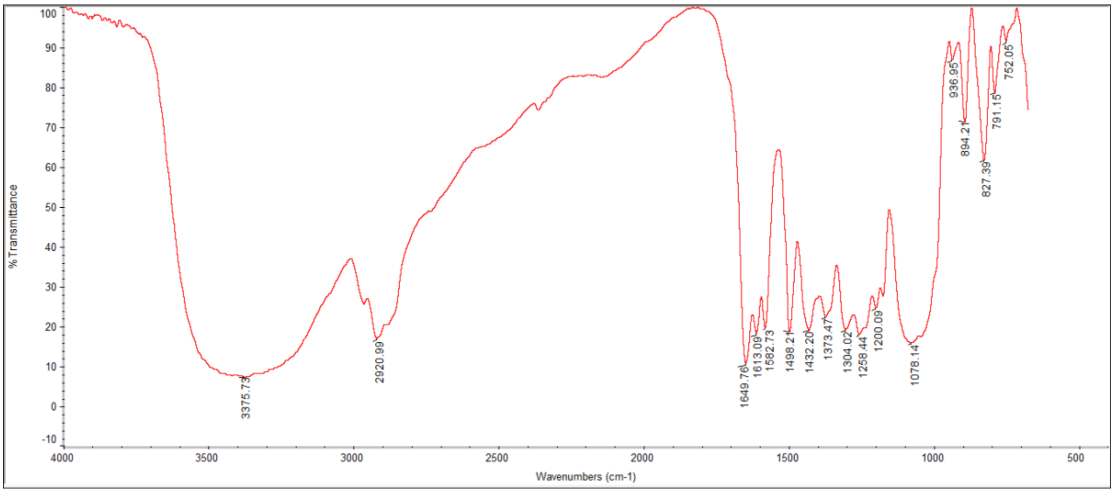

Supplementary Figure 122 IR (KBr disc) spectrum of **12**.

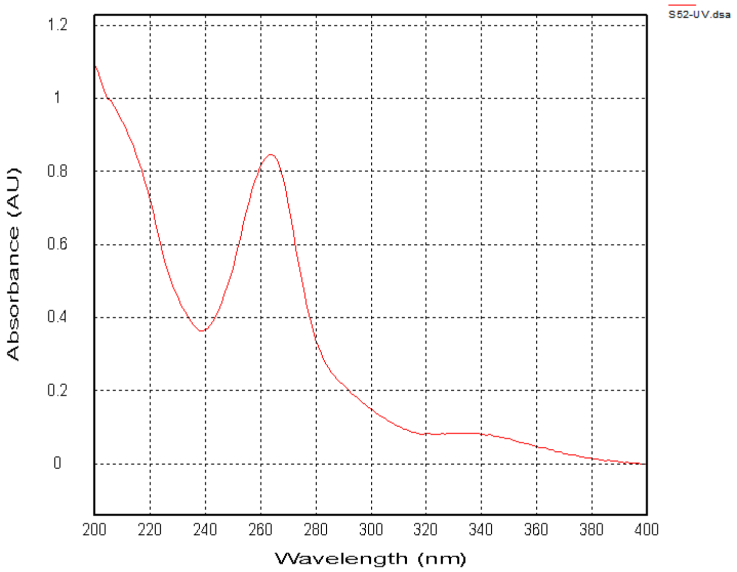

Supplementary Figure 123 UV spectrum of **12**.

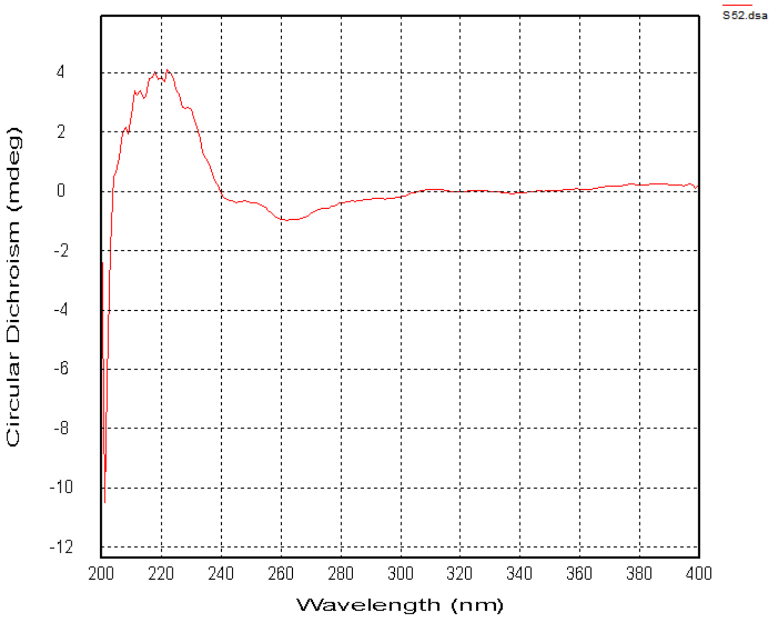

Supplementary Figure 124 ECD spectrum of **12.**

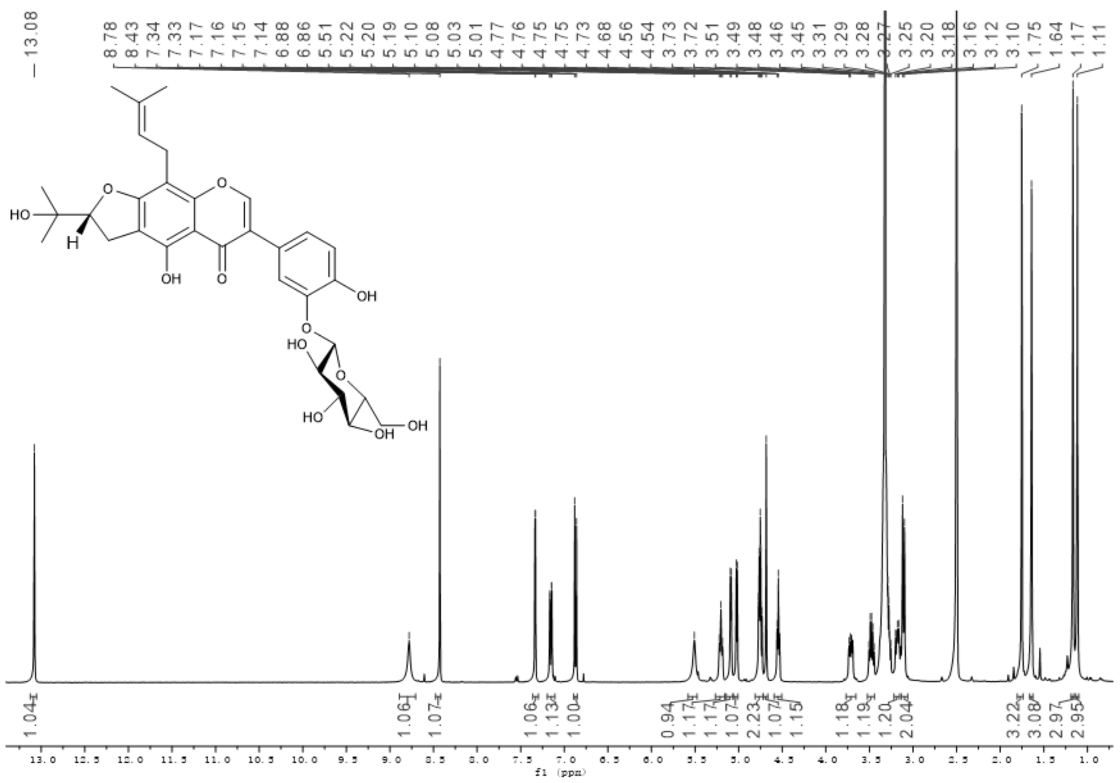

Supplementary Figure 125 ^{1}H NMR (400 MHz) spectrum of **13** in DMSO.

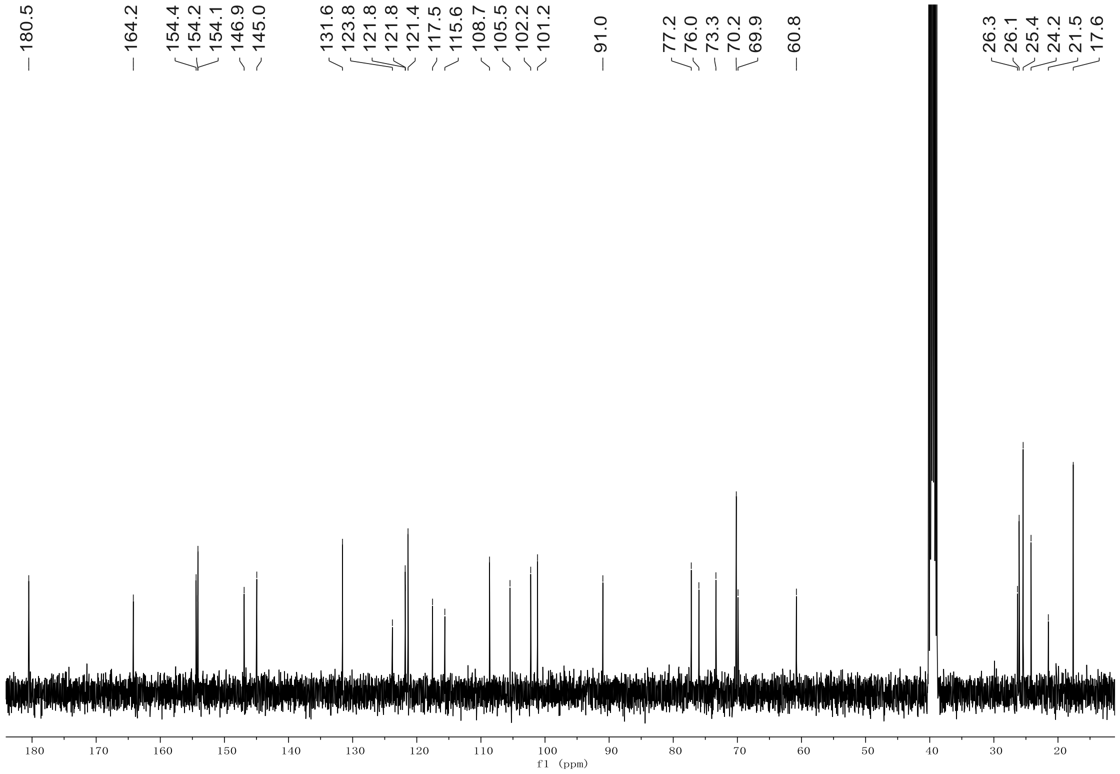

Supplementary Figure 126 ^{13}C NMR (100 MHz) spectrum of **13** in DMSO.

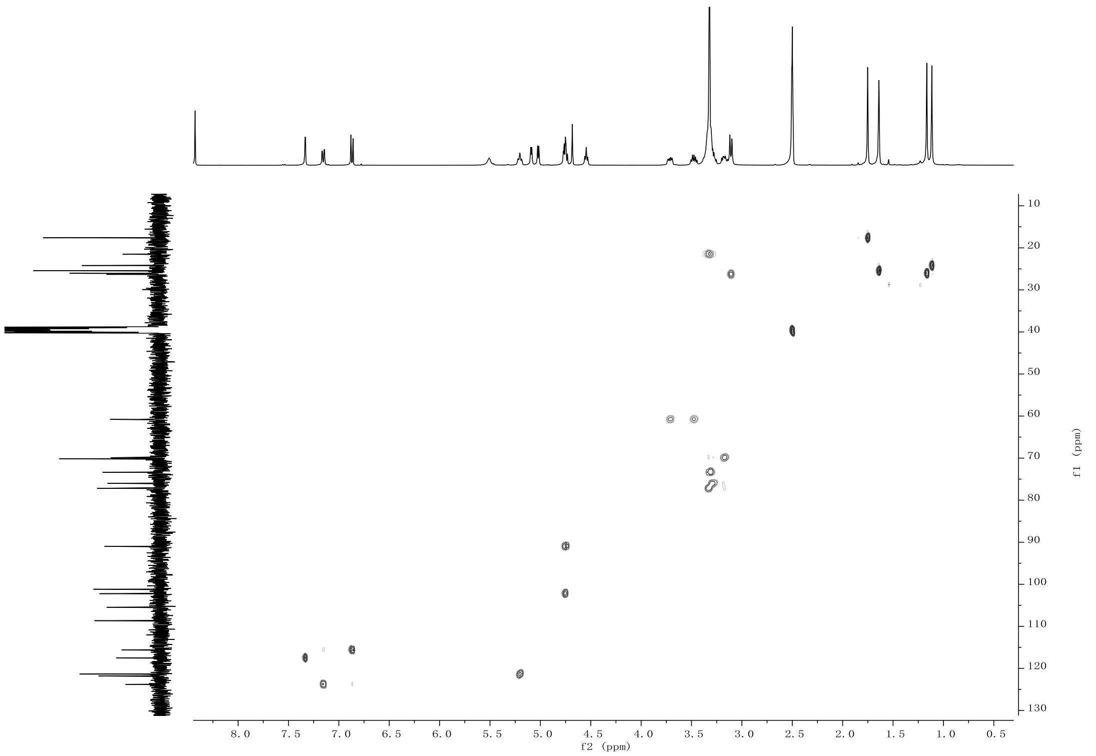

Supplementary Figure 127 HSQC (400 MHz) spectrum of **13** in DMSO.

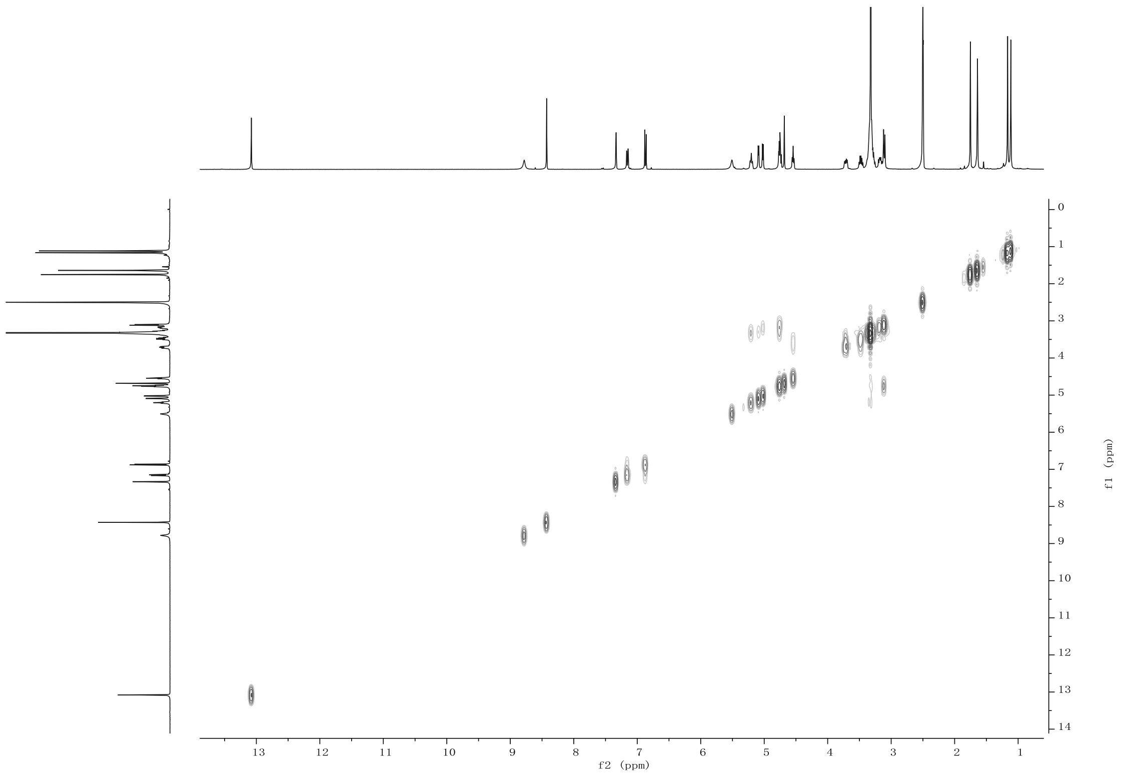

Supplementary Figure 128 ^{1}H-^{1}H COSY (600 MHz) spectrum of **13** in DMSO.

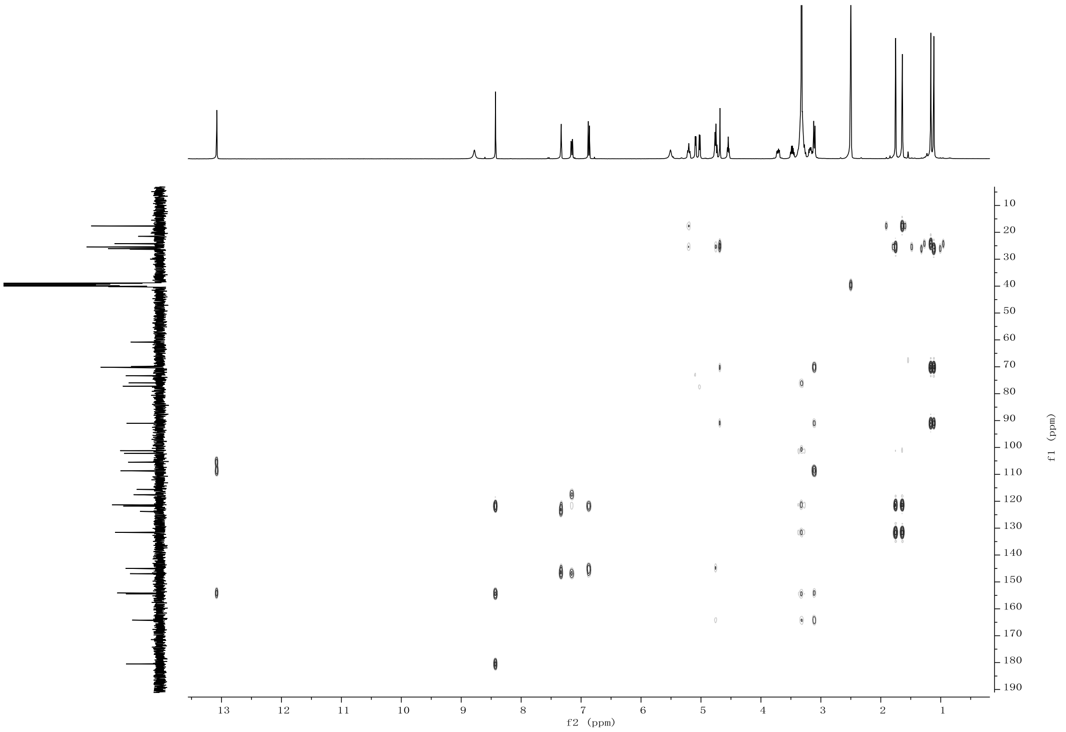

Supplementary Figure 129 HMBC (400 MHz) spectrum of **13** in DMSO.

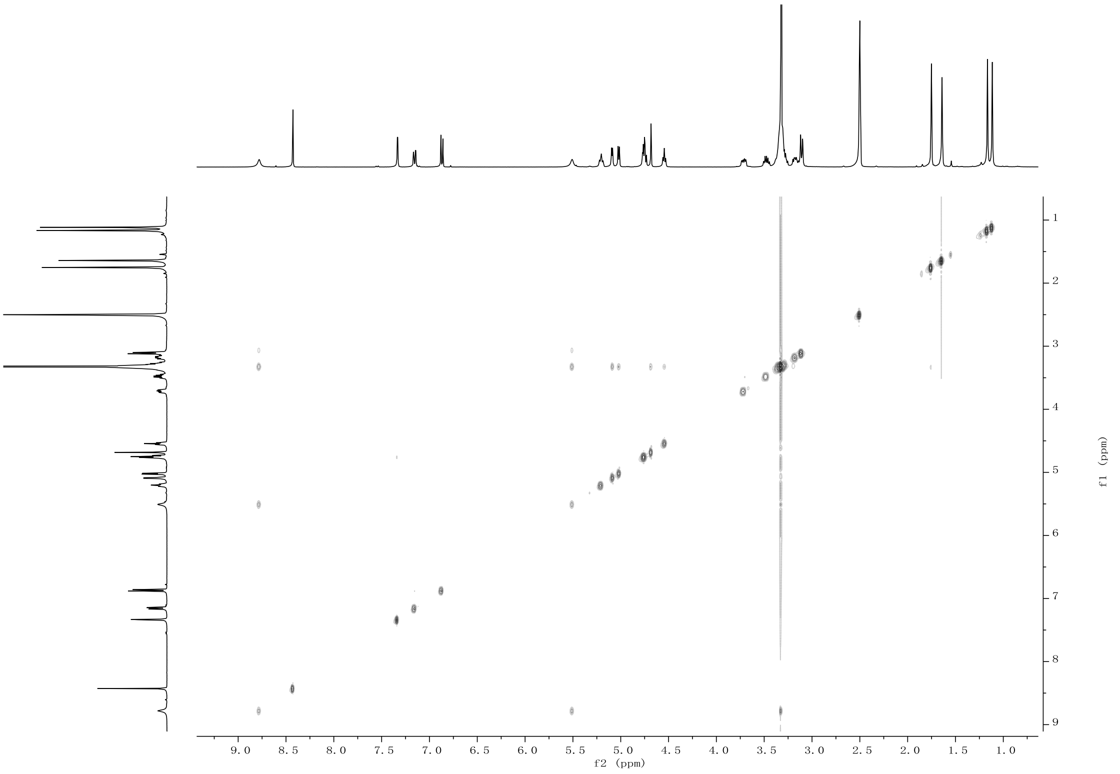

Supplementary Figure 130 ^{1}H-^{1}H NOESY (600 MHz) spectrum of **13** in DMSO.

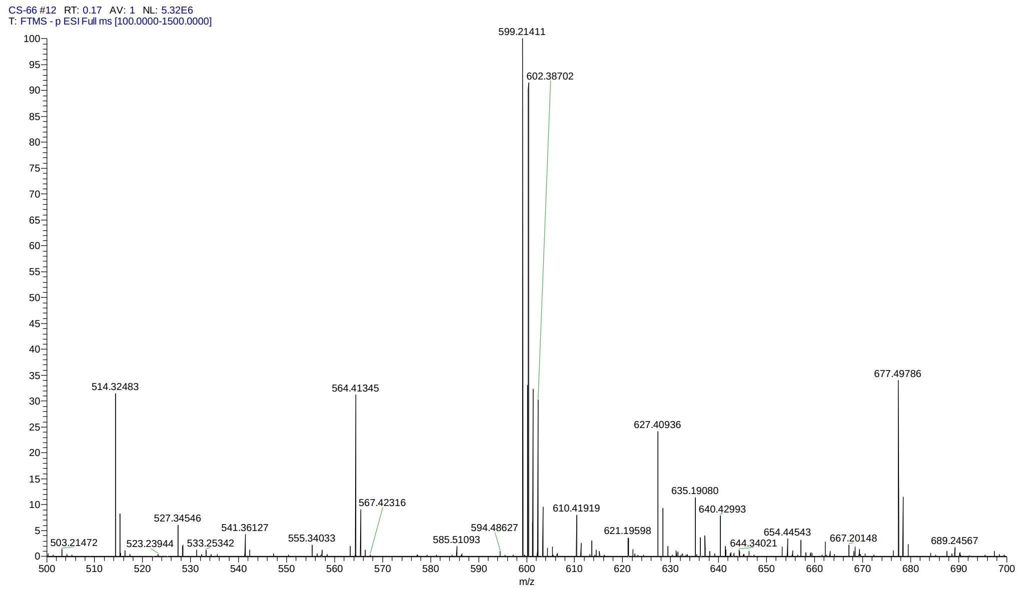

Supplementary Figure 131 HRESIMS spectrum of **13**.

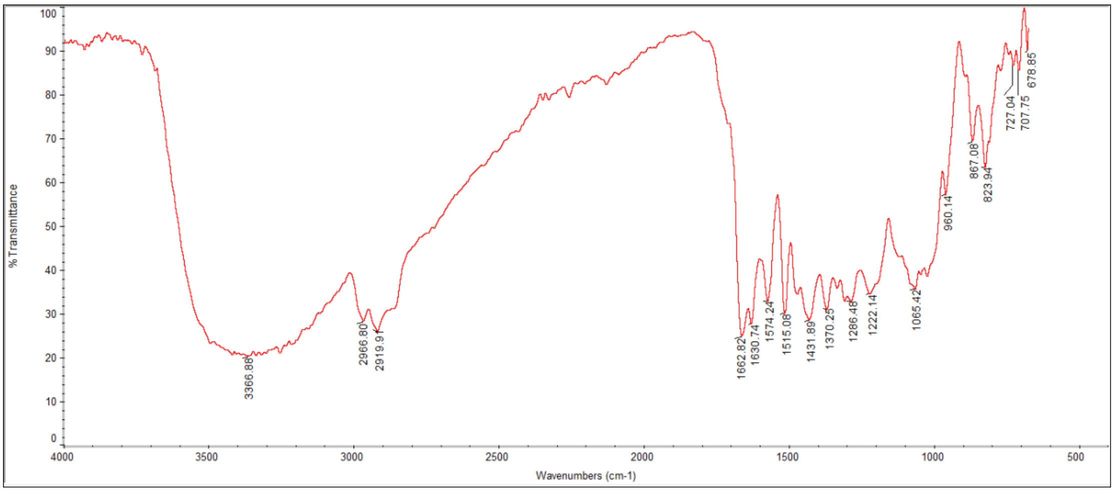

Supplementary Figure 132 IR (KBr disc) spectrum of **13**.

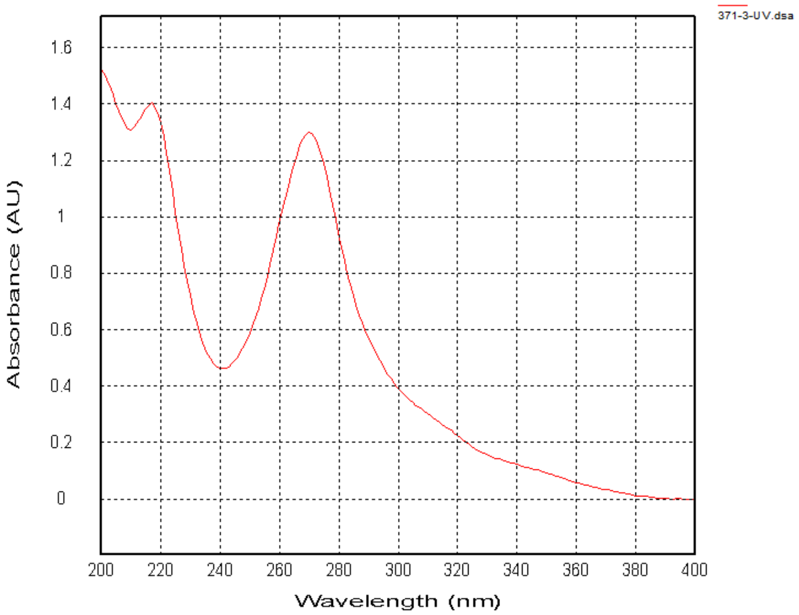

Supplementary Figure 133 UV spectrum of **13**.

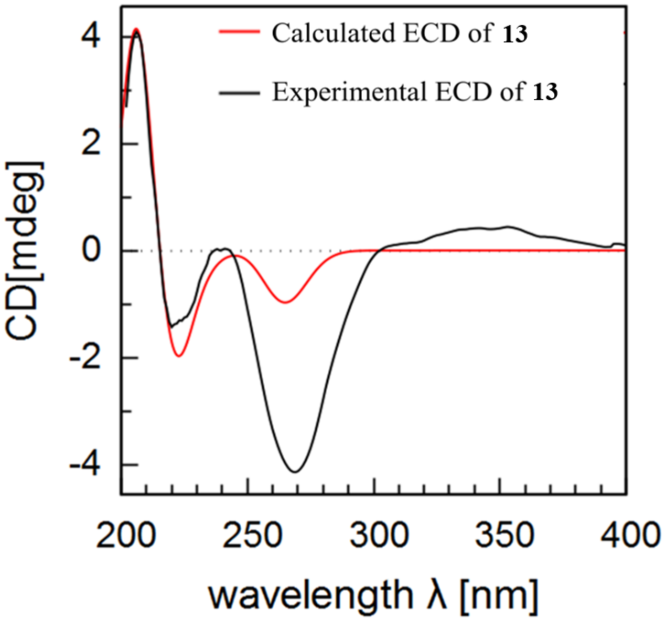

Supplementary Figure 134 Experimental ECD spectra and calculated ECD spectrum of **13**.

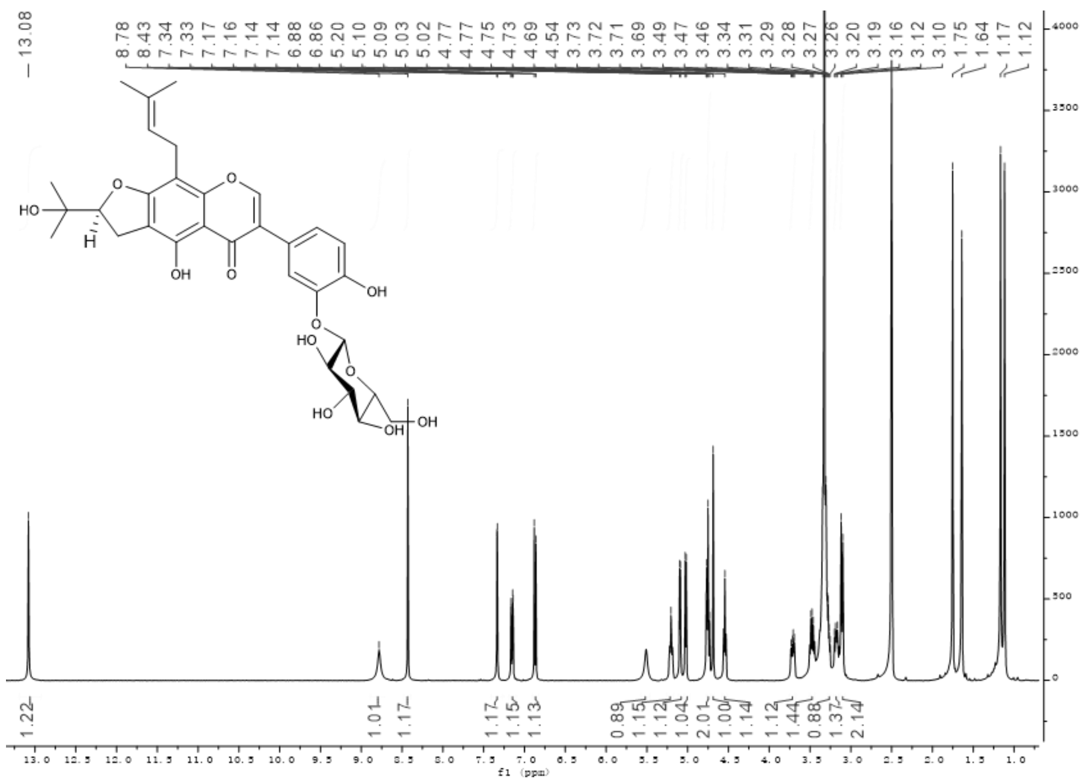

Supplementary Figure 135 ^{1}H NMR (600 MHz) spectrum of **14** in DMSO.

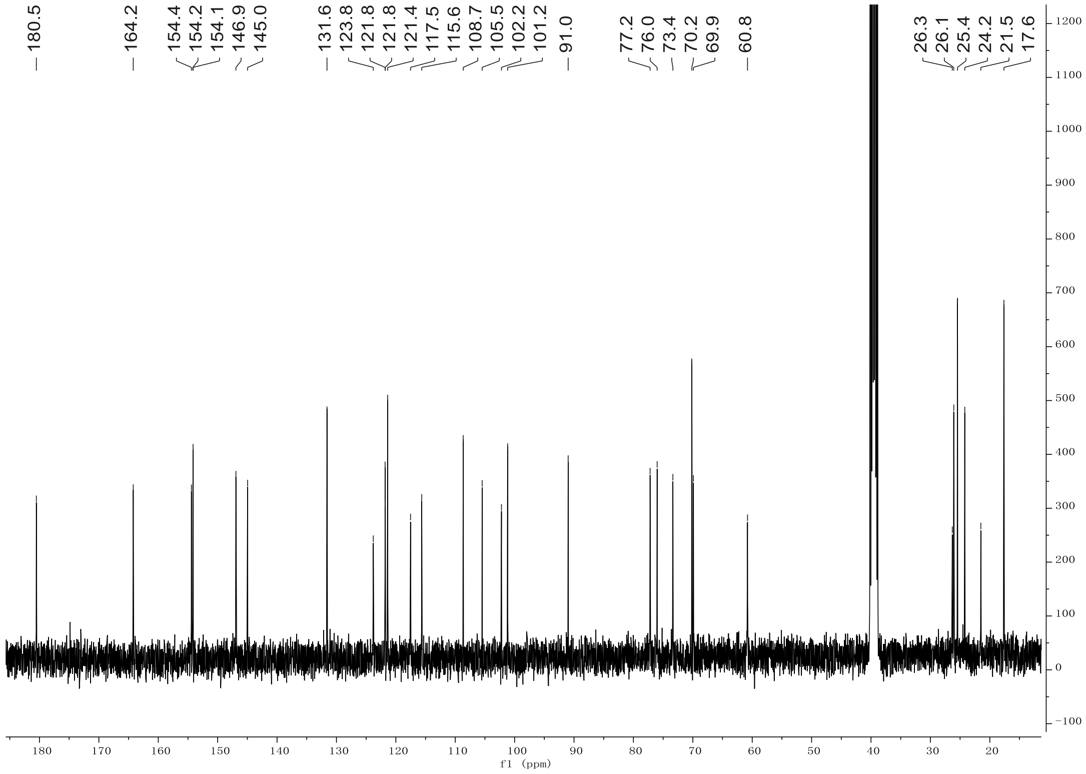

Supplementary Figure 136 ^{13}C NMR (150 MHz) spectrum of **14** in DMSO.

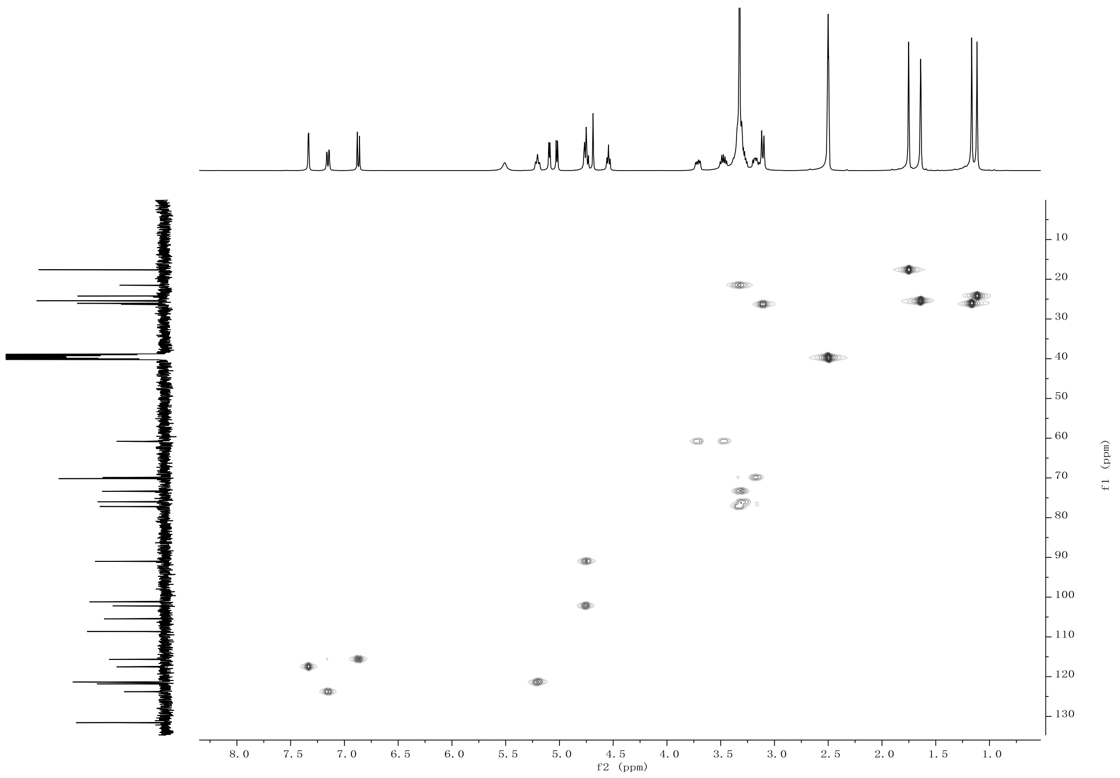

Supplementary Figure 137 HMQC (600 MHz) spectrum of **14** in DMSO.

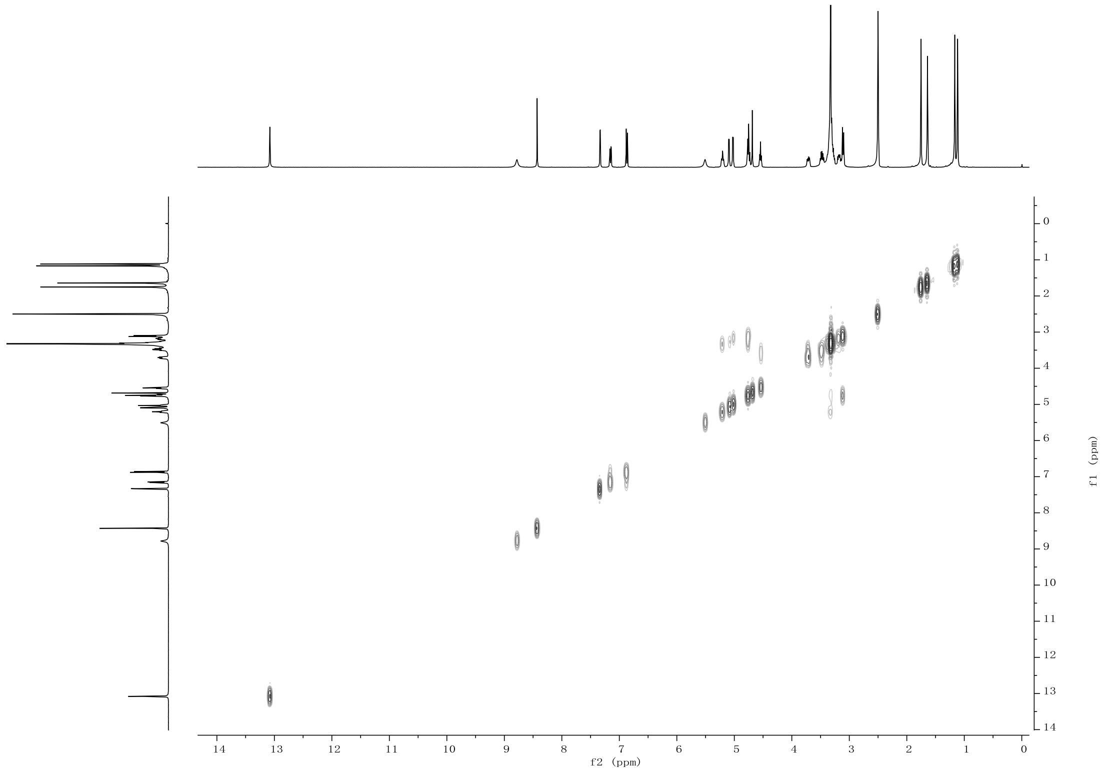

Supplementary Figure 138 ^{1}H-^{1}H COSY (600 MHz) spectrum of **14** in DMSO.

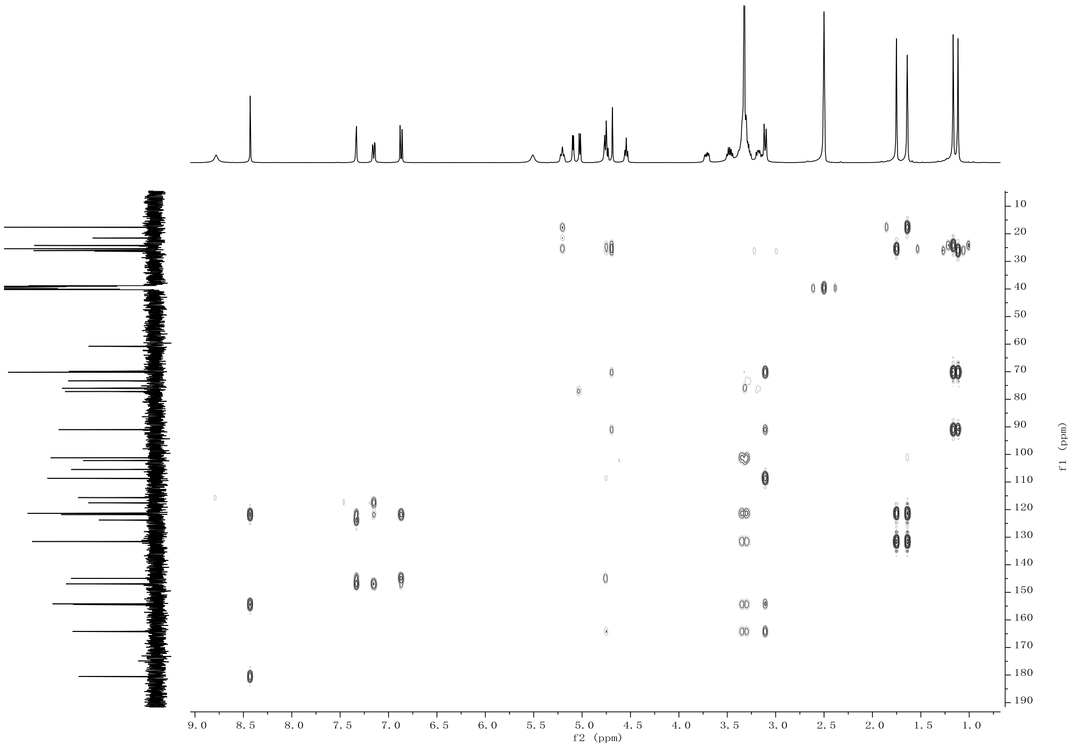

Supplementary Figure 139 HMBC (600 MHz) spectrum of **14** in DMSO.

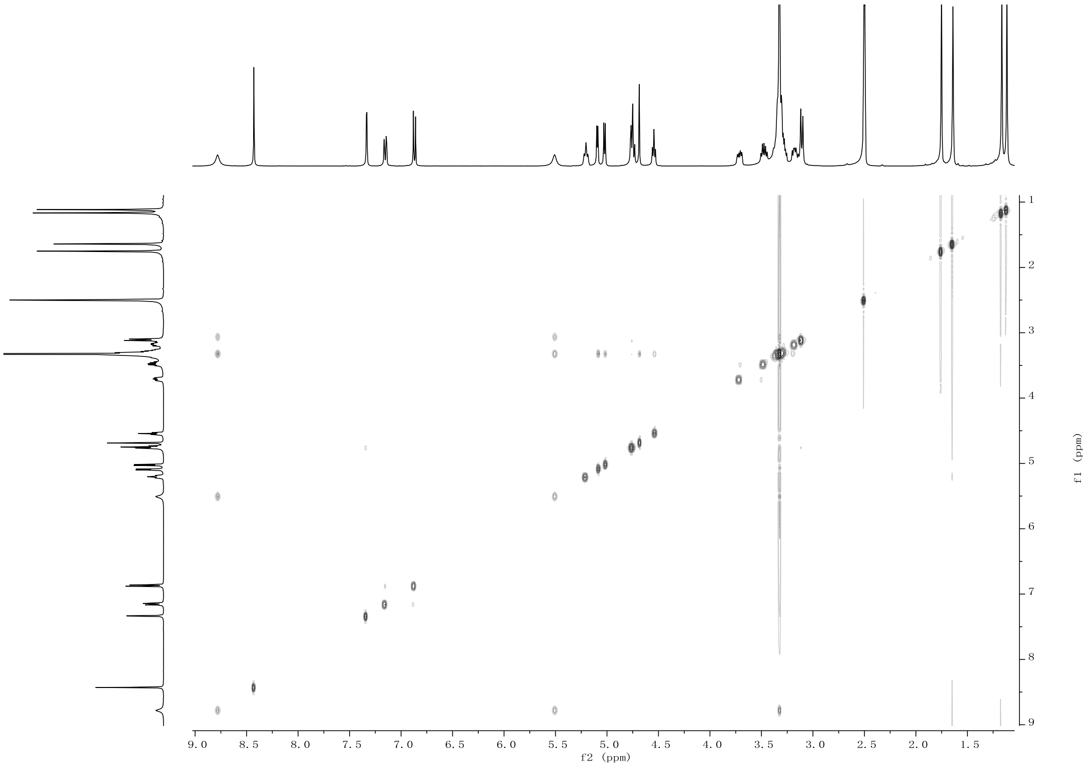

Supplementary Figure 140 ^{1}H-^{1}H NOESY (600 MHz) spectrum of **14** in DMSO.

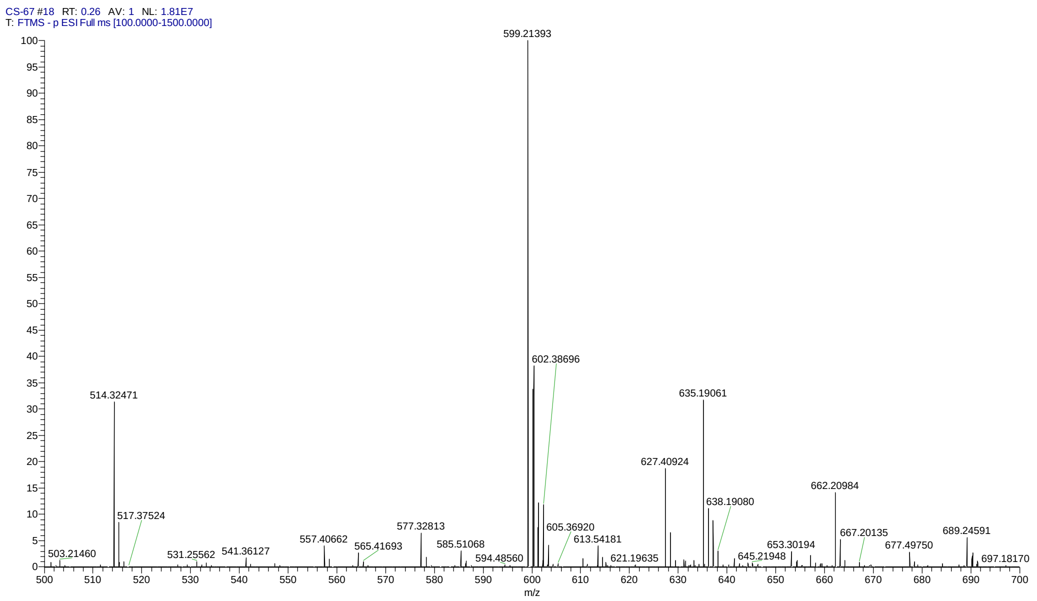

Supplementary Figure 141 HRESIMS spectrum of **14**.

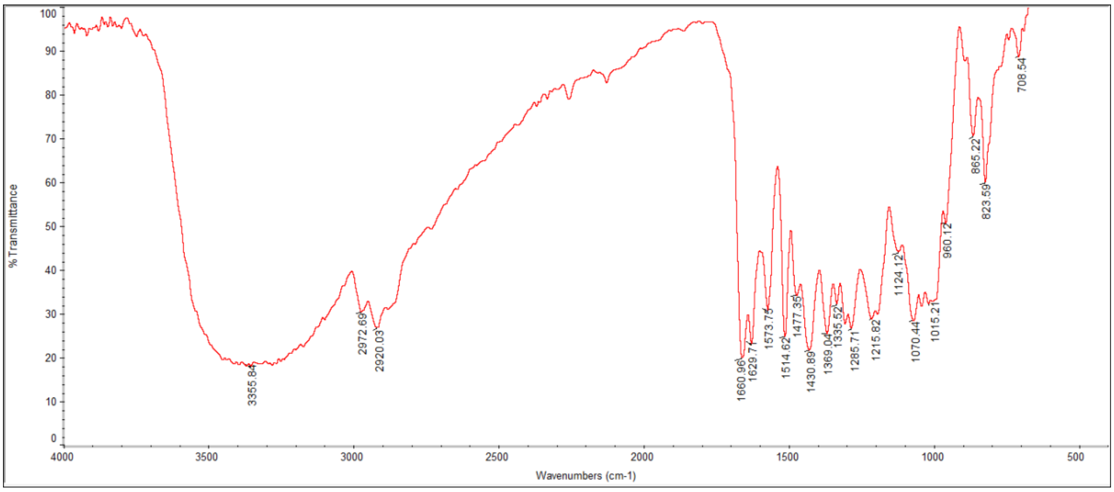

Supplementary Figure 142 IR (KBr disc) spectrum of **14**.

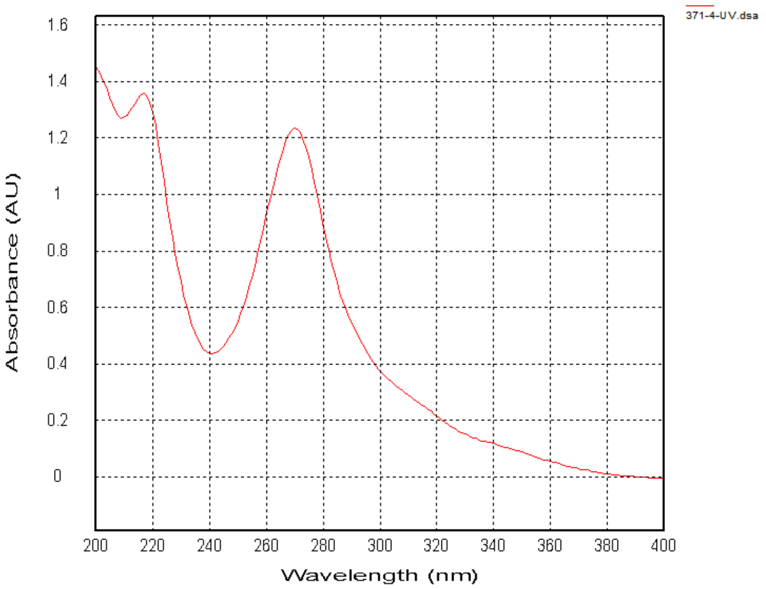

Supplementary Figure 143 UV spectrum of **14**.

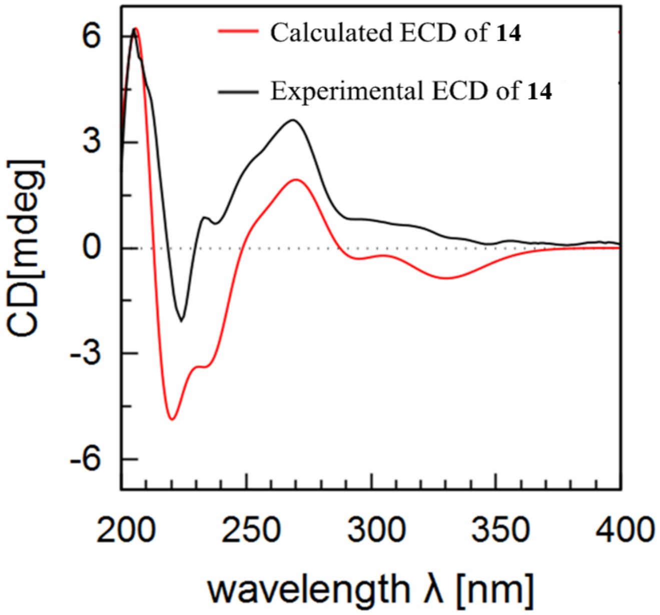

Supplementary Figure 144 Experimental ECD spectra and calculated ECD spectrum of **14**.

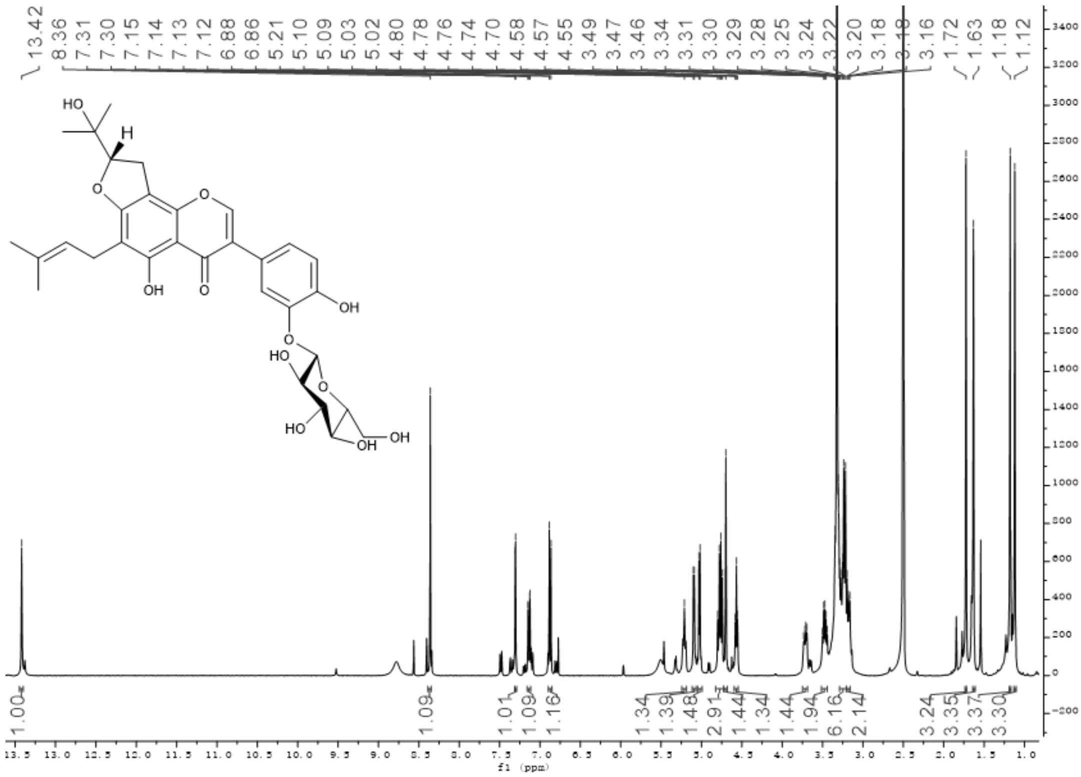

Supplementary Figure 145 ^{1}H NMR (600 MHz) spectrum of **15** in DMSO.

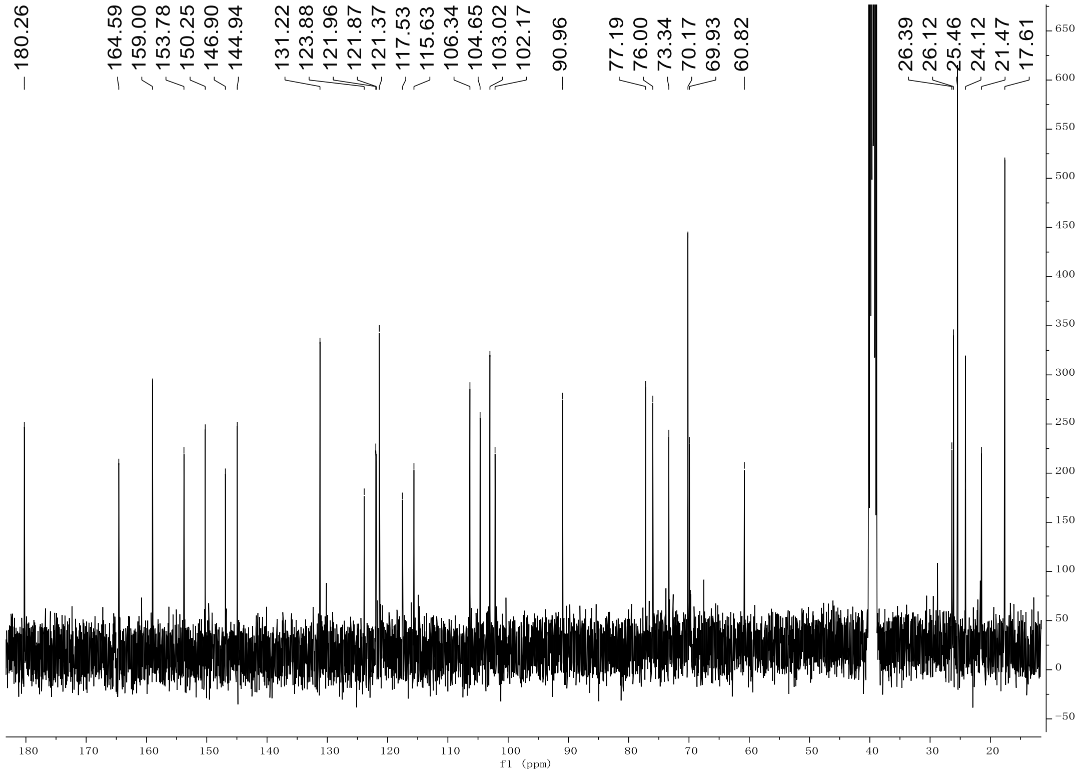

Supplementary Figure 146 ^{13}C NMR (150 MHz) spectrum of **15** in DMSO.

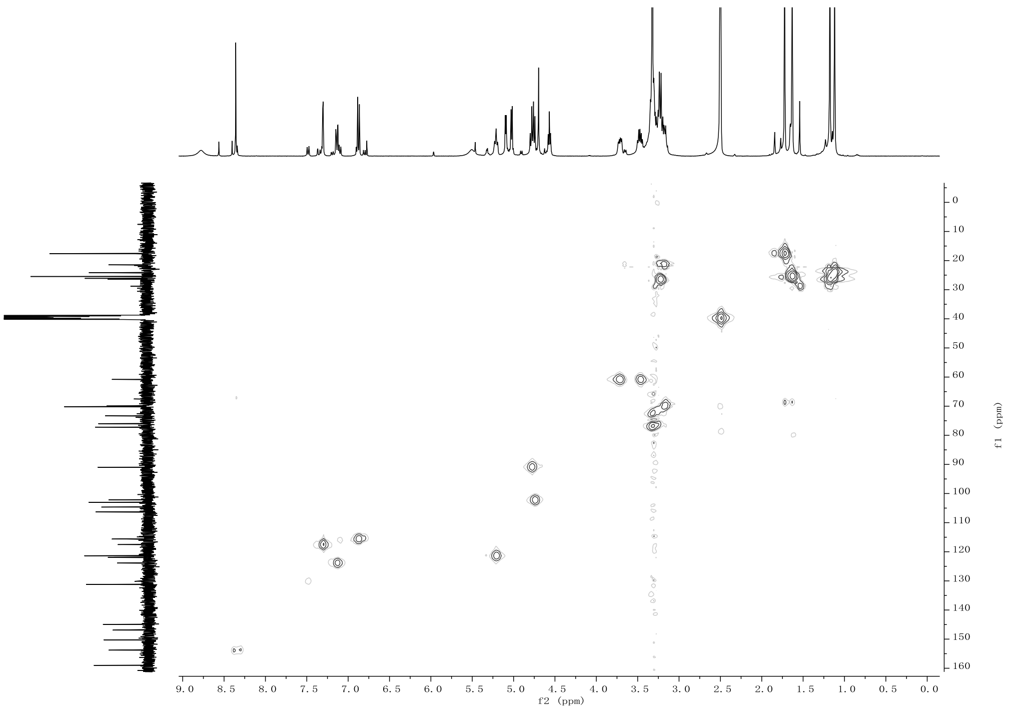

Supplementary Figure 147 HMQC (600 MHz) spectrum of **15** in DMSO.

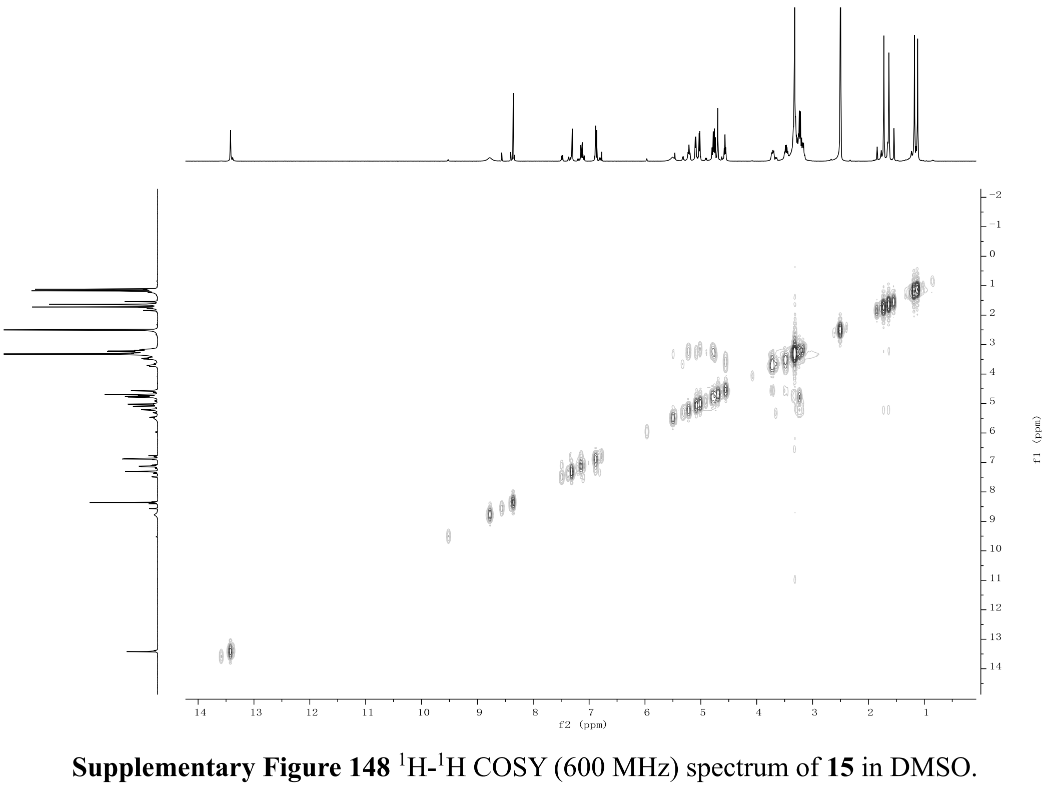

Supplementary Figure 148 ^{1}H-^{1}H COSY (600 MHz) spectrum of **15** in DMSO.

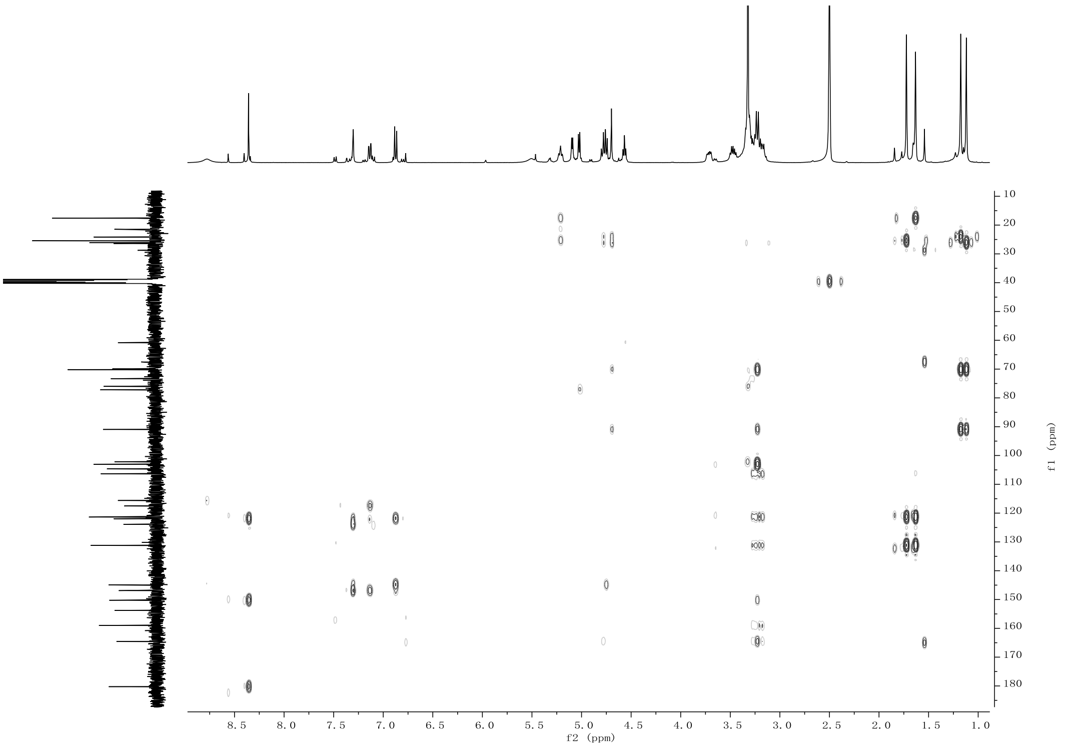

Supplementary Figure 149 HMBC (600 MHz) spectrum of **15** in DMSO.

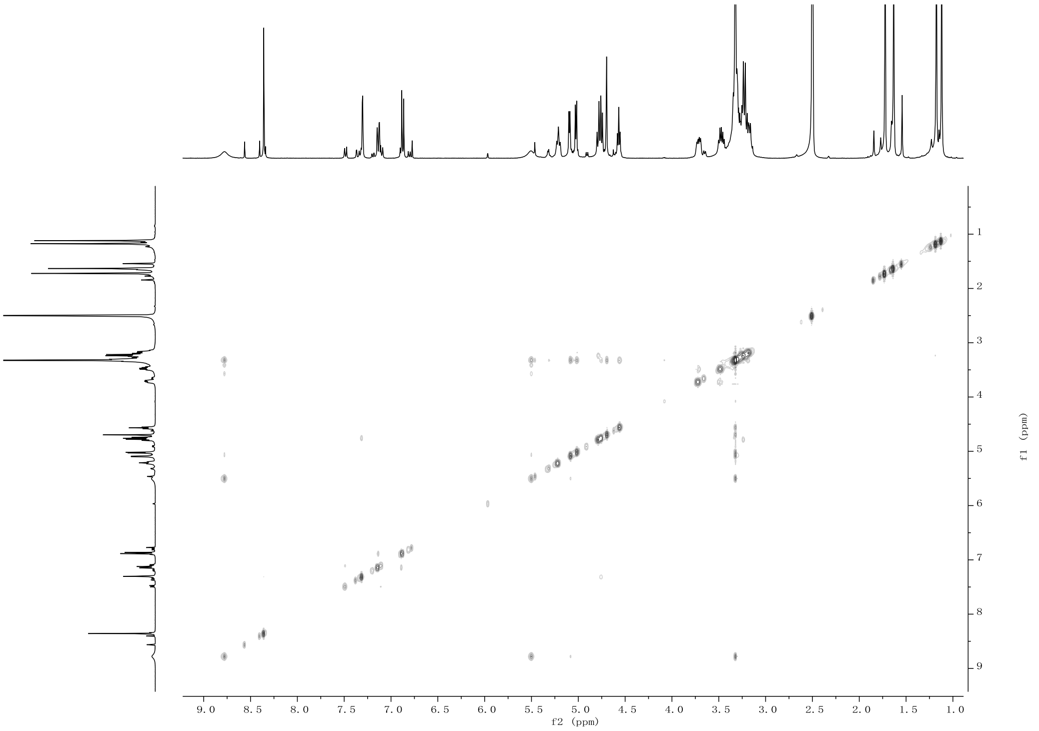

Supplementary Figure 150 ^{1}H-^{1}H NOESY (600 MHz) spectrum of **15** in DMSO.

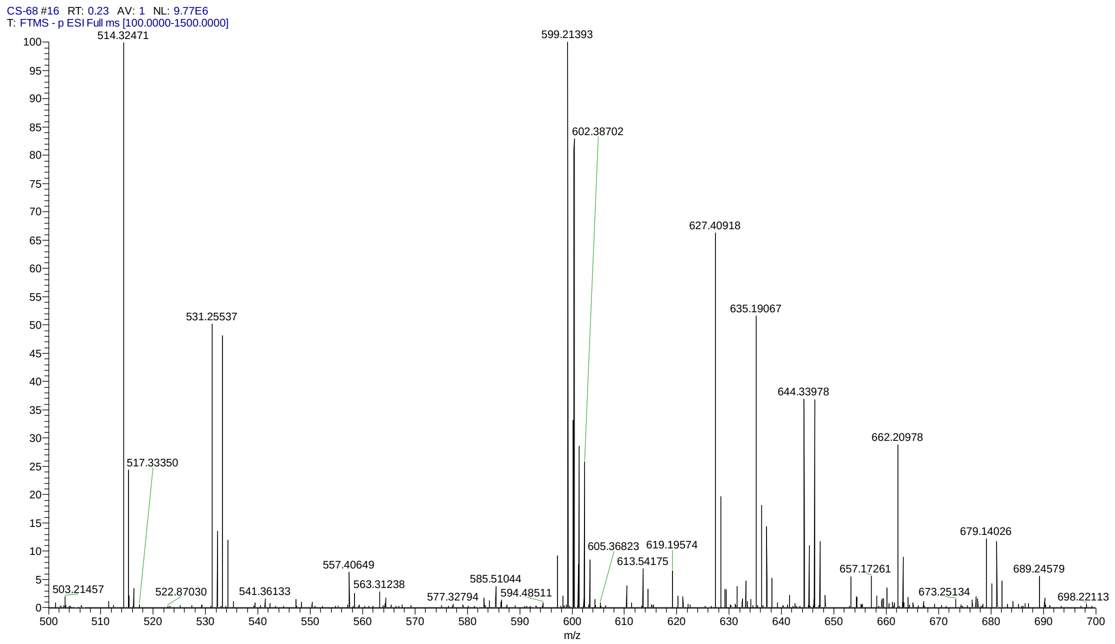

Supplementary Figure 151 HRESIMS spectrum of **15**.

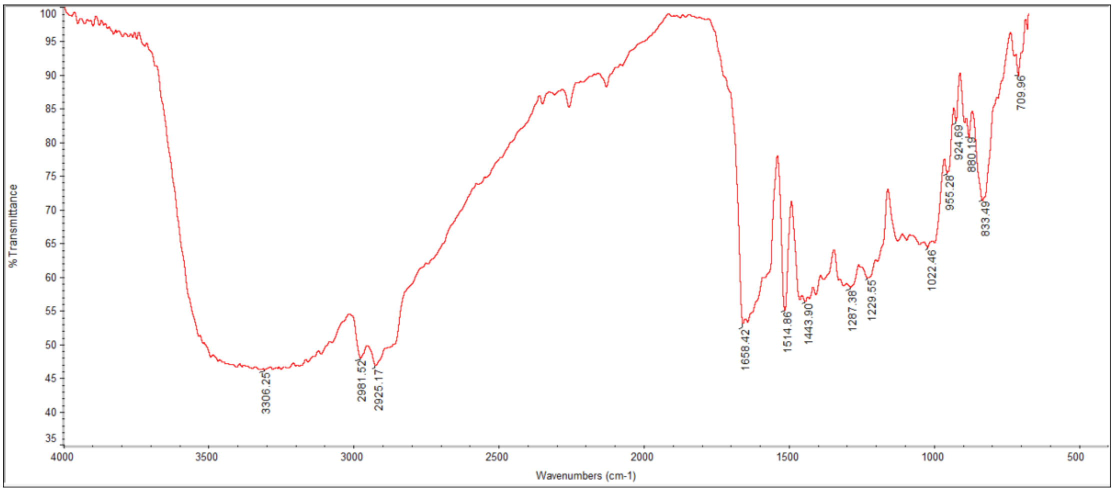

Supplementary Figure 152 IR (KBr disc) spectrum of **15**.

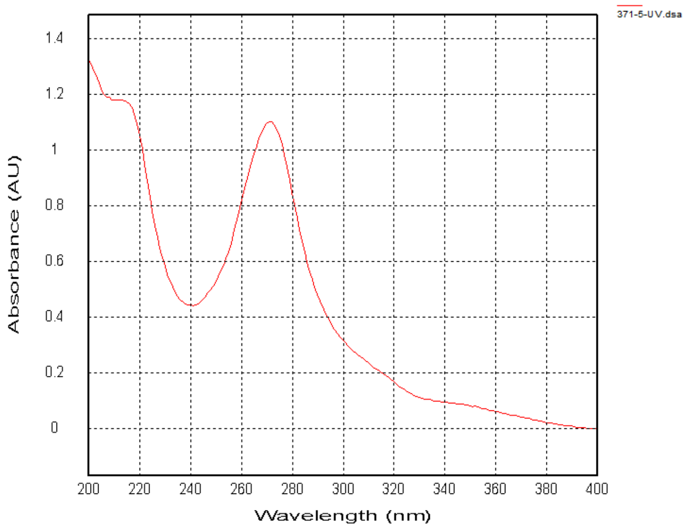

Supplementary Figure 153 UV spectrum of **15**.

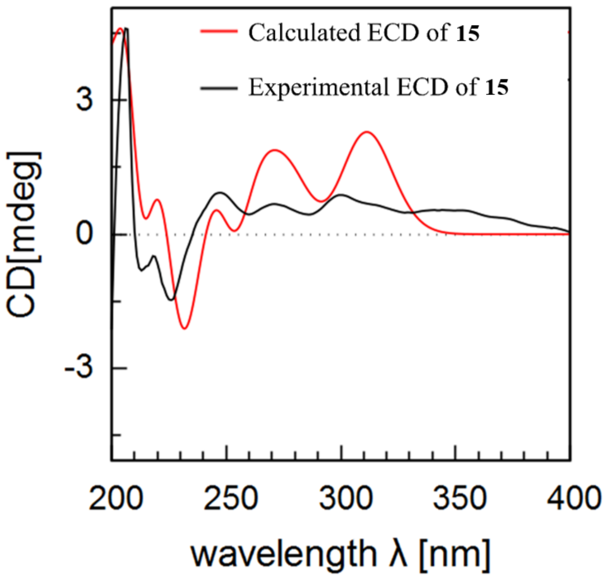

Supplementary Figure 154 Experimental ECD spectra and calculated ECD spectrum of **15**.

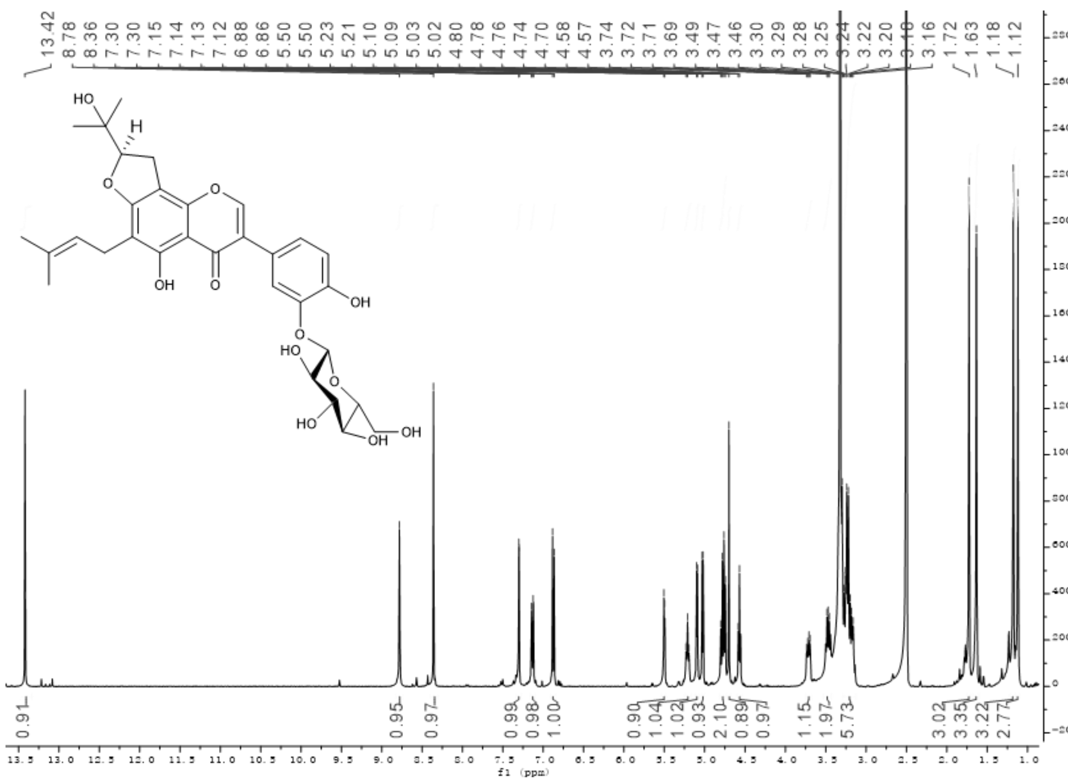

Supplementary Figure 155 ^{1}H NMR (600 MHz) spectrum of **16** in DMSO.

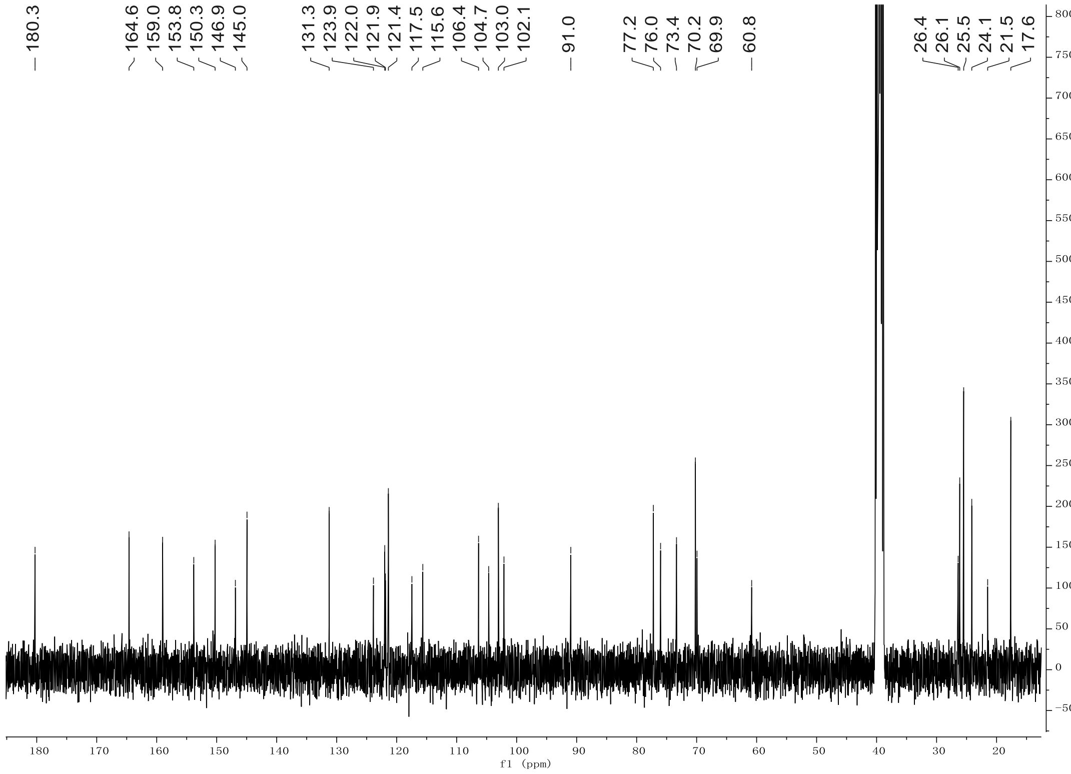

Supplementary Figure 156 ^{13}C NMR (150 MHz) spectrum of **16** in DMSO.

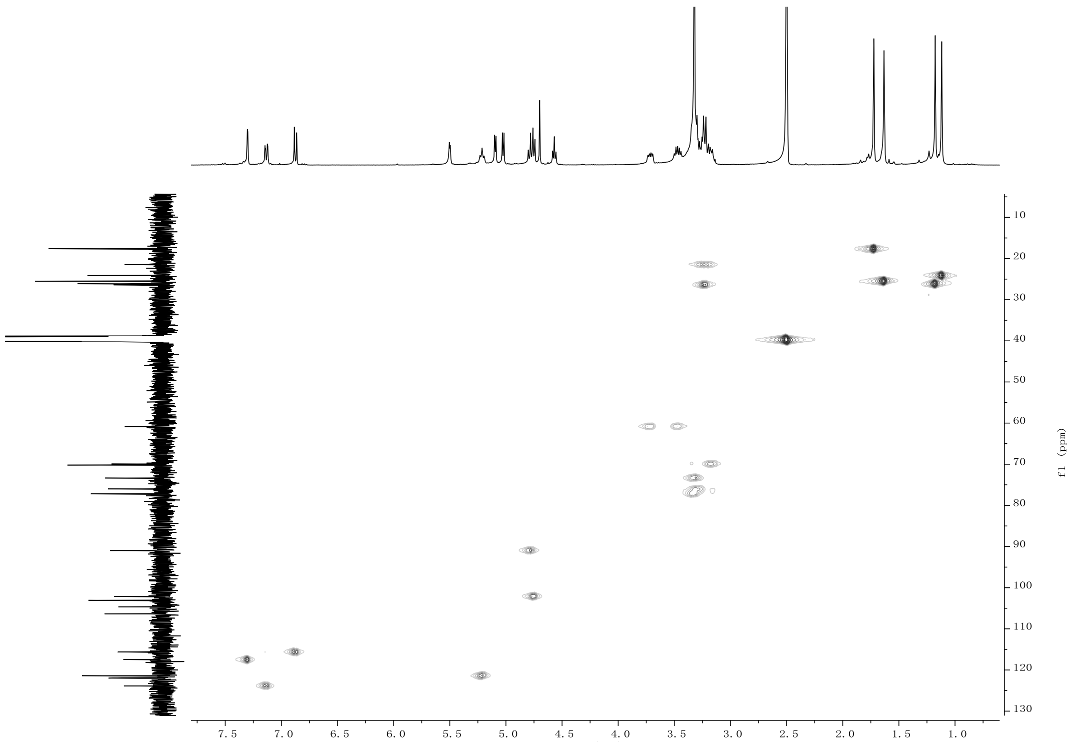

Supplementary Figure 157 HMQC (600 MHz) spectrum of **16** in DMSO.

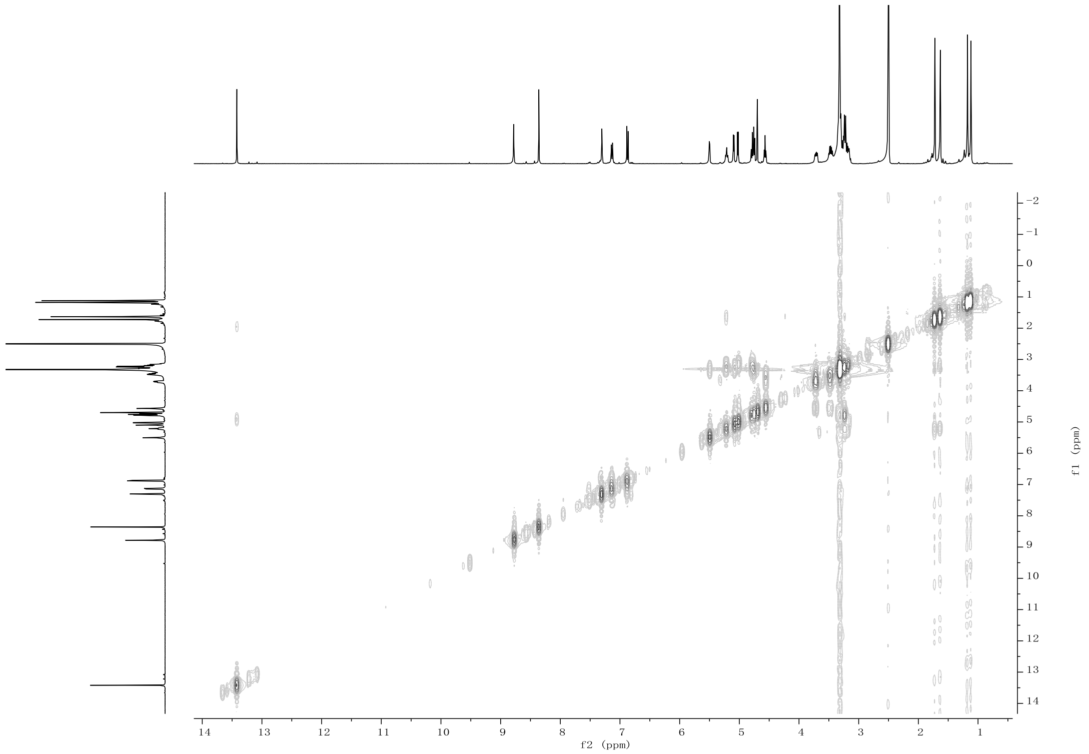

Supplementary Figure 158 ^{1}H-^{1}H COSY (600 MHz) spectrum of **16** in DMSO.

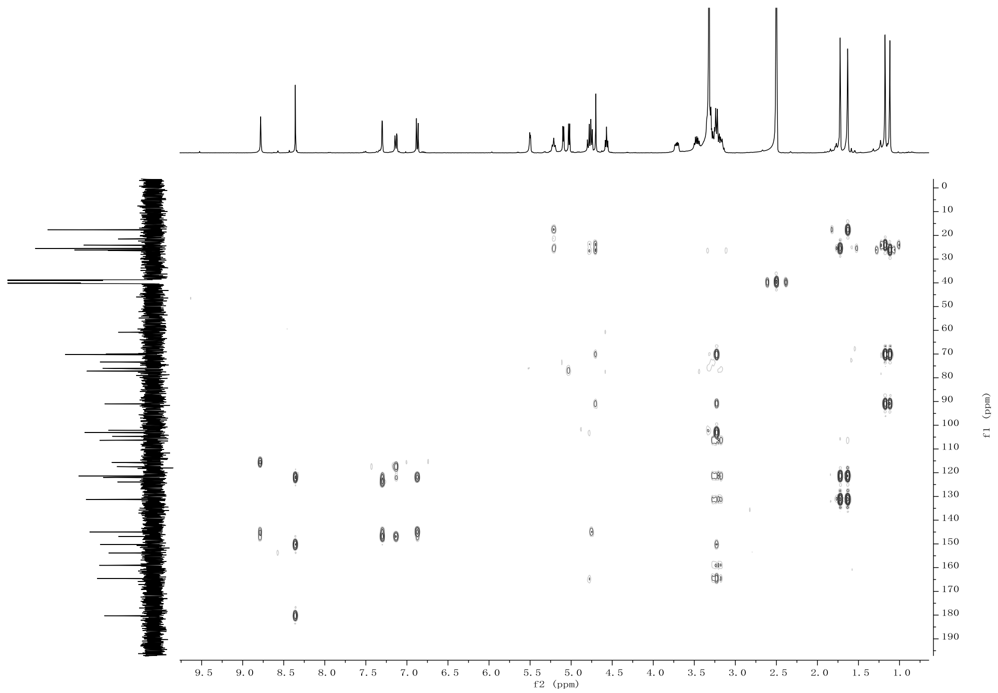

Supplementary Figure 159 HMBC (600 MHz) spectrum of **16** in DMSO.

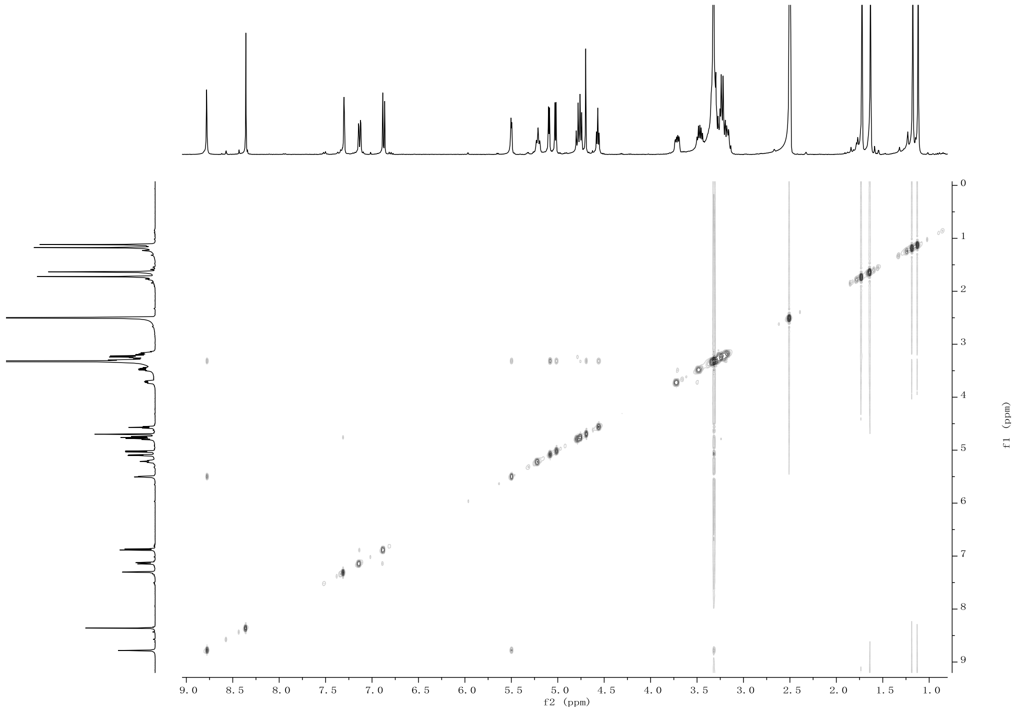

Supplementary Figure S160 ^{1}H-^{1}H NOESY (600 MHz) spectrum of **16** in DMSO.

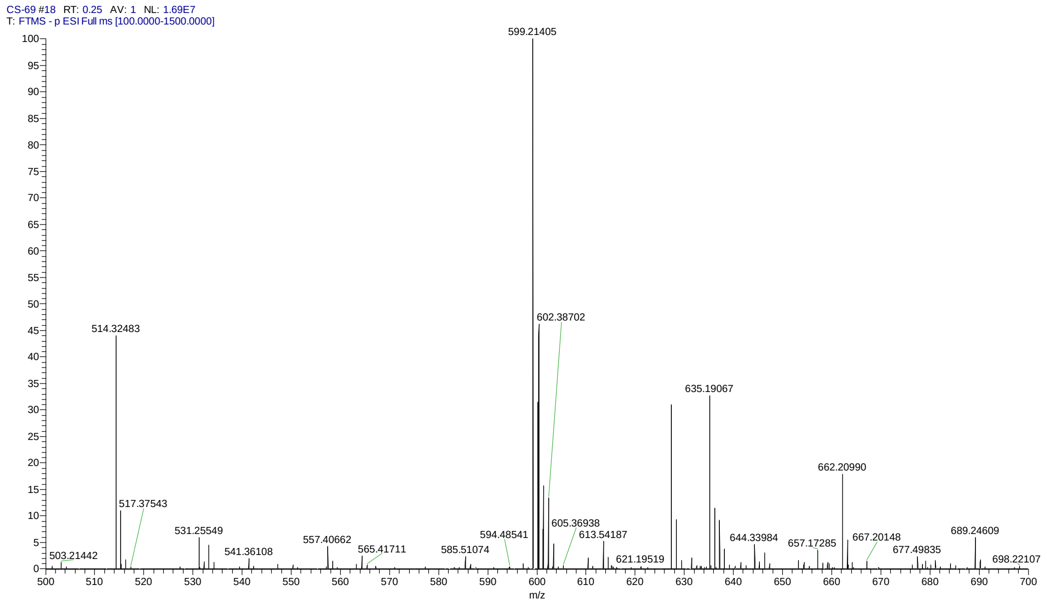

Supplementary Figure 161 HRESIMS spectrum of **16**.

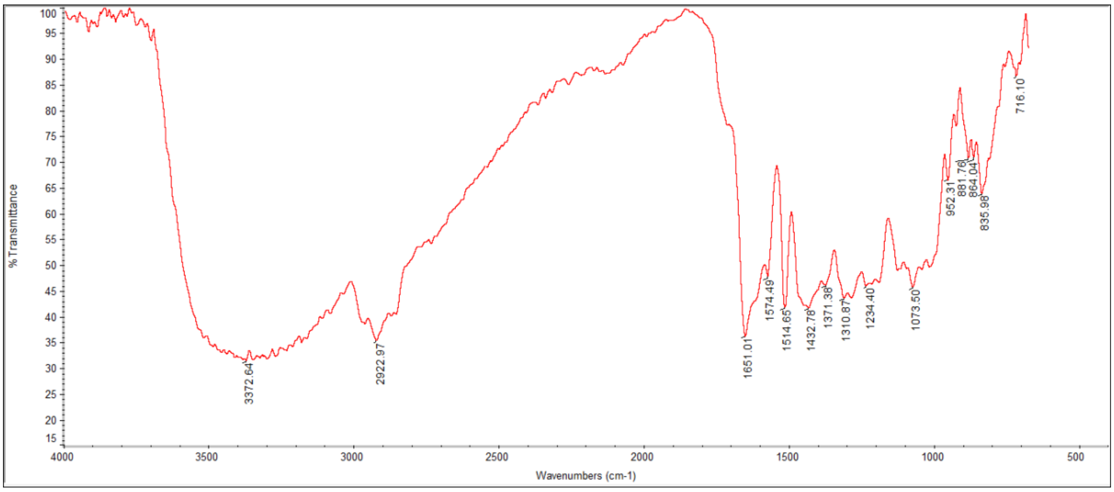

Supplementary Figure 162 IR (KBr disc) spectrum of **16**.

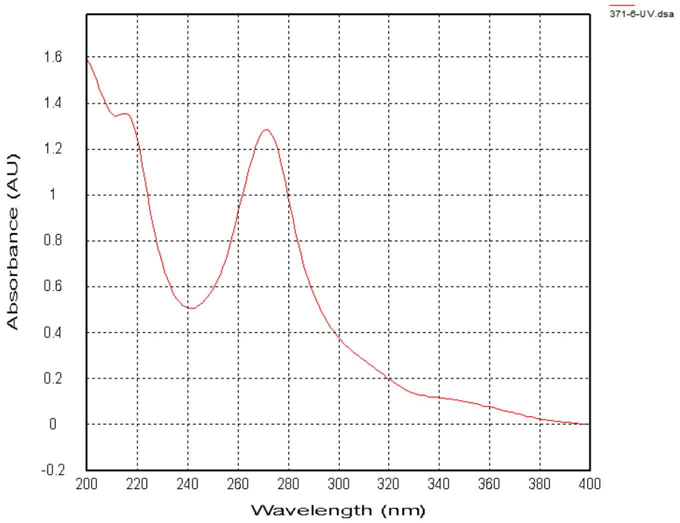

Supplementary Figure 163 UV spectrum of **16**.

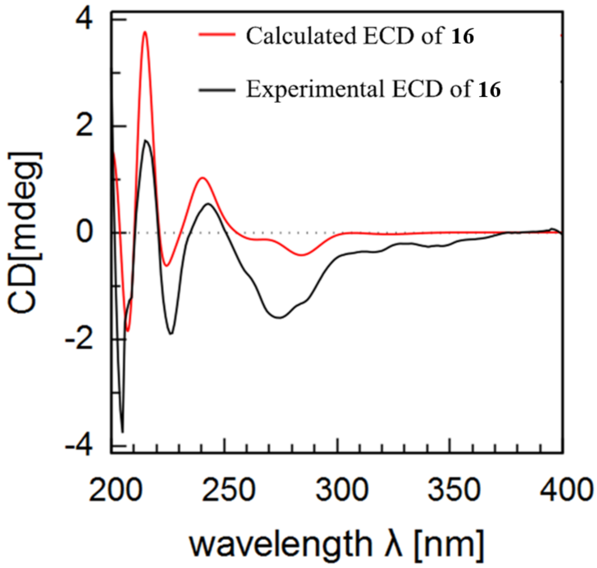

Supplementary Figure 164 Experimental ECD spectra and calculated ECD spectrum of **16**.

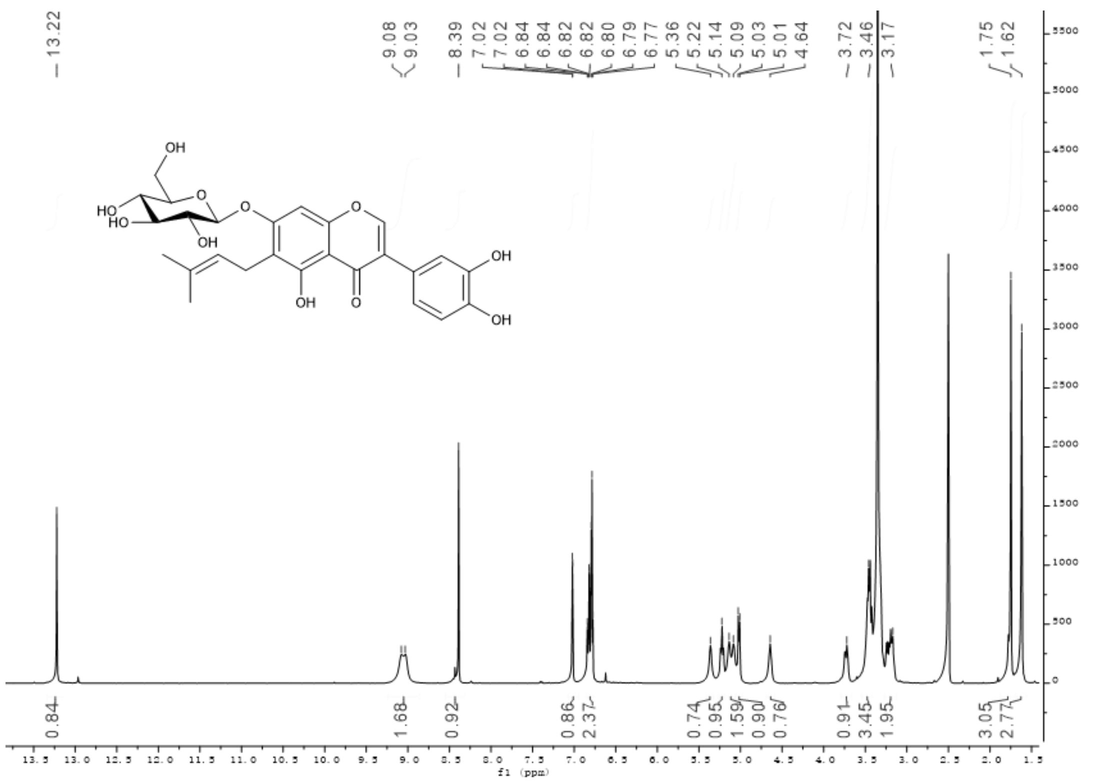

Supplementary Figure 165 ^{1}H NMR (400 MHz) spectrum of **17** in DMSO.

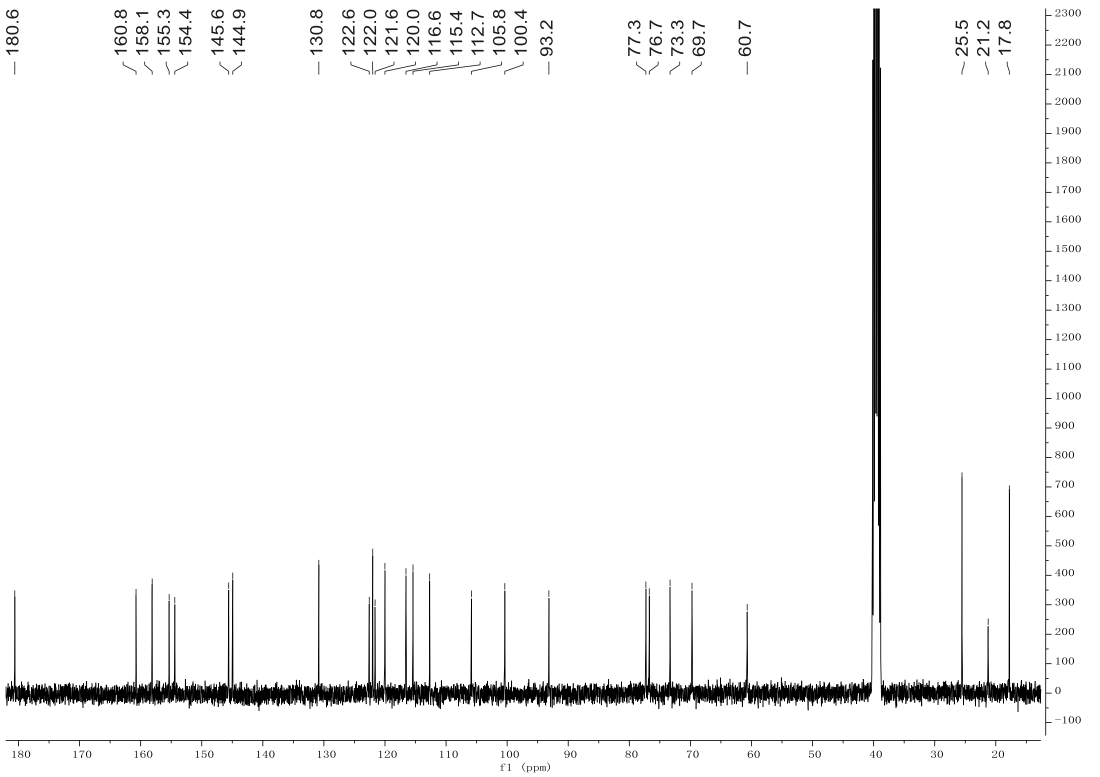

Supplementary Figure 166 ^{13}C NMR (100 MHz) spectrum of **17** in DMSO.

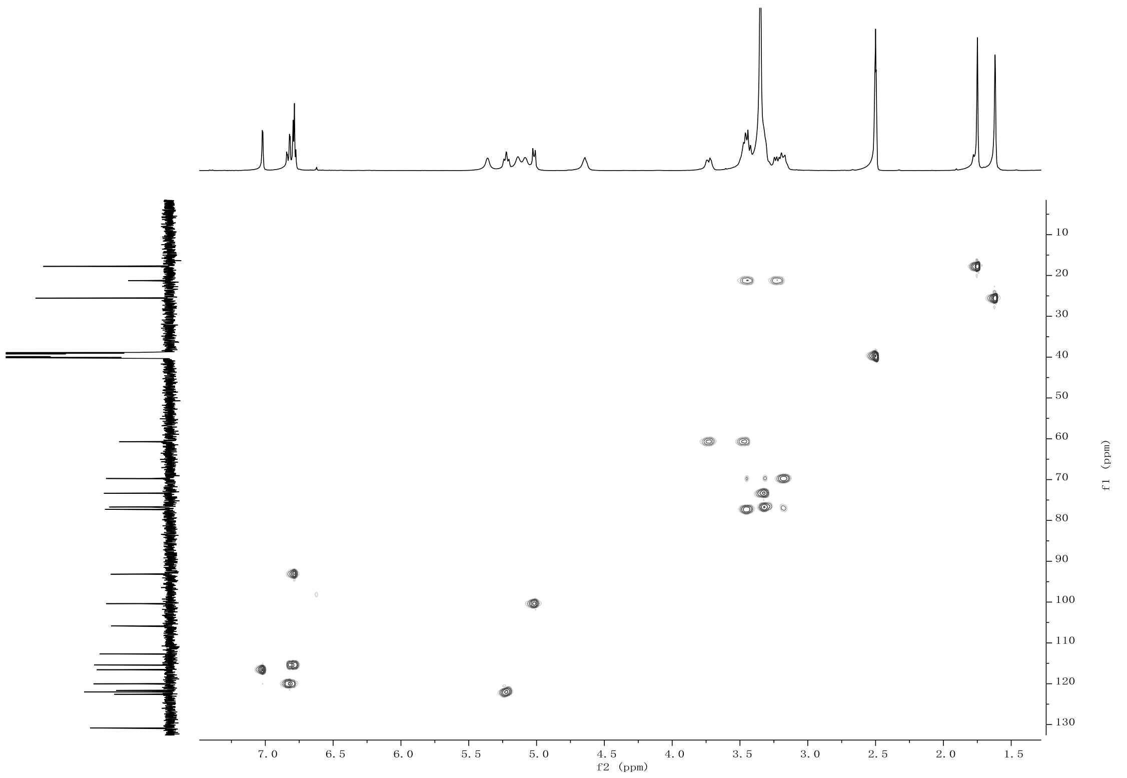

Supplementary Figure 167 HSQC (400 MHz) spectrum of **17** in DMSO.

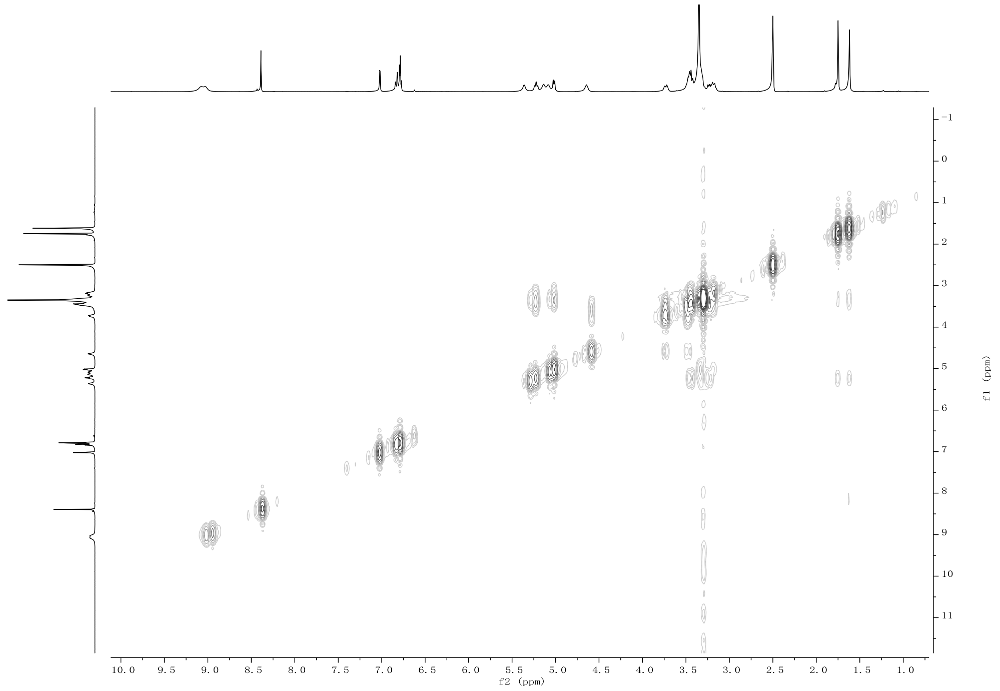

Supplementary Figure 168 ^{1}H-^{1}H COSY (600 MHz) spectrum of **17** in DMSO.

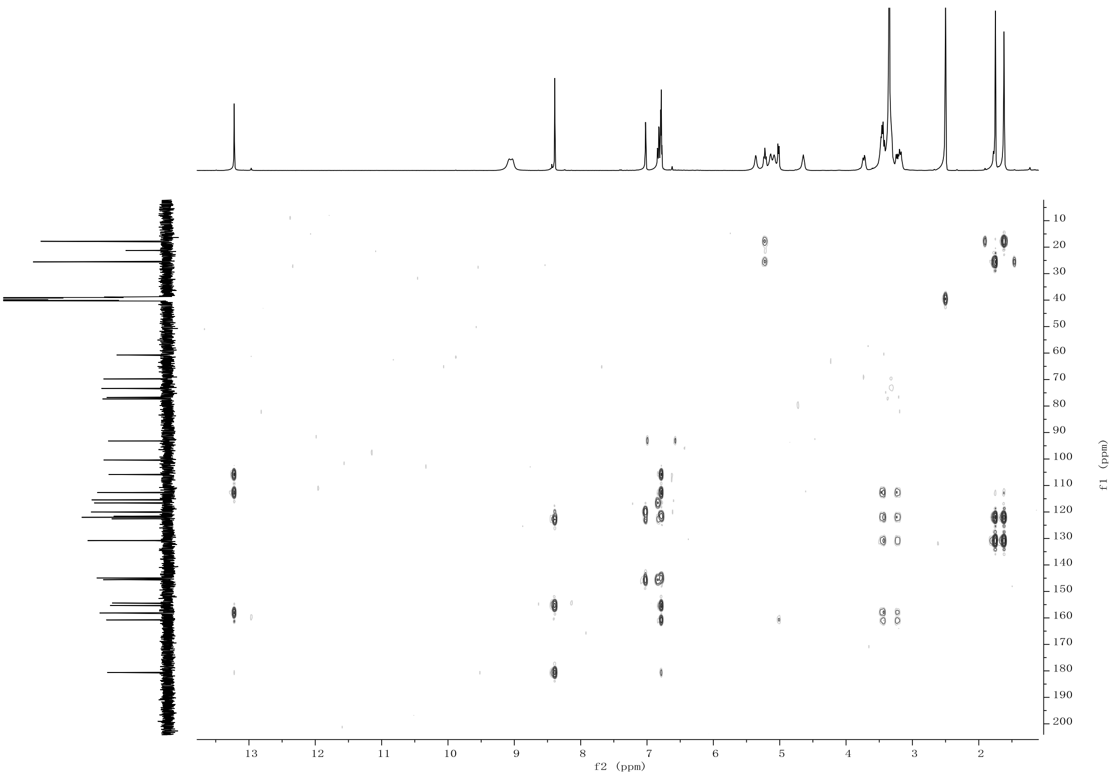

Supplementary Figure 169 HMBC (400 MHz) spectrum of **17** in DMSO.

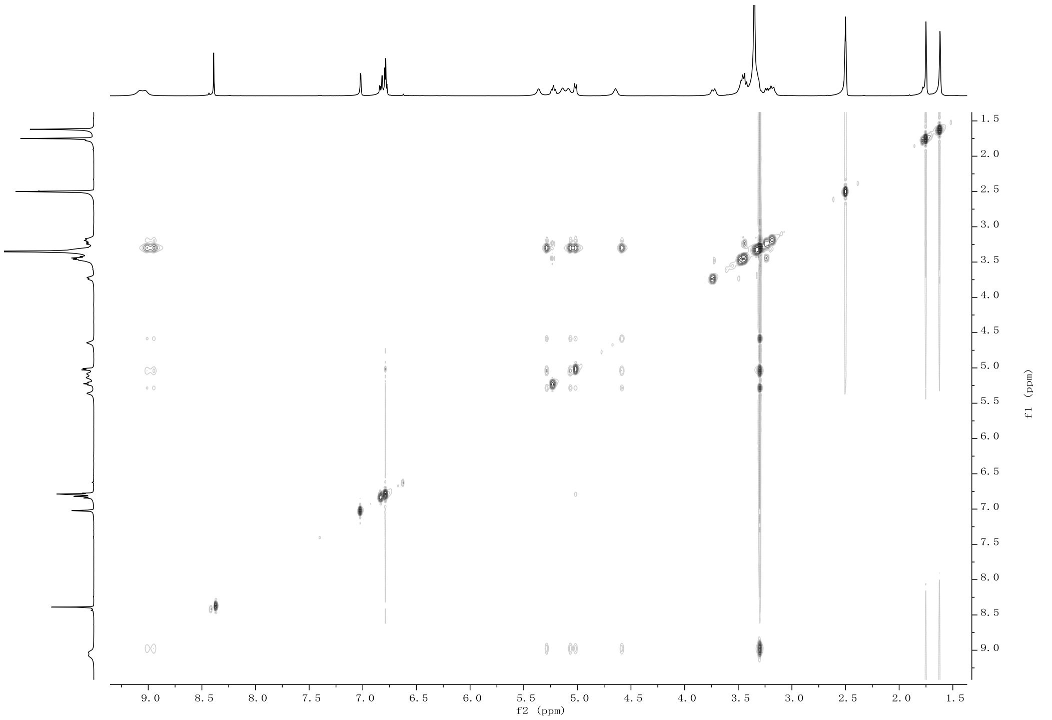

Supplementary Figure 170 ^{1}H-^{1}H NOESY (600 MHz) spectrum of **17** in DMSO.

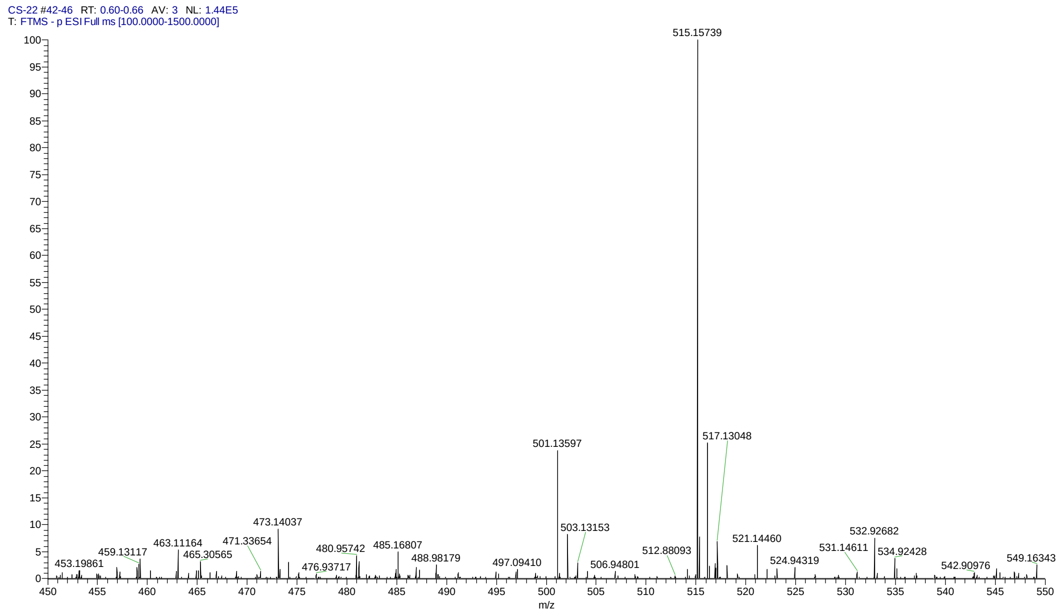

Supplementary Figure 171 HRESIMS spectrum of **17**.

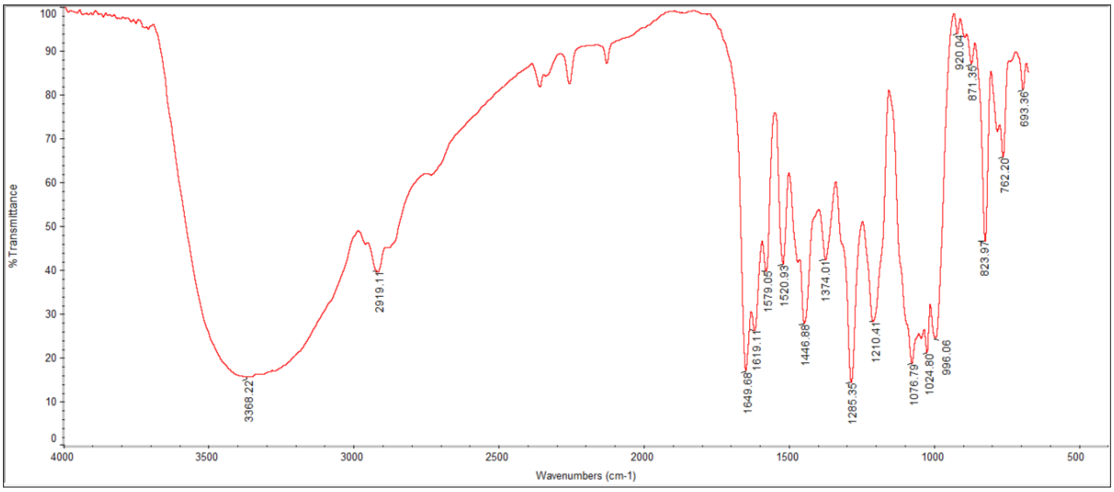

Supplementary Figure 172 IR (KBr disc) spectrum of **17**.

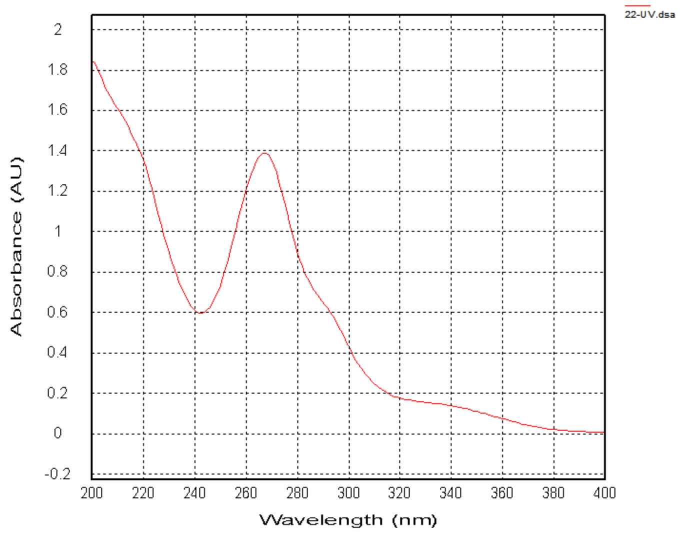

Supplementary Figure 173 UV spectrum of **17**.

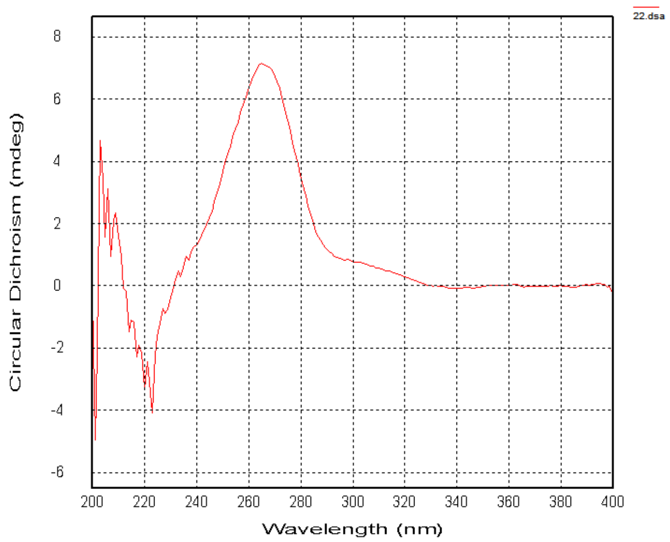

Supplementary Figure 174 ECD spectrum of **17**.

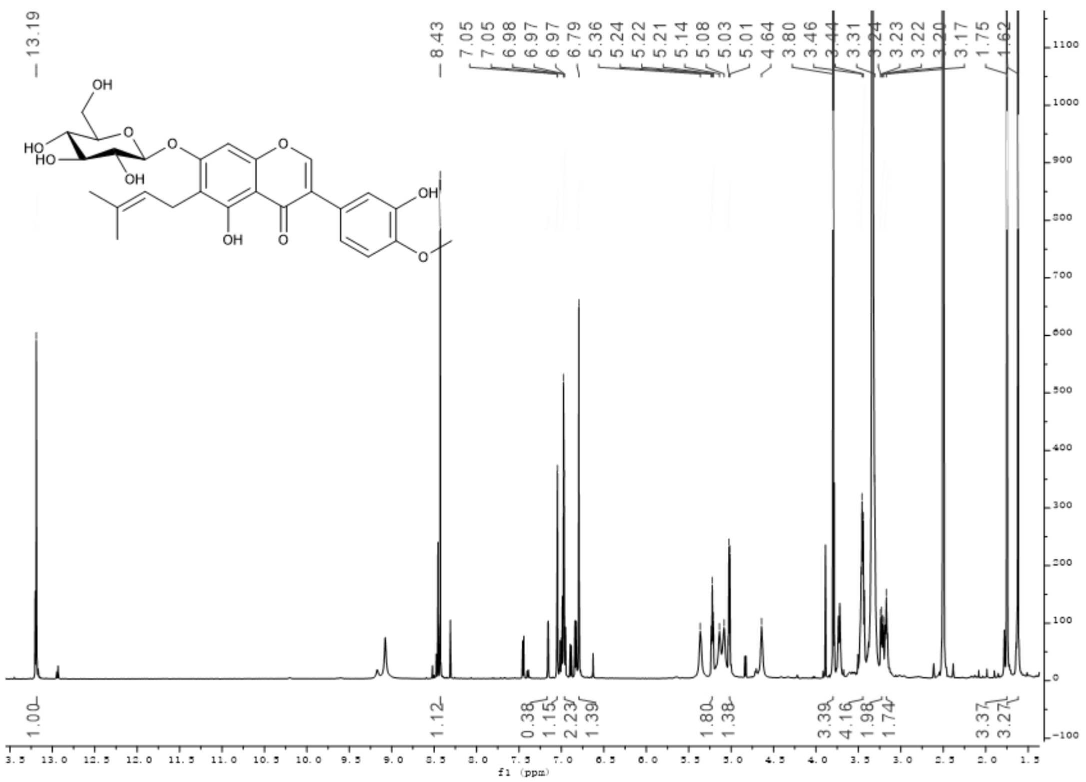

Supplementary Figure 175 ^{1}H NMR (600 MHz) spectrum of **18** in DMSO.

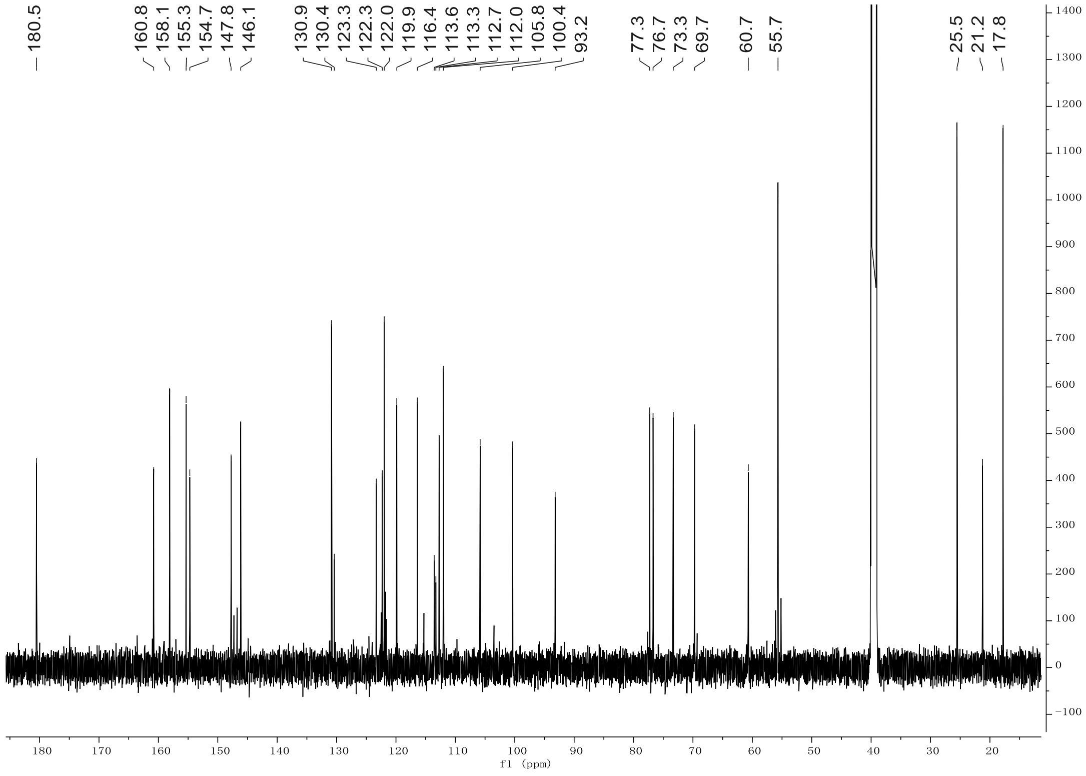

Supplementary Figure 176 ^{13}C NMR (150 MHz) spectrum of **18** in DMSO.

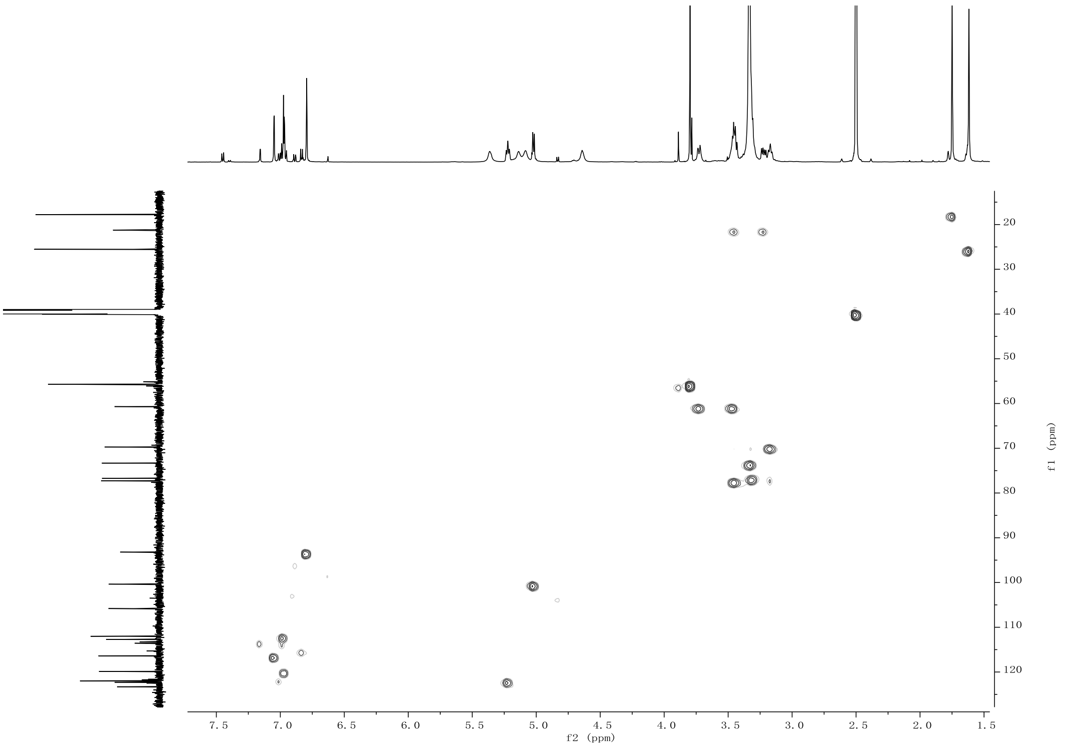

Supplementary Figure 177 HMQC (600 MHz) spectrum of **18** in DMSO.

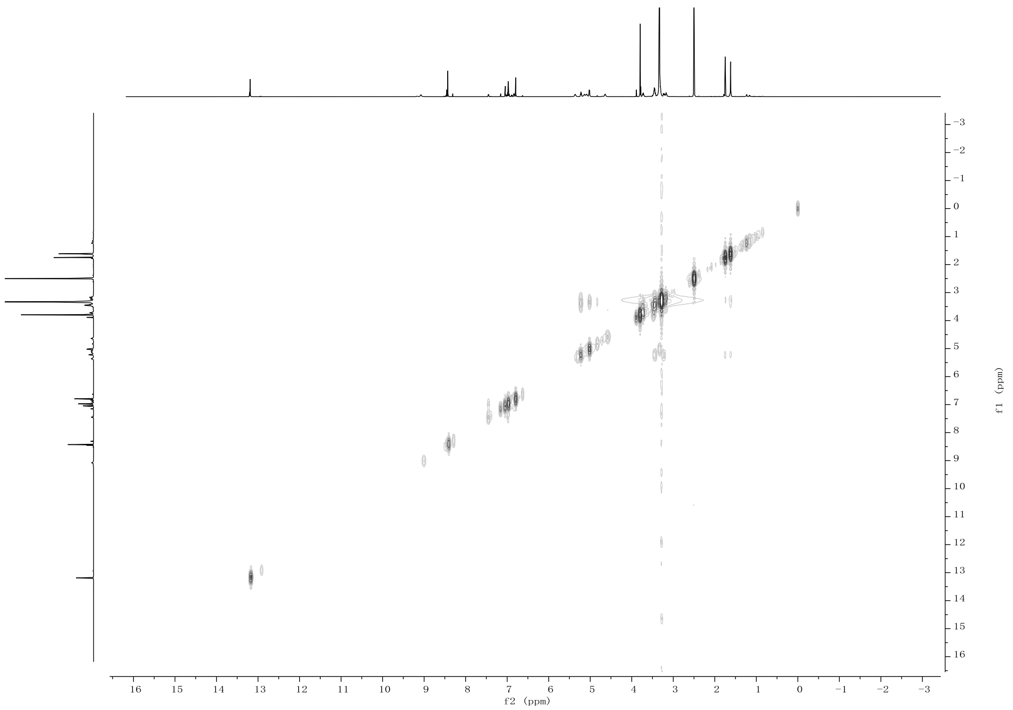

Supplementary Figure 178 ^{1}H-^{1}H COSY (600 MHz) spectrum of **18** in DMSO.

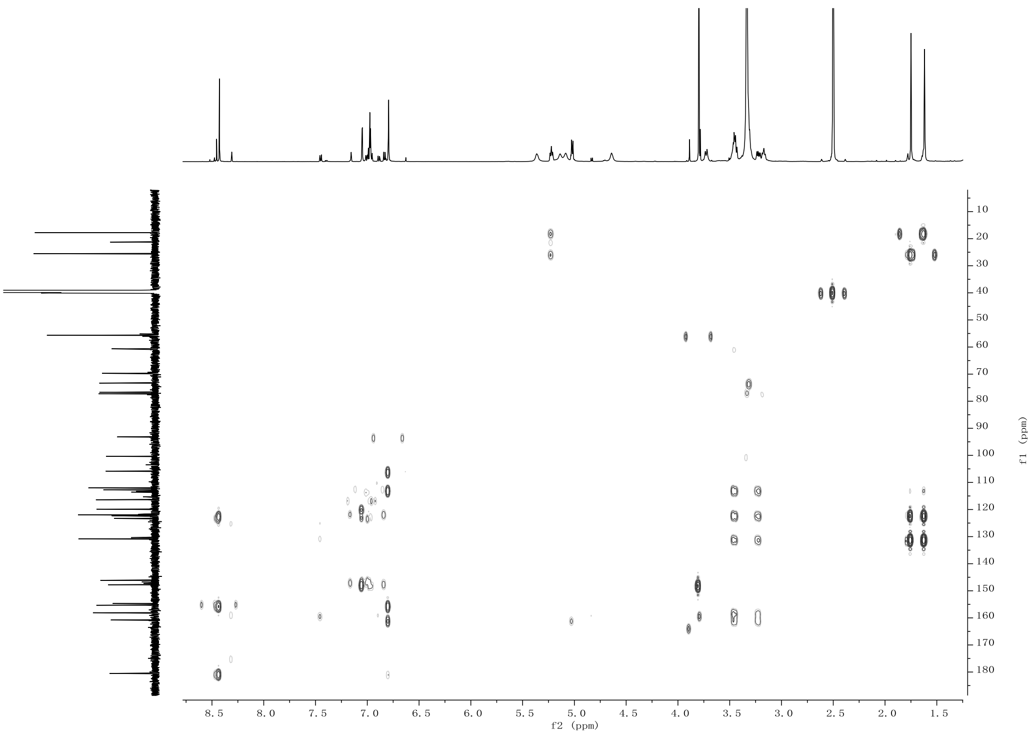

Supplementary Figure 179 HMBC (600 MHz) spectrum of **18** in DMSO.

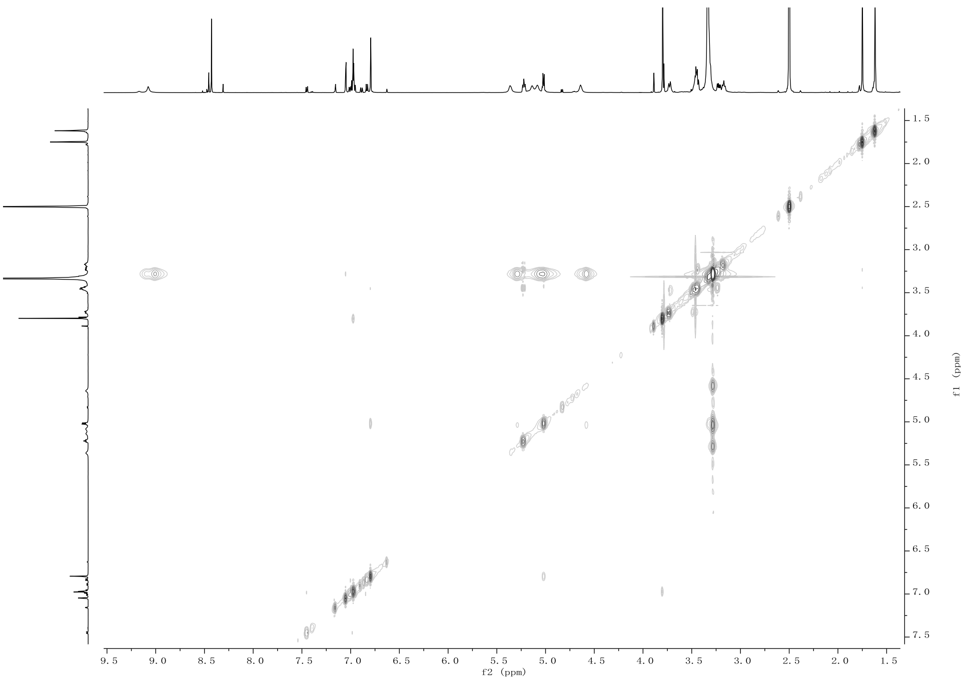

Supplementary Figure 180 ^{1}H-^{1}H NOESY (600 MHz) spectrum of **18** in DMSO.

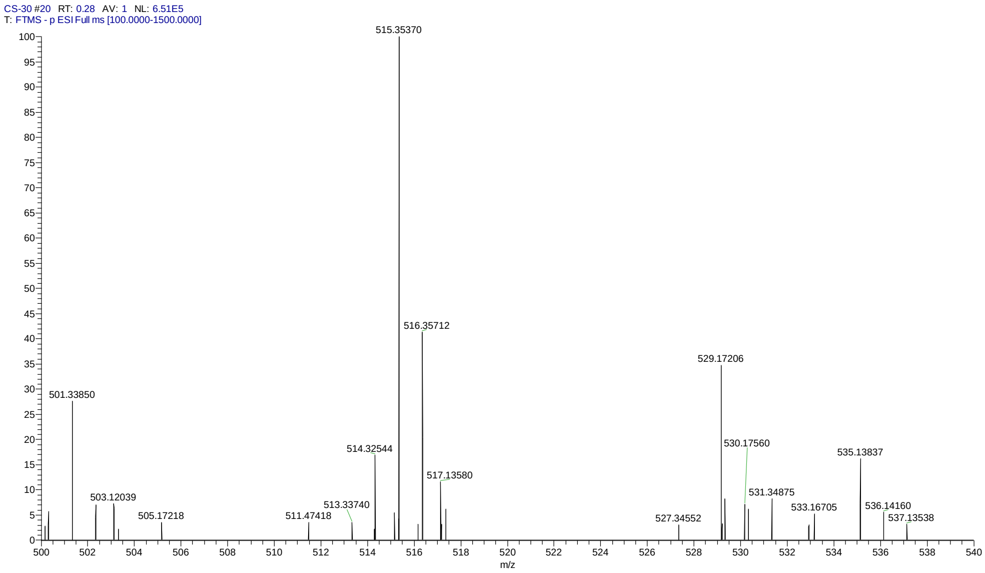

Supplementary Figure 181 HRESIMS spectrum of **18**.

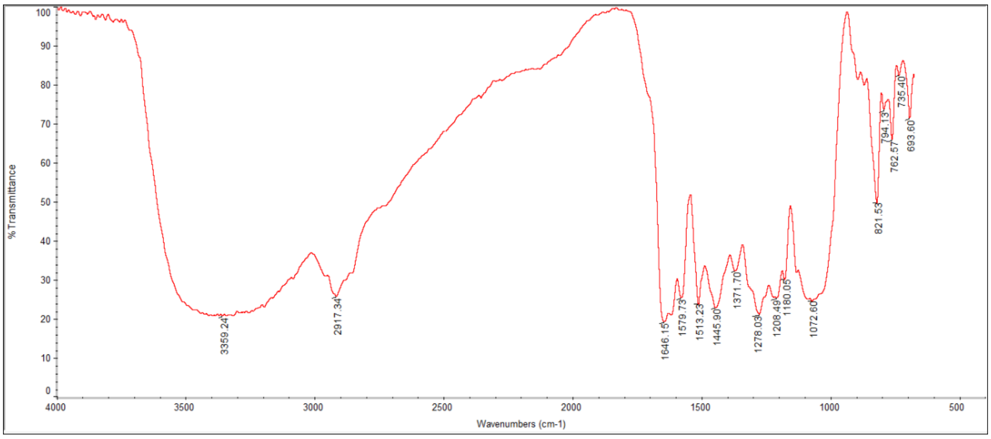

Supplementary Figure 182 IR (KBr disc) spectrum of **18**.

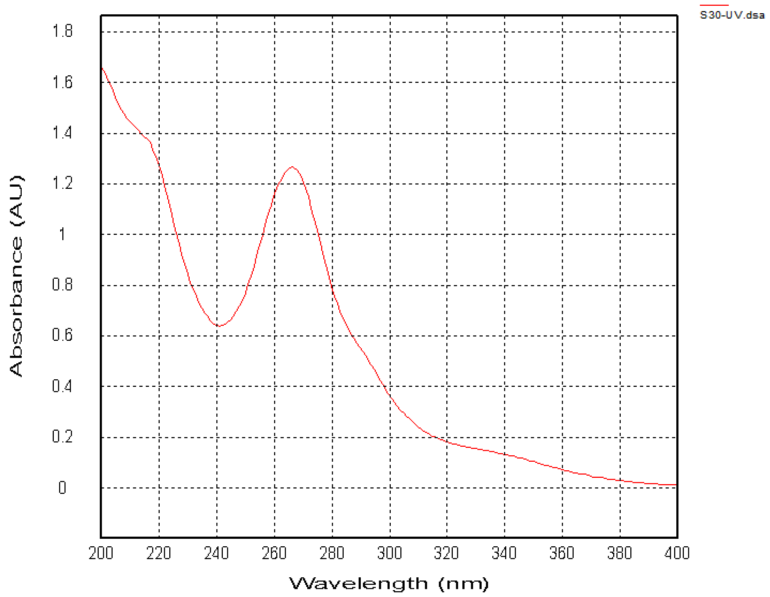

Supplementary Figure 183 UV spectrum of **18**.

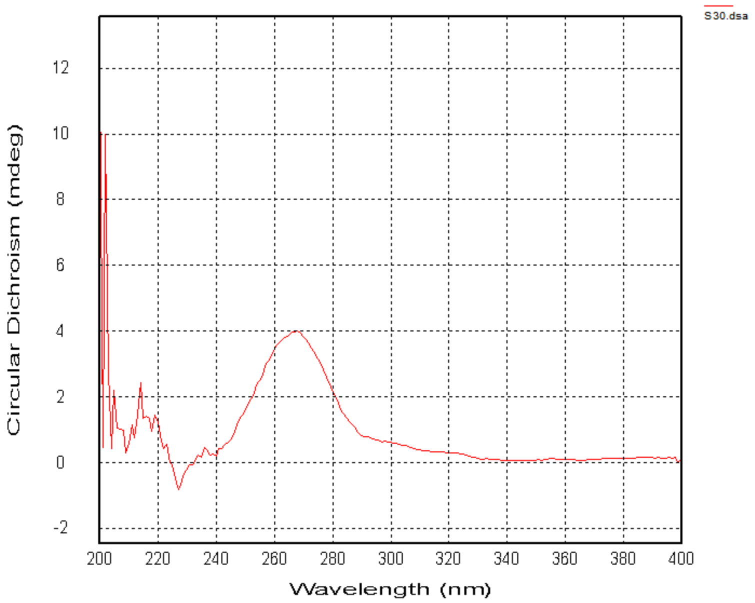

Supplementary Figure 184 ECD spectrum of **18**.

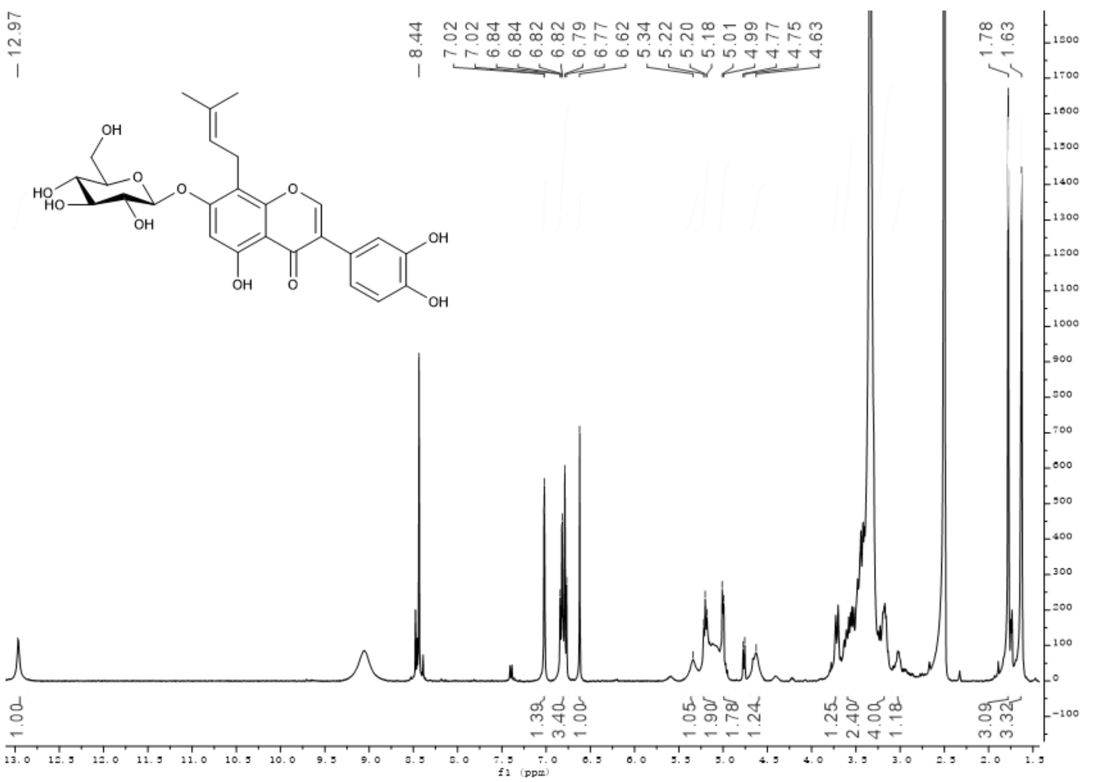

Supplementary Figure 185 ^{1}H NMR (400 MHz) spectrum of **19** in DMSO.

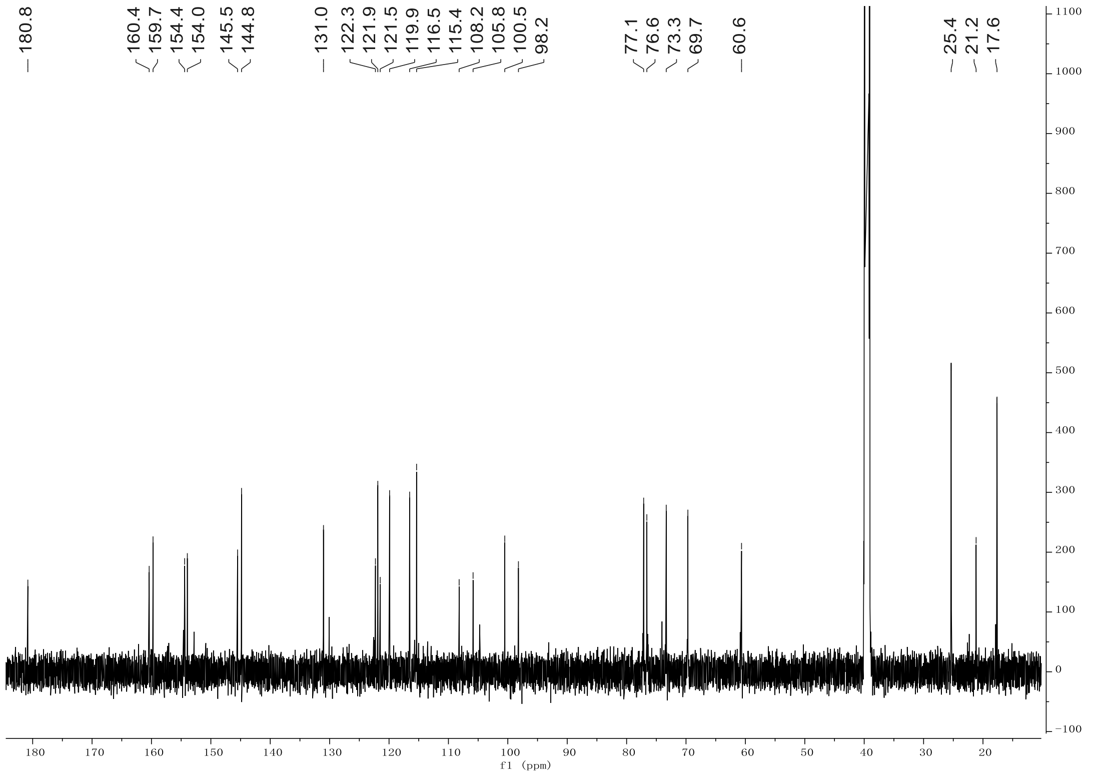

Supplementary Figure 186 ^{13}C NMR (100 MHz) spectrum of **19** in DMSO.

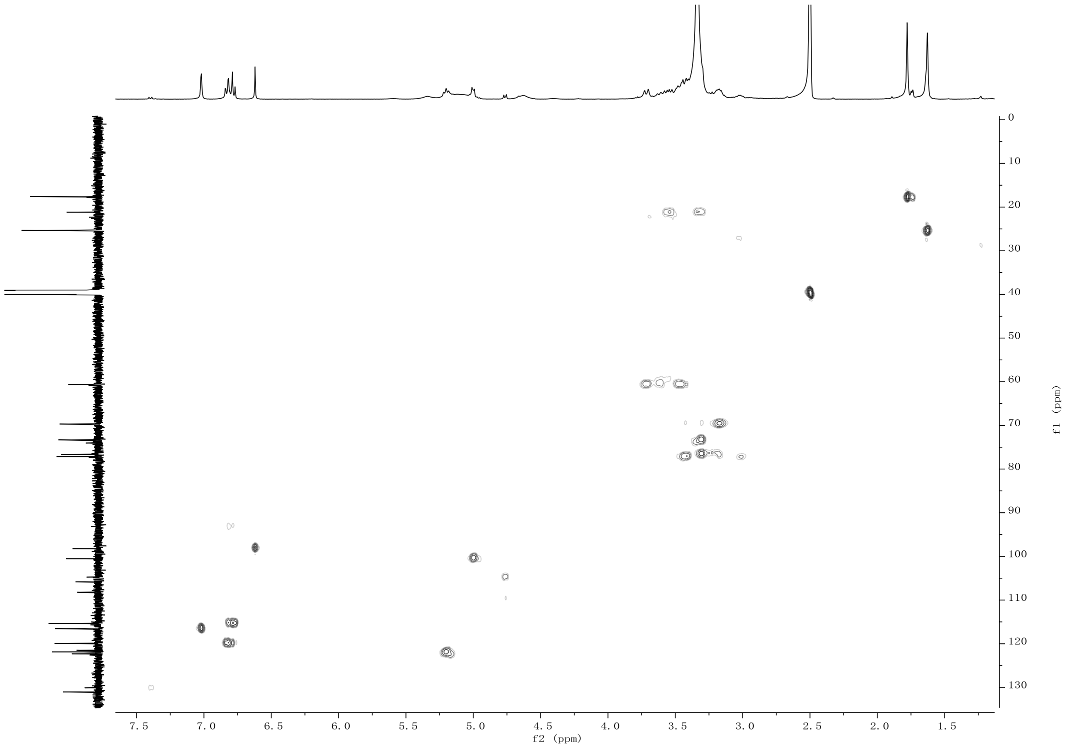

Supplementary Figure 187 HSQC (400 MHz) spectrum of **19** in DMSO.

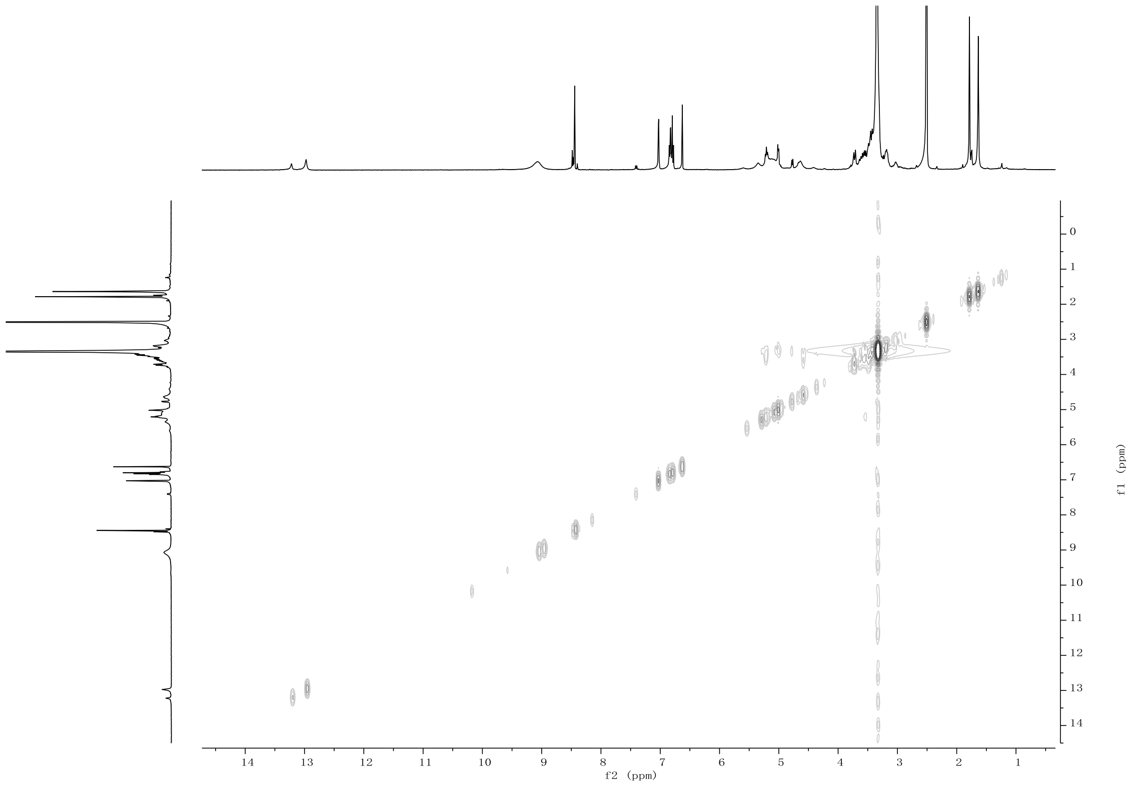

Supplementary Figure 188 ^{1}H-^{1}H COSY (600 MHz) spectrum of **19** in DMSO.

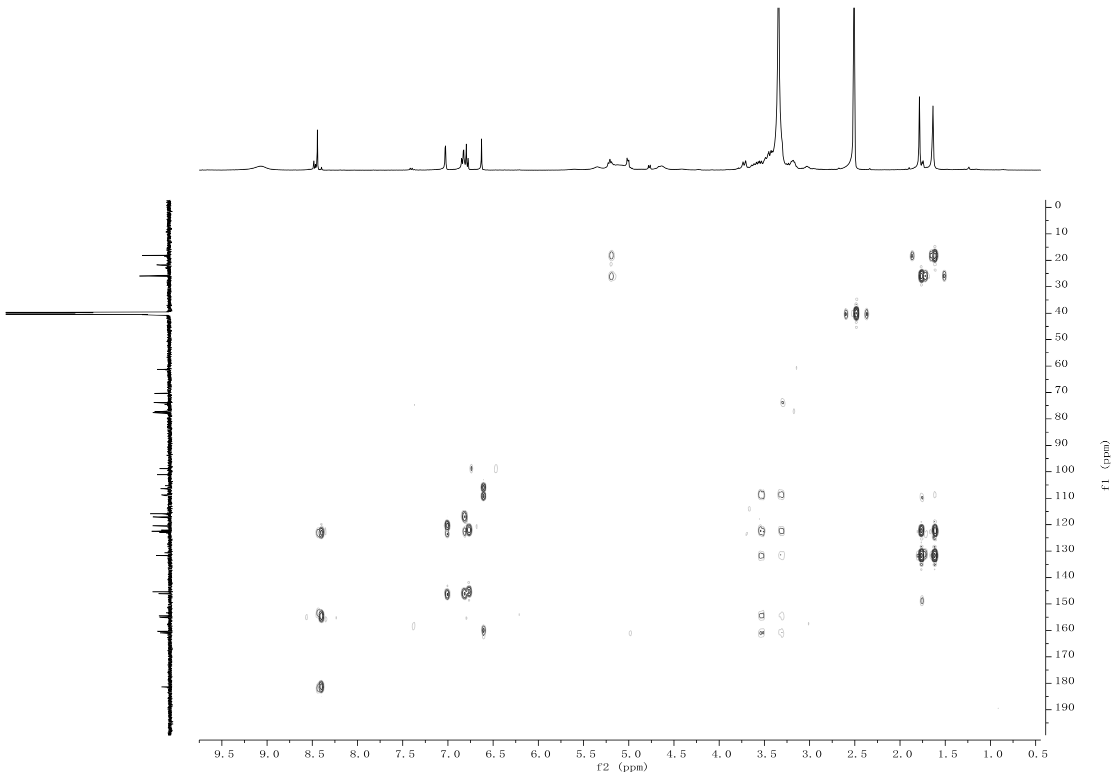

Supplementary Figure 189 HMBC (400 MHz) spectrum of **19** in DMSO.

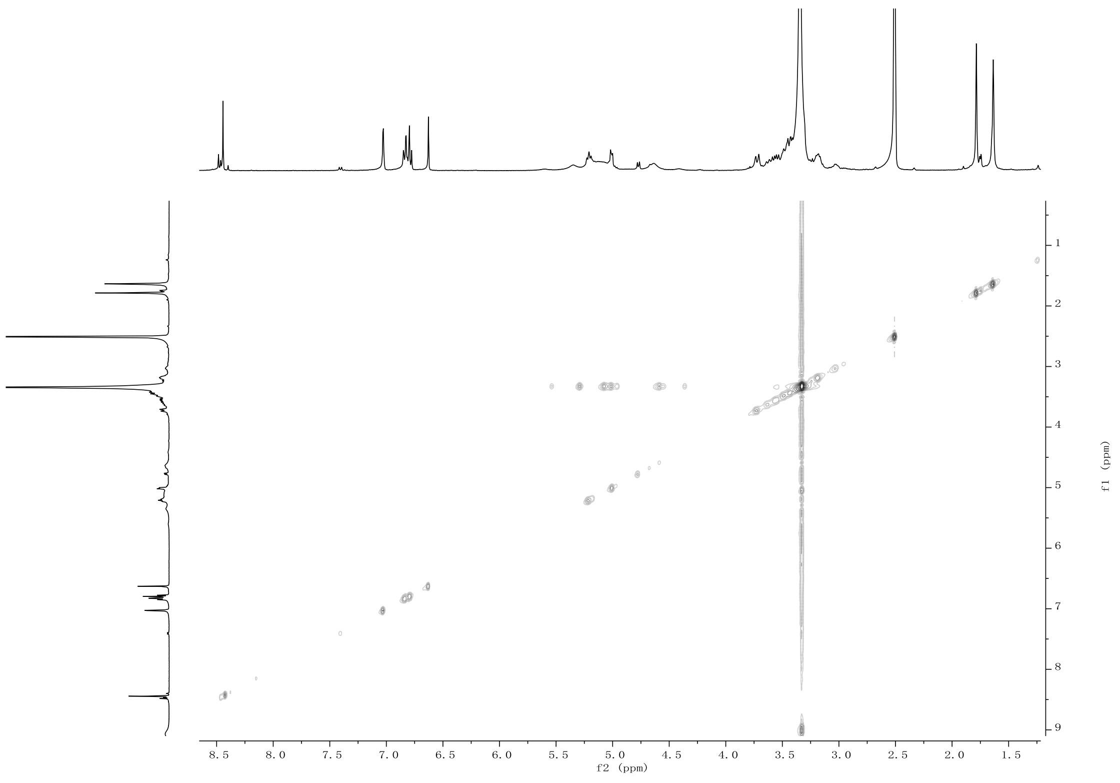

Supplementary Figure 190 ^{1}H-^{1}H NOESY (600 MHz) spectrum of **19** in DMSO.

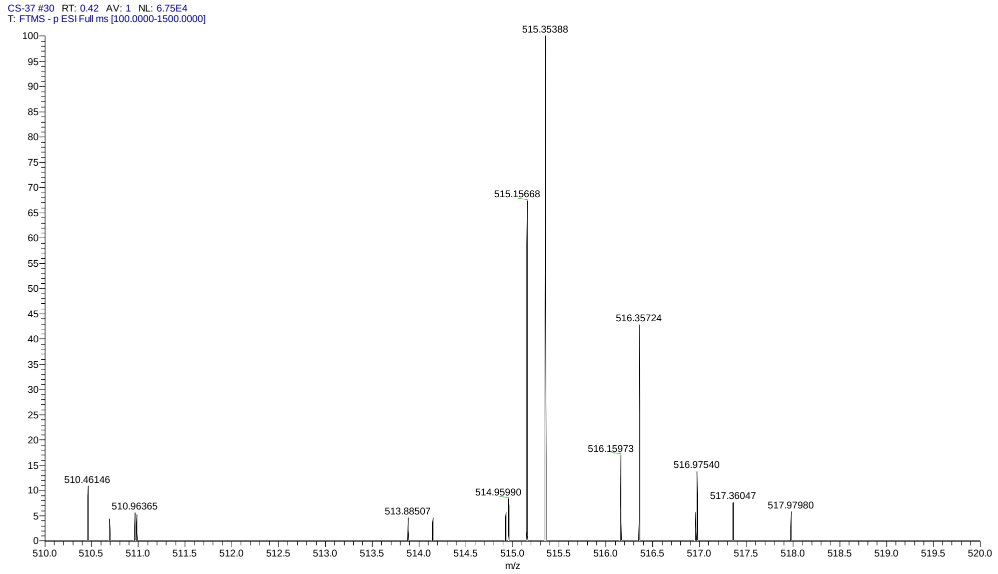

Supplementary Figure 191 HRESIMS spectrum of **19**.

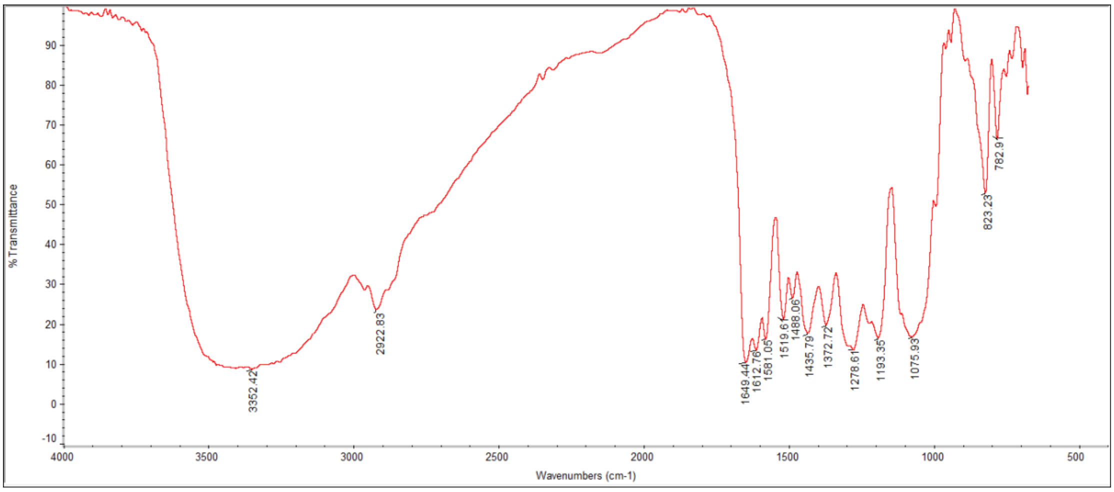

Supplementary Figure 192 IR (KBr disc) spectrum of **19**.

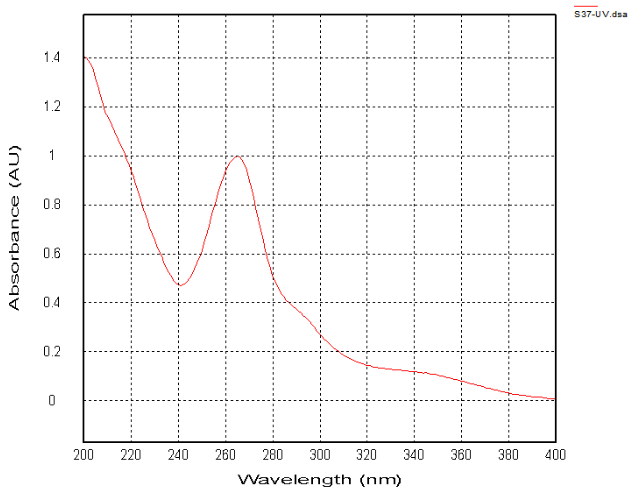

Supplementary Figure 193 UV spectrum of **19**.

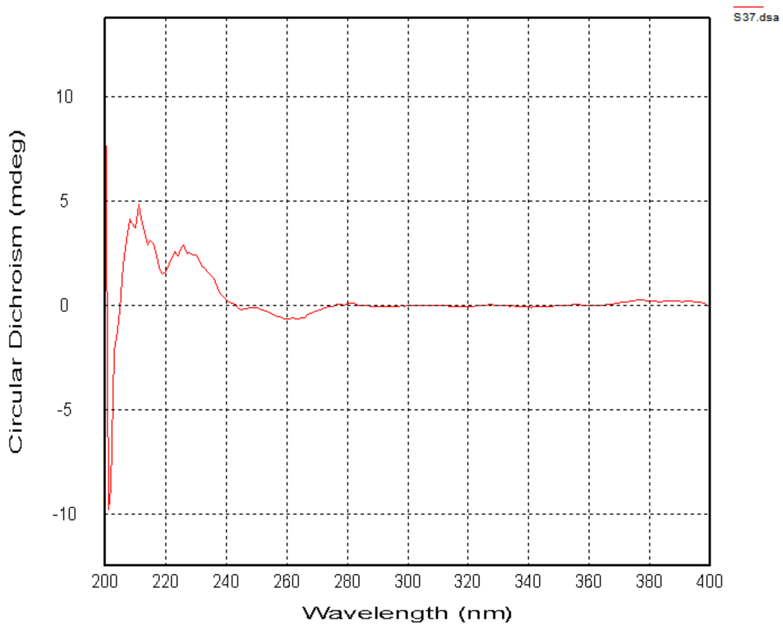

Supplementary Figure 194 ECD spectrum of **19**.

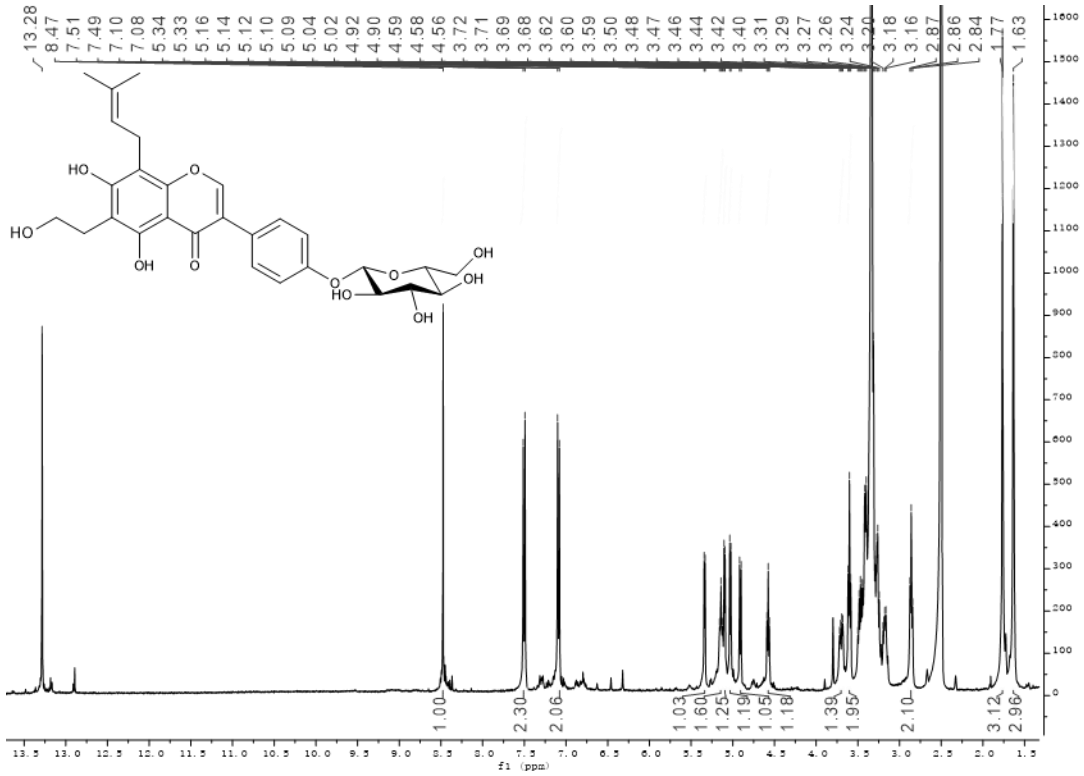

Supplementary Figure 195 ^{1}H NMR (400 MHz) spectrum of **20** in DMSO.

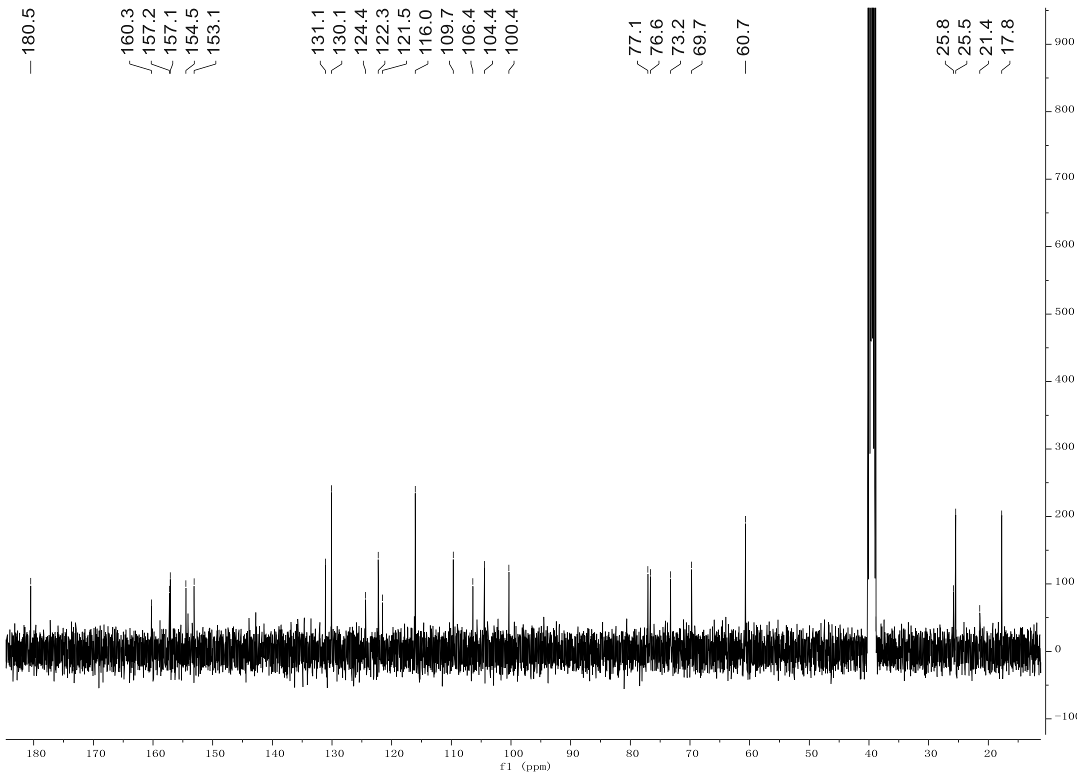

Supplementary Figure 196 ^{13}C NMR (100 MHz) spectrum of **20** in DMSO.

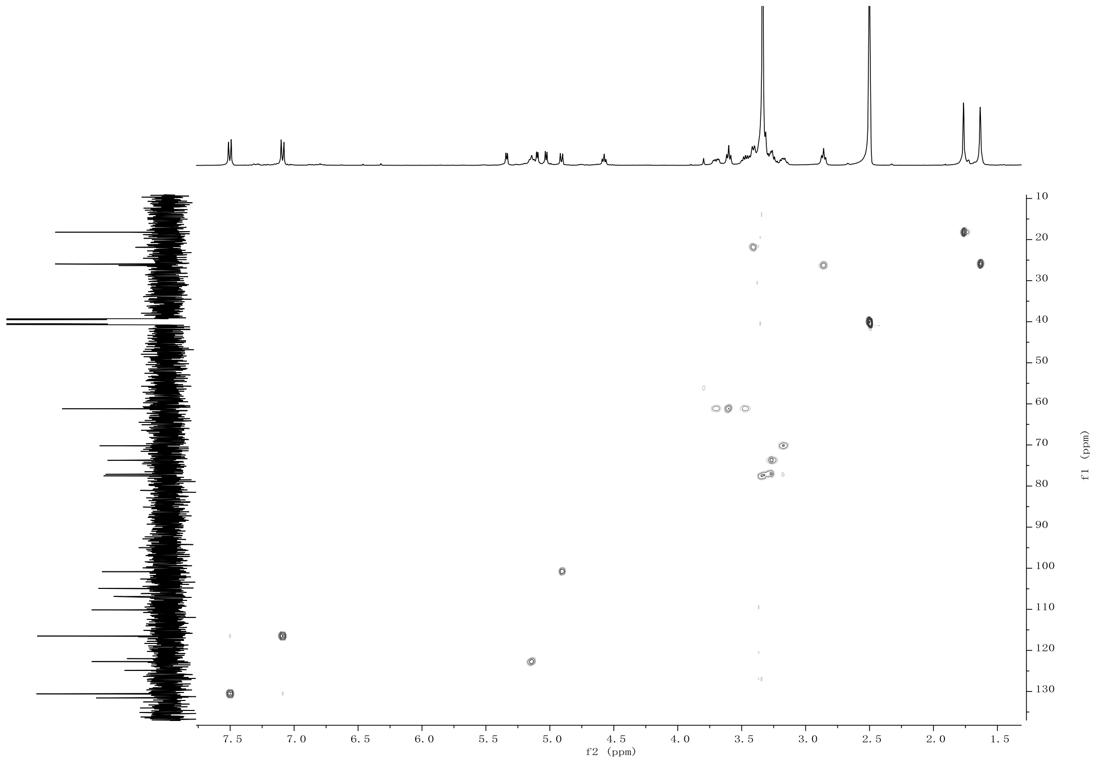

Supplementary Figure 197 HSQC (400 MHz) spectrum of **20** in DMSO.

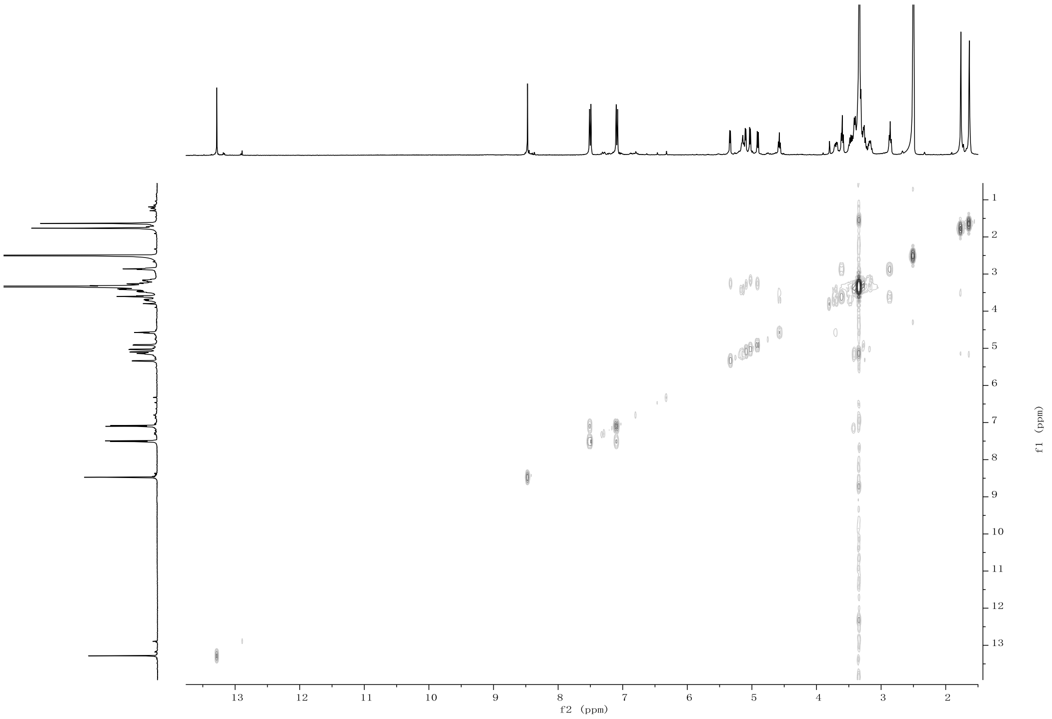

Supplementary Figure 198 ^{1}H-^{1}H COSY (600 MHz) spectrum of **20** in DMSO.

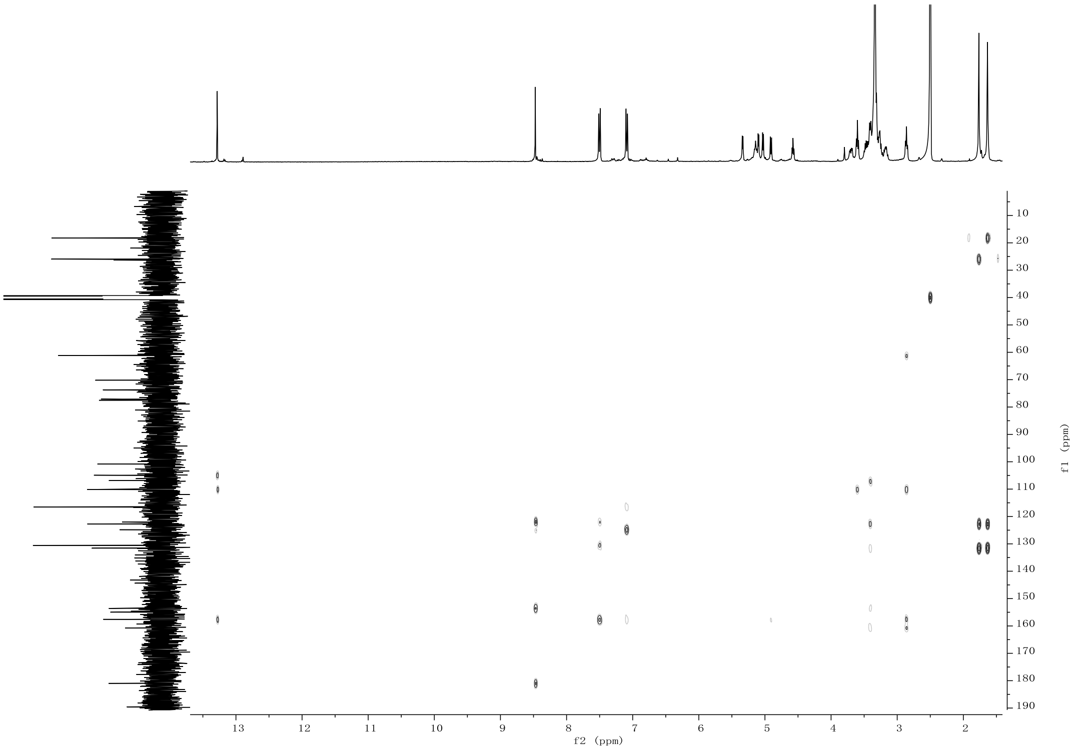

Supplementary Figure 199 HMBC (400 MHz) spectrum of **20** in DMSO.

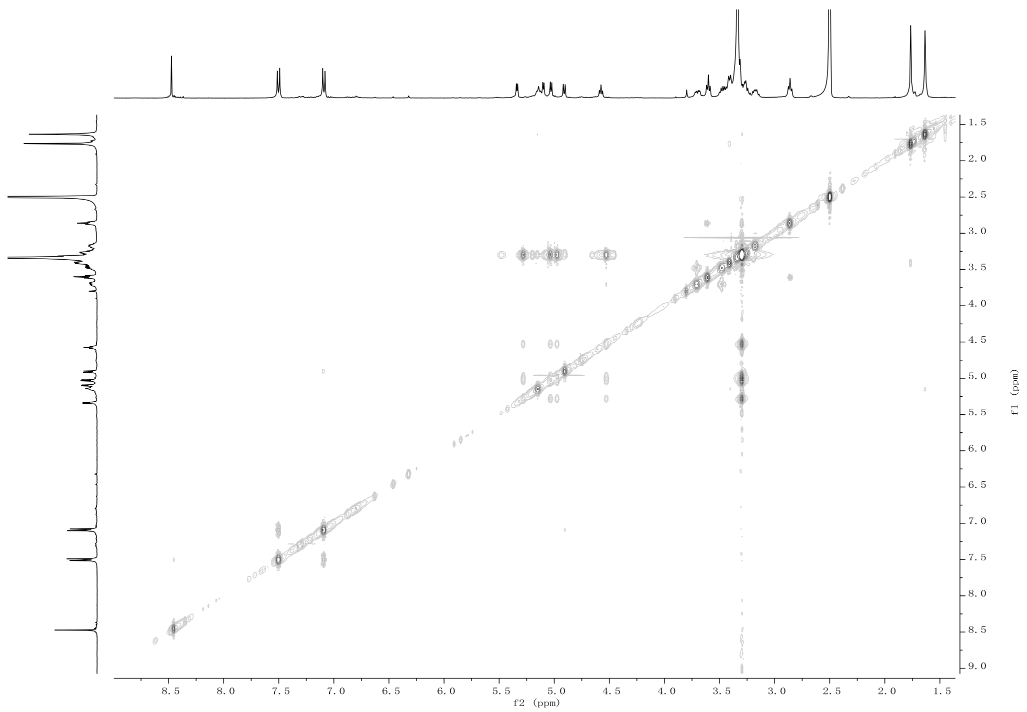

Supplementary Figure 200 ^{1}H-^{1}H NOESY (600 MHz) spectrum of **20** in DMSO.

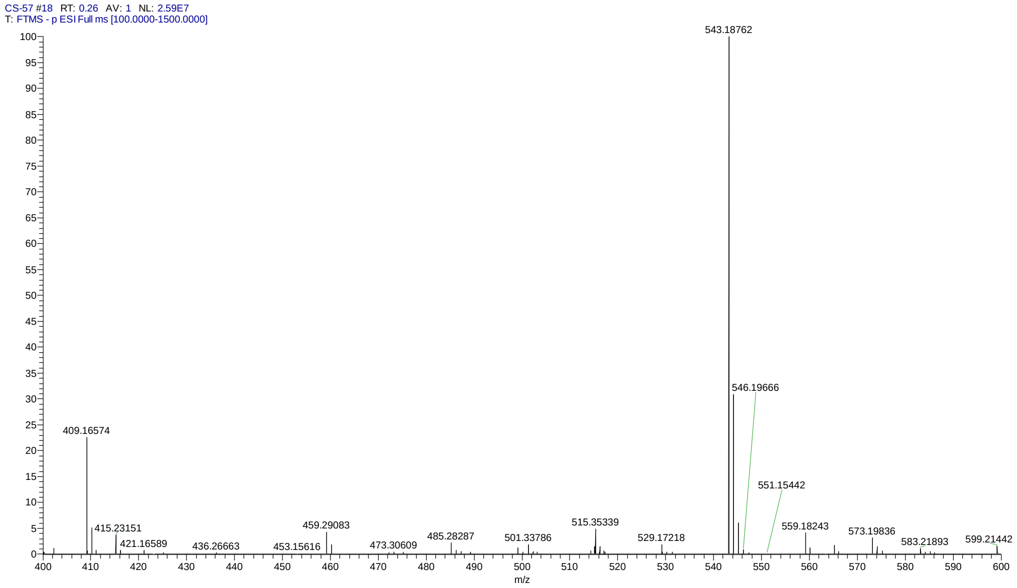

Supplementary Figure 201 HRESIMS spectrum of **20**.

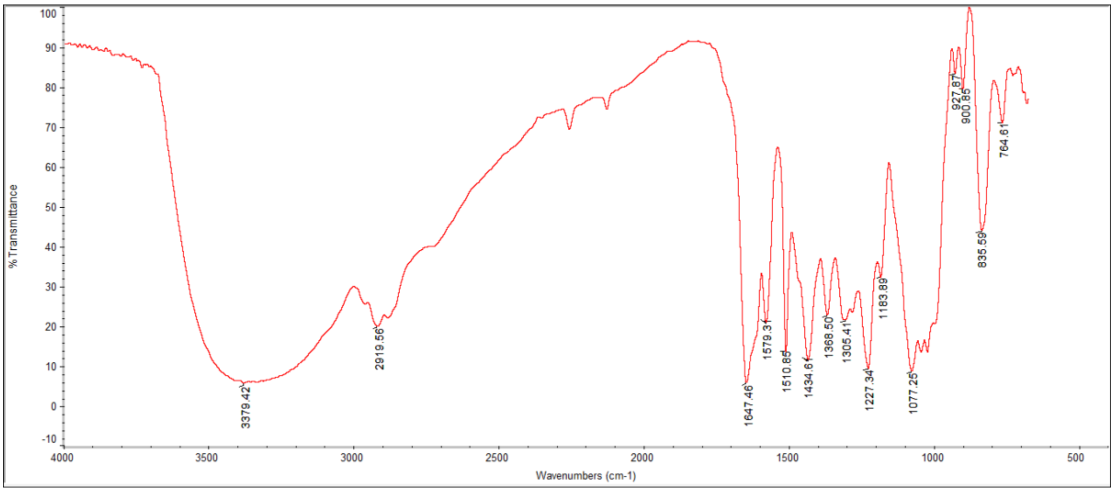

Supplementary Figure 202 IR (KBr disc) spectrum of **20**.

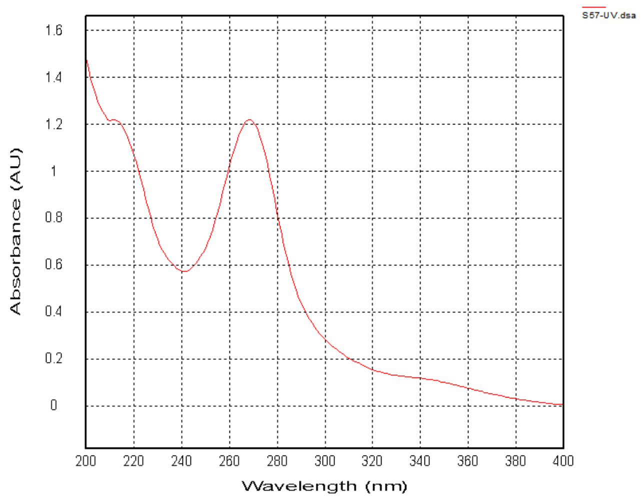

Supplementary Figure 203 UV spectrum of **20**.

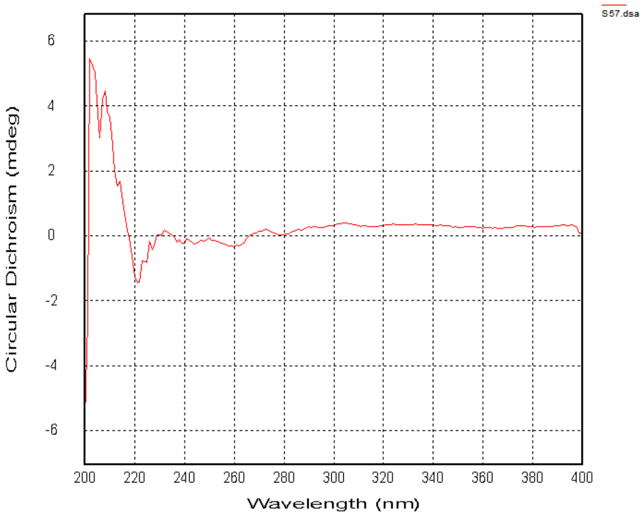

Supplementary Figure 204 ECD spectrum of **20**.

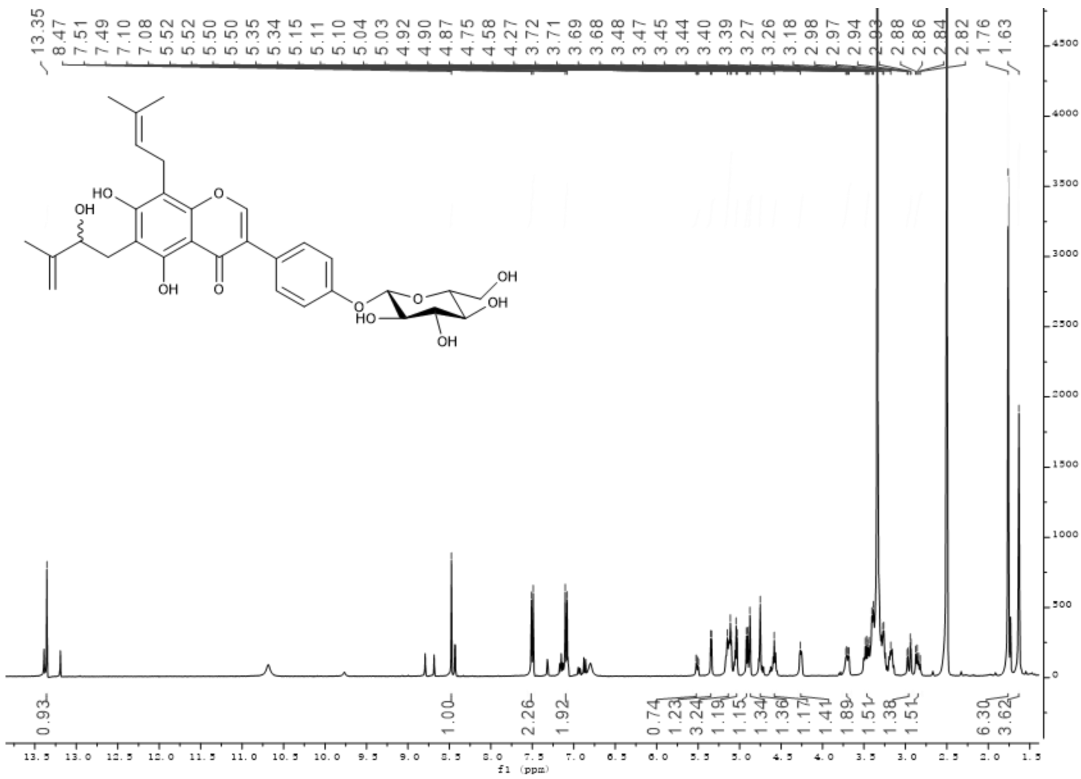

Supplementary Figure 205 ^{1}H NMR (400 MHz) spectrum of **21** in DMSO.

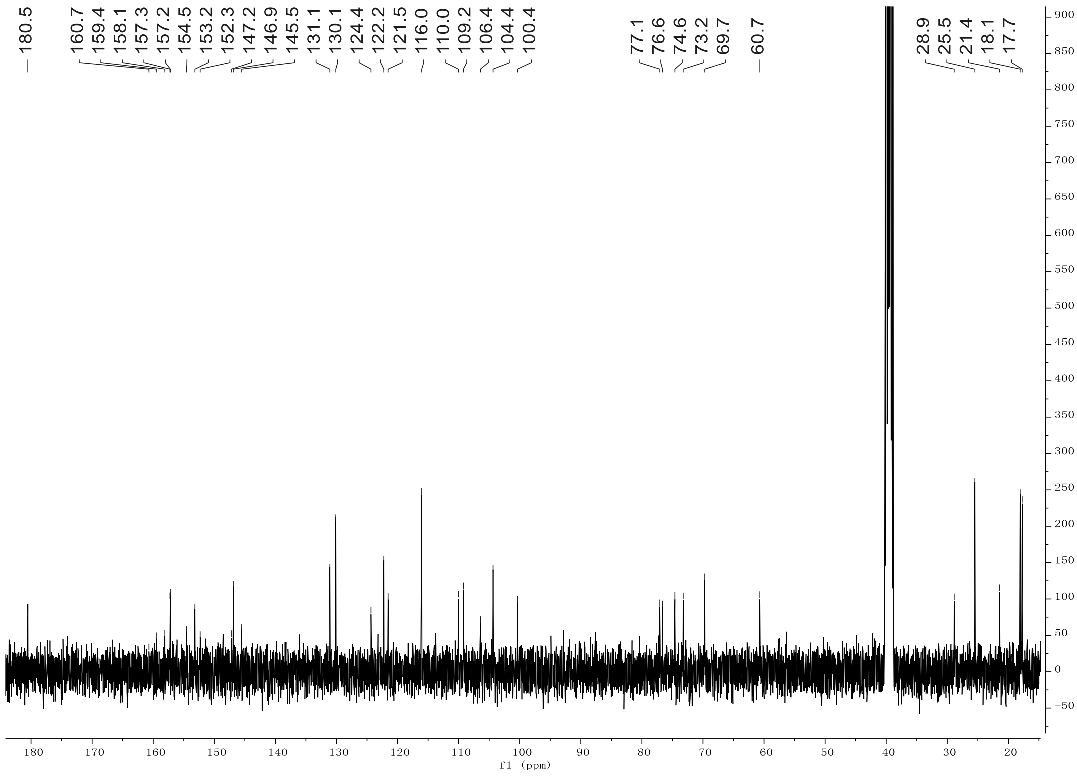

Supplementary Figure 206 ^{13}C NMR (100 MHz) spectrum of **21** in DMSO.

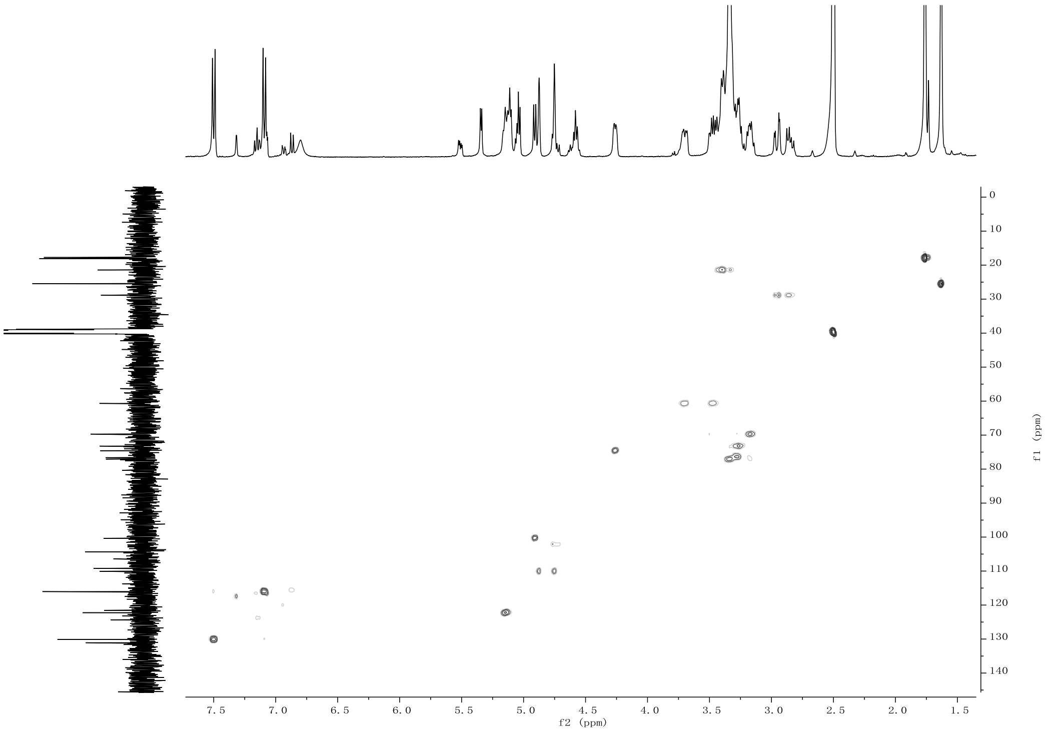

Supplementary Figure 207 HSQC (400 MHz) spectrum of **21** in DMSO.

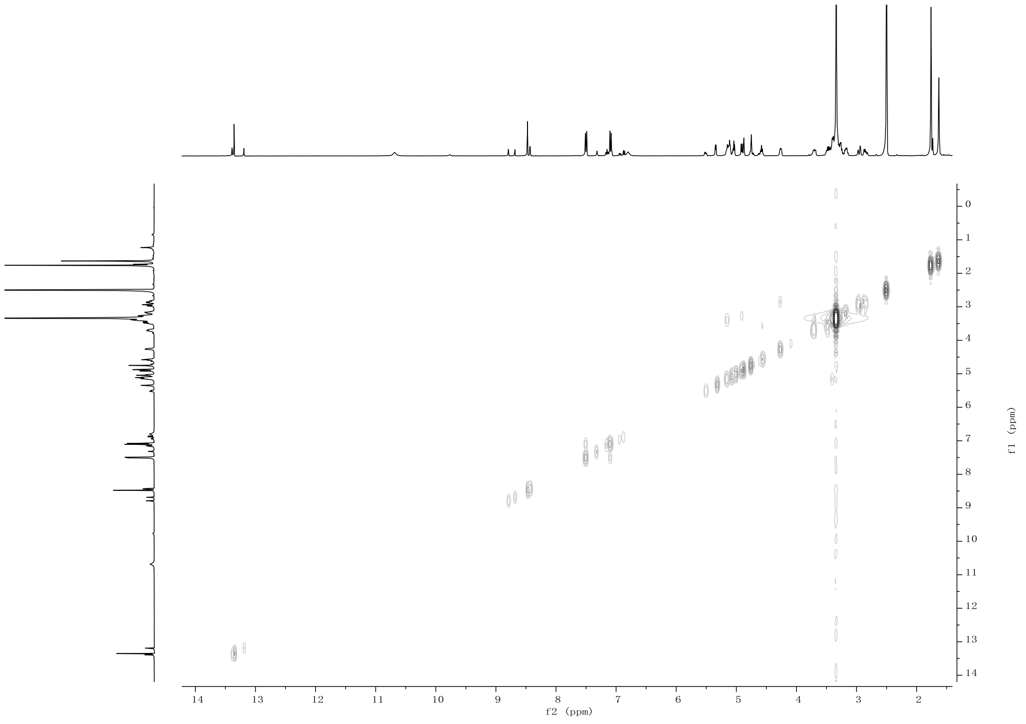

Supplementary Figure 208 ^{1}H-^{1}H COSY (600 MHz) spectrum of **21** in DMSO.

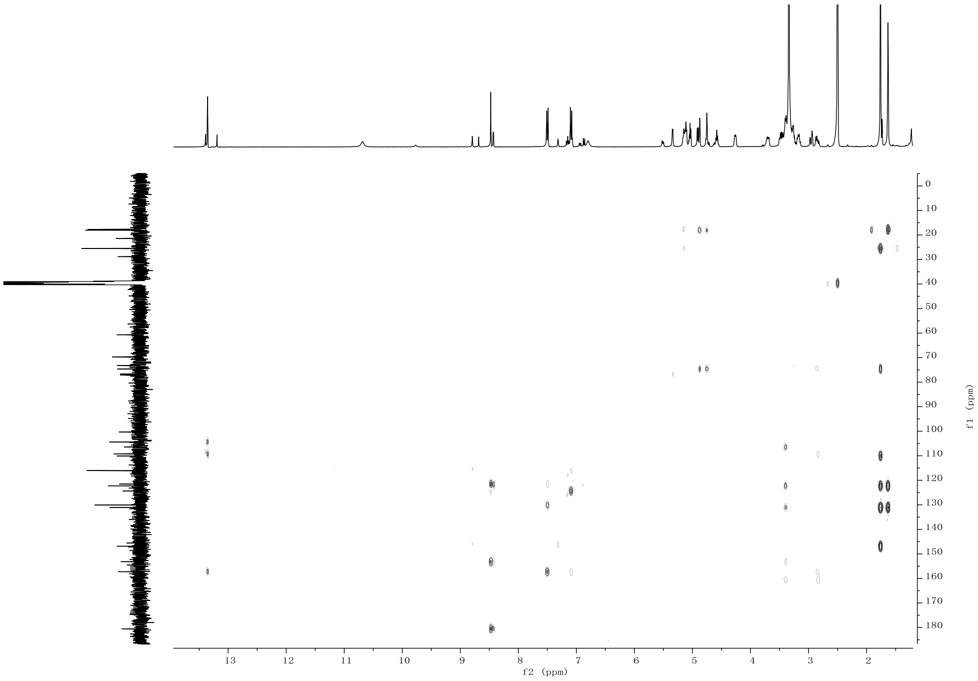

Supplementary Figure 209 HMBC (400 MHz) spectrum of **21** in DMSO.

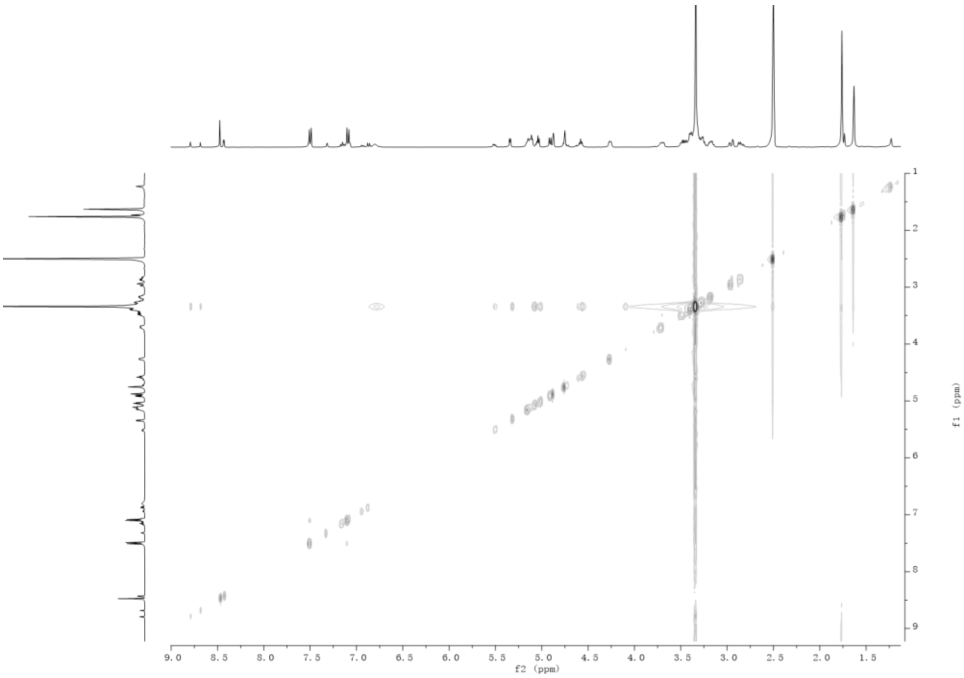

Supplementary Figure 210 ^{1}H-^{1}H NOESY (600 MHz) spectrum of **21** in DMSO.

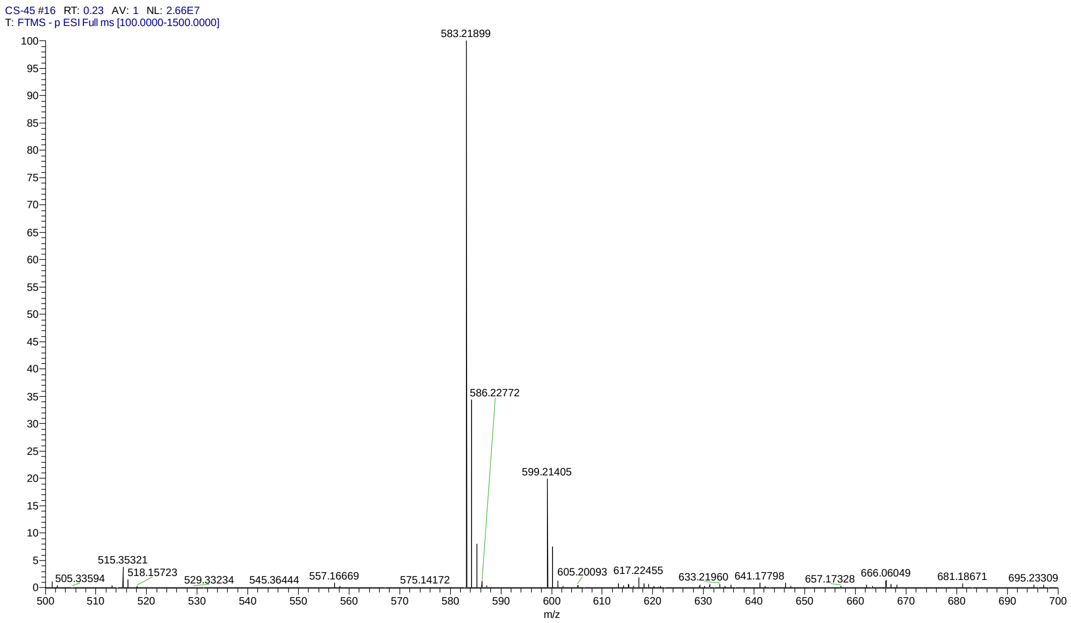

Supplementary Figure 211 HRESIMS spectrum of **21**.

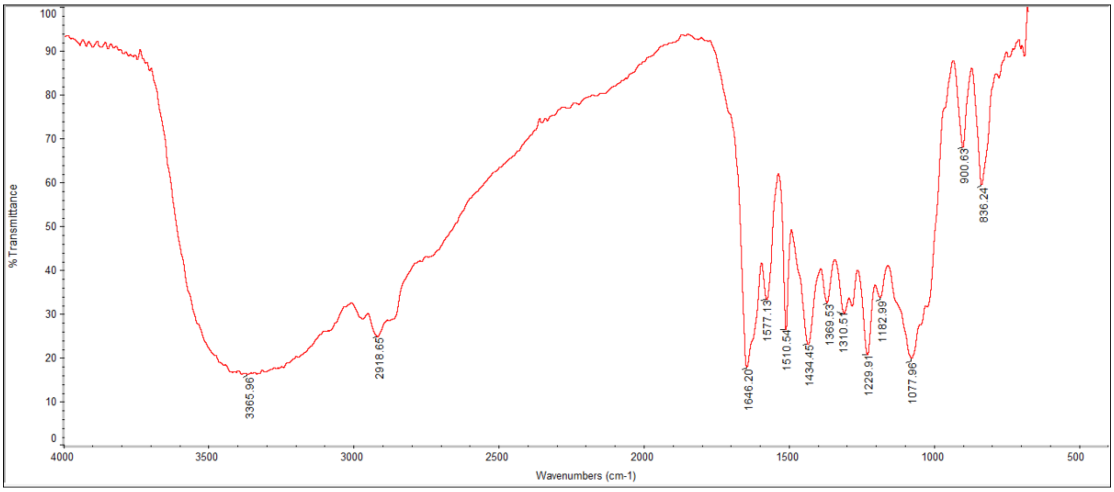

Supplementary Figure 212 IR (KBr disc) spectrum of **21**.

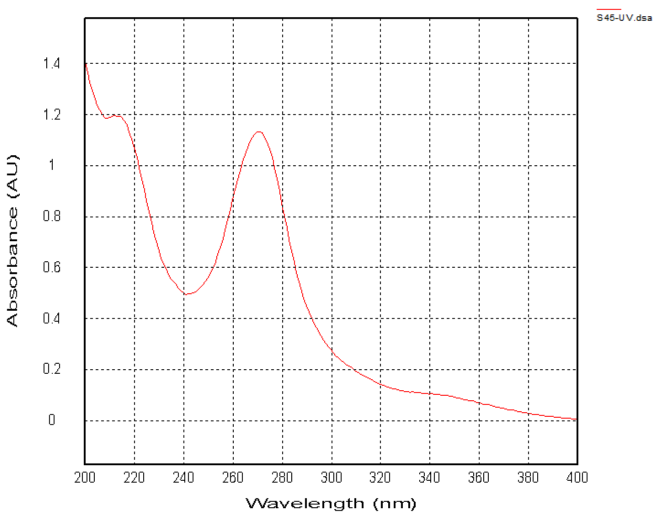

Supplementary Figure 213 UV spectrum of **21**.

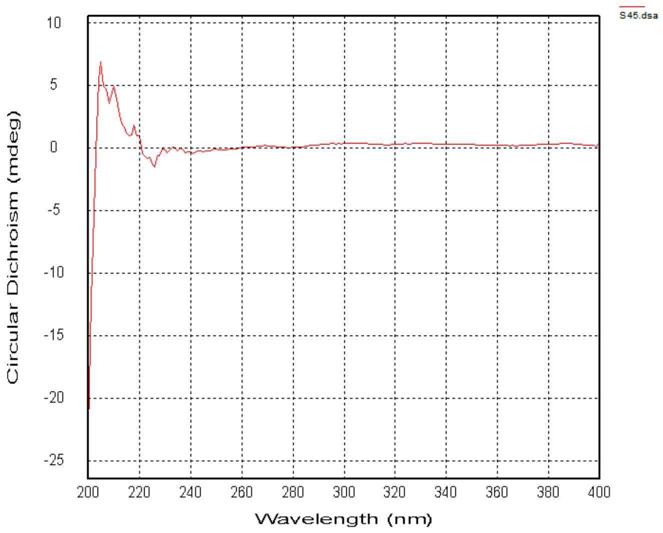

Supplementary Figure 214 ECD spectrum of **21**.

Supplementary Figure 215 Microbial biotransformation of glycosylation of 5 by *Bacillus* species in vitro by LC-MS.

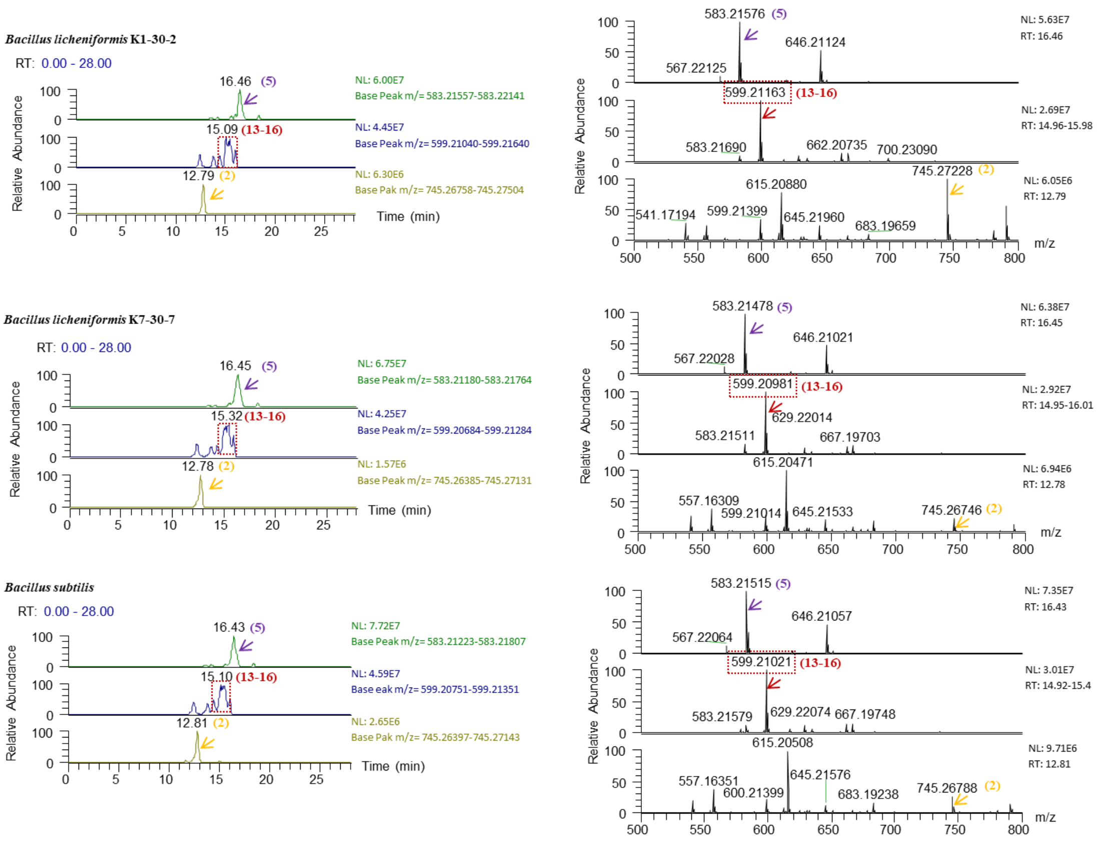

Converted products from three *Bacillus* species with silexcrin E (**5**) as substrate through microbial transformation in vitro detected by LC-MS with retention time and deprotonated molecular ion peaks.

Supplementary Figure 216 Microbial biotransformation of silkworm gut strains in vitro.

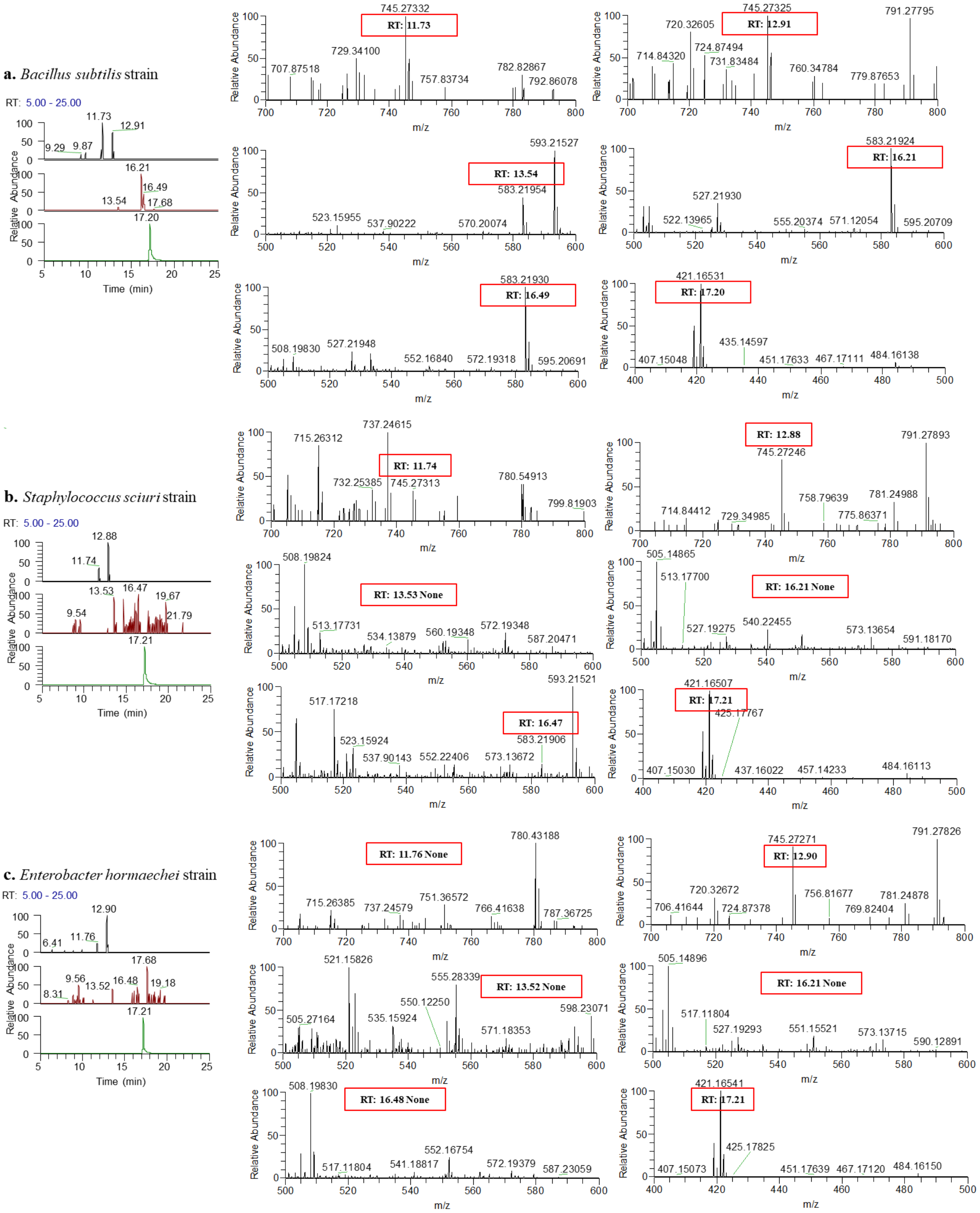

Microbial biotransformation by three silkworm intestinal strains, *B. subtilis* (a), *S. sciuri* (b) and *E. hormaechei* (c) of DPL (**24**) in vitro.

Supplementary Figure 217 Comparative cocoons and silk from mulberry leaves (MLs)-fed silkworms and CTLs-fed silkworms.

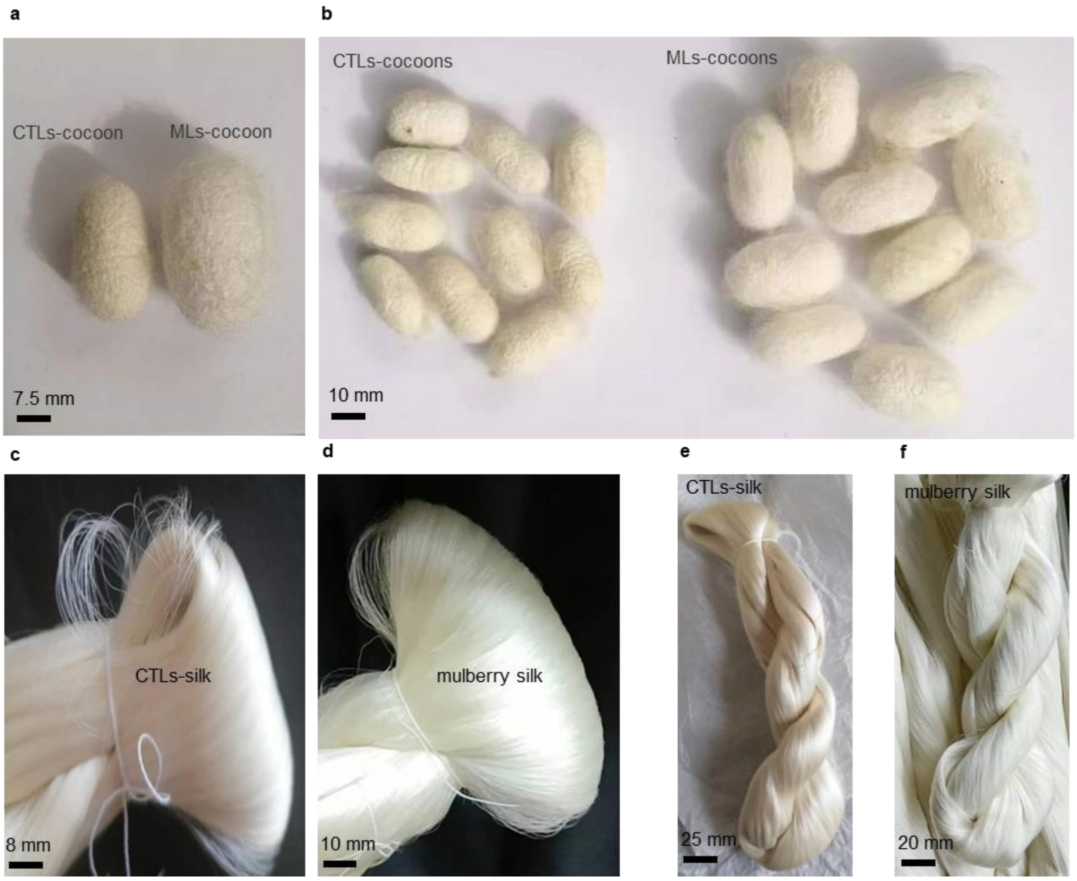

a Comparison of individual cocoon sizes. The MLs-cocoon was larger in size than the CTLs-cocoon. **b** Comparison of MLs-cocoons and CTLs-cocoons in size. **c-f** Comparison of mulberry silk and CTLs-silk in different quality. CTLs-silk was tougher and thicker, and mulberry silk was more fine, white and soft[16]. Data and pictures are provided from our co-authors, J.Z. and J.S.. Reproduced with permission from Supplementary Reference16, reprinted with permission from Guangdong Canye. Reprints and permissions information is available according to Copyright Law of the People's Republic of China.

Supplementary Figure 218 Standard curves of compounds 1-5 and 24.

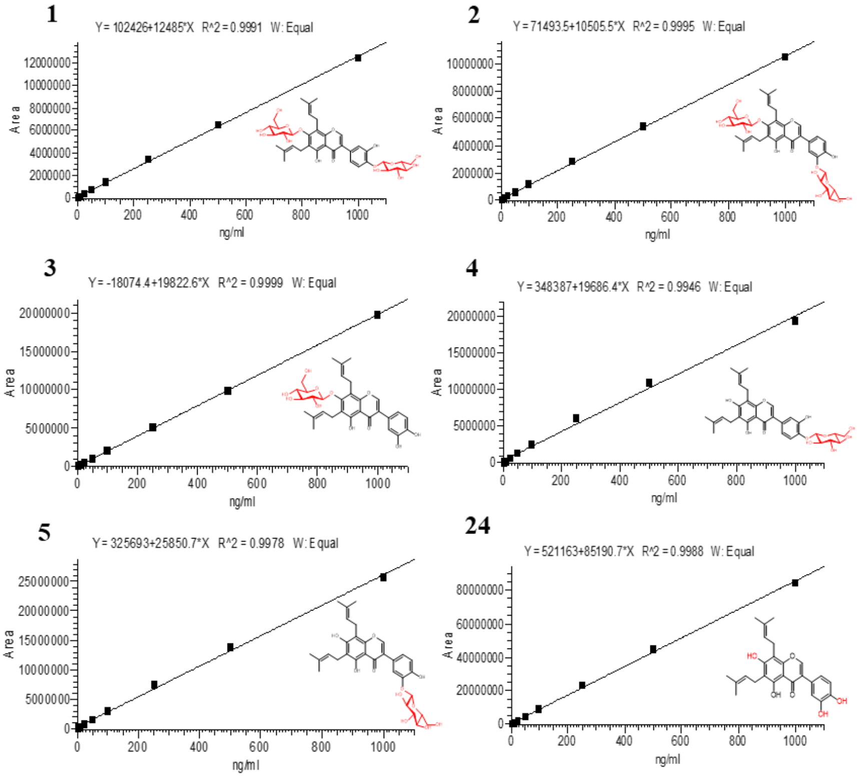

The standard curves of compounds **1-5** and **24** were obtained by HPLC-MS.

Supplementary References

1. Hano. Y, Nomura. T. Constituents of the Chinese crude drug "Sang Bai Pi" (*Morus* root barks). IV. Structures of four new flavonoids, sanggenon H, I, J and K. *Heterocycles* **20**, 1071-1076 (1983).
2. Taylor RB, Corley DG, Tempesta M. 2,3-dihydroauriculatin, a prenylated isoflavanone from Erythrina senegalensis. application of the selective INEPT technique. . *J Nat Prod* **49**, 670-673 (1986).
3. Yang SS, Gao F, Mabry TJ*, et al.* Flavonoids from Lotus creticus. *Phytochemlstr* **28**, 1749-1750 (1989).
4. Fukai T, Wang QH, Takayama M*, et al.* Structures of five new prenylated flavonoids, Gancaonins L, M, N, O and P from parts of Glycyrrhiza uralensis. *Heterocycles* **31**, 373-382 (1990).
5. Singhal AK, Shanrma RP, Thyagarajan G*, et al.* New prenylated isoflavones and a prenylated dihydroflavonol from Miliettia pachycarpa. *Phytochemistry* **19**, 929-934 (1980).
6. Ito C, Murata T, Itoigawa M*, et al.* Induction of apoptosis by isoflavonoids from the leaves of Millettia taiwaniana in human leukemia HL-60 cells. *Planta Med* **72**, 424-429 (2006).
7. Ito C, Itoigawa M, Kumagaya M*, et al.* Isoflavonoids with Antiestrogenic Activity from Millettia pachycarpa. *J Nat Prod* **69**, 138-141 (2006).
8. Li H, Yang M, Miao J*, et al.* Prenylated isoflavones from Flemingia philippinensis. *Magn Reson Chem* **46**, 1203-1207 (2008).
9. Al-Maharik N, Botting NP. Synthesis of lupiwighteone via a para-Claisen–Cope rearrangement. *Tetrahedron* **59**, 4177-4181 (2003).
10. Ji S, Liang W-F, Li Z-W*, et al.* Efficient and selective glucosylation of prenylated phenolic compounds by Mucor hiemalis. *RSC Advances* **6**, 20791-20799 (2016).
11. Pistelli L, Bertoli A, Giachi I*, et al.* Flavonoids from Genista ephedroides. *J Nat Prod* **61**, 1404-1406 (1998).
12. Maximo P, Lourenco A, Feio SS*, et al.* Flavonoids from Ulex airensis and Ulex europaeus ssp. europaeus. *J Nat Prod* **65**, 175-178 (2002).
13. Sato M, Tanaka H, Tani N*, et al.* Different antibacterial actions of isoflavones isolated from Erythrina poeppigiana against methicillin-resistant Staphylococcus aureus. *Lett Appl Microbiol* **43**, 243-248 (2006).
14. Abdel-Kader MS, Amer ME, Tang S*, et al.* Two new isoflavone derivatives from the roots of an Egyptian collection of Lotus polyphyllos. *Nat Prod Res* **20**, 922-926 (2006).
15. Sekinea T, Inagakia M, Ikegamia F*, et al.* Six diprenylisoflavones, derrisisoflavones A-F, from Derris scandens. *Phytochemistry* **52** 87-94 (1999).
16. Zhao JC, Sang JF, Liu WM, *et al*. A study on the feeding experiment of *Cudrania tricuspidate* (Carr). Bur. all-age silkworm and its silk characteristics. *Guangdong Canye* **55**, 4-6+13 (2021).
